# Supplementary figures and images for: Identification of KIF4A as a pan-cancer diagnostic and prognostic biomarker via bioinformatics analysis and validation in osteosarcoma cell lines (part 1 of 2)
Source: PeerJ. 2021 May 21;9:e11455. doi: 10.7717/peerj.11455 (PMC8142929; doi:10.7717/peerj.11455)

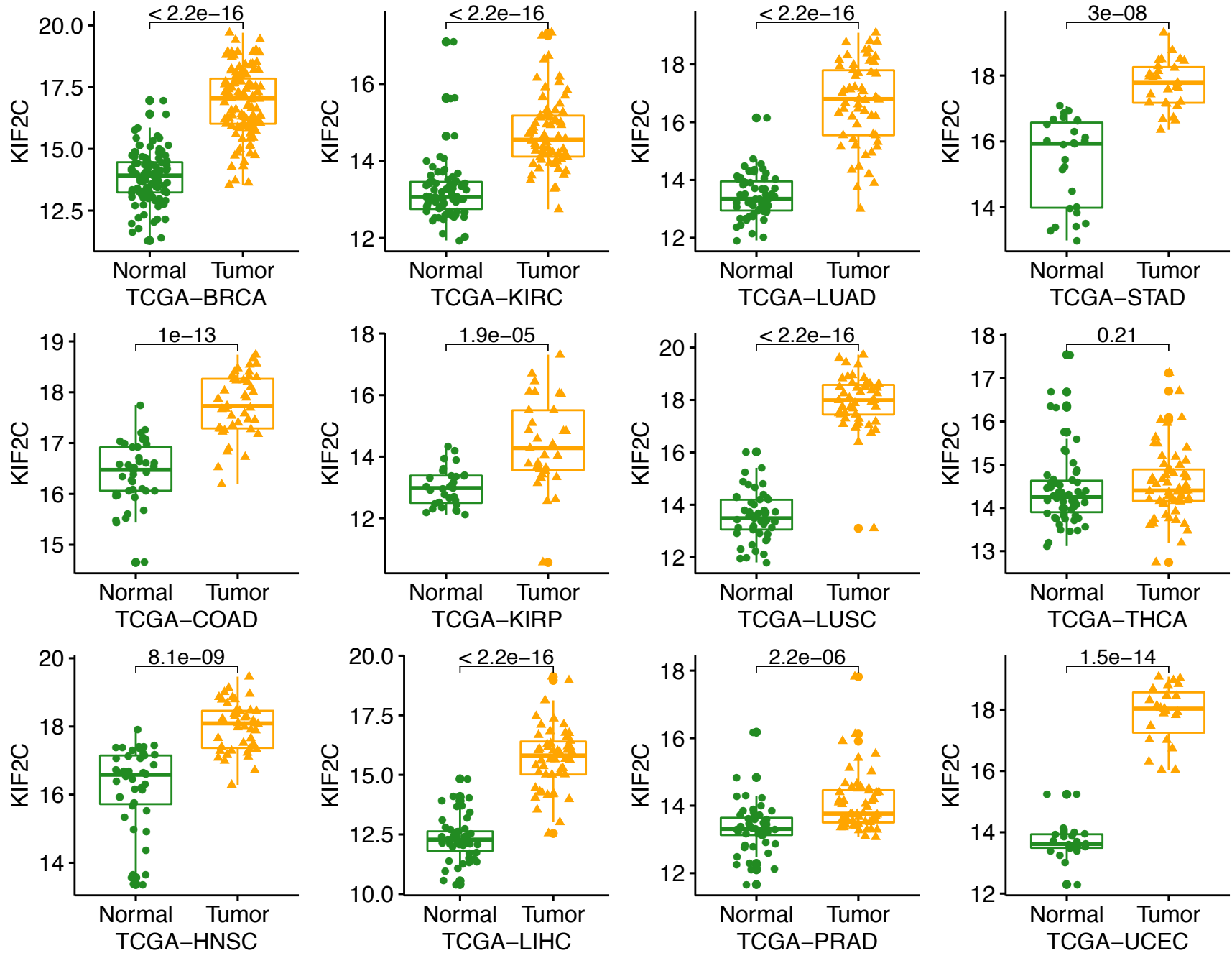

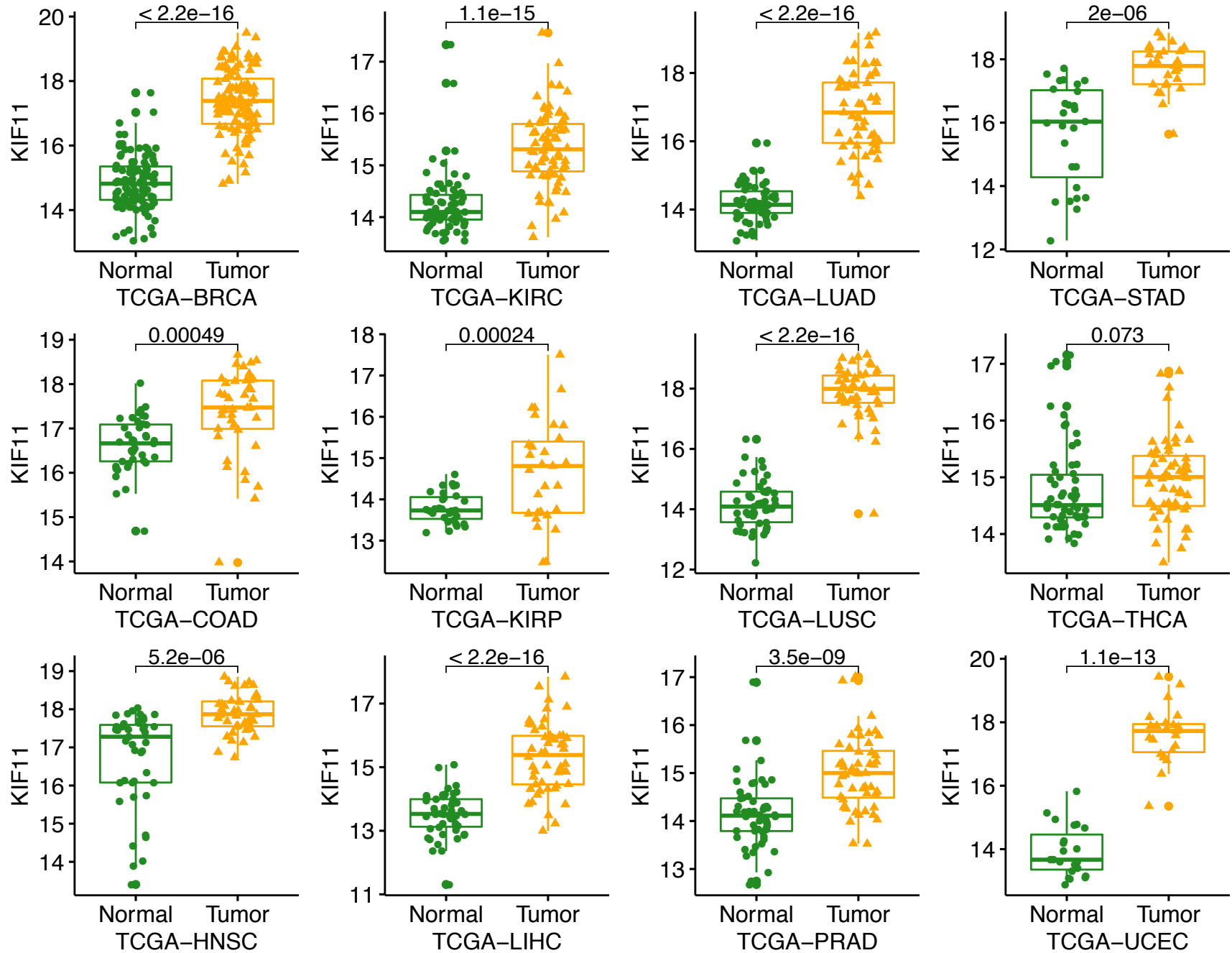

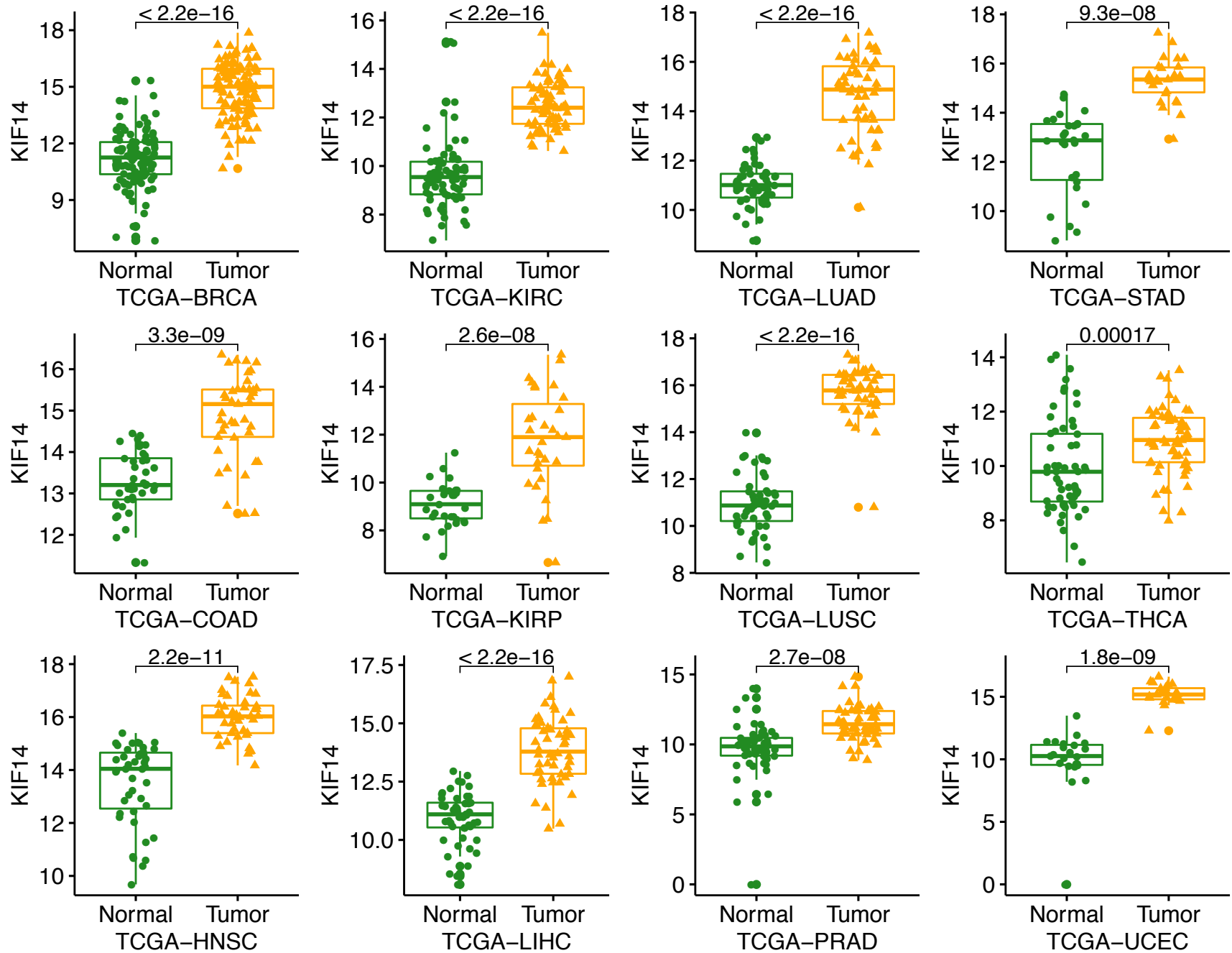

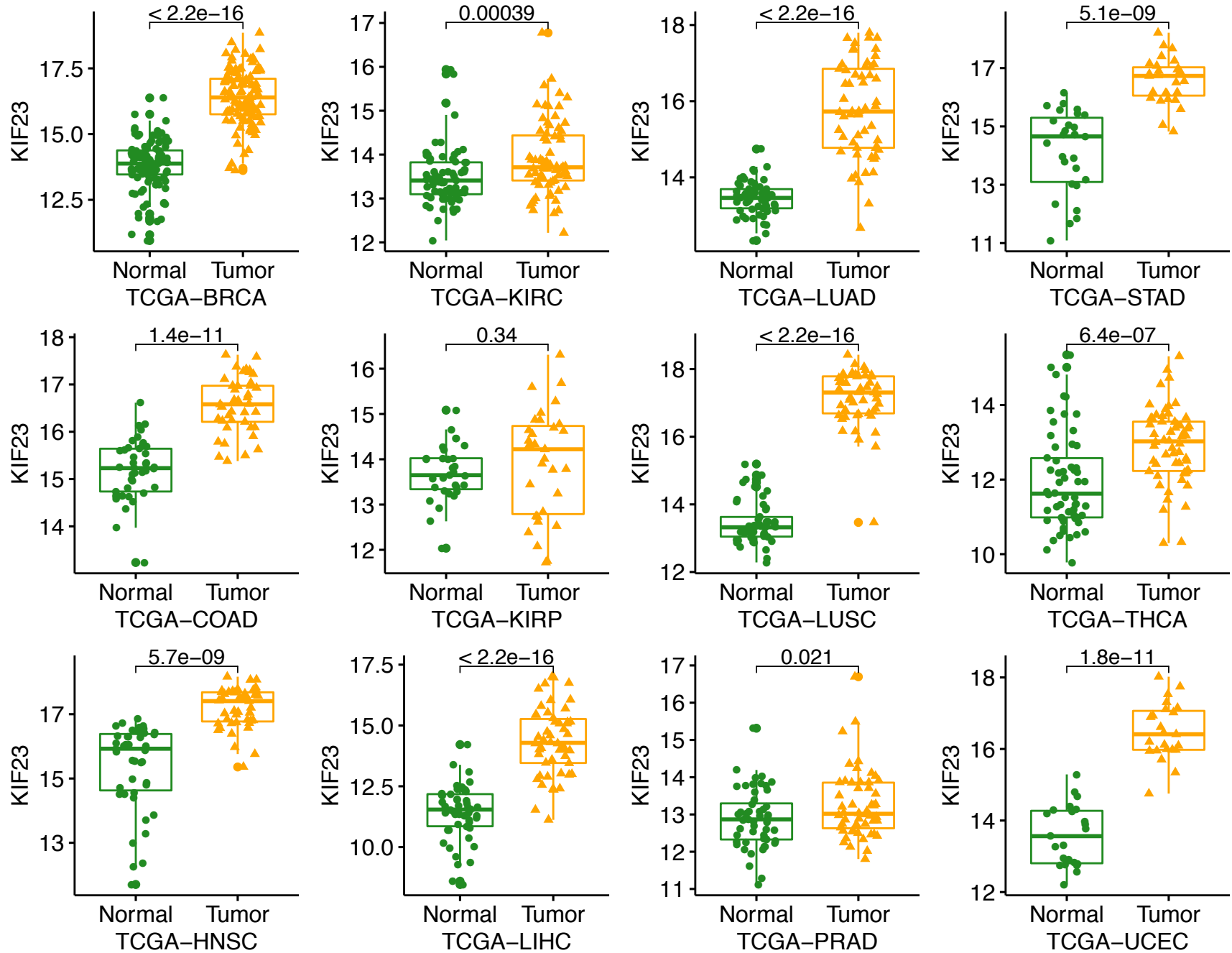

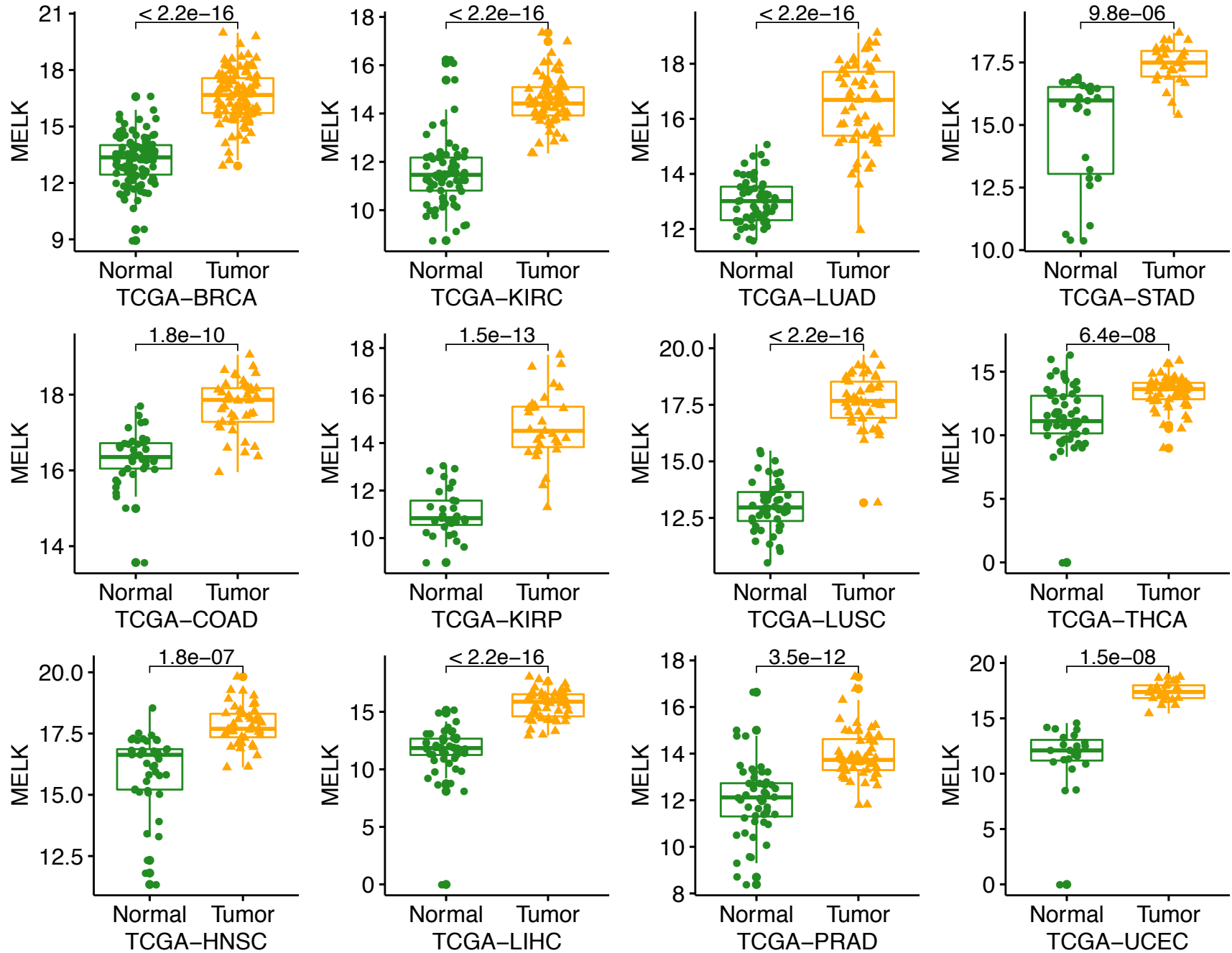

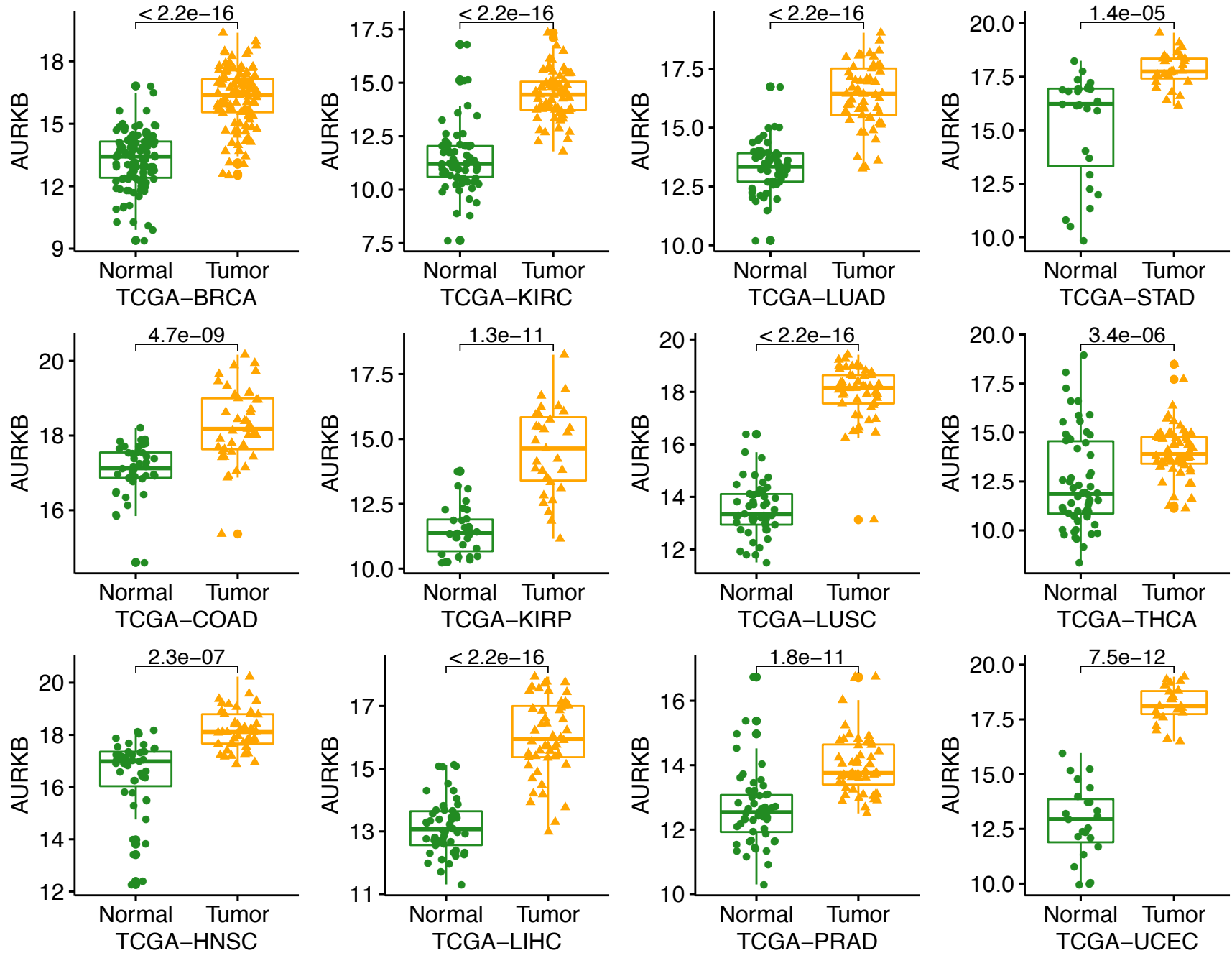

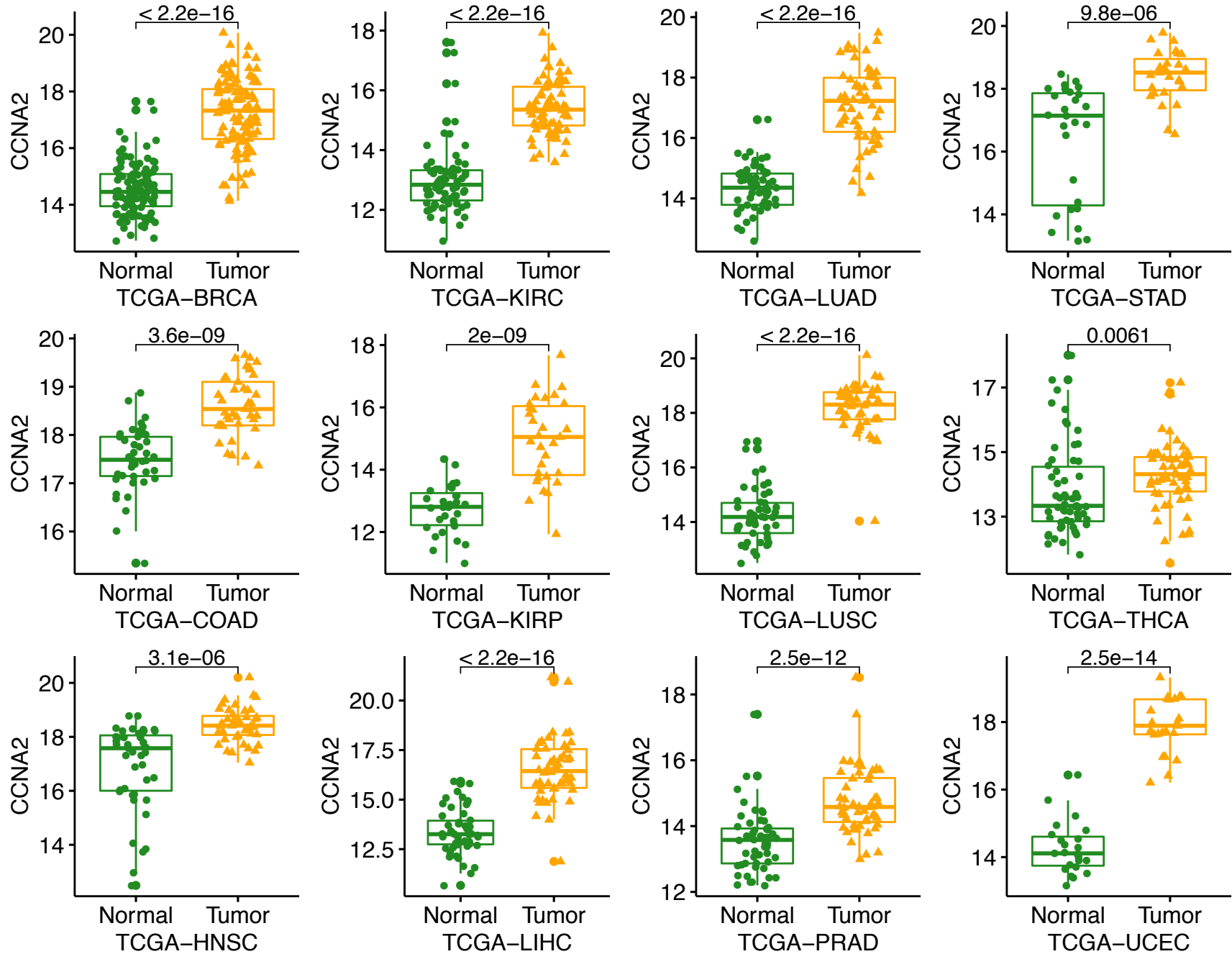

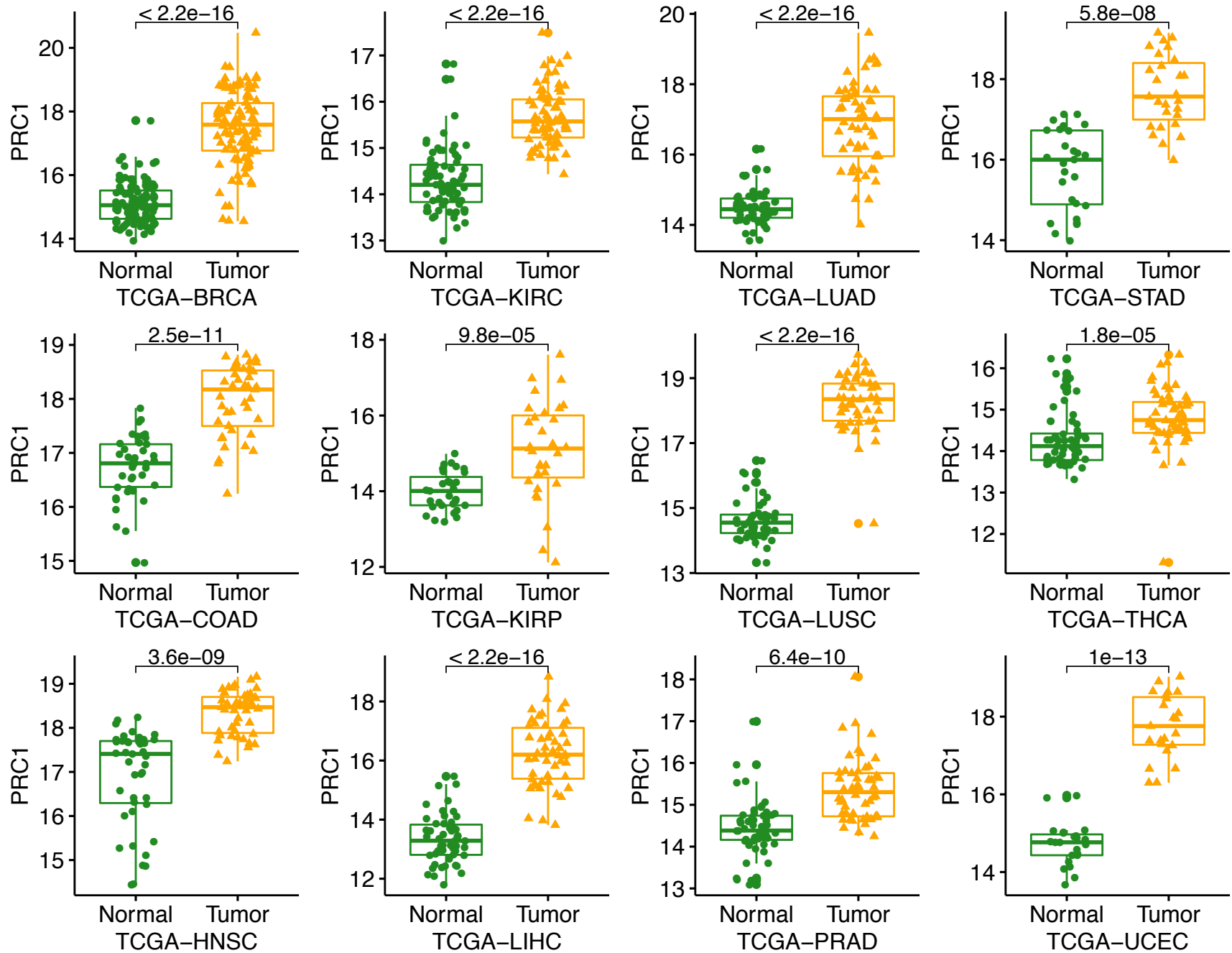

Supplement: Supplemental Information 1 [file peerj-09-11455-s001.pdf]

**
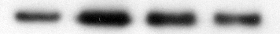
** KIF4A


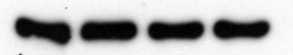
 actin

hFOB1.19、mg63、U-20S、Hos

| 编号 | hFOB | mg63 | U-20S | Hos |
| --- | --- | --- | --- | --- |
| KIF4A | 19.10 | 70.85 | 51.85 | 30.46 |
| actin | 115.86 | 119.09 | 112.82 | 101.84 |
| KIF4A/actin | 0.16 | 0.59 | 0.46 | 0.30 |

Supplement: Supplemental Information 4 [file peerj-09-11455-s004.zip › fig2B-WB/Gray value.docx]

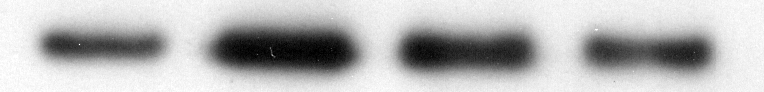

Supplement: Supplemental Information 4 [file peerj-09-11455-s004.zip › fig2B-WB/KIF4A-1.tif]

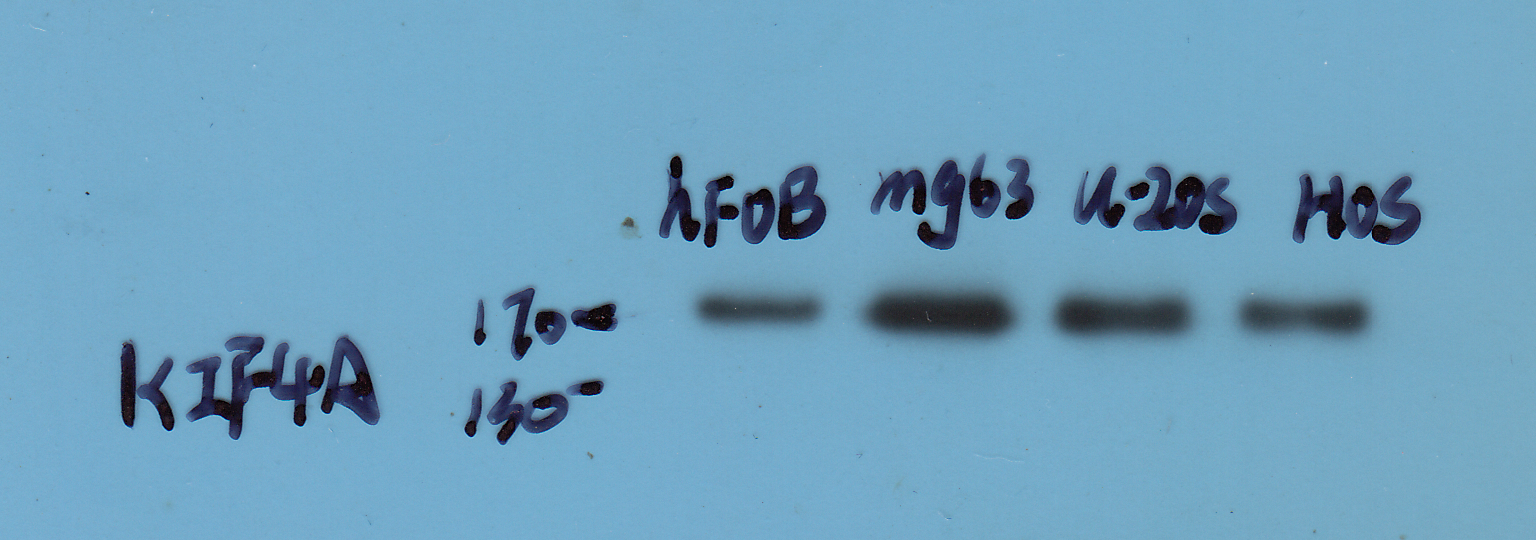

Supplement: Supplemental Information 4 [file peerj-09-11455-s004.zip › fig2B-WB/KIF4A.tif]

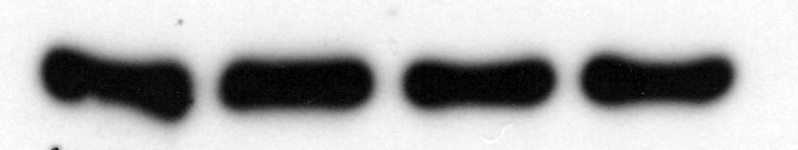

Supplement: Supplemental Information 4 [file peerj-09-11455-s004.zip › fig2B-WB/β-actin-1.tif]

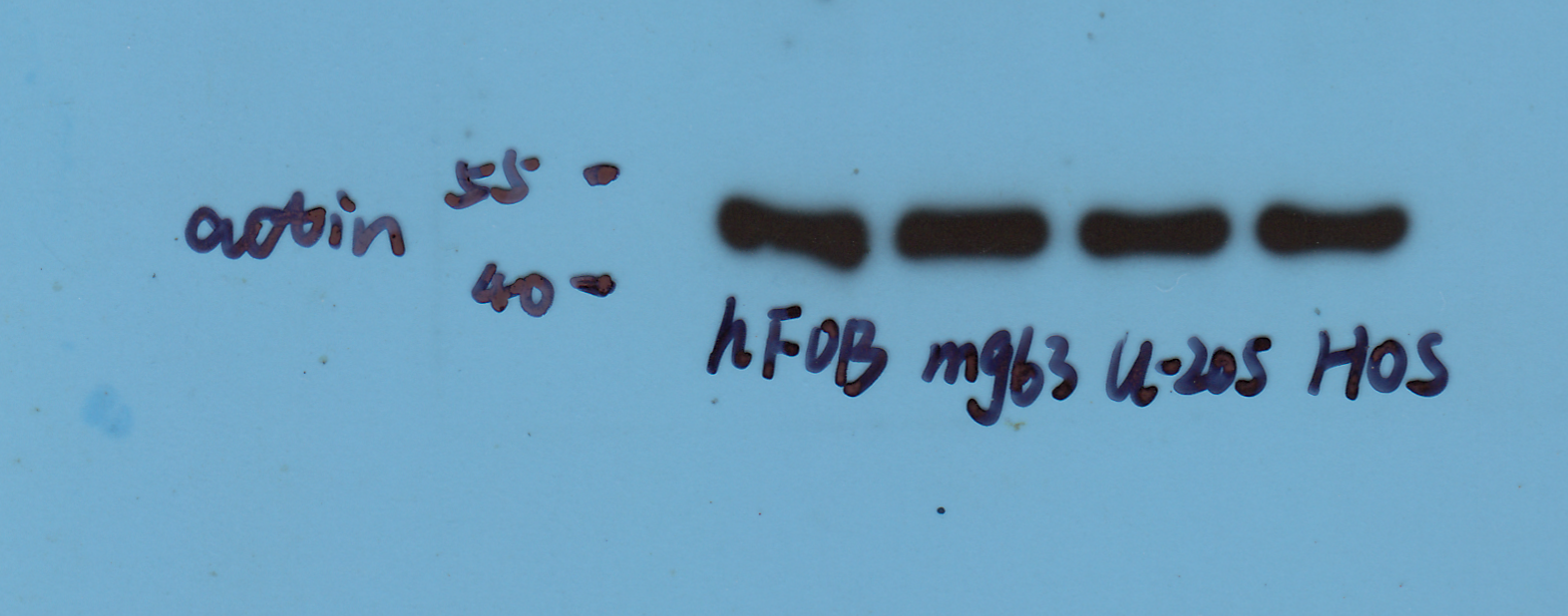

Supplement: Supplemental Information 4 [file peerj-09-11455-s004.zip › fig2B-WB/β-actin.tif]

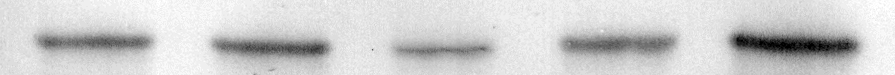

Supplement: Supplemental Information 6 [file peerj-09-11455-s006.zip › fig3B-WB/MG63-KIF4A-1.tif]

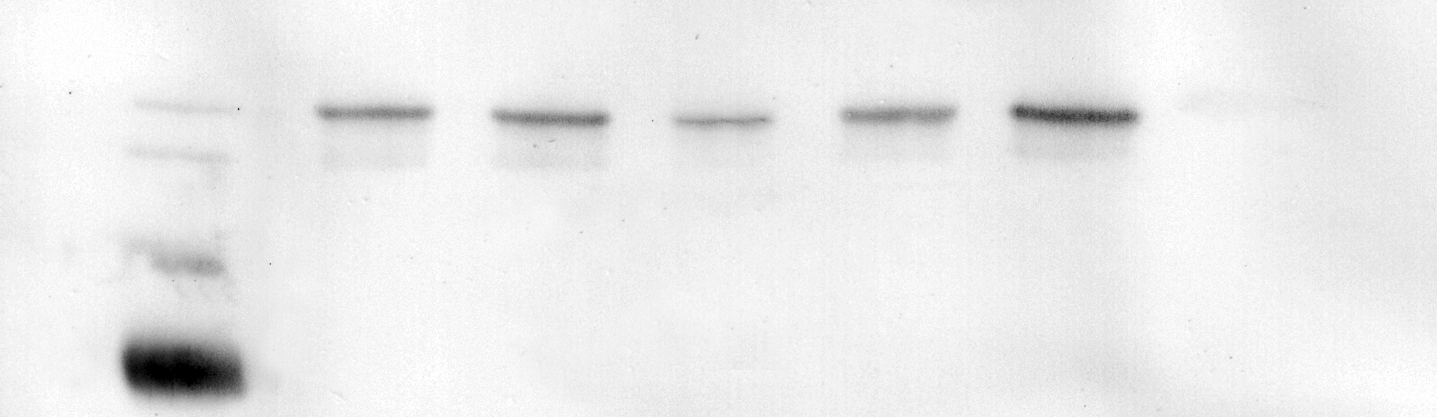

Supplement: Supplemental Information 6 [file peerj-09-11455-s006.zip › fig3B-WB/MG63-KIF4A.tif]

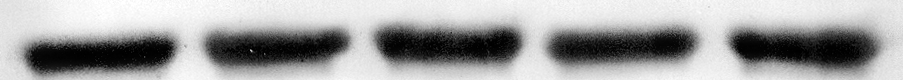

Supplement: Supplemental Information 6 [file peerj-09-11455-s006.zip › fig3B-WB/MG63-β-actin-1.tif]

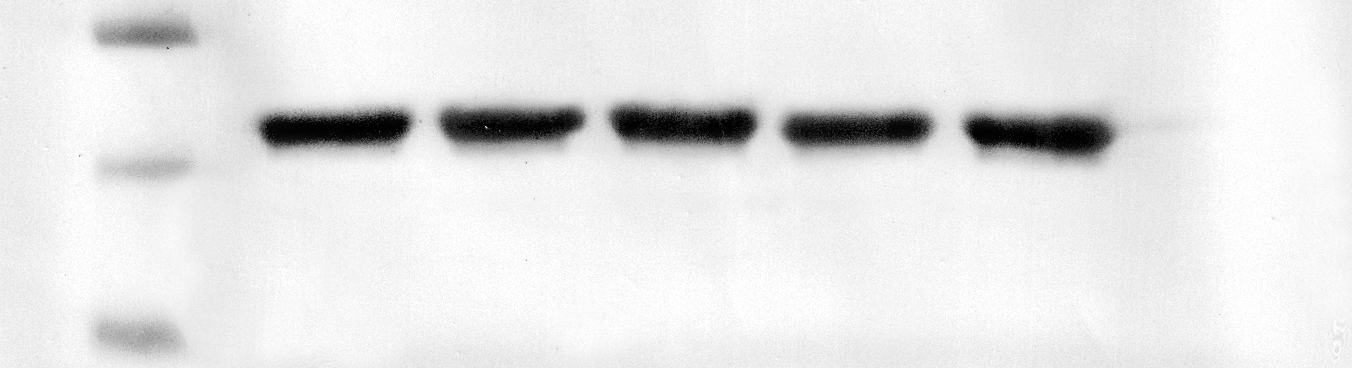

Supplement: Supplemental Information 6 [file peerj-09-11455-s006.zip › fig3B-WB/MG63-β-actin.tif]

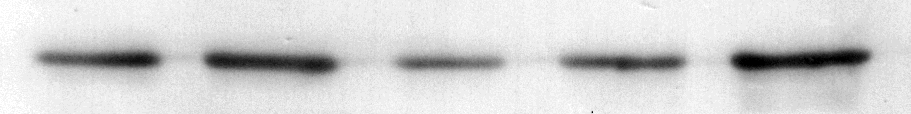

Supplement: Supplemental Information 6 [file peerj-09-11455-s006.zip › fig3B-WB/U20S-KIF4A-1.tif]

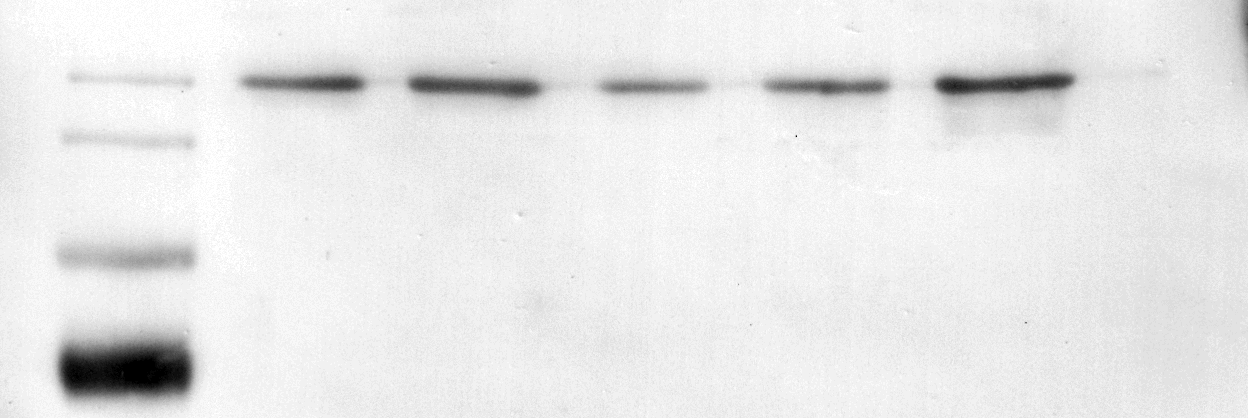

Supplement: Supplemental Information 6 [file peerj-09-11455-s006.zip › fig3B-WB/U20S-KIF4A.tif]

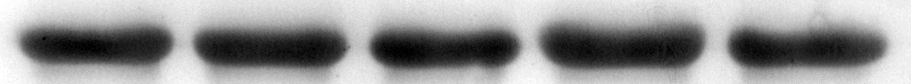

Supplement: Supplemental Information 6 [file peerj-09-11455-s006.zip › fig3B-WB/U20S-β-actin-1.tif]

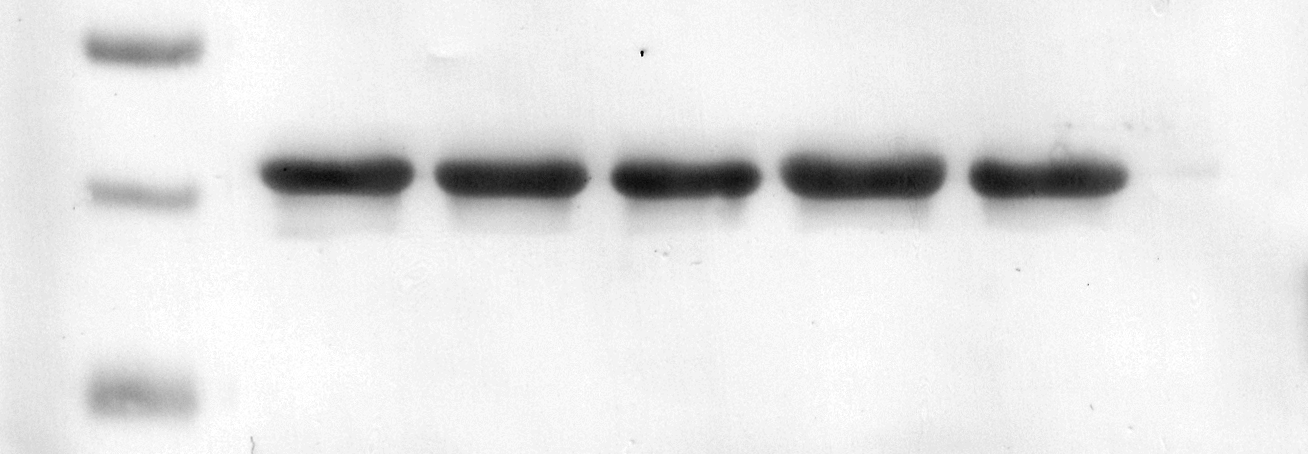

Supplement: Supplemental Information 6 [file peerj-09-11455-s006.zip › fig3B-WB/U20S-β-actin.tif]

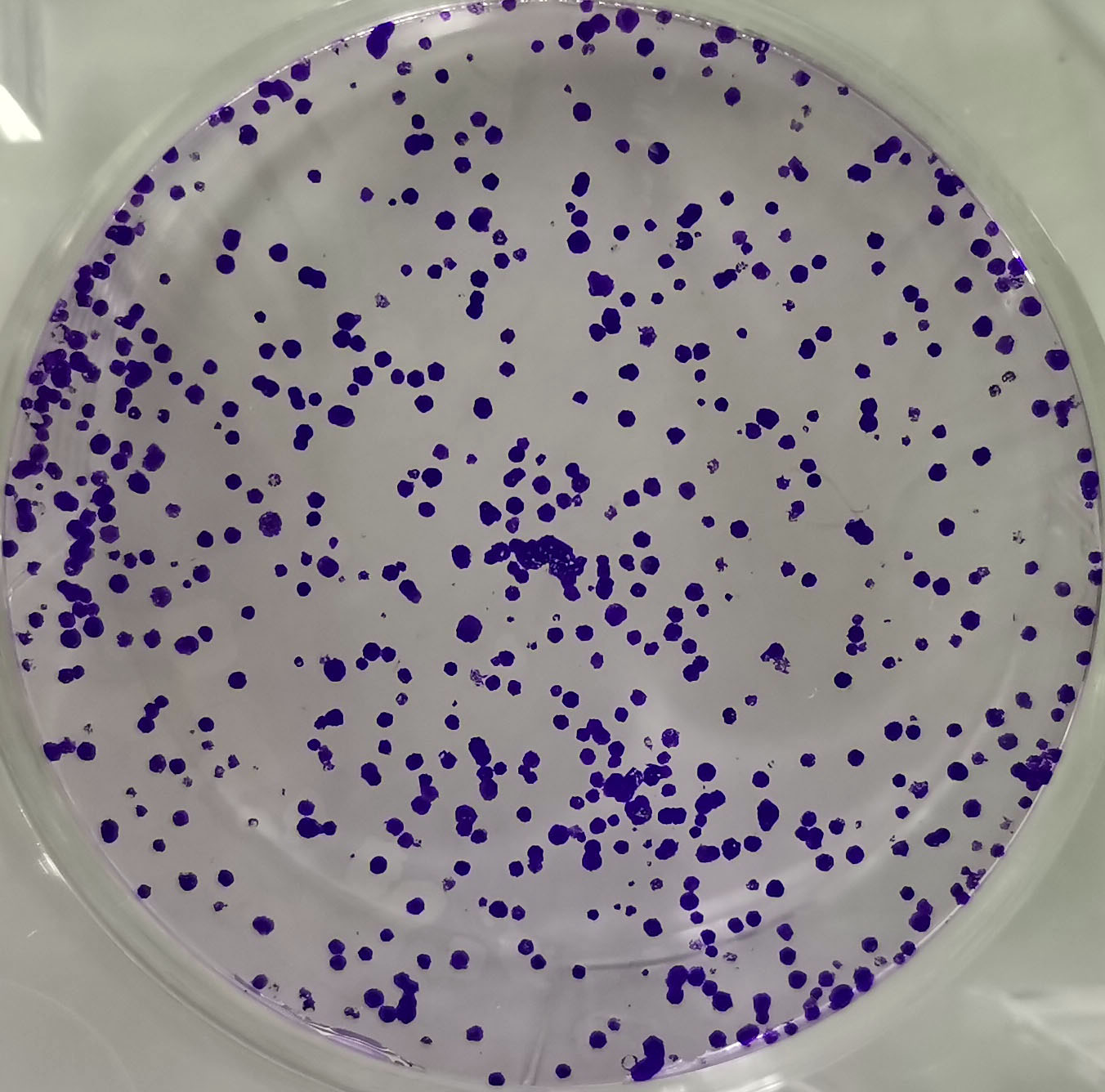

Supplement: Supplemental Information 8 [file peerj-09-11455-s008.zip › fig3D-Cell colony formation/MG63/Control-1.jpg]

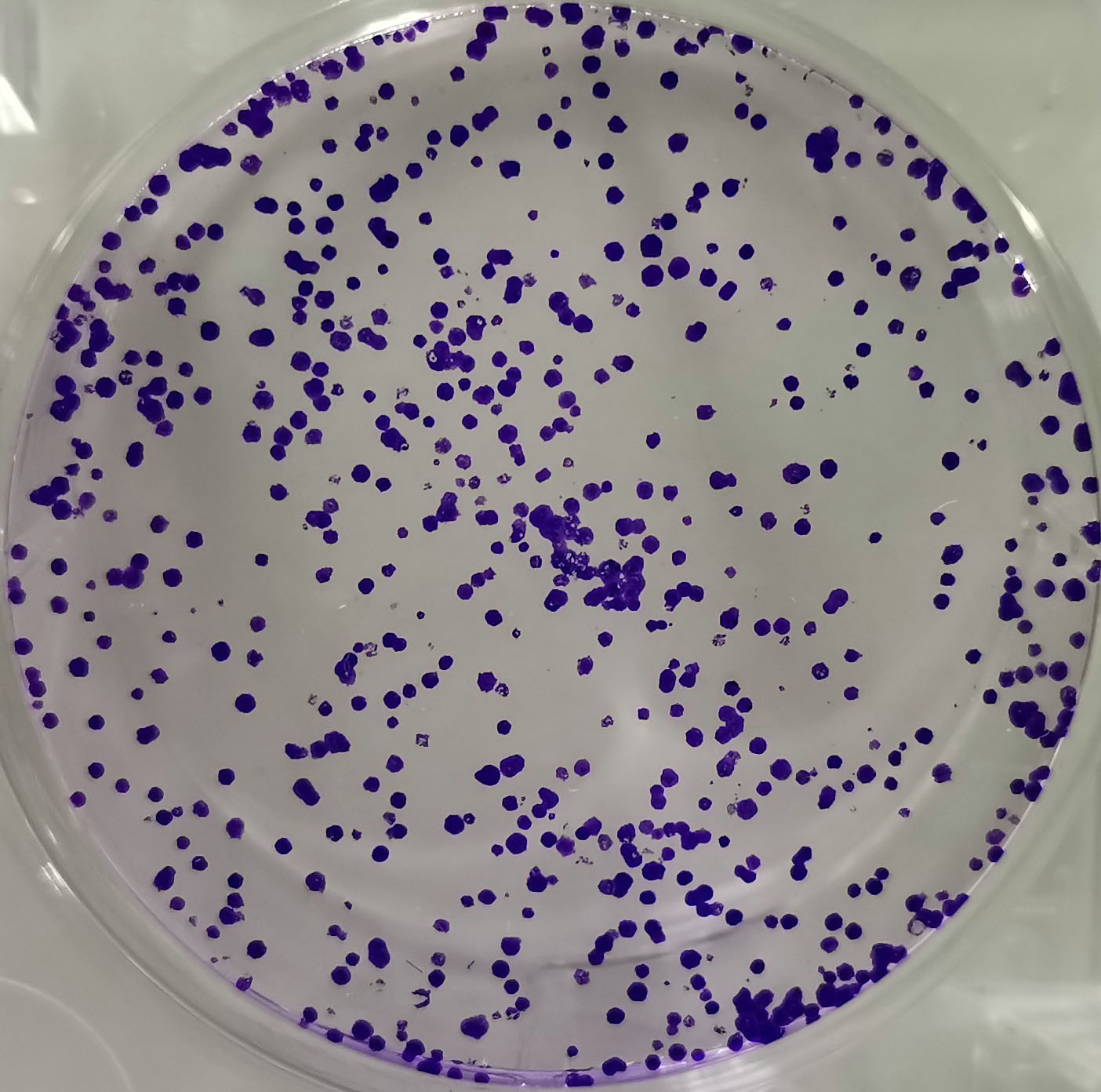

Supplement: Supplemental Information 8 [file peerj-09-11455-s008.zip › fig3D-Cell colony formation/MG63/Control-2.jpg]

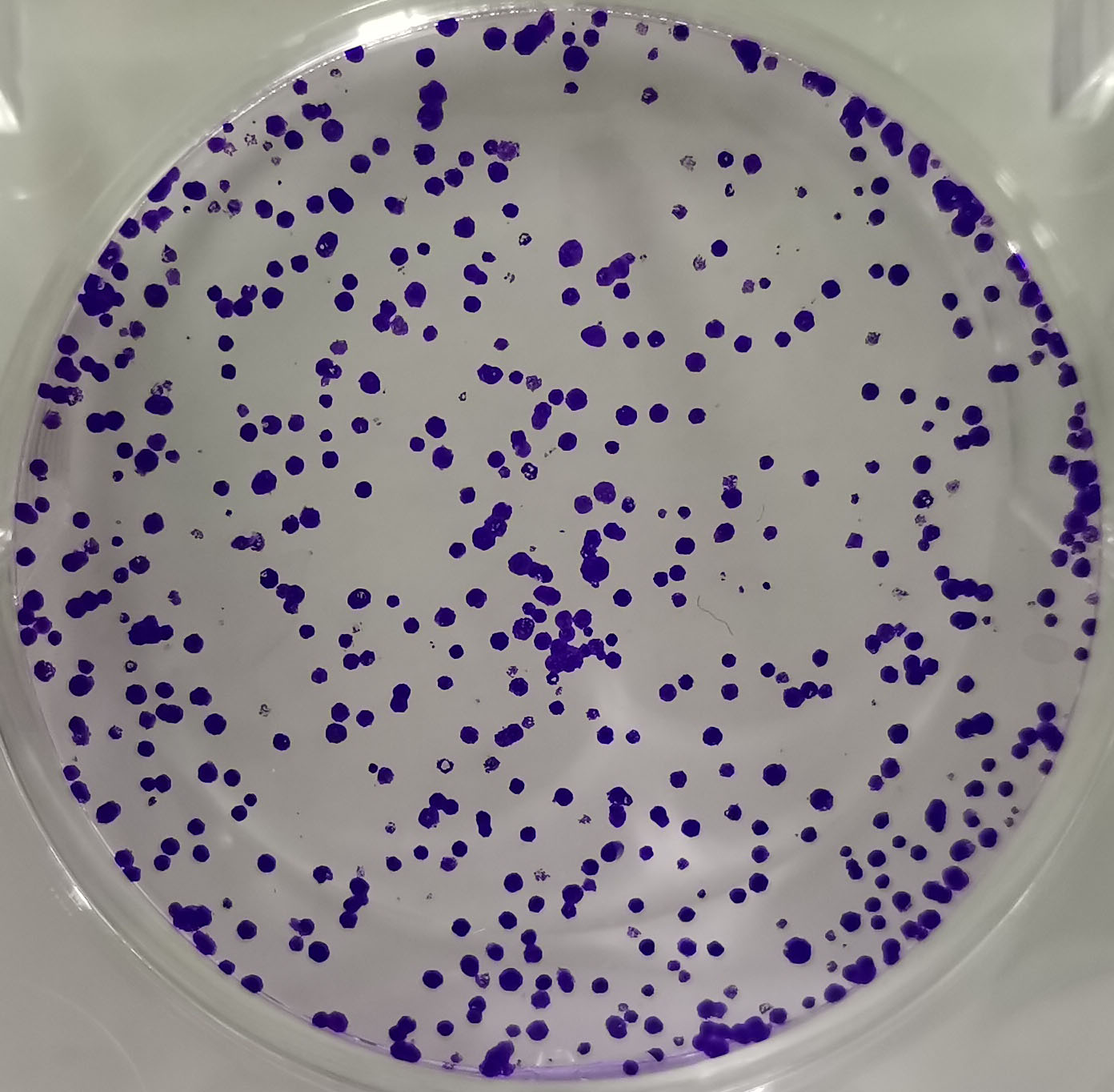

Supplement: Supplemental Information 8 [file peerj-09-11455-s008.zip › fig3D-Cell colony formation/MG63/Control-3.jpg]

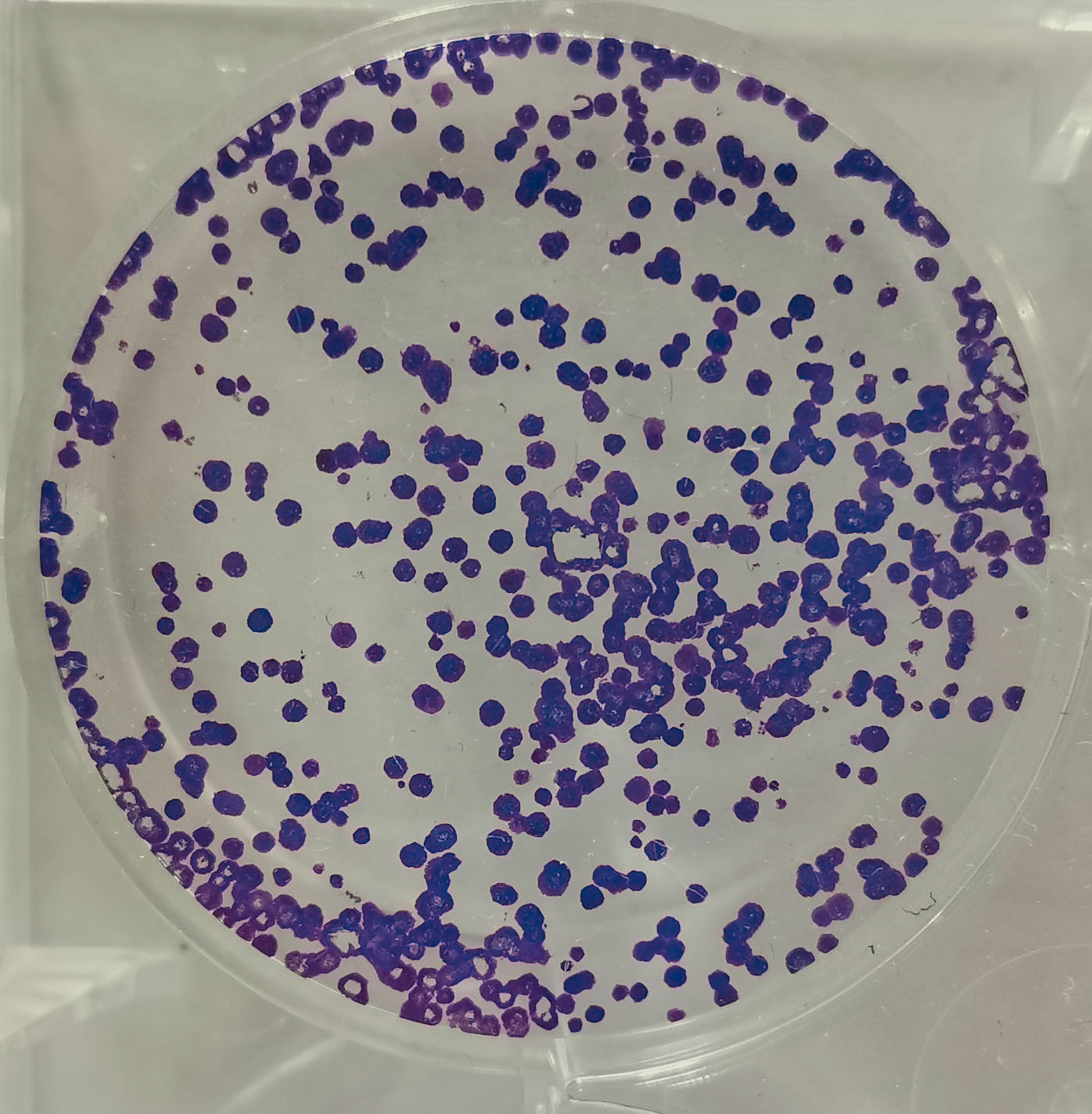

Supplement: Supplemental Information 8 [file peerj-09-11455-s008.zip › fig3D-Cell colony formation/MG63/oe-KIF4A-1.jpg]

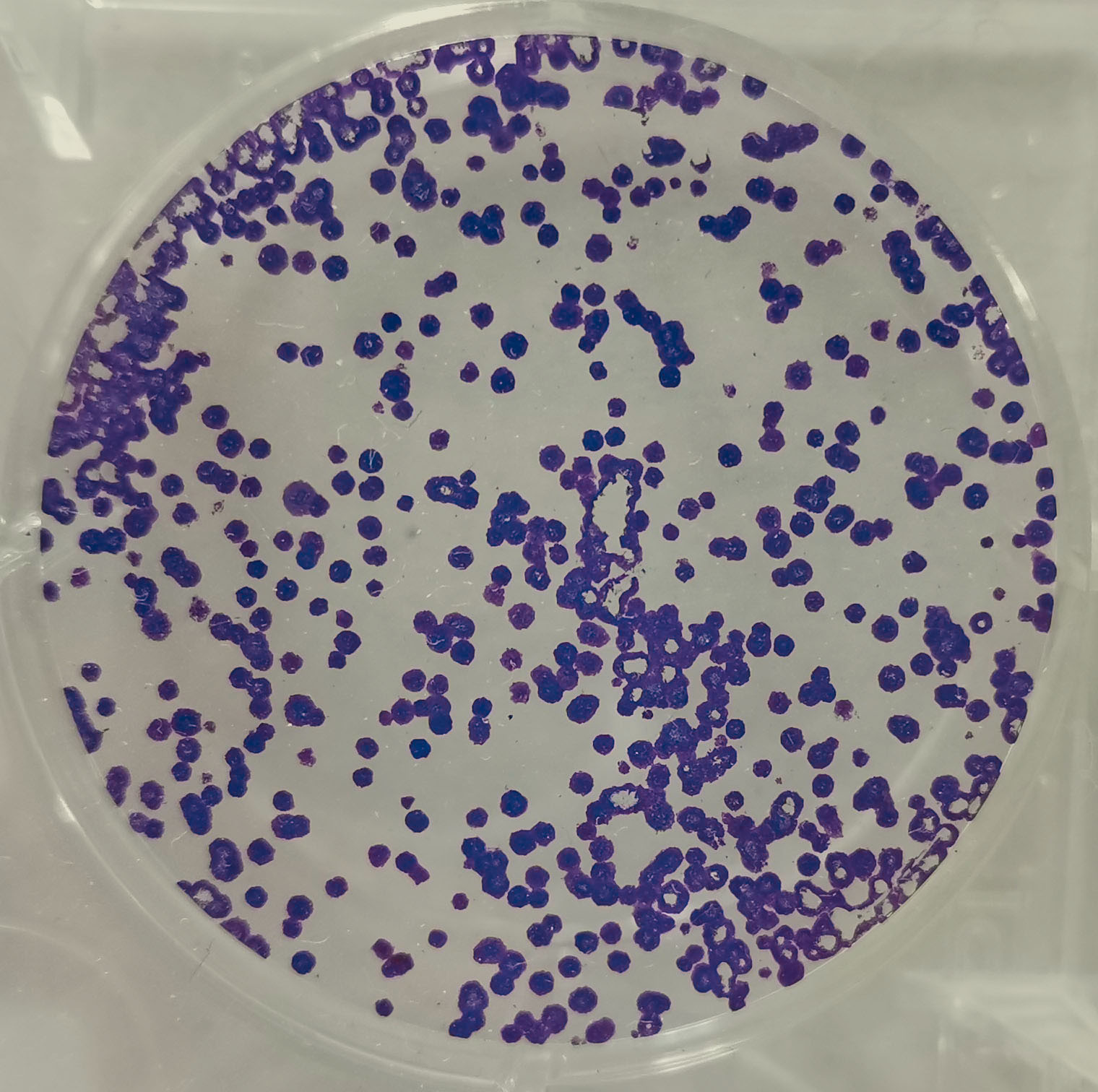

Supplement: Supplemental Information 8 [file peerj-09-11455-s008.zip › fig3D-Cell colony formation/MG63/oe-KIF4A-2.jpg]

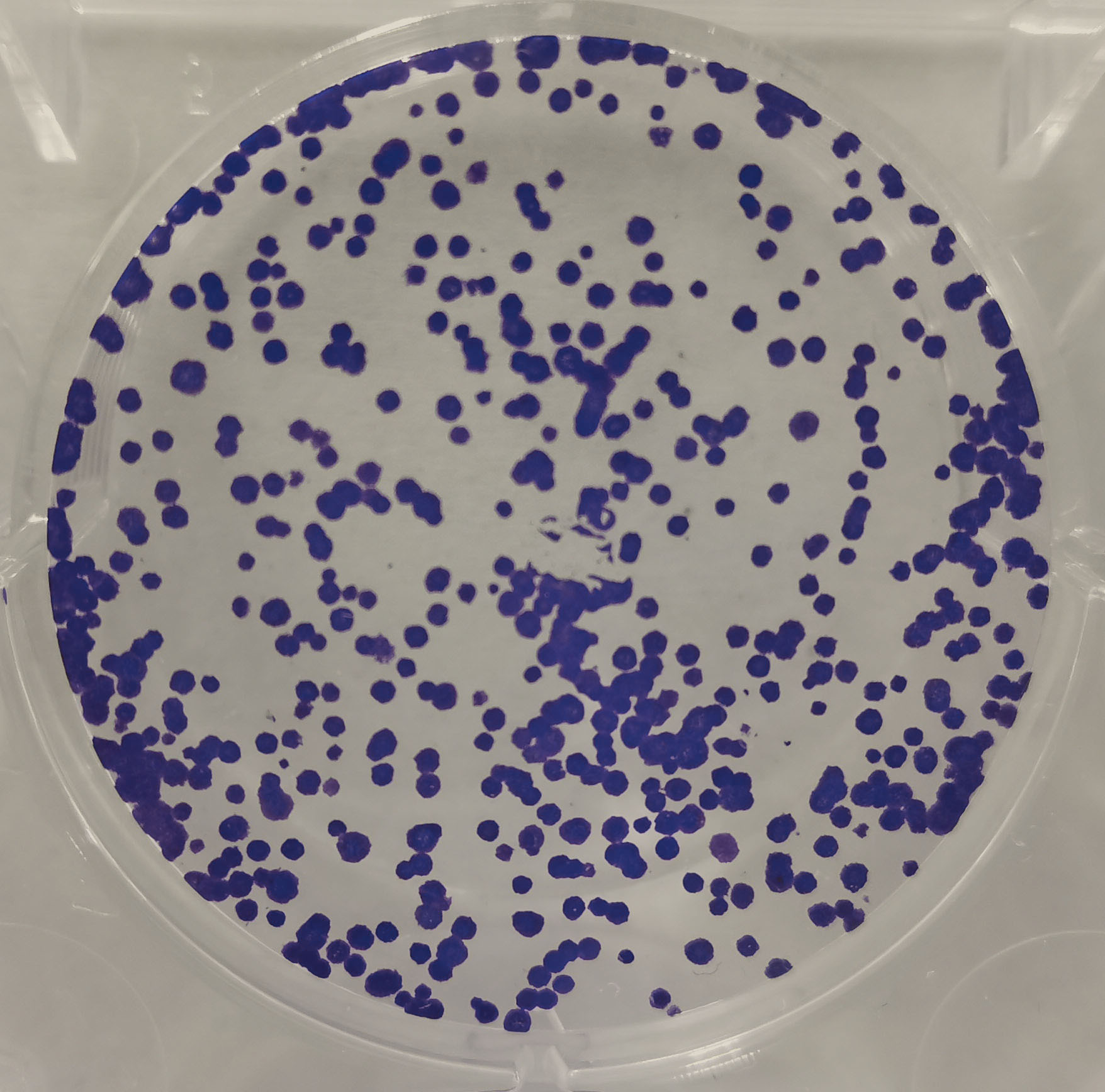

Supplement: Supplemental Information 8 [file peerj-09-11455-s008.zip › fig3D-Cell colony formation/MG63/oe-KIF4A-3.jpg]

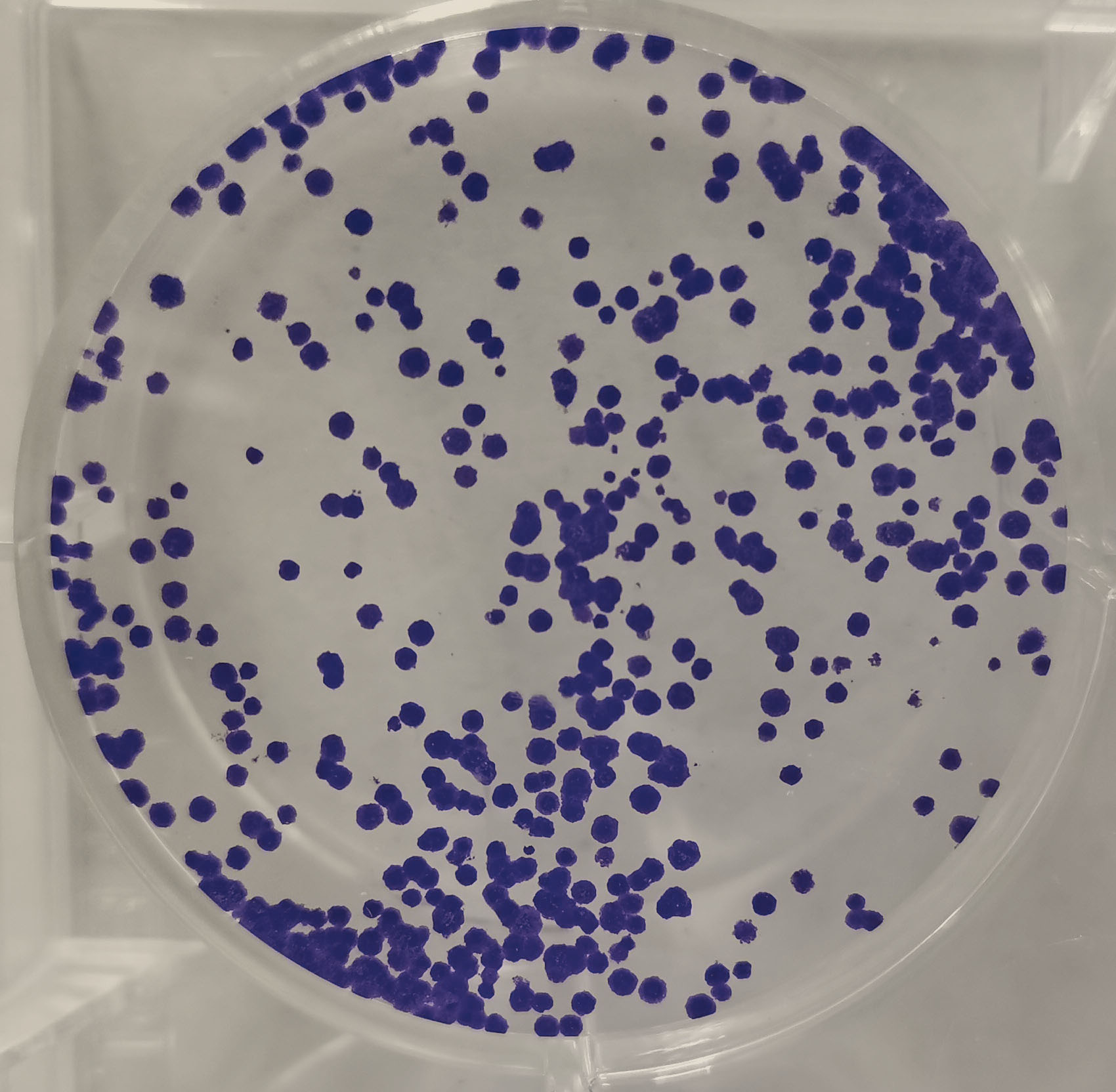

Supplement: Supplemental Information 8 [file peerj-09-11455-s008.zip › fig3D-Cell colony formation/MG63/oe-NC-1.jpg]

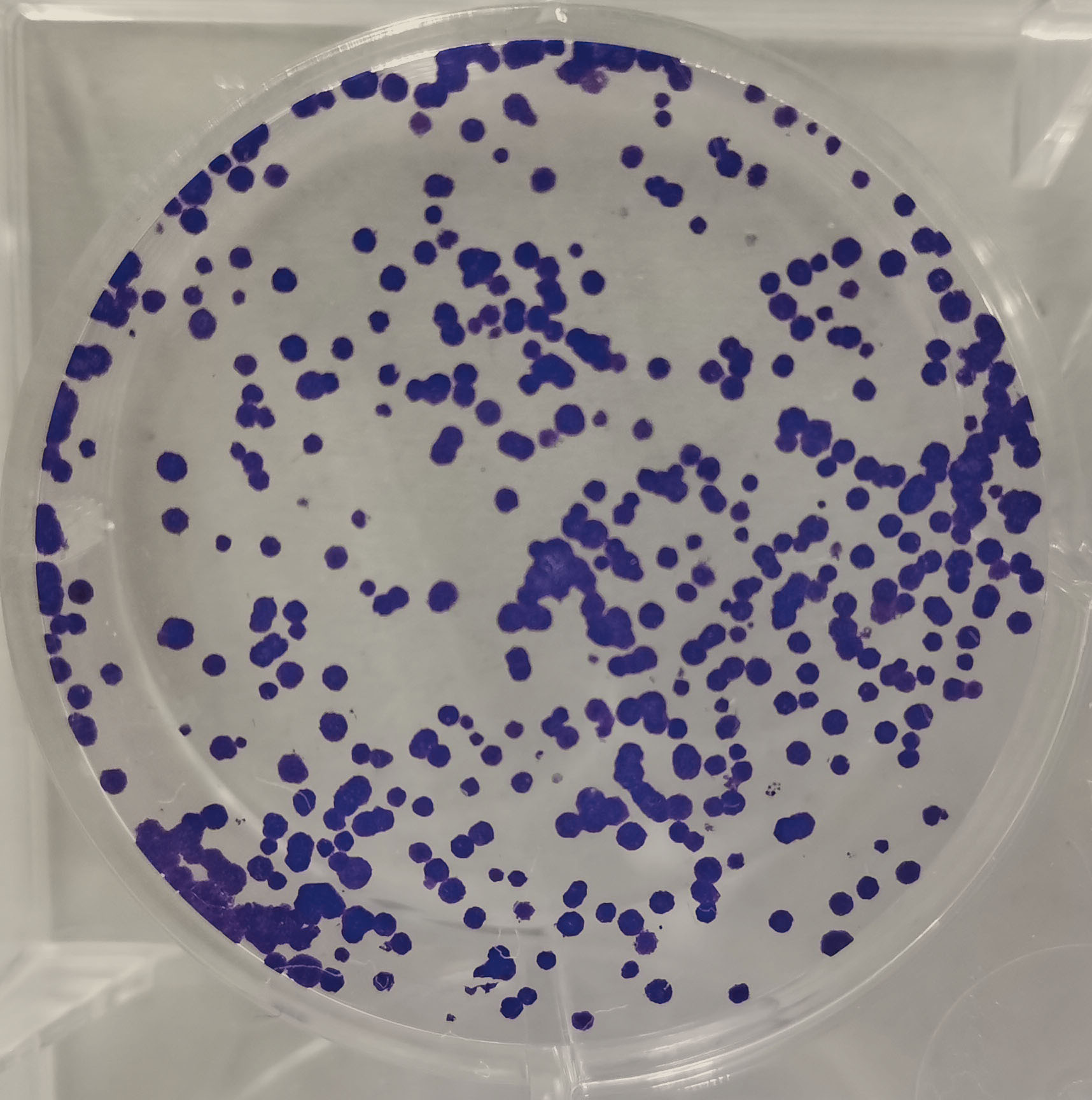

Supplement: Supplemental Information 8 [file peerj-09-11455-s008.zip › fig3D-Cell colony formation/MG63/oe-NC-2.jpg]

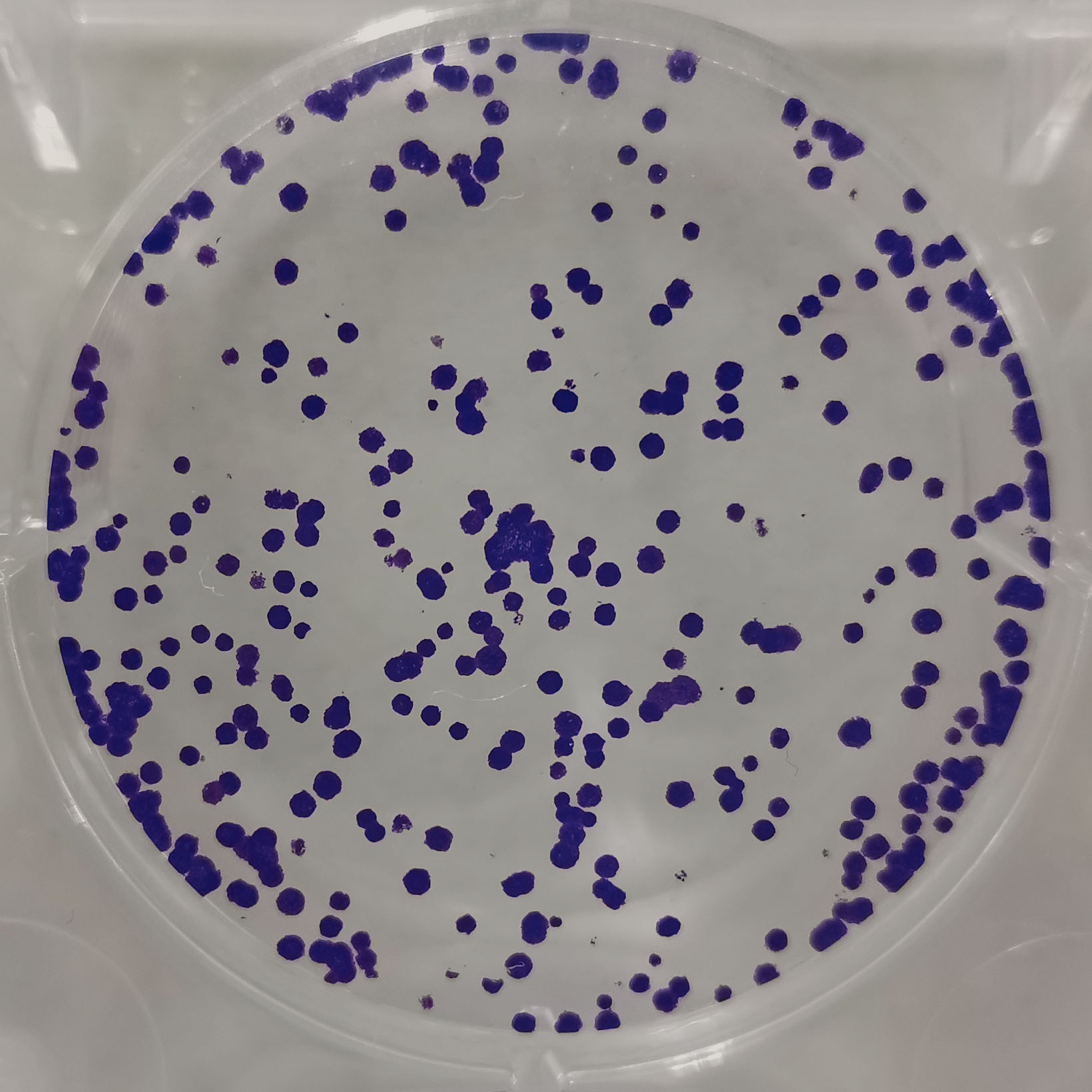

Supplement: Supplemental Information 8 [file peerj-09-11455-s008.zip › fig3D-Cell colony formation/MG63/oe-NC-3.jpg]

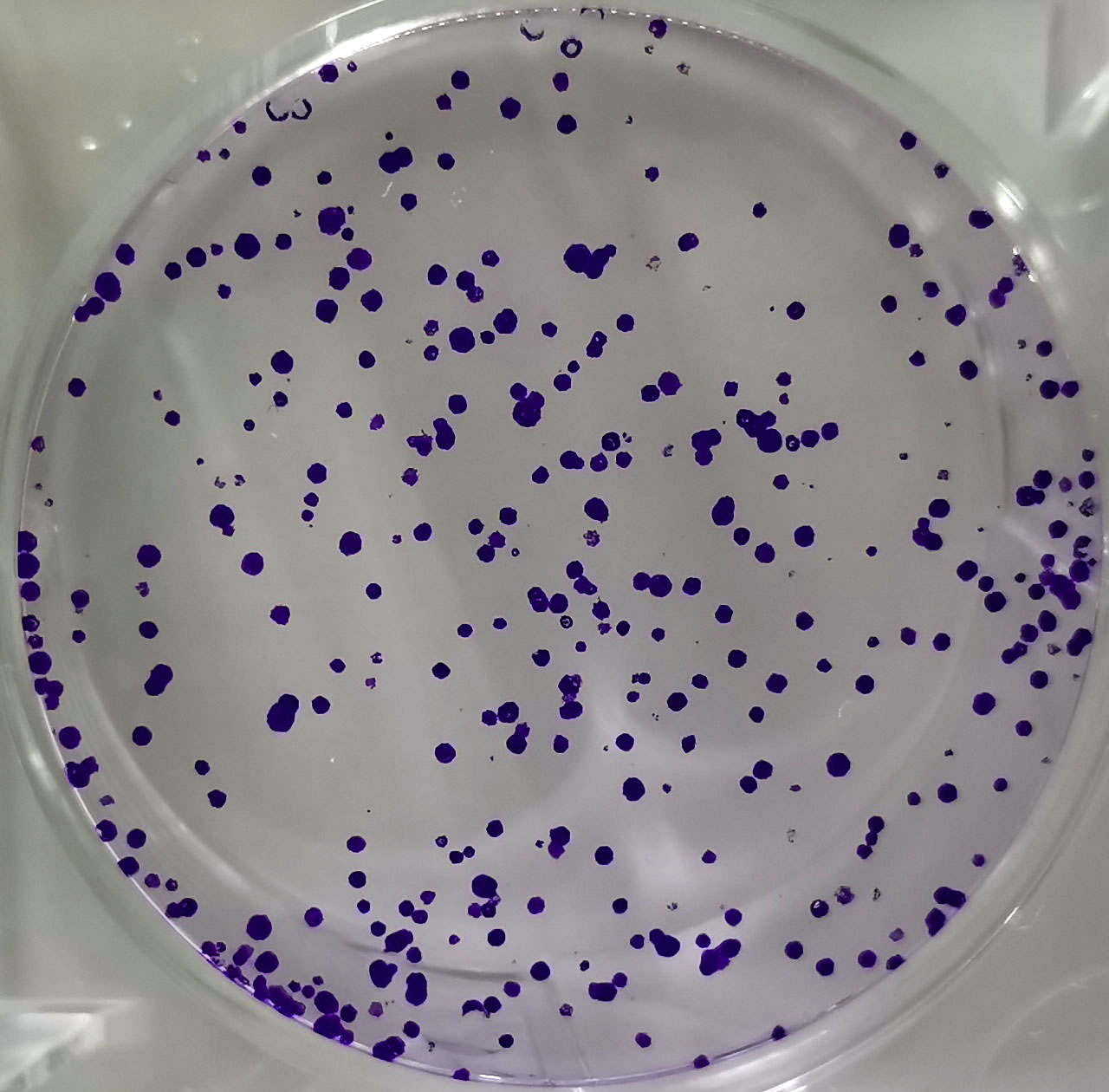

Supplement: Supplemental Information 8 [file peerj-09-11455-s008.zip › fig3D-Cell colony formation/MG63/si-KIF4A-1.jpg]

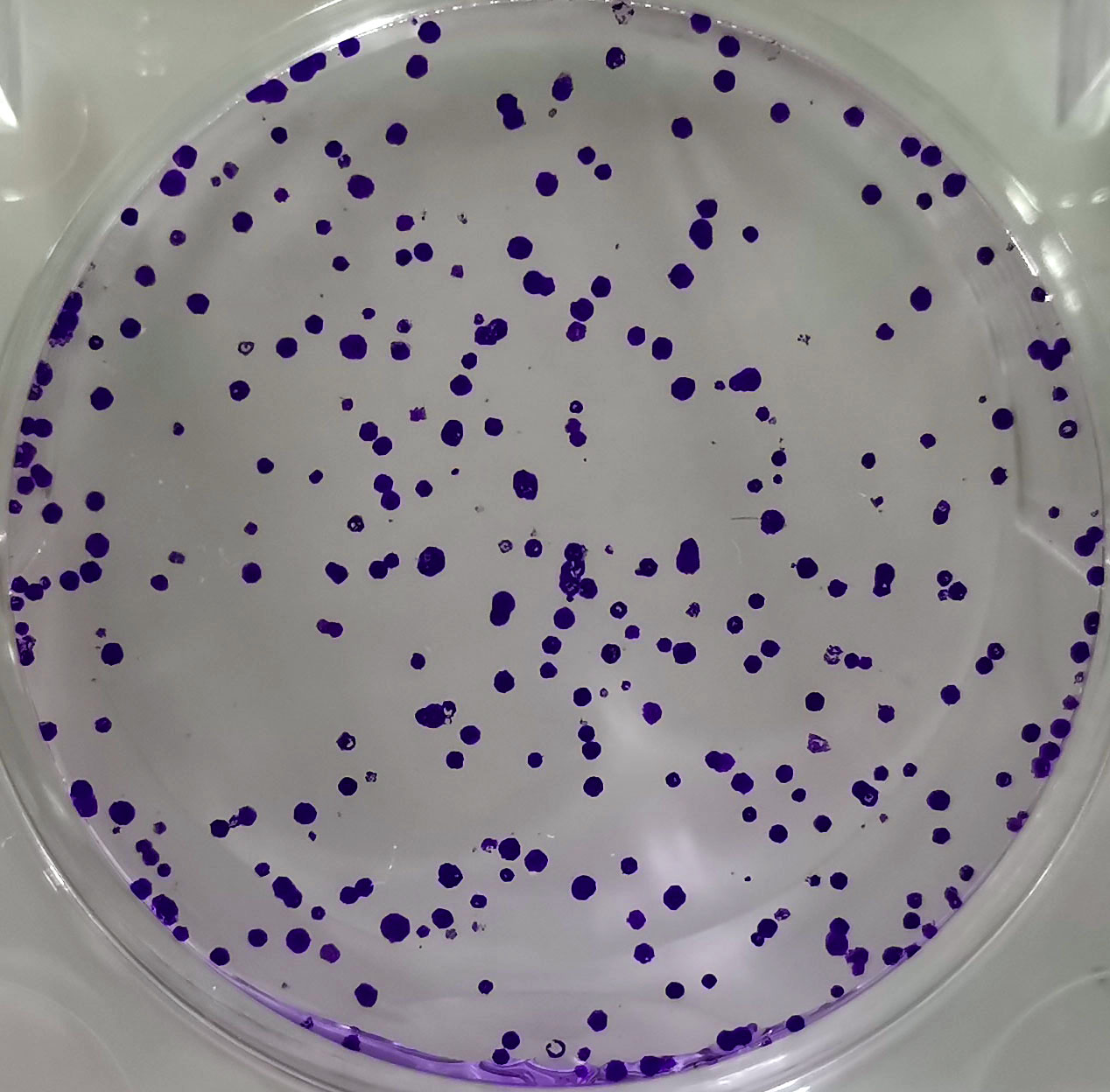

Supplement: Supplemental Information 8 [file peerj-09-11455-s008.zip › fig3D-Cell colony formation/MG63/si-KIF4A-2.jpg]

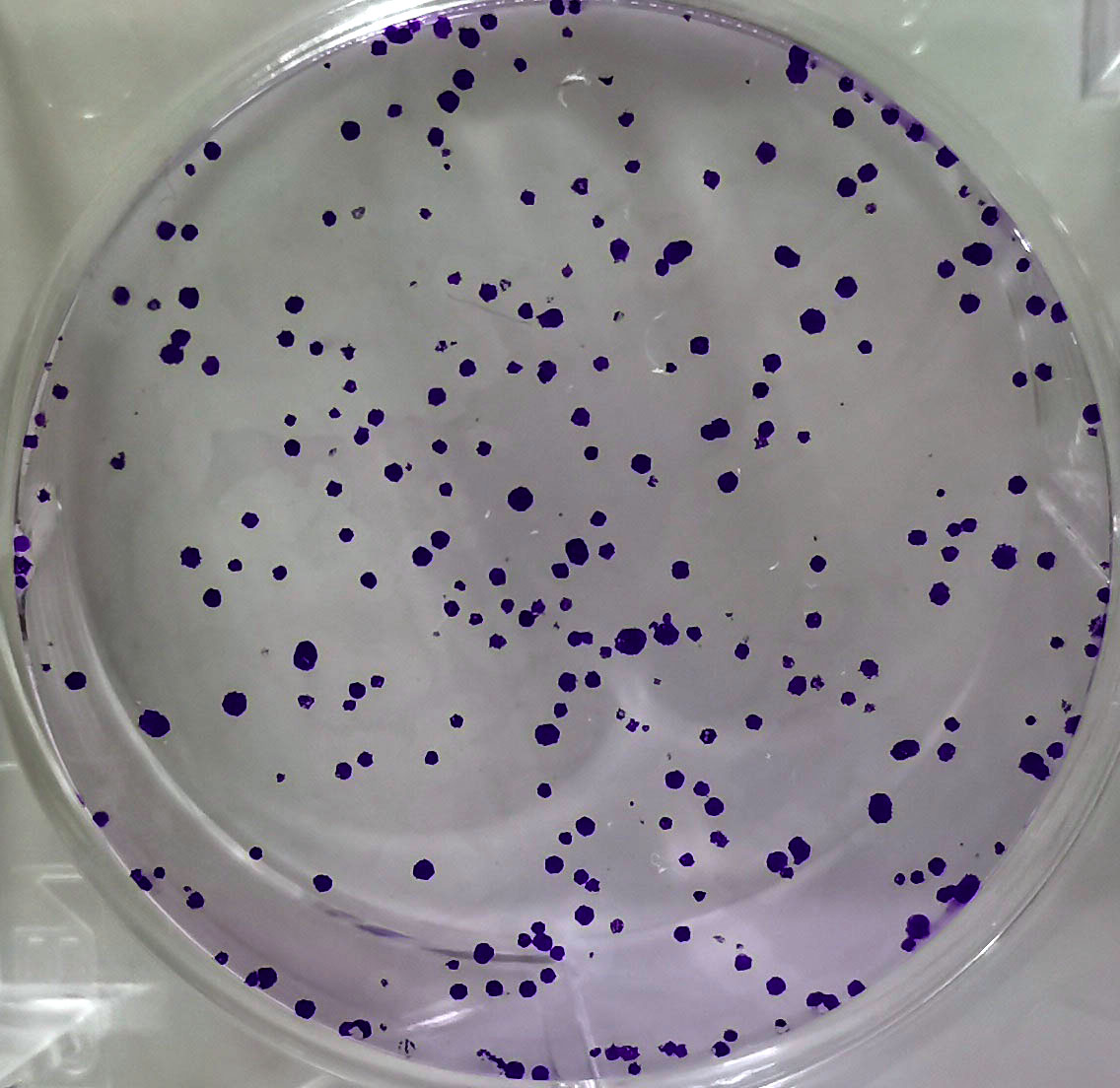

Supplement: Supplemental Information 8 [file peerj-09-11455-s008.zip › fig3D-Cell colony formation/MG63/si-KIF4A-3.jpg]

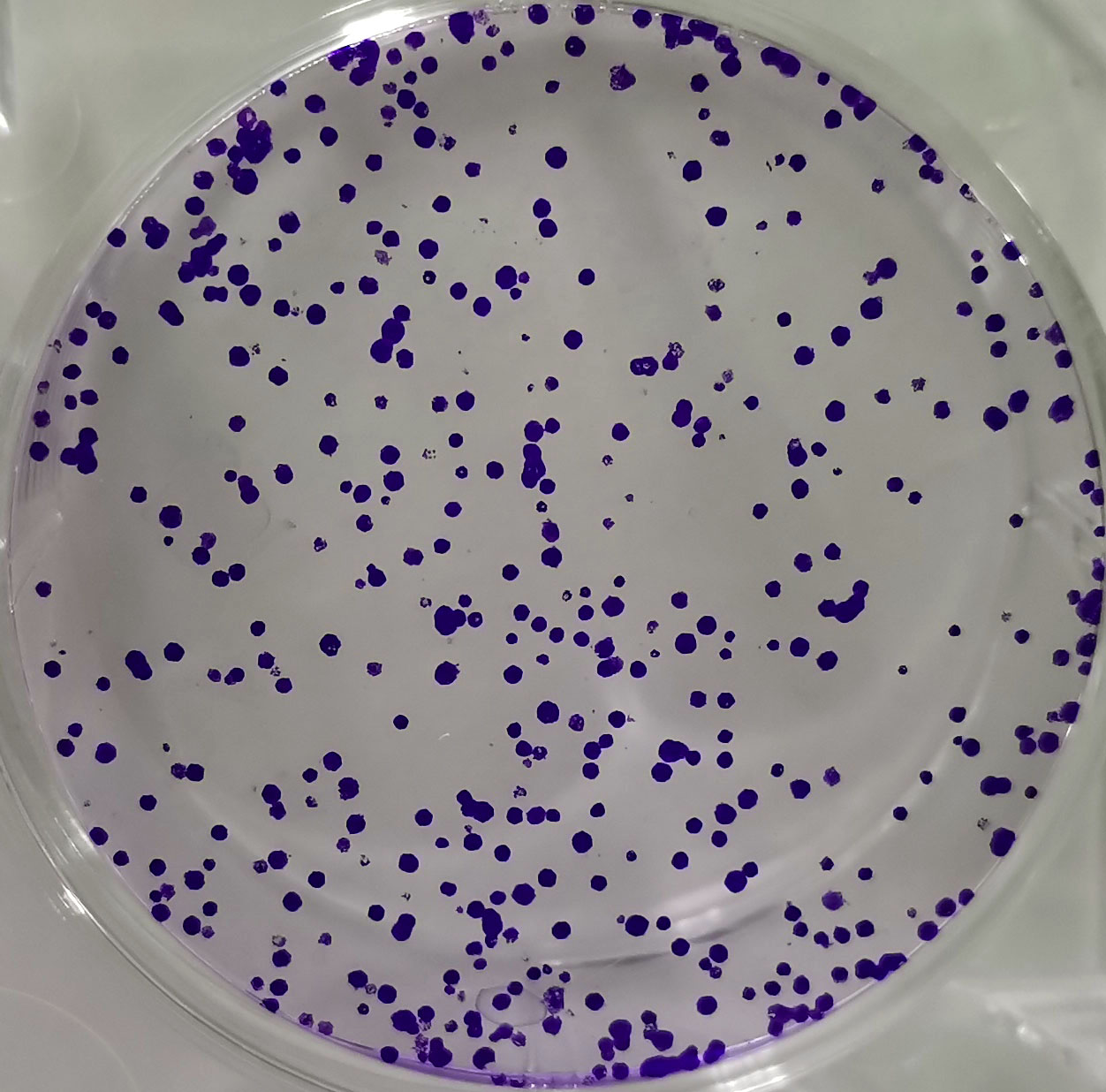

Supplement: Supplemental Information 8 [file peerj-09-11455-s008.zip › fig3D-Cell colony formation/MG63/si-NC-1.jpg]

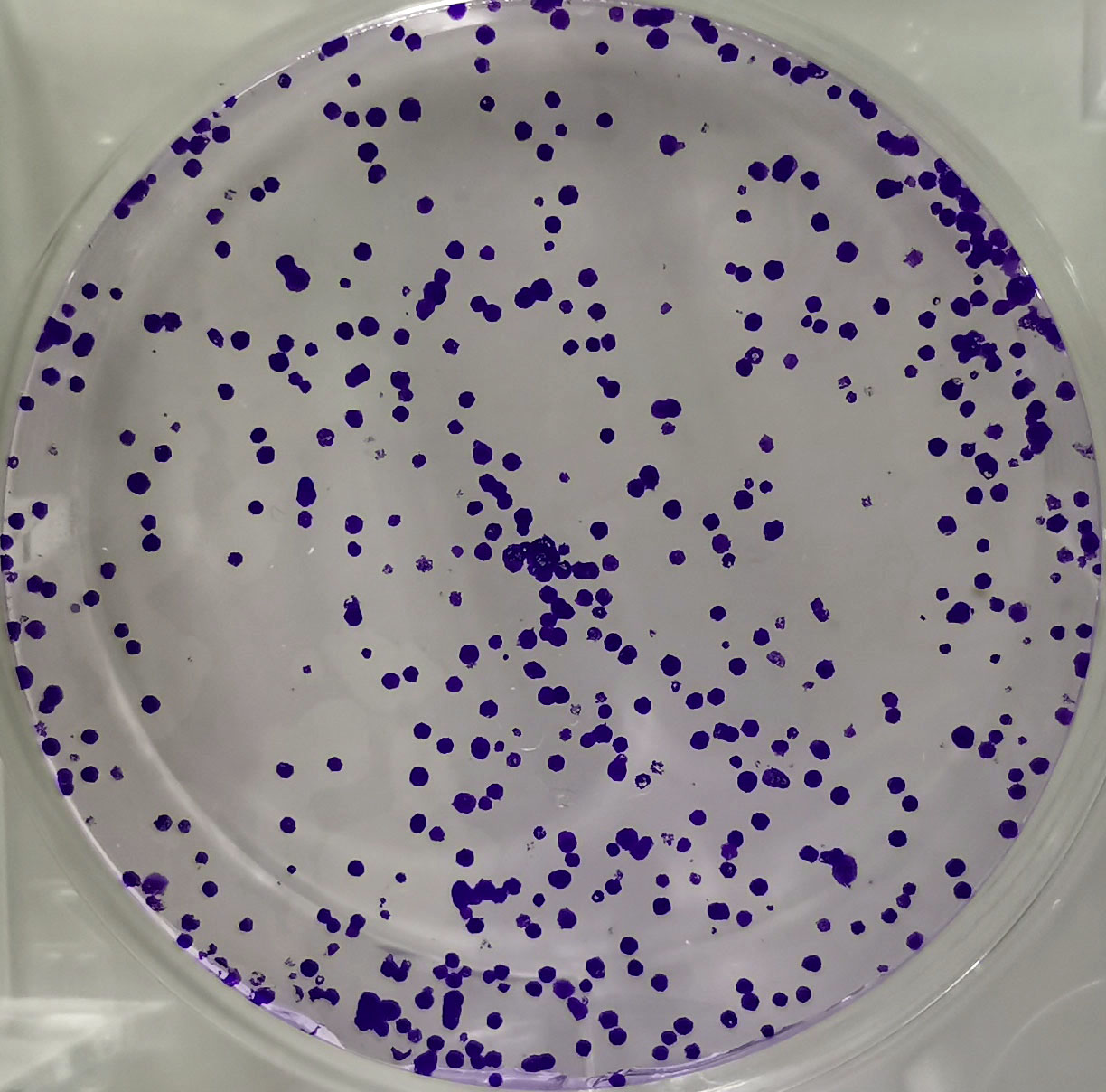

Supplement: Supplemental Information 8 [file peerj-09-11455-s008.zip › fig3D-Cell colony formation/MG63/si-NC-2.jpg]

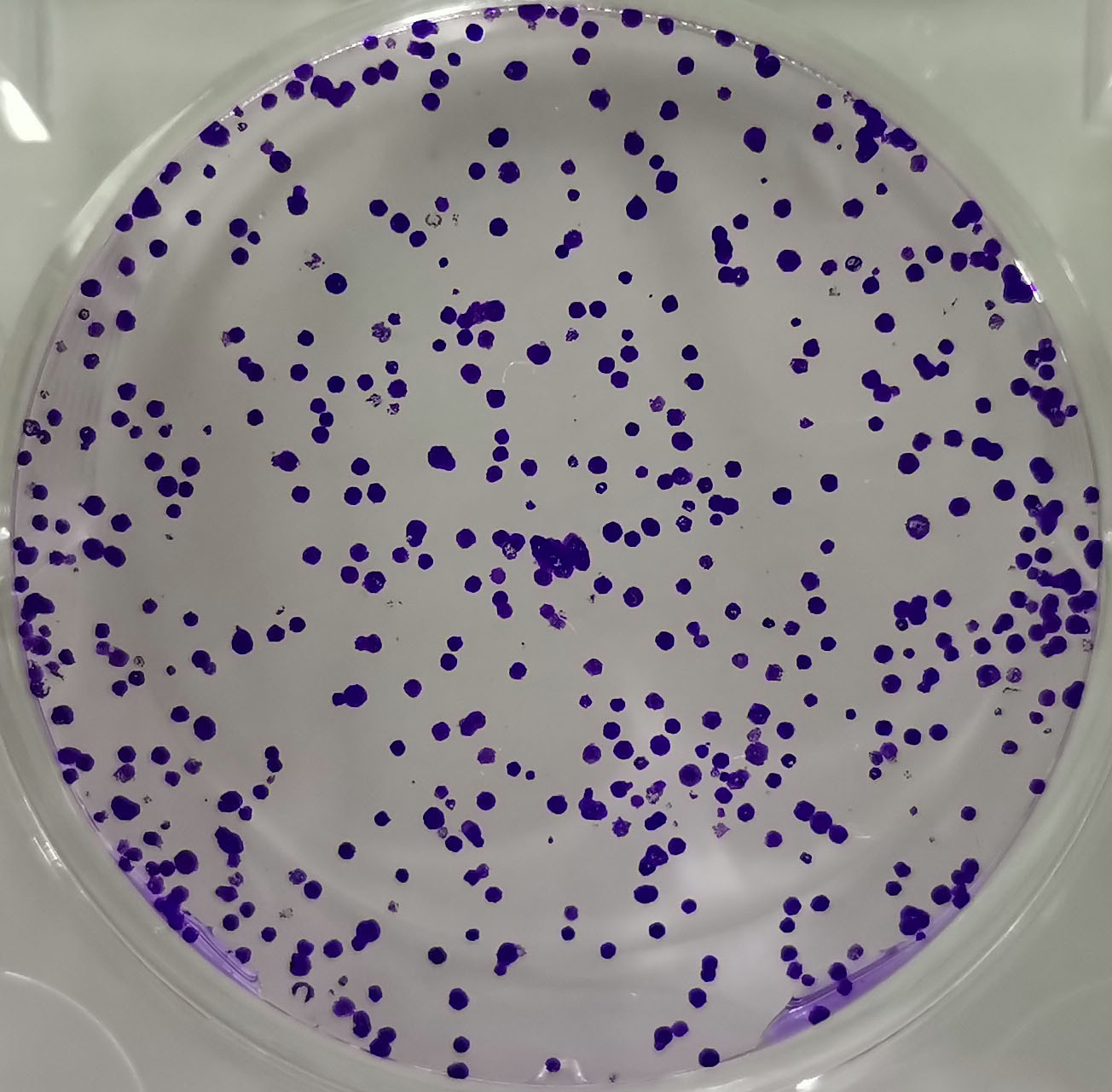

Supplement: Supplemental Information 8 [file peerj-09-11455-s008.zip › fig3D-Cell colony formation/MG63/si-NC-3.jpg]

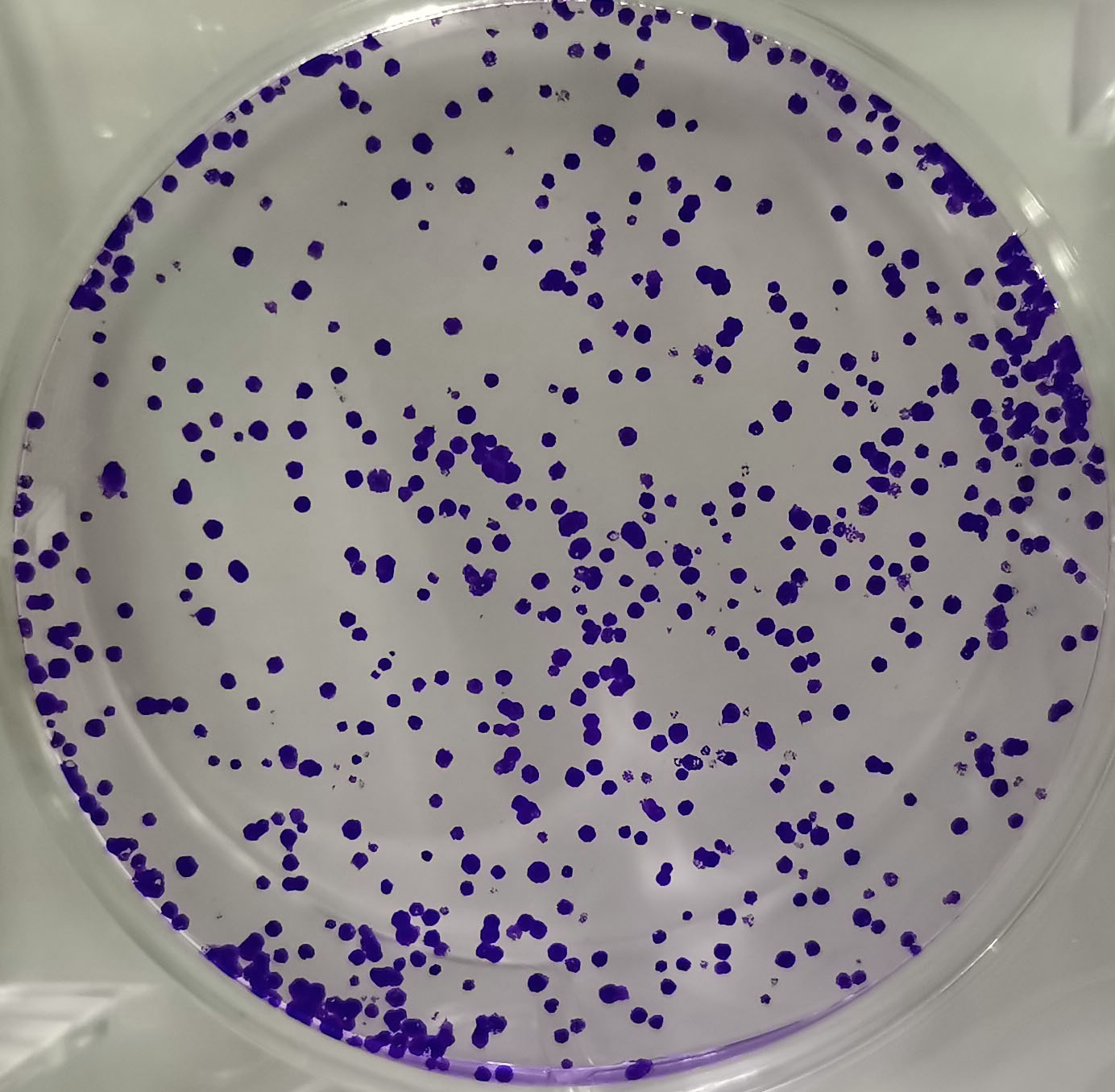

Supplement: Supplemental Information 8 [file peerj-09-11455-s008.zip › fig3D-Cell colony formation/U-20S/Control-1.jpg]

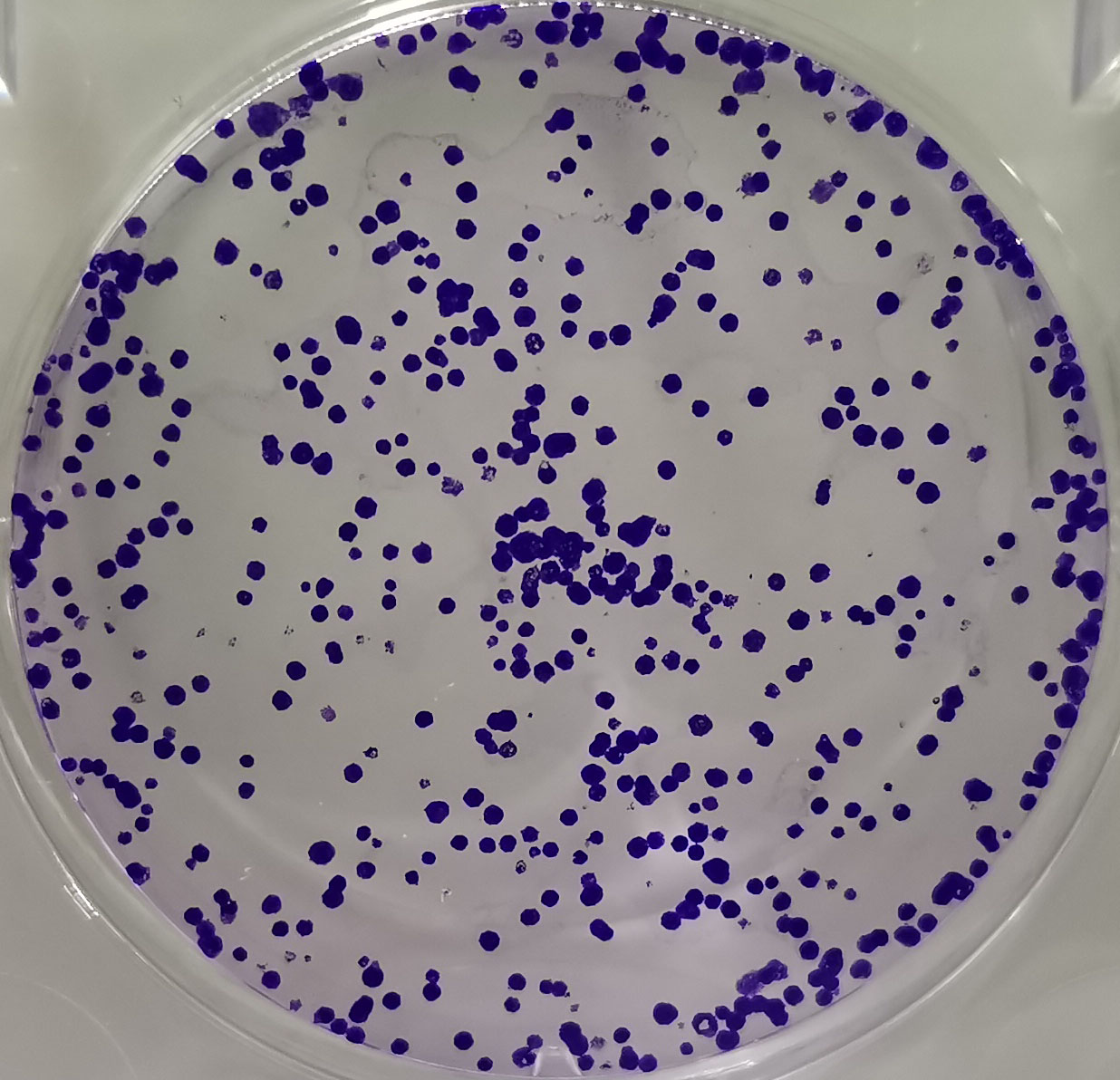

Supplement: Supplemental Information 8 [file peerj-09-11455-s008.zip › fig3D-Cell colony formation/U-20S/Control-2.jpg]

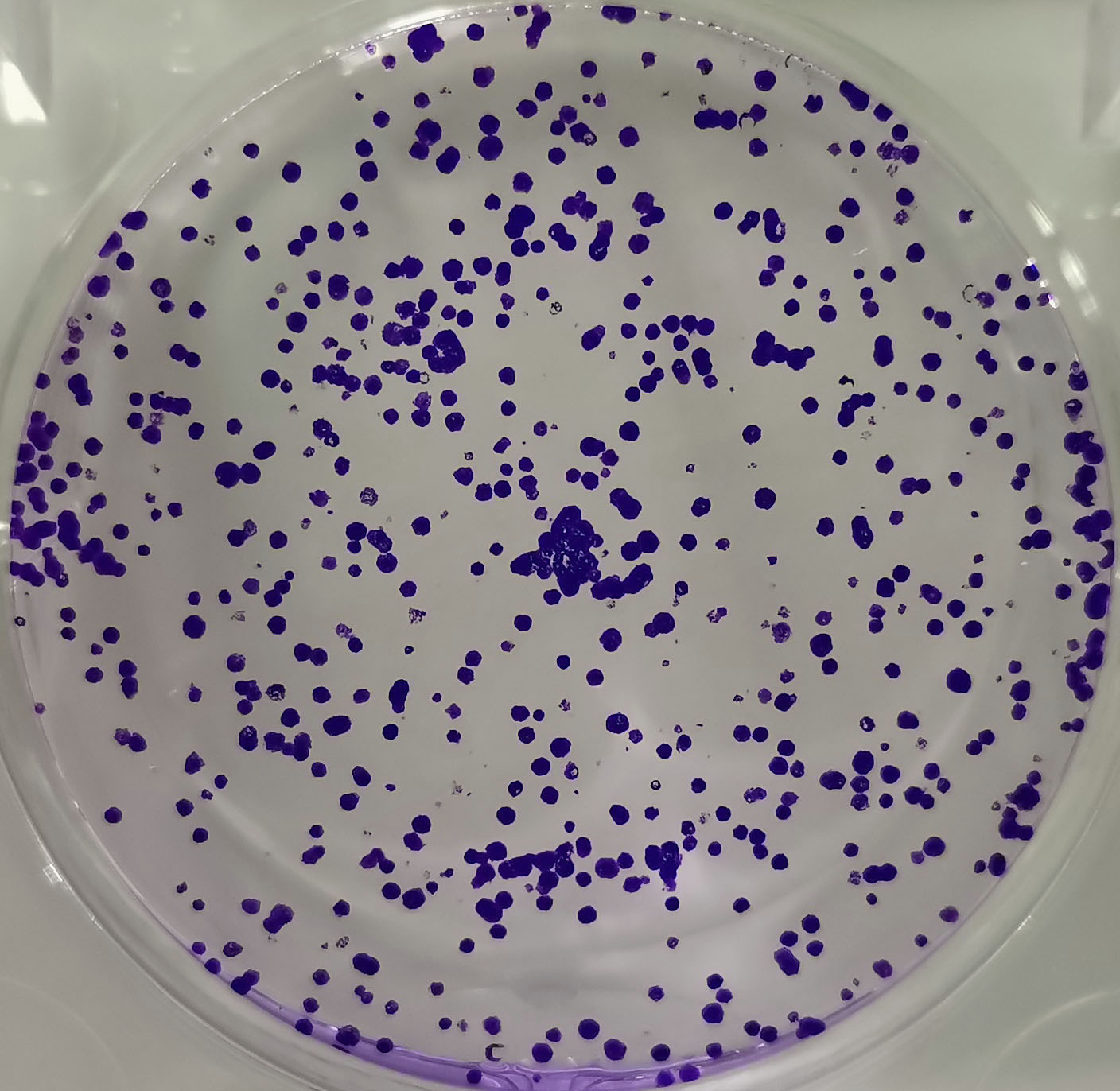

Supplement: Supplemental Information 8 [file peerj-09-11455-s008.zip › fig3D-Cell colony formation/U-20S/Control-3.jpg]

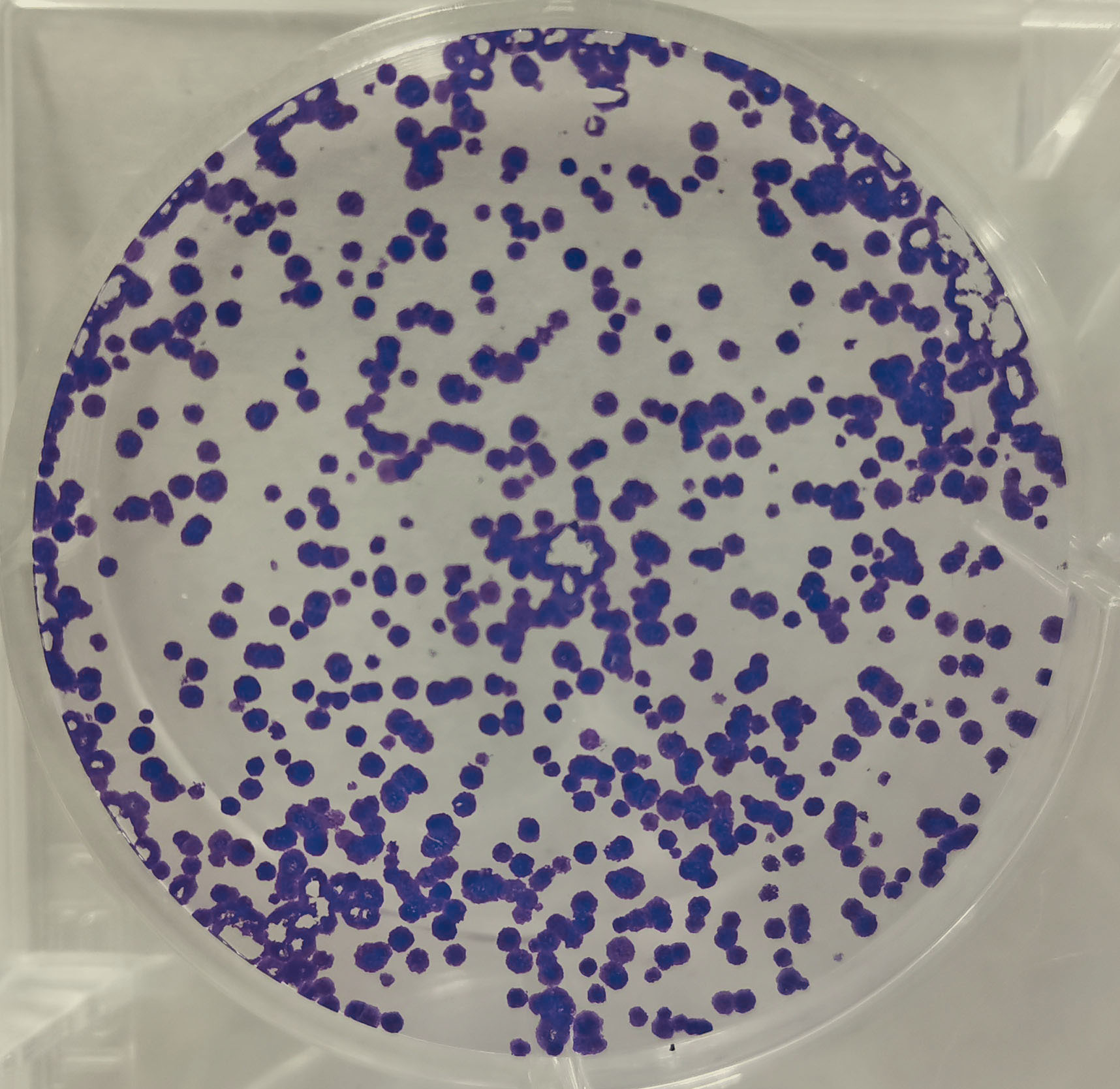

Supplement: Supplemental Information 8 [file peerj-09-11455-s008.zip › fig3D-Cell colony formation/U-20S/oe-KIF4A-1.jpg]

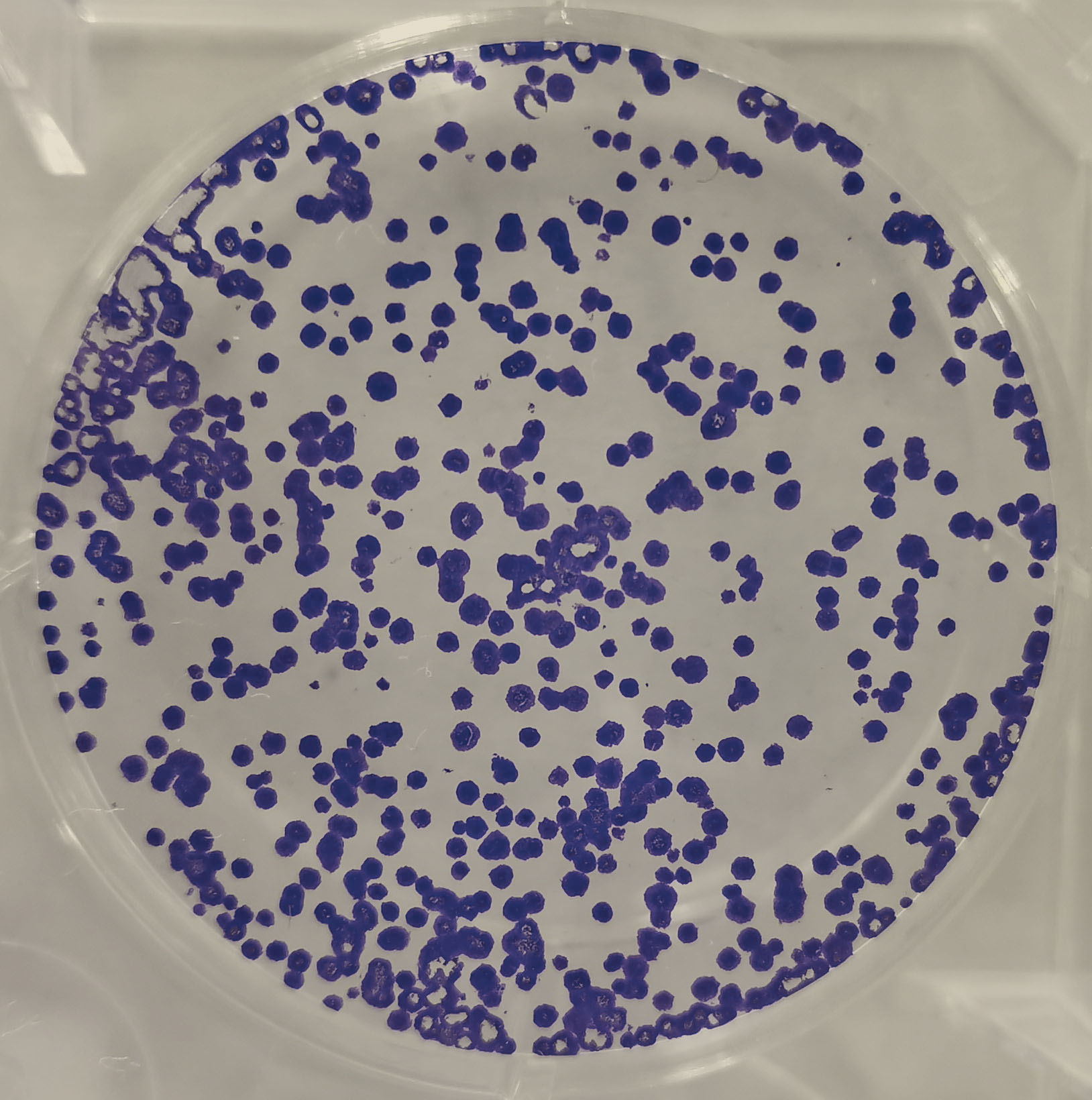

Supplement: Supplemental Information 8 [file peerj-09-11455-s008.zip › fig3D-Cell colony formation/U-20S/oe-KIF4A-2.jpg]

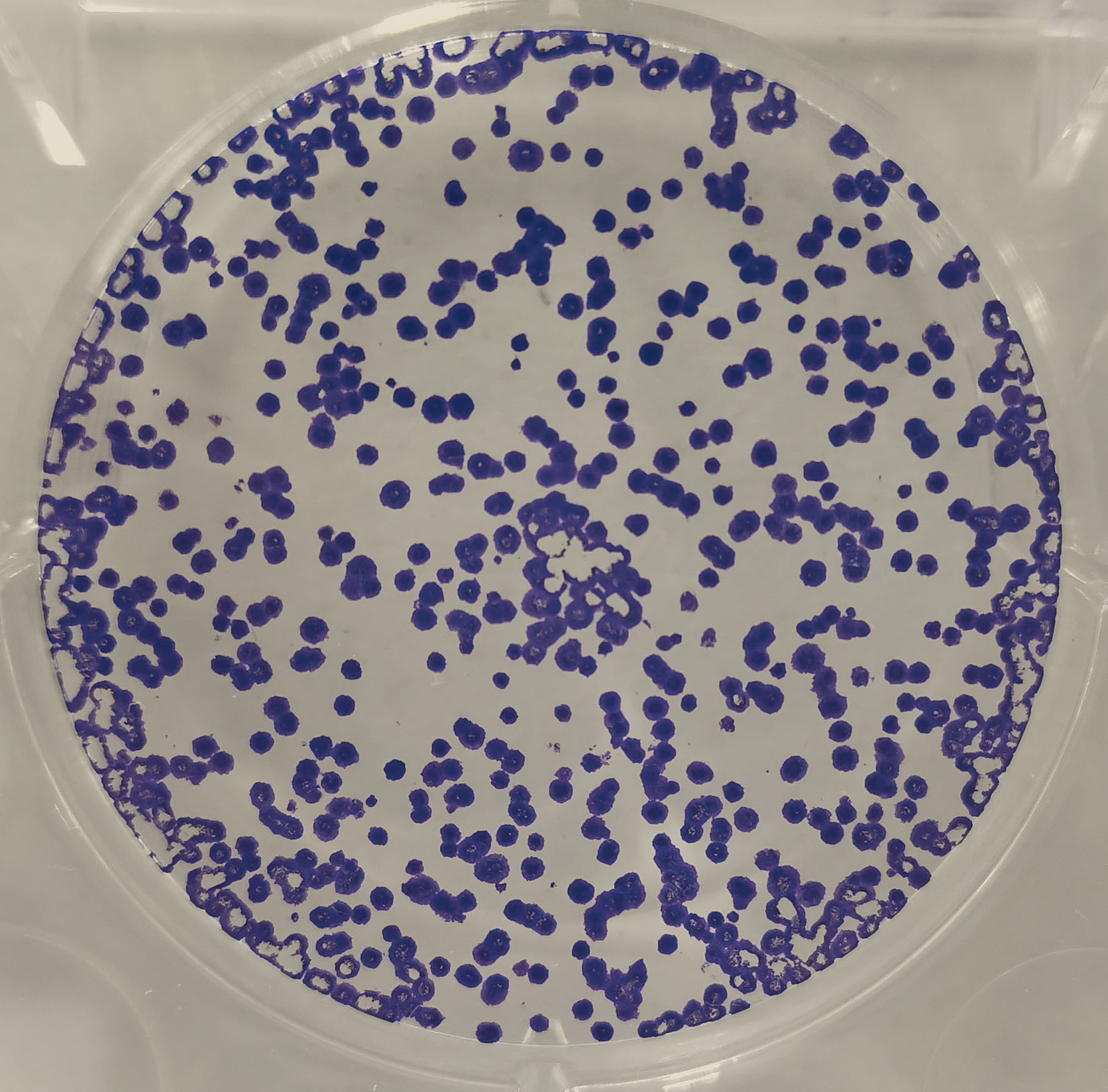

Supplement: Supplemental Information 8 [file peerj-09-11455-s008.zip › fig3D-Cell colony formation/U-20S/oe-KIF4A-3.jpg]

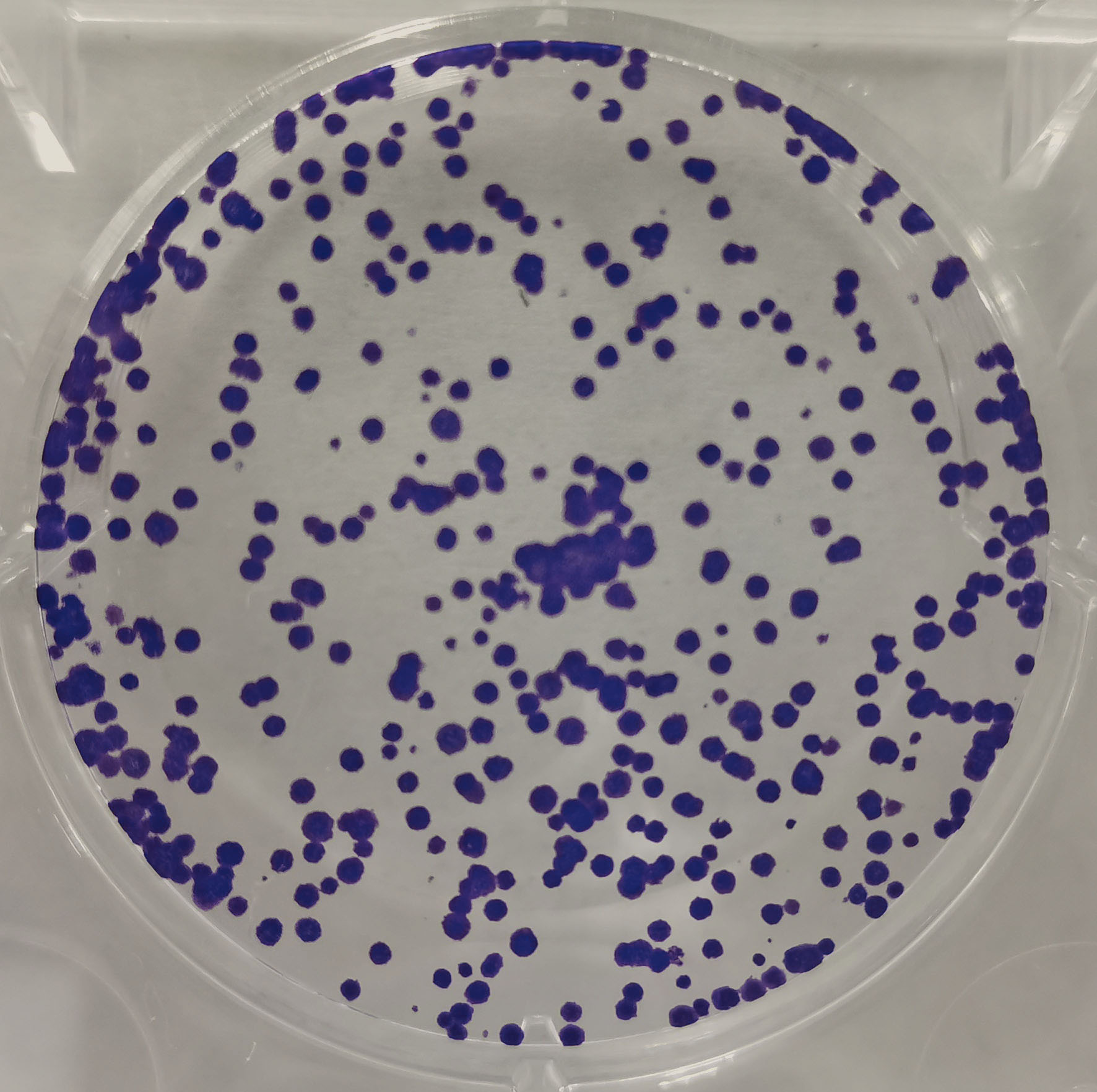

Supplement: Supplemental Information 8 [file peerj-09-11455-s008.zip › fig3D-Cell colony formation/U-20S/oe-NC-1.jpg]

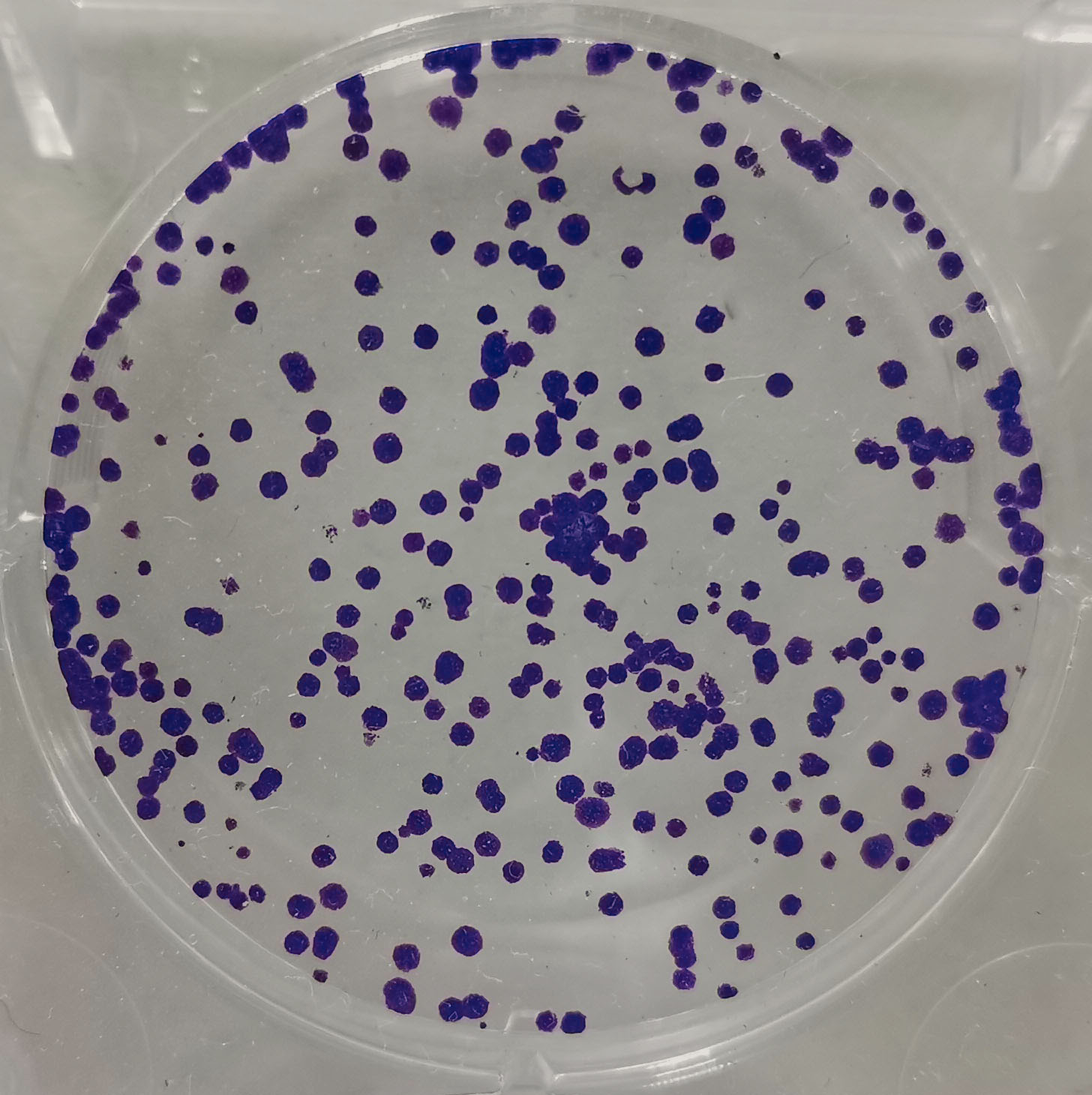

Supplement: Supplemental Information 8 [file peerj-09-11455-s008.zip › fig3D-Cell colony formation/U-20S/oe-NC-2.jpg]

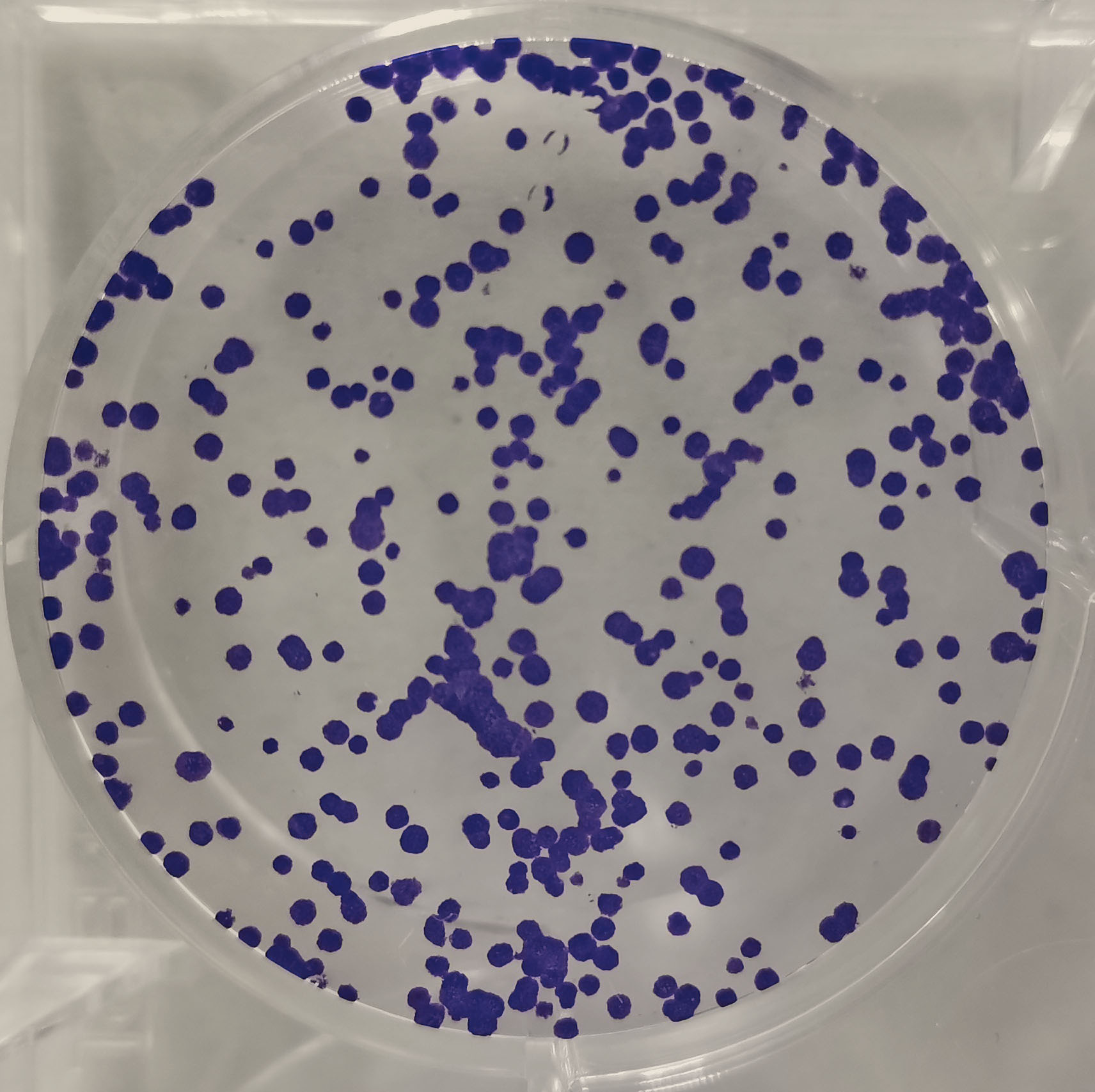

Supplement: Supplemental Information 8 [file peerj-09-11455-s008.zip › fig3D-Cell colony formation/U-20S/oe-NC-3.jpg]

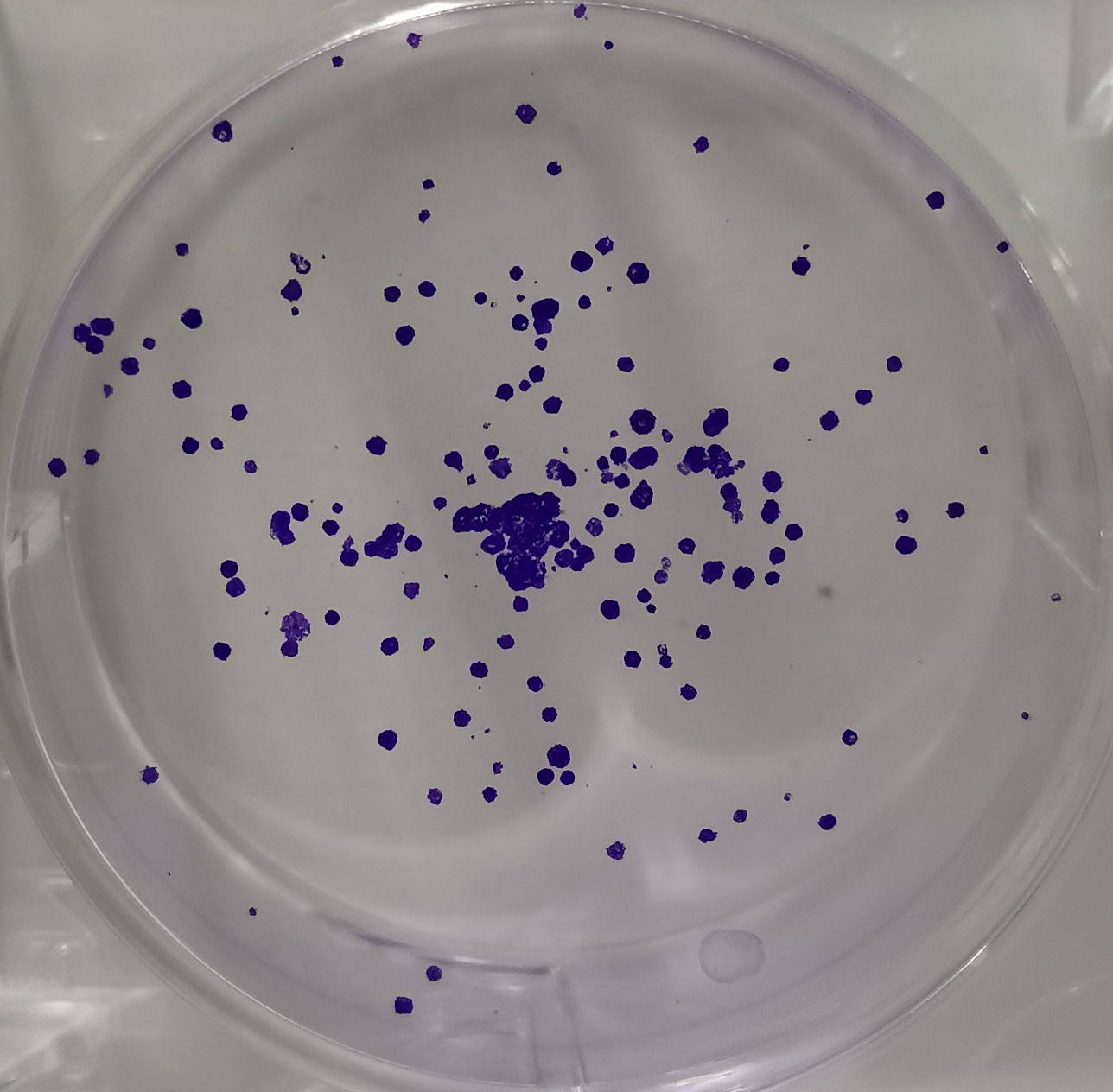

Supplement: Supplemental Information 8 [file peerj-09-11455-s008.zip › fig3D-Cell colony formation/U-20S/si-KIF4A-1.jpg]

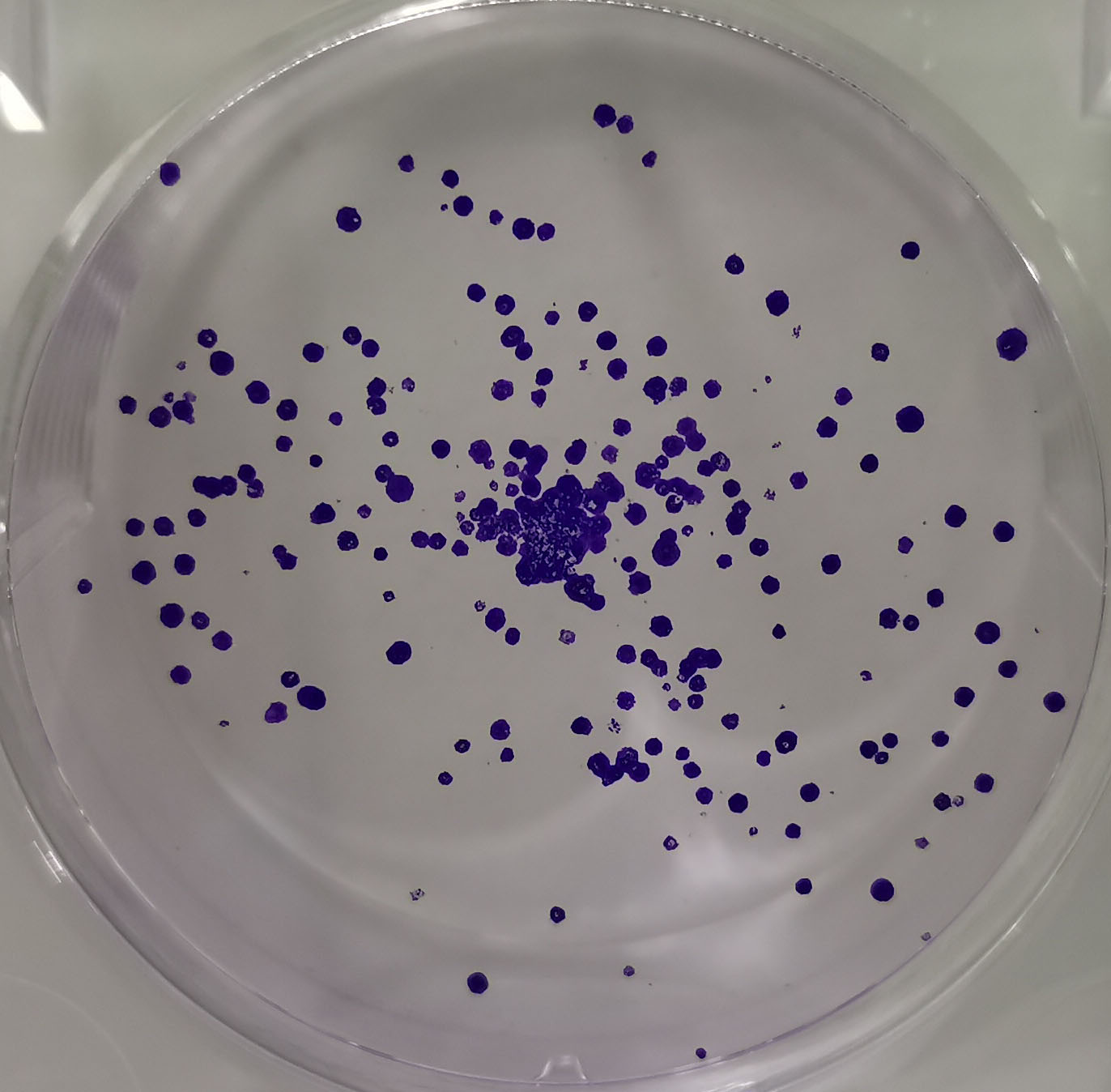

Supplement: Supplemental Information 8 [file peerj-09-11455-s008.zip › fig3D-Cell colony formation/U-20S/si-KIF4A-2.jpg]

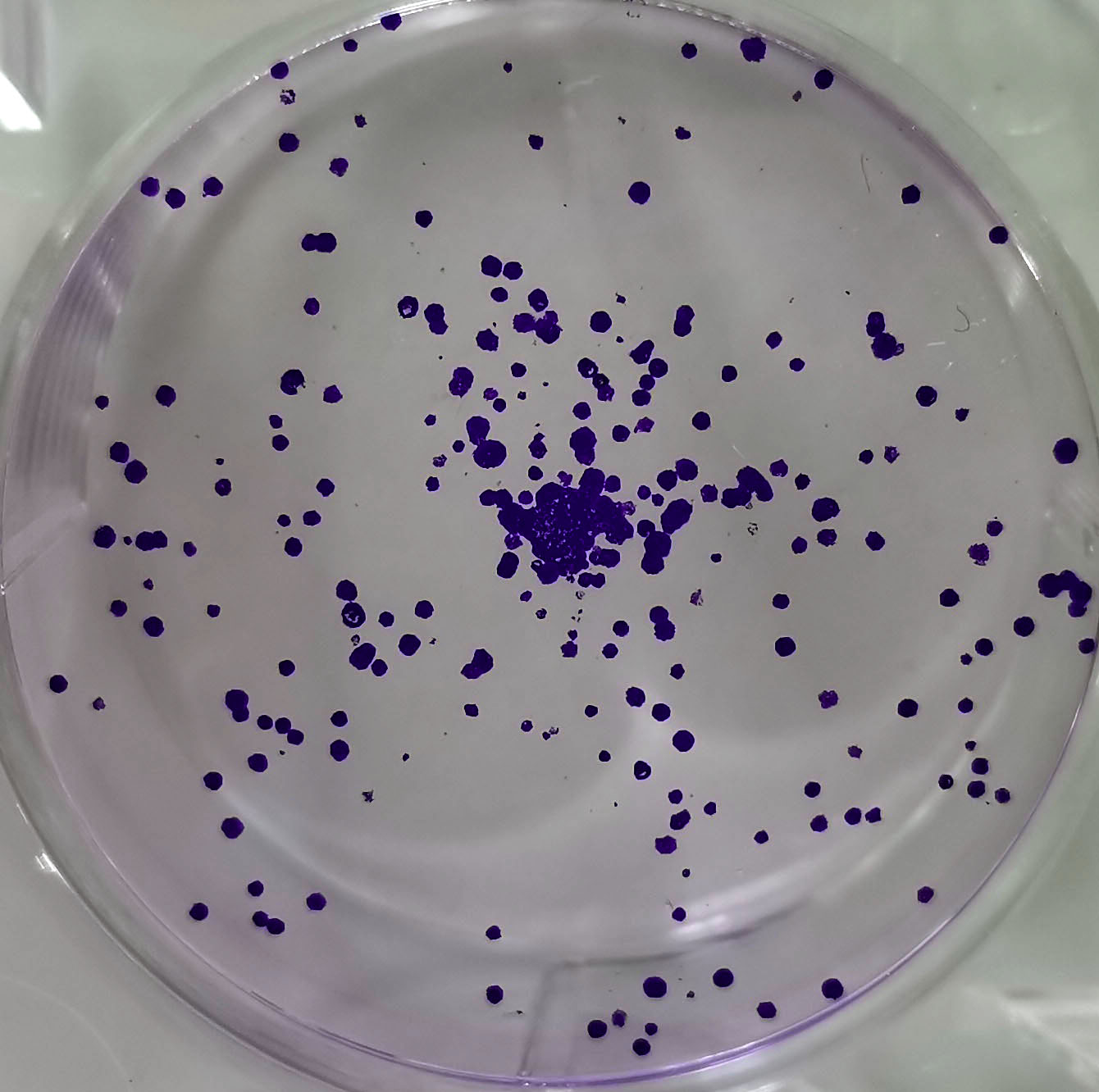

Supplement: Supplemental Information 8 [file peerj-09-11455-s008.zip › fig3D-Cell colony formation/U-20S/si-KIF4A-3.jpg]

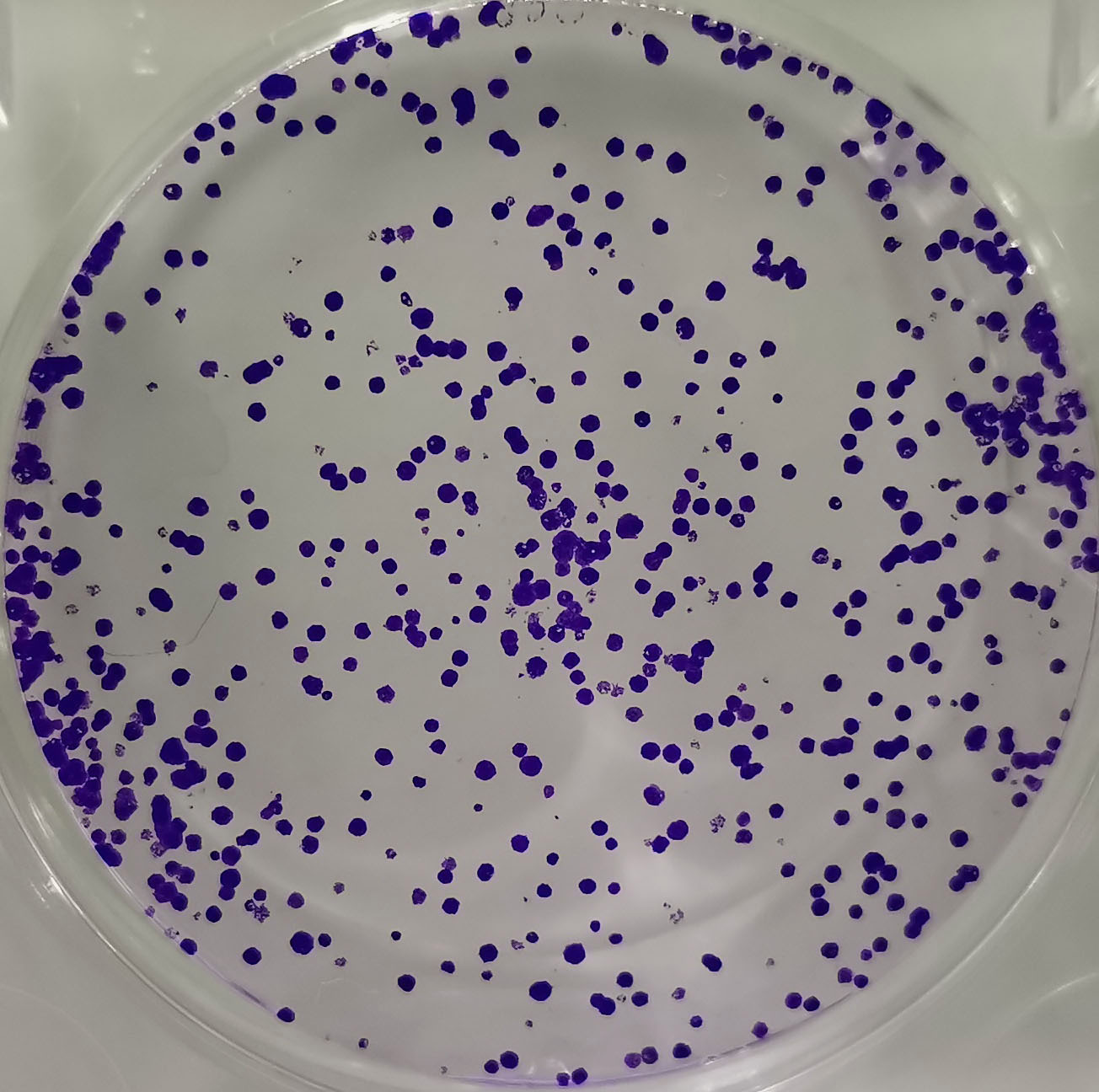

Supplement: Supplemental Information 8 [file peerj-09-11455-s008.zip › fig3D-Cell colony formation/U-20S/si-NC-1.jpg]

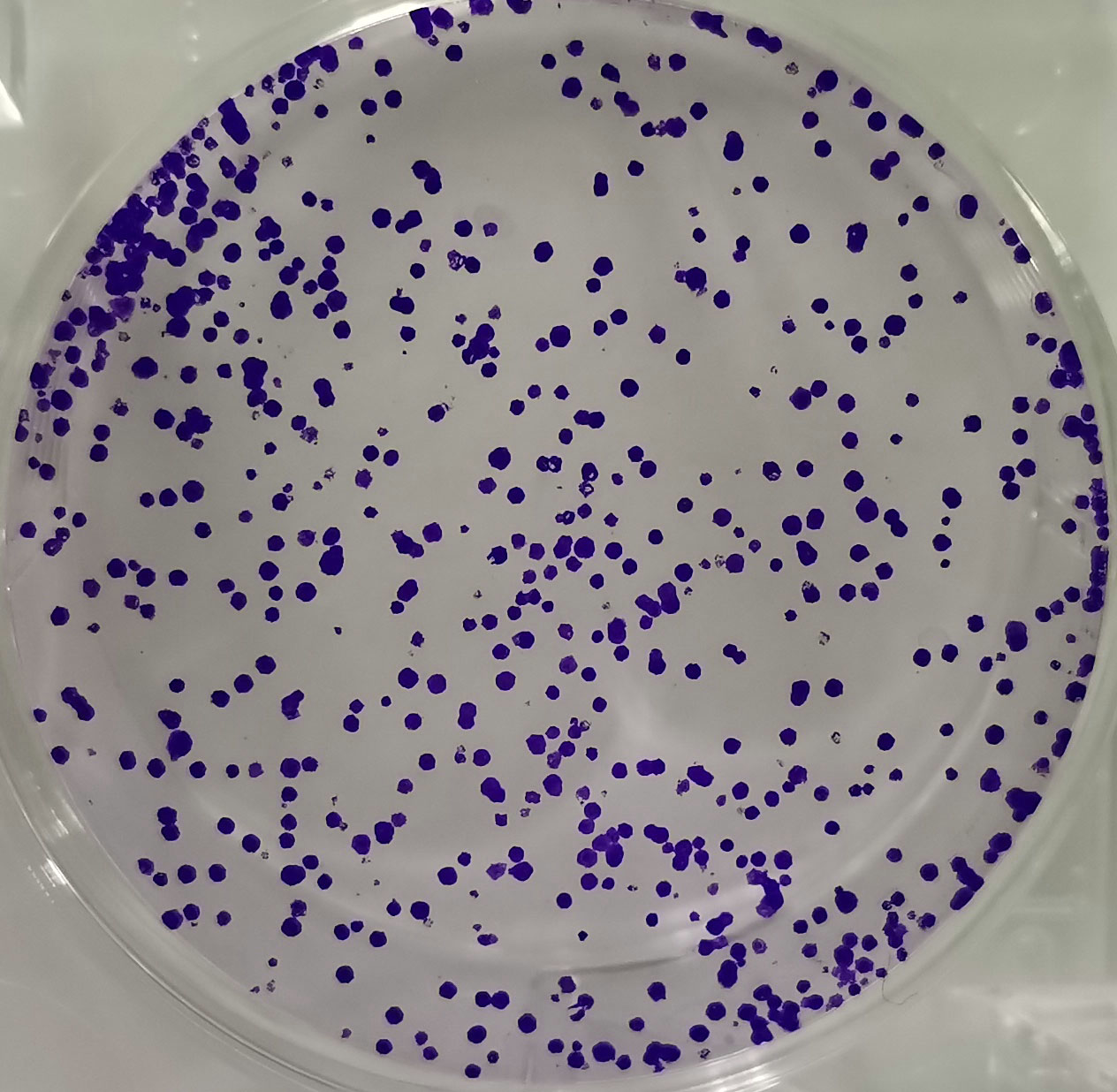

Supplement: Supplemental Information 8 [file peerj-09-11455-s008.zip › fig3D-Cell colony formation/U-20S/si-NC-2.jpg]

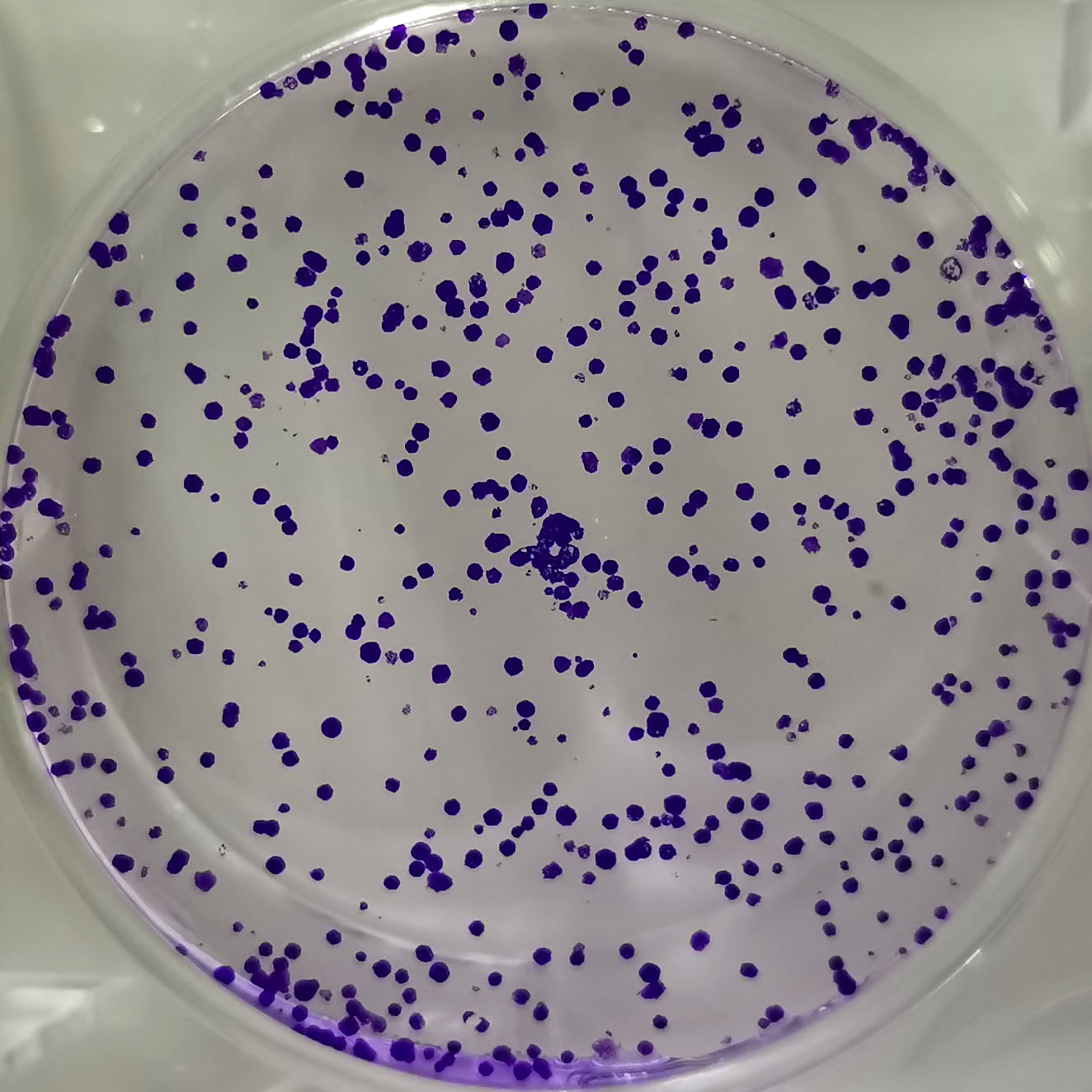

Supplement: Supplemental Information 8 [file peerj-09-11455-s008.zip › fig3D-Cell colony formation/U-20S/si-NC-3.jpg]

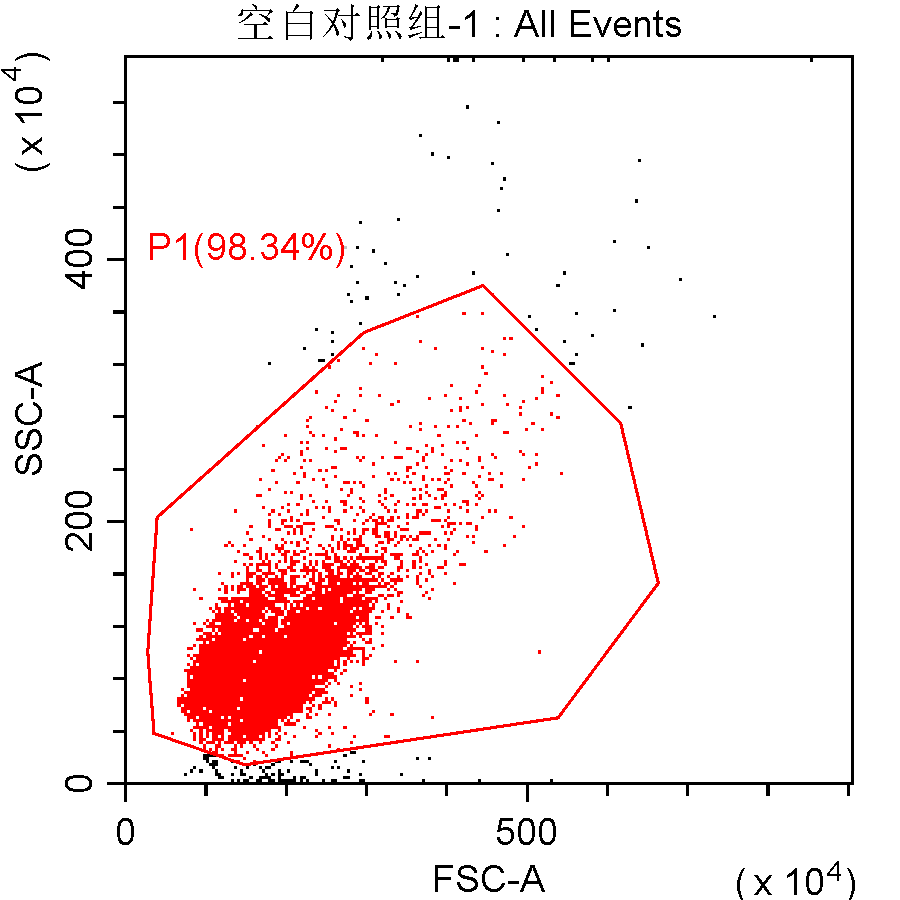

Supplement: Supplemental Information 11 [file peerj-09-11455-s011.zip › fig5A-Apoptosis/MG63/Control-1_Plot1.bmp]

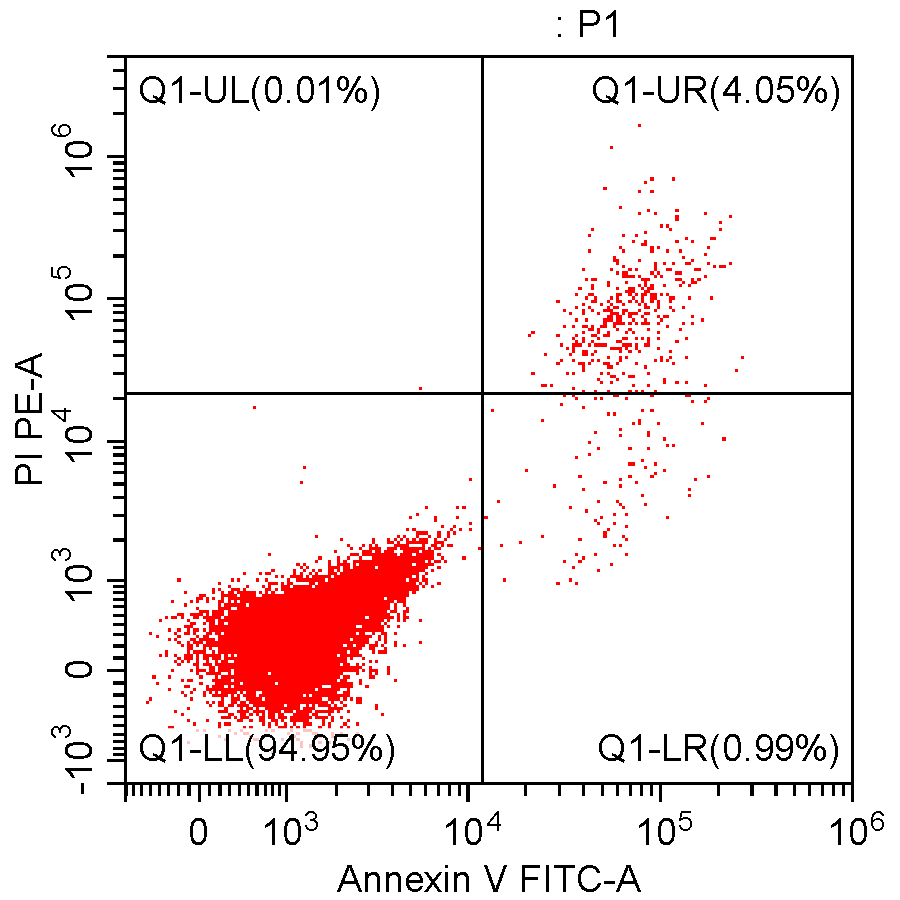

Supplement: Supplemental Information 11 [file peerj-09-11455-s011.zip › fig5A-Apoptosis/MG63/Control-1_Plot2.bmp]

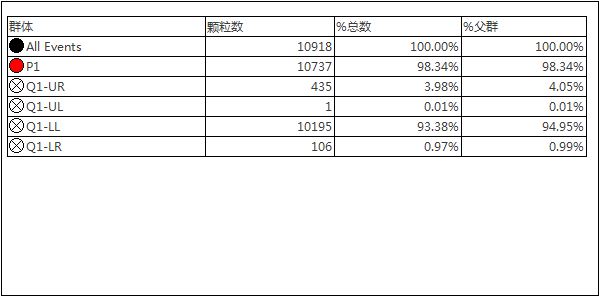

Supplement: Supplemental Information 11 [file peerj-09-11455-s011.zip › fig5A-Apoptosis/MG63/Control-1_Statistics1.bmp]

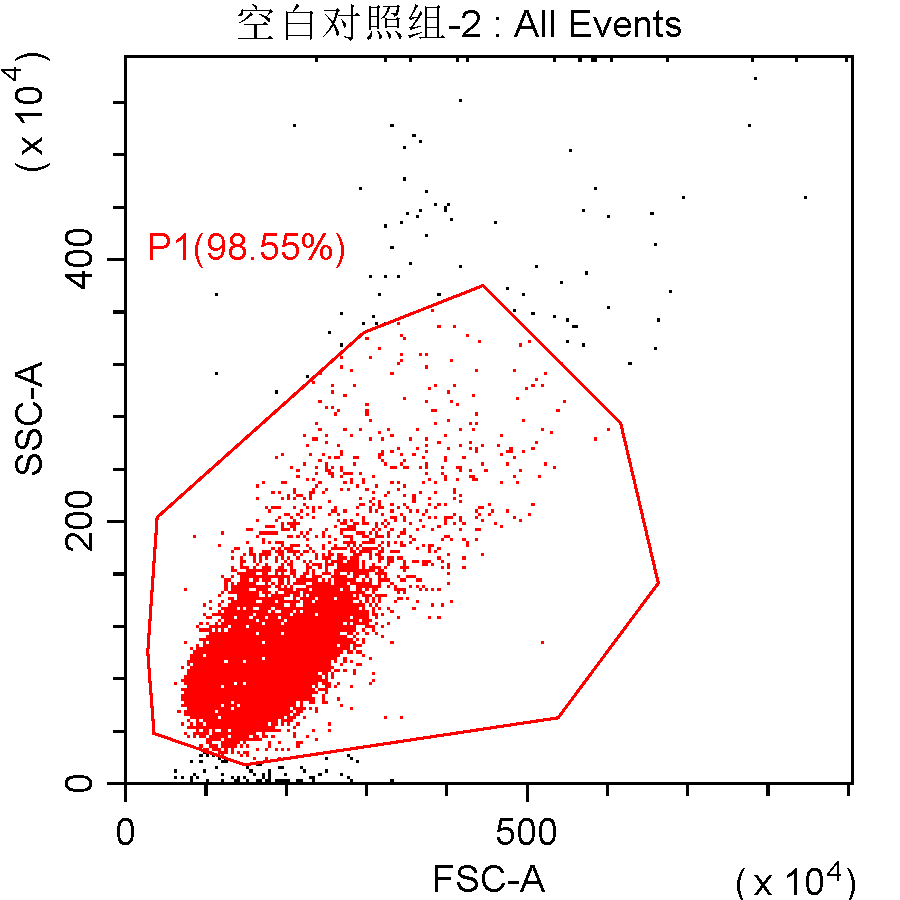

Supplement: Supplemental Information 11 [file peerj-09-11455-s011.zip › fig5A-Apoptosis/MG63/Control-2_Plot1.bmp]

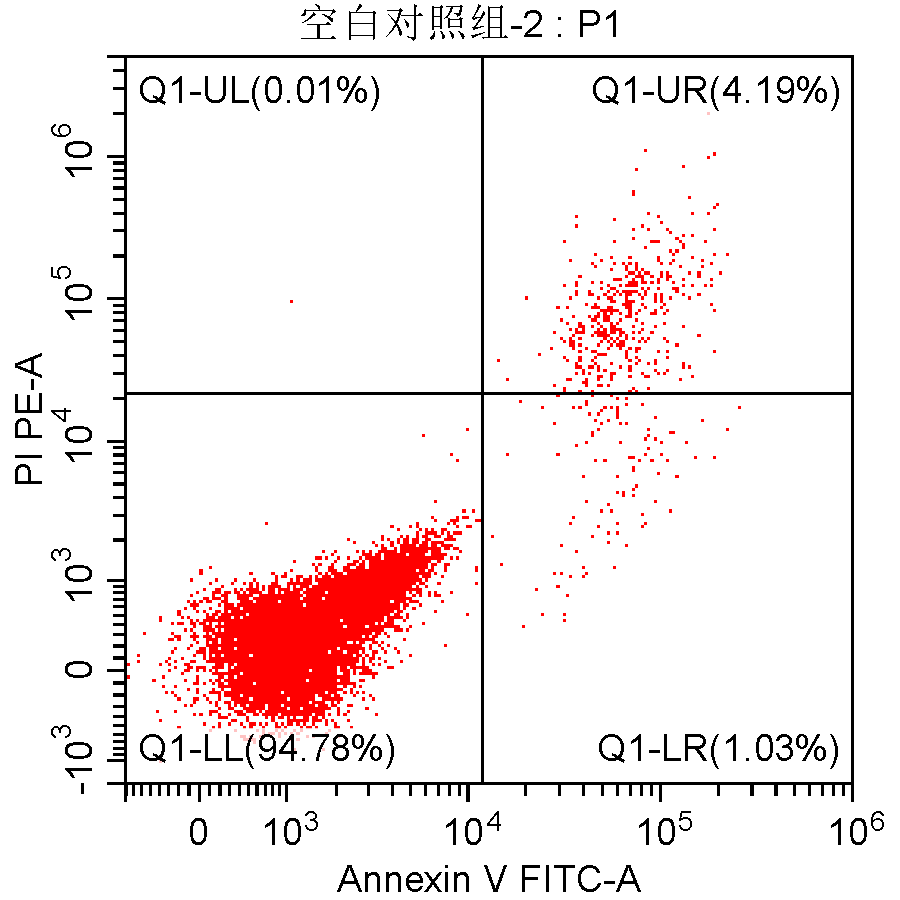

Supplement: Supplemental Information 11 [file peerj-09-11455-s011.zip › fig5A-Apoptosis/MG63/Control-2_Plot2.bmp]

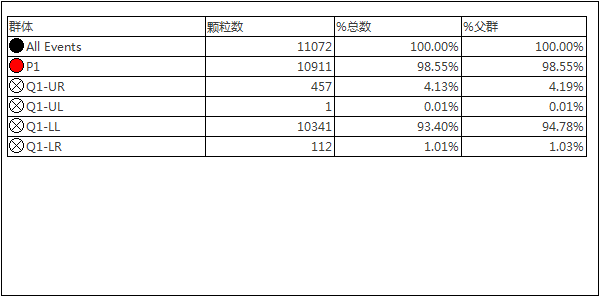

Supplement: Supplemental Information 11 [file peerj-09-11455-s011.zip › fig5A-Apoptosis/MG63/Control-2_Statistics1.bmp]

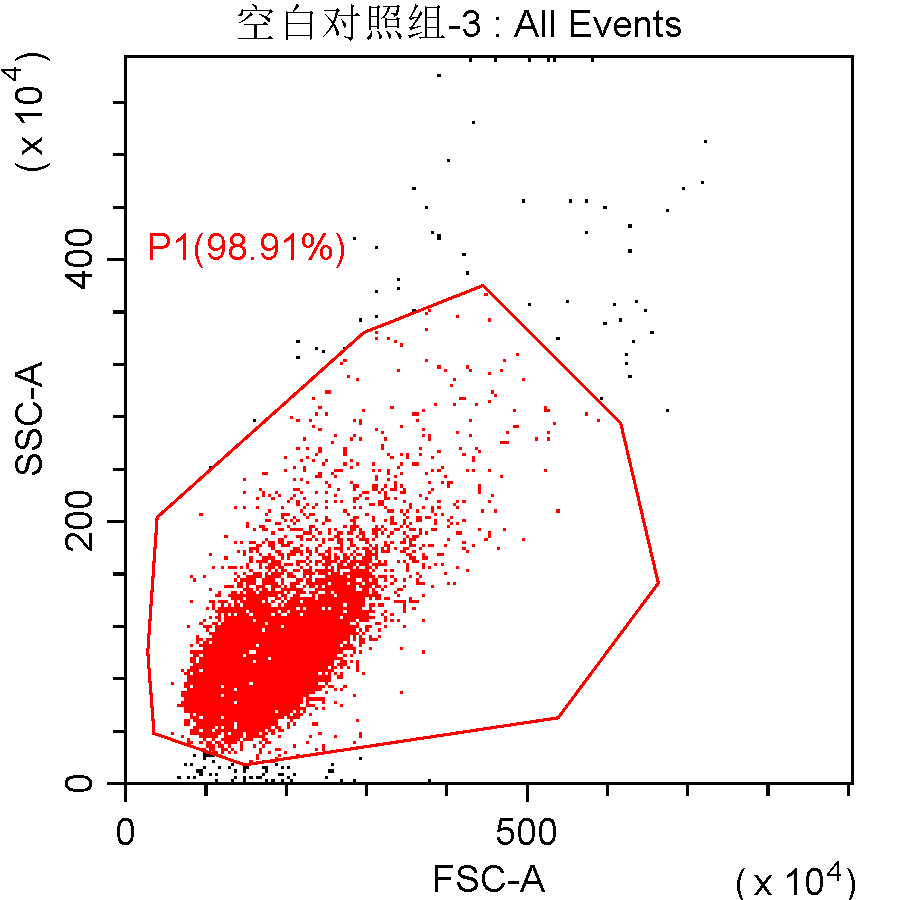

Supplement: Supplemental Information 11 [file peerj-09-11455-s011.zip › fig5A-Apoptosis/MG63/Control-3_Plot1.bmp]

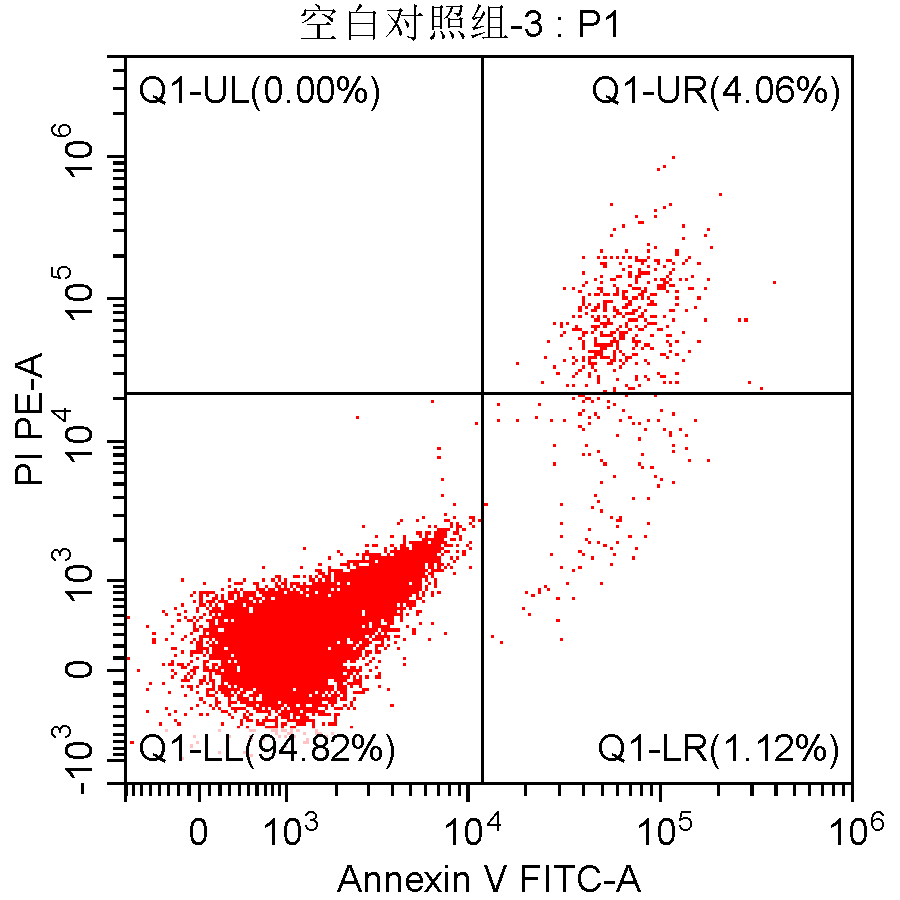

Supplement: Supplemental Information 11 [file peerj-09-11455-s011.zip › fig5A-Apoptosis/MG63/Control-3_Plot2.bmp]

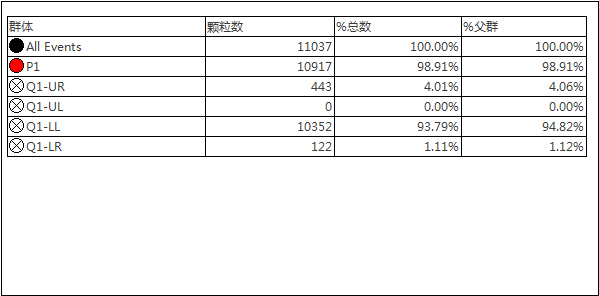

Supplement: Supplemental Information 11 [file peerj-09-11455-s011.zip › fig5A-Apoptosis/MG63/Control-3_Statistics1.bmp]

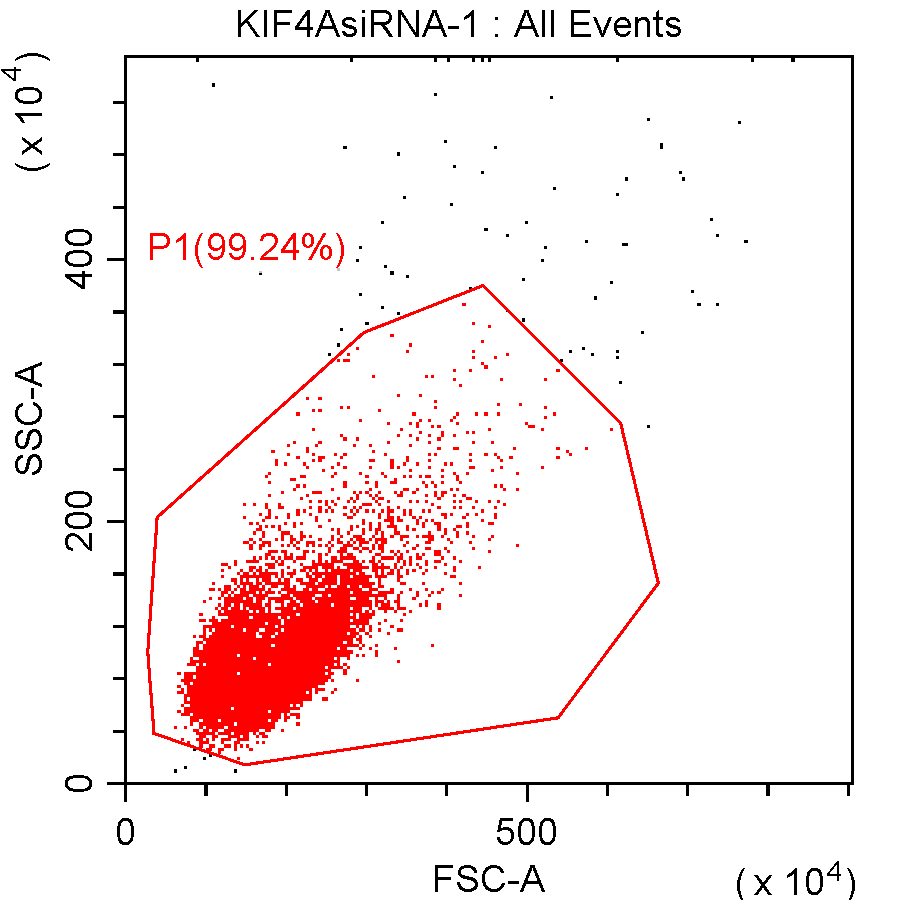

Supplement: Supplemental Information 11 [file peerj-09-11455-s011.zip › fig5A-Apoptosis/MG63/KIF4AsiRNA-1_Plot1.bmp]

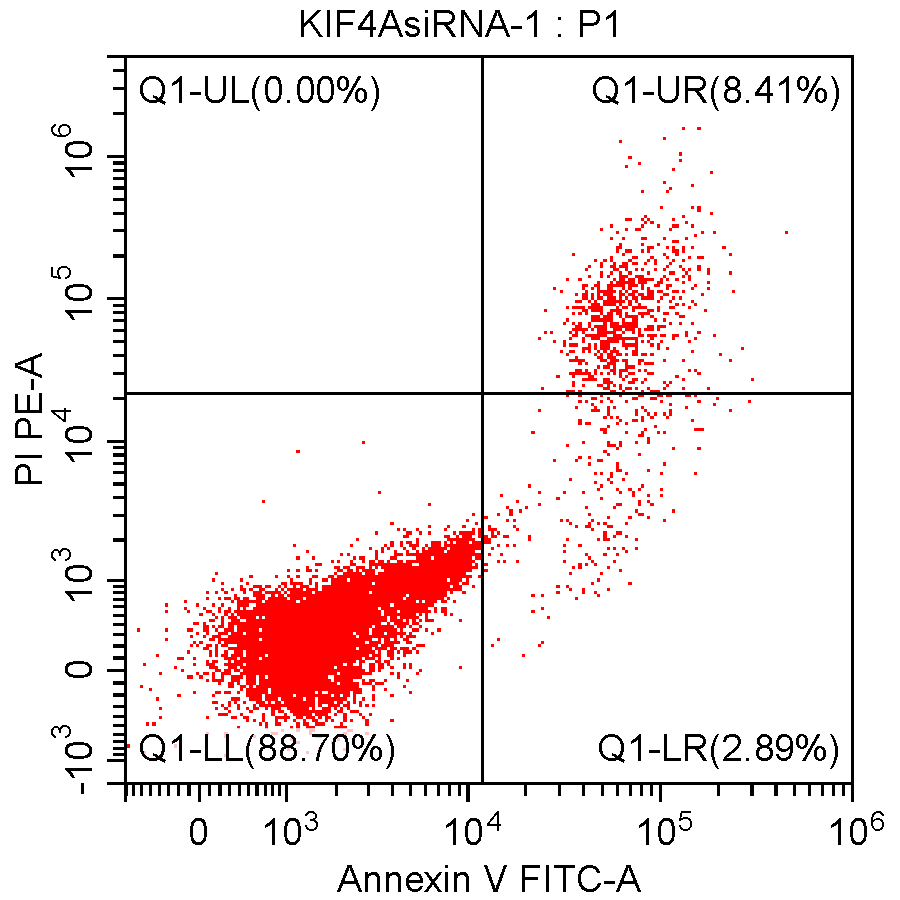

Supplement: Supplemental Information 11 [file peerj-09-11455-s011.zip › fig5A-Apoptosis/MG63/KIF4AsiRNA-1_Plot2.bmp]

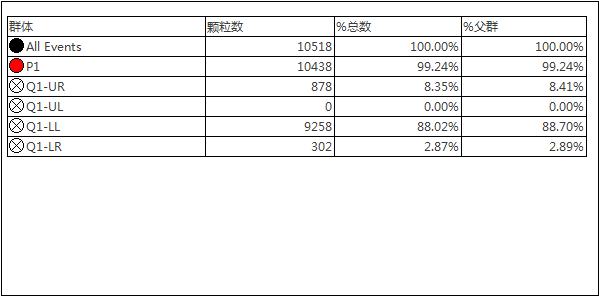

Supplement: Supplemental Information 11 [file peerj-09-11455-s011.zip › fig5A-Apoptosis/MG63/KIF4AsiRNA-1_Statistics1.bmp]

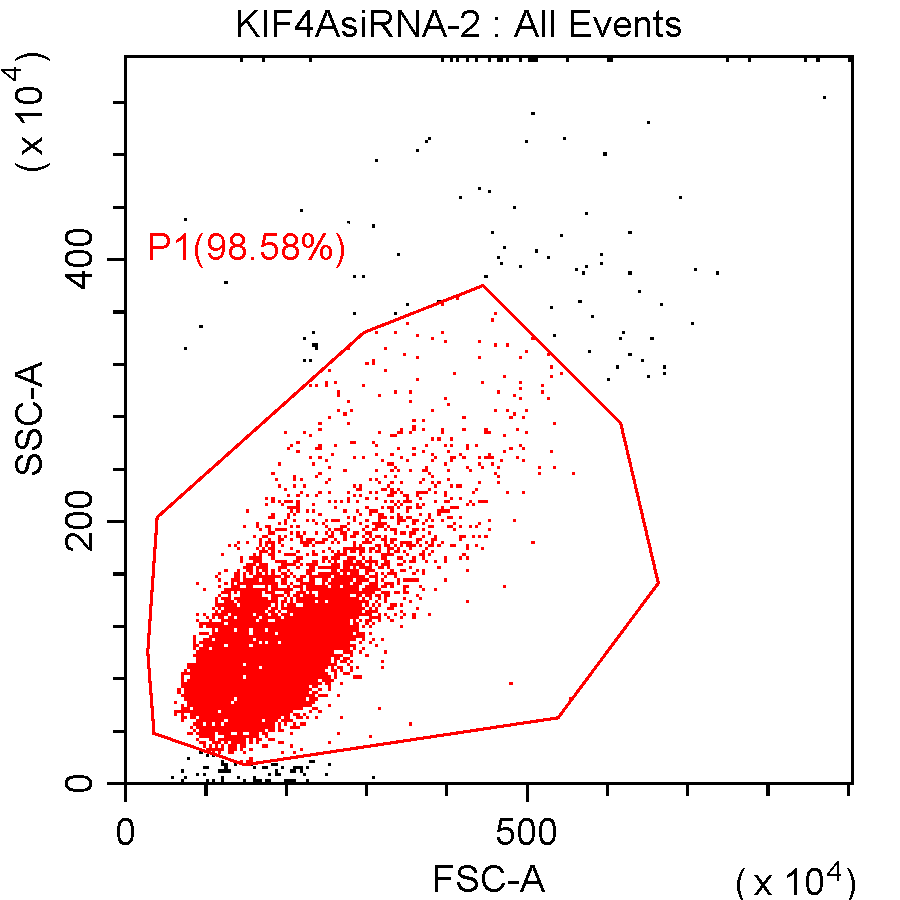

Supplement: Supplemental Information 11 [file peerj-09-11455-s011.zip › fig5A-Apoptosis/MG63/KIF4AsiRNA-2_Plot1.bmp]

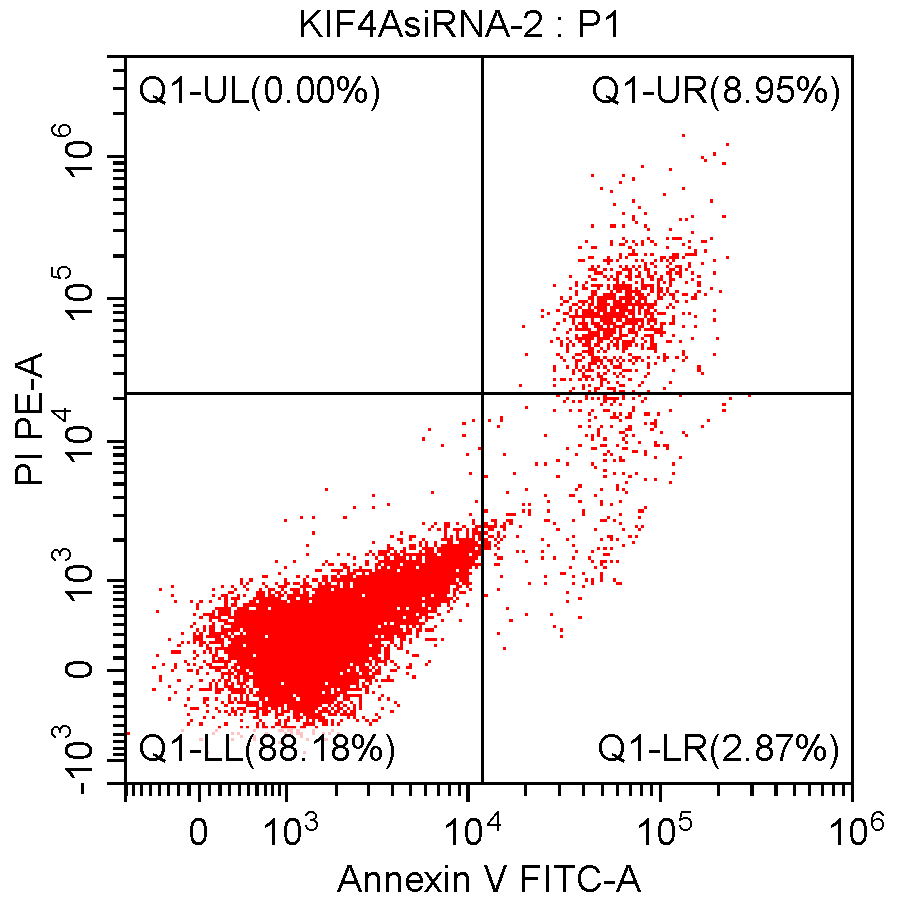

Supplement: Supplemental Information 11 [file peerj-09-11455-s011.zip › fig5A-Apoptosis/MG63/KIF4AsiRNA-2_Plot2.bmp]

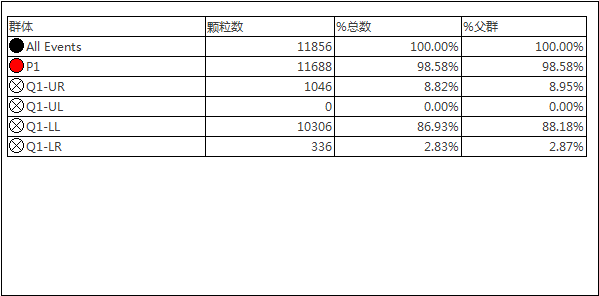

Supplement: Supplemental Information 11 [file peerj-09-11455-s011.zip › fig5A-Apoptosis/MG63/KIF4AsiRNA-2_Statistics1.bmp]

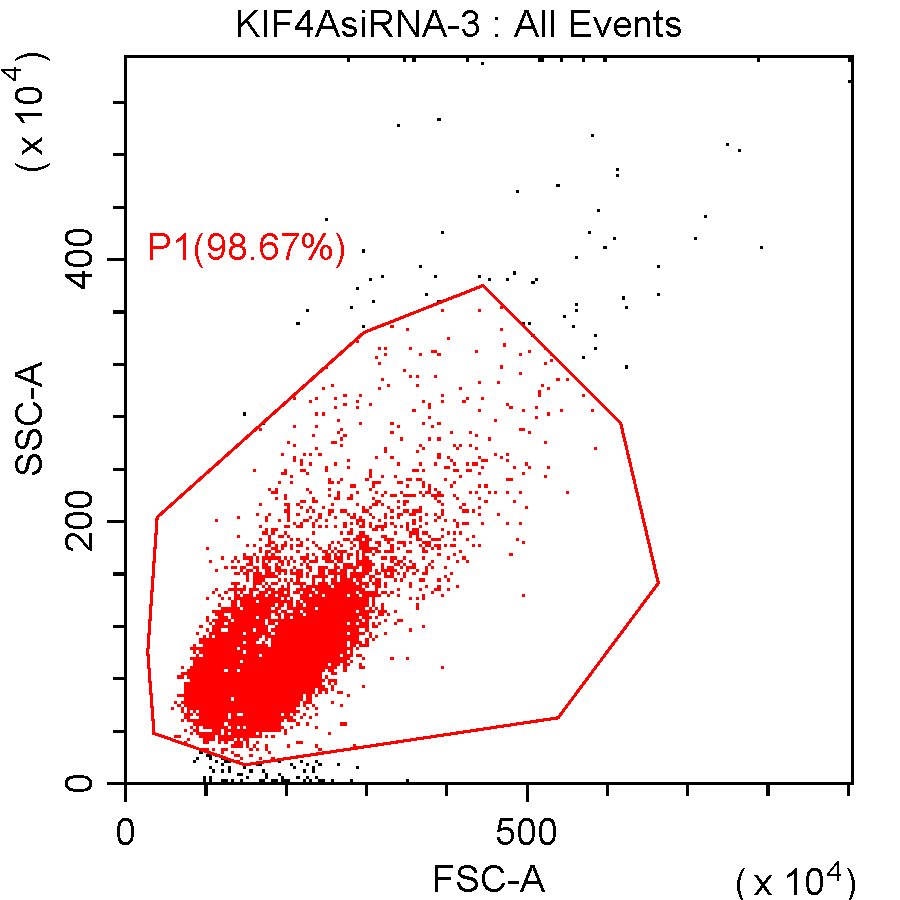

Supplement: Supplemental Information 11 [file peerj-09-11455-s011.zip › fig5A-Apoptosis/MG63/KIF4AsiRNA-3_Plot1.bmp]

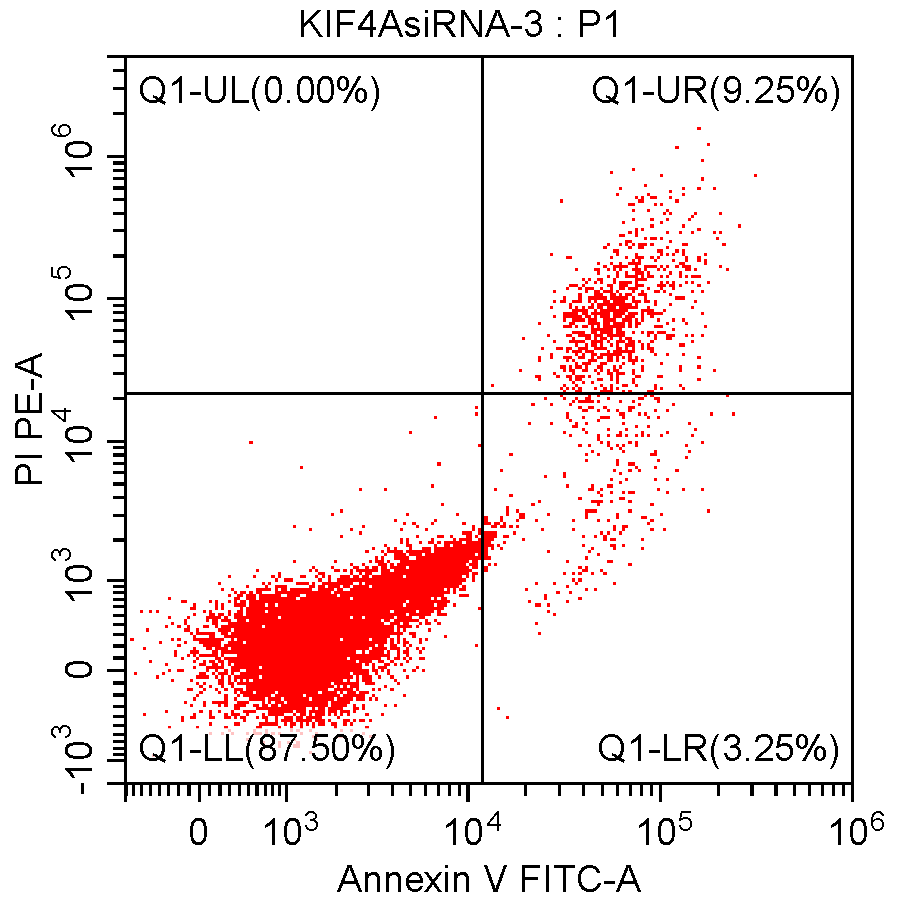

Supplement: Supplemental Information 11 [file peerj-09-11455-s011.zip › fig5A-Apoptosis/MG63/KIF4AsiRNA-3_Plot2.bmp]

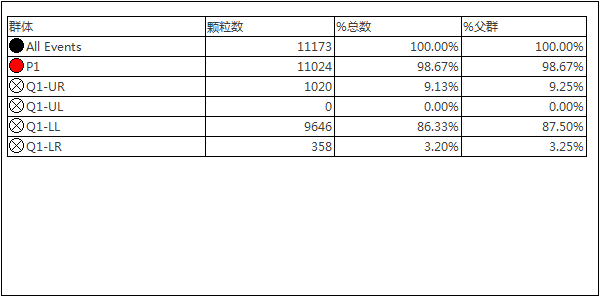

Supplement: Supplemental Information 11 [file peerj-09-11455-s011.zip › fig5A-Apoptosis/MG63/KIF4AsiRNA-3_Statistics1.bmp]

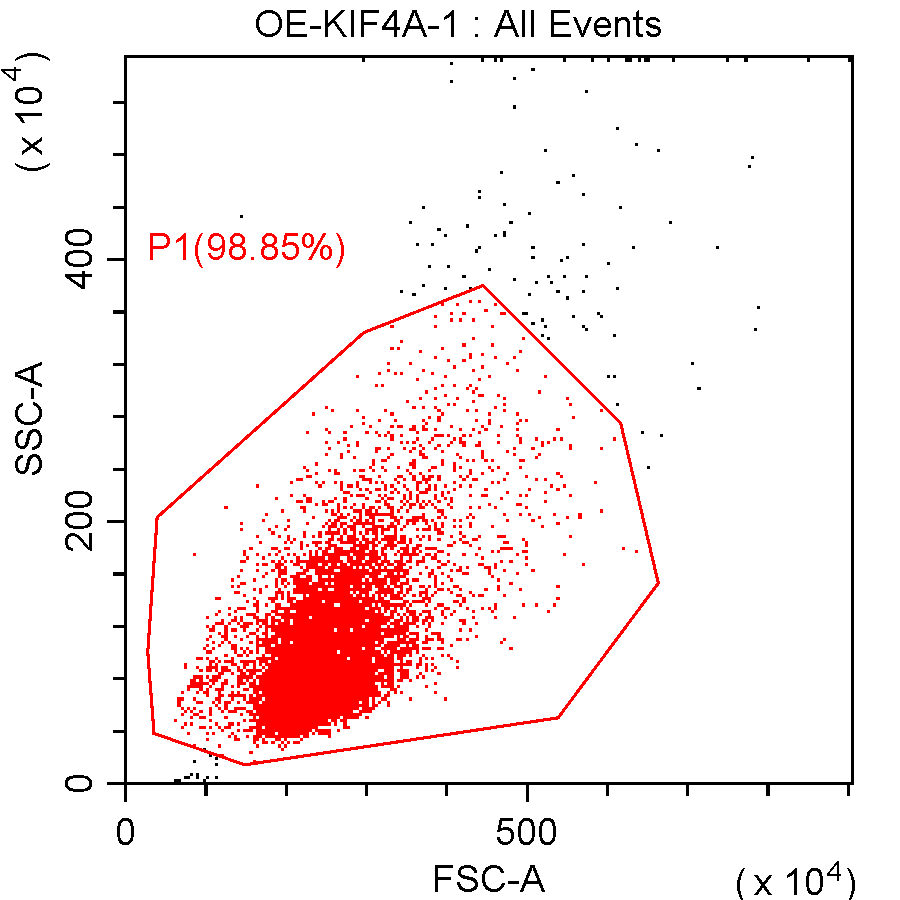

Supplement: Supplemental Information 11 [file peerj-09-11455-s011.zip › fig5A-Apoptosis/MG63/OE-KIF4A-1_Plot1.bmp]

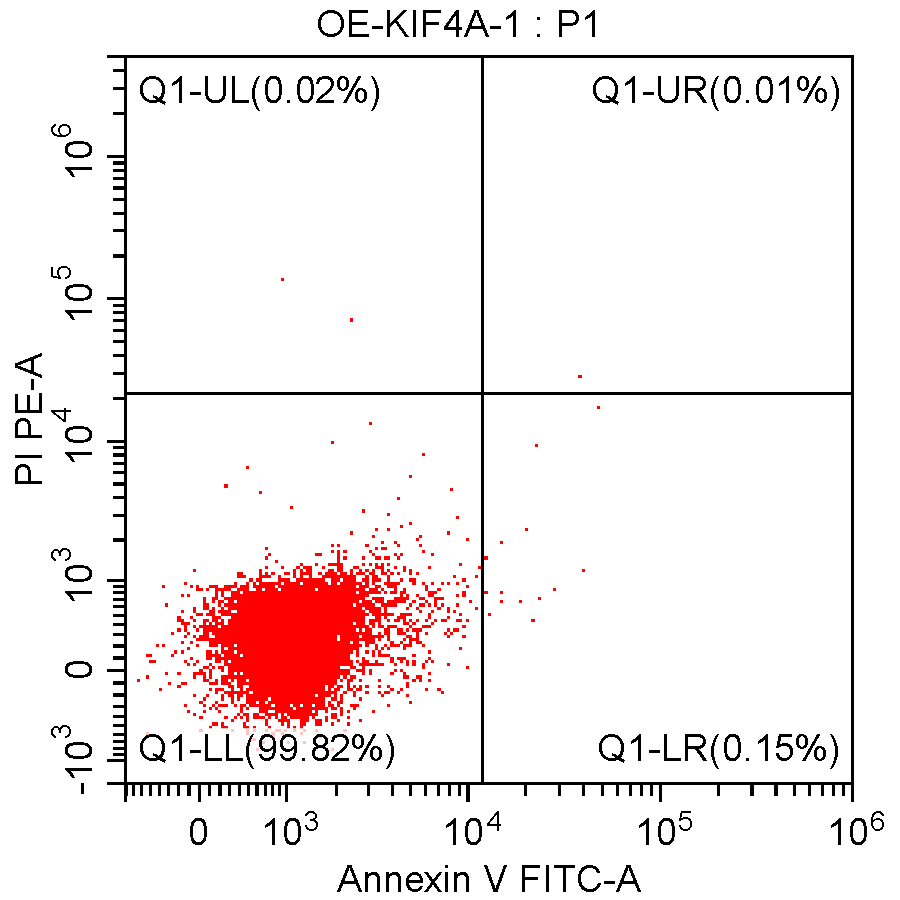

Supplement: Supplemental Information 11 [file peerj-09-11455-s011.zip › fig5A-Apoptosis/MG63/OE-KIF4A-1_Plot2.bmp]

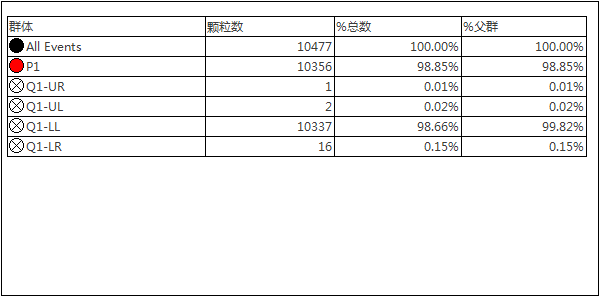

Supplement: Supplemental Information 11 [file peerj-09-11455-s011.zip › fig5A-Apoptosis/MG63/OE-KIF4A-1_Statistics1.bmp]

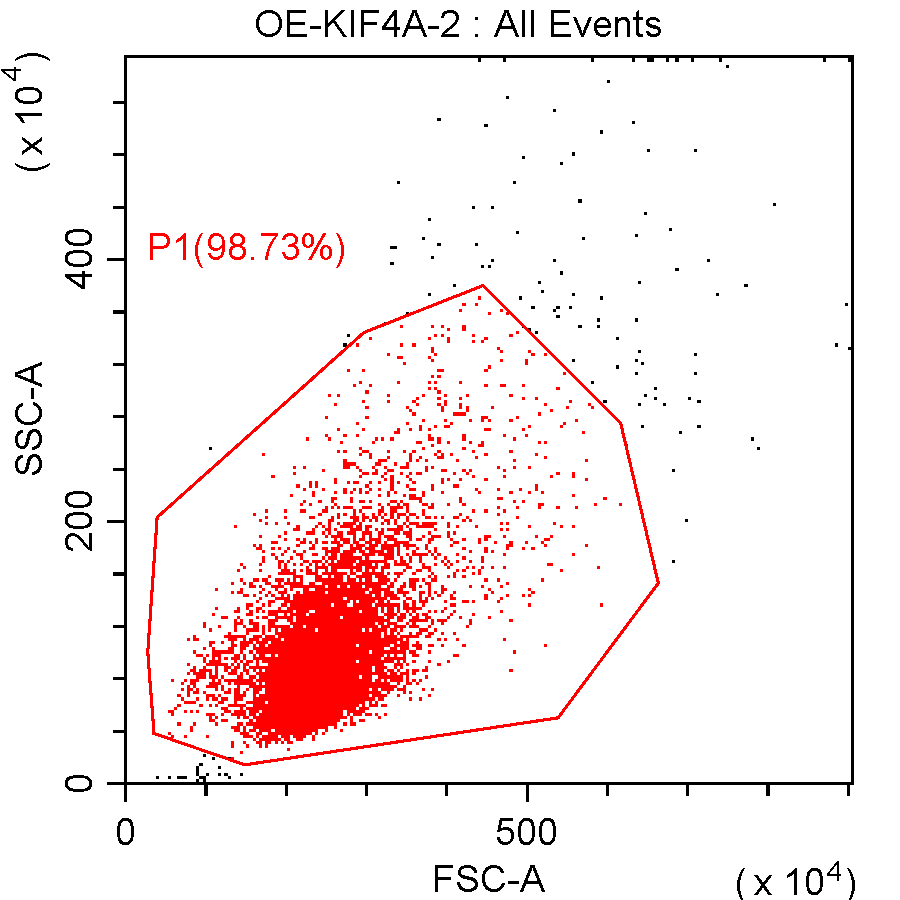

Supplement: Supplemental Information 11 [file peerj-09-11455-s011.zip › fig5A-Apoptosis/MG63/OE-KIF4A-2_Plot1.bmp]

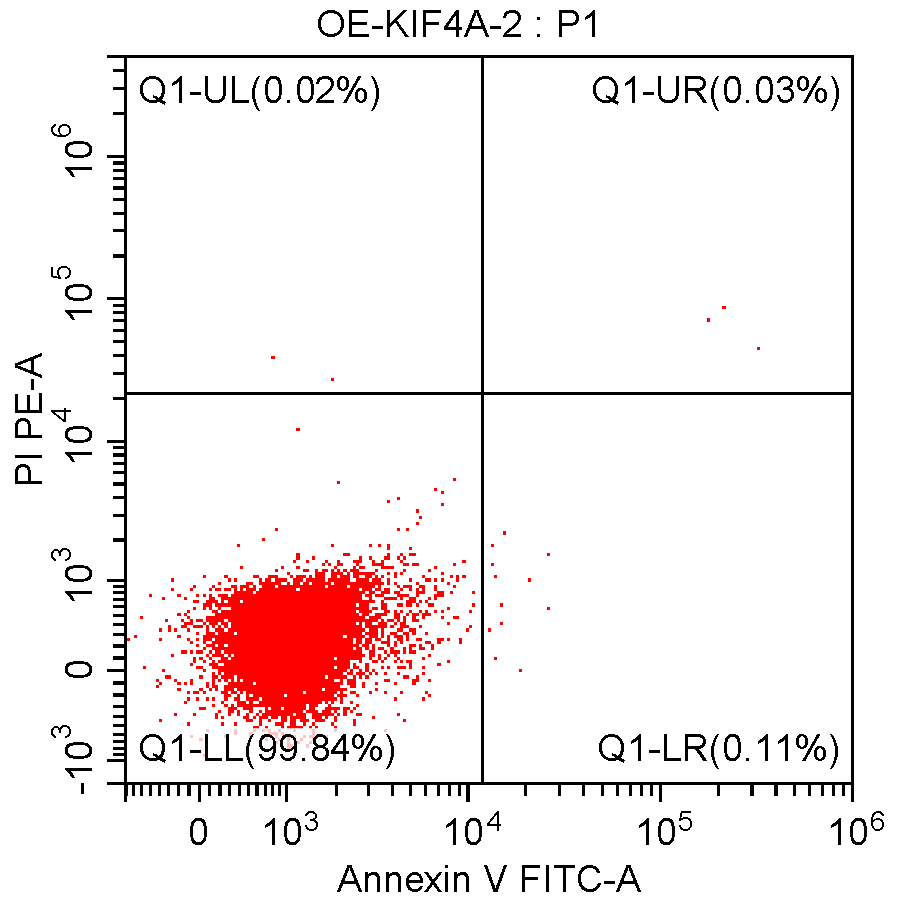

Supplement: Supplemental Information 11 [file peerj-09-11455-s011.zip › fig5A-Apoptosis/MG63/OE-KIF4A-2_Plot2.bmp]

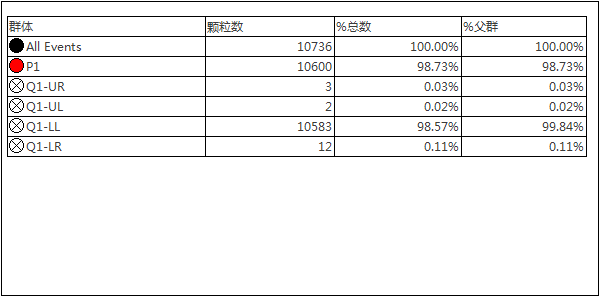

Supplement: Supplemental Information 11 [file peerj-09-11455-s011.zip › fig5A-Apoptosis/MG63/OE-KIF4A-2_Statistics1.bmp]

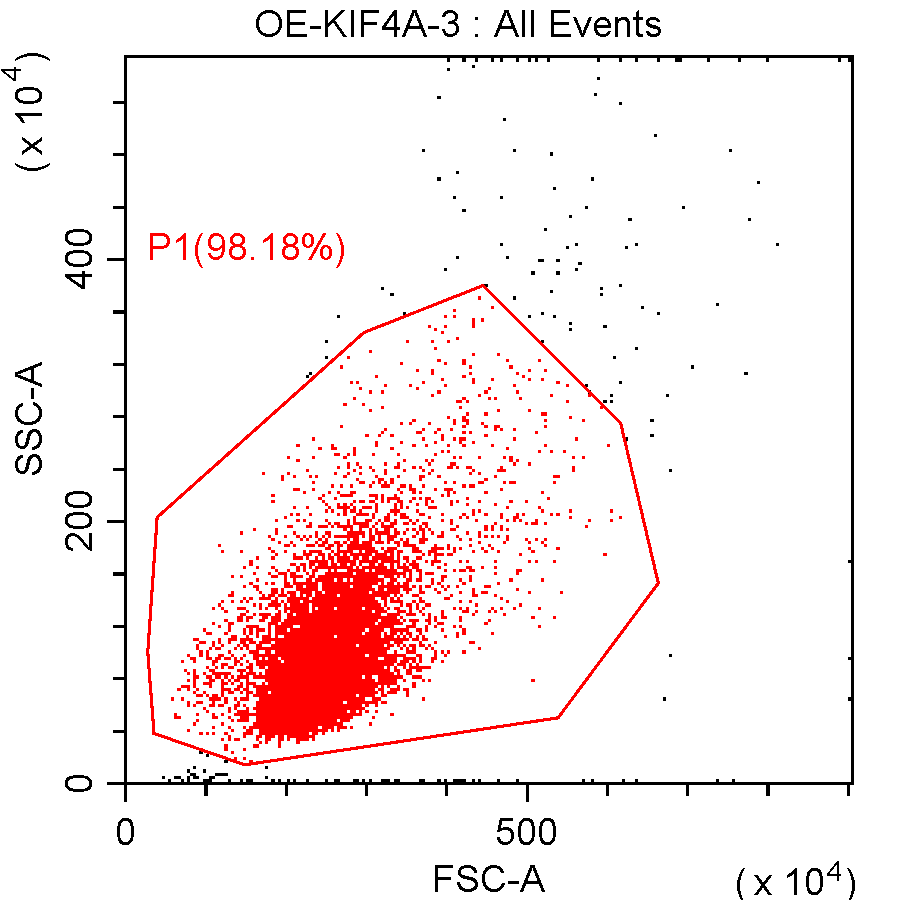

Supplement: Supplemental Information 11 [file peerj-09-11455-s011.zip › fig5A-Apoptosis/MG63/OE-KIF4A-3_Plot1.bmp]

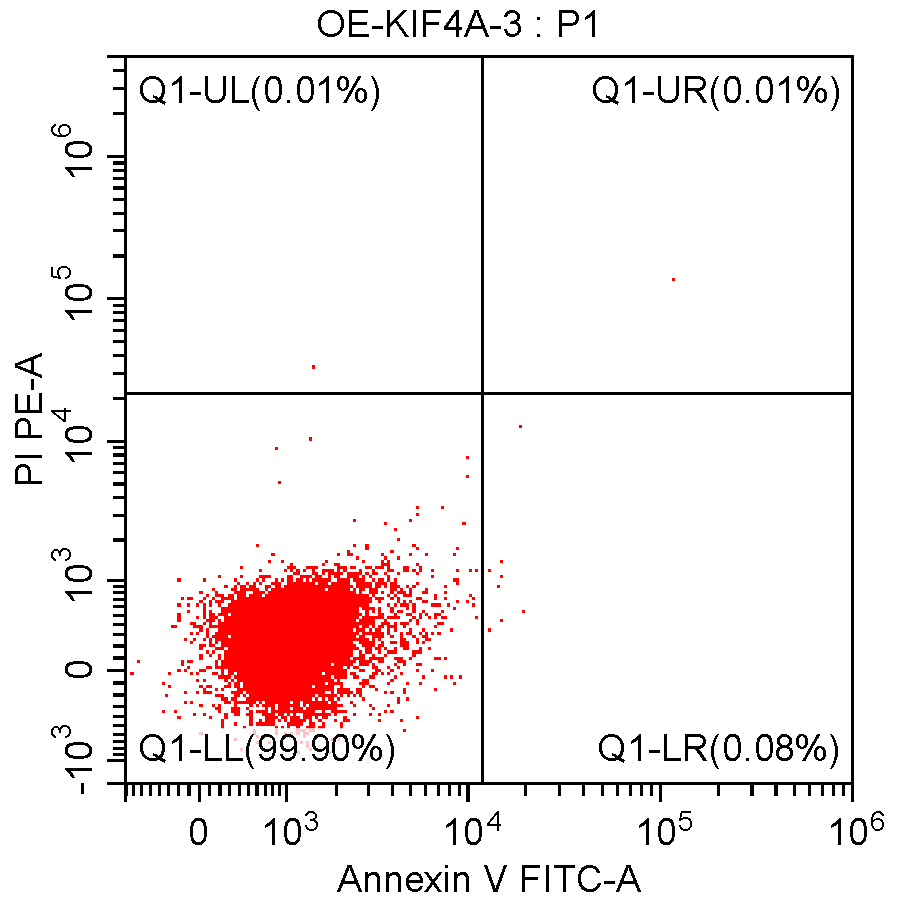

Supplement: Supplemental Information 11 [file peerj-09-11455-s011.zip › fig5A-Apoptosis/MG63/OE-KIF4A-3_Plot2.bmp]

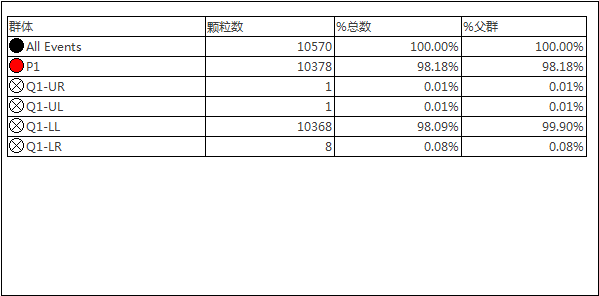

Supplement: Supplemental Information 11 [file peerj-09-11455-s011.zip › fig5A-Apoptosis/MG63/OE-KIF4A-3_Statistics1.bmp]

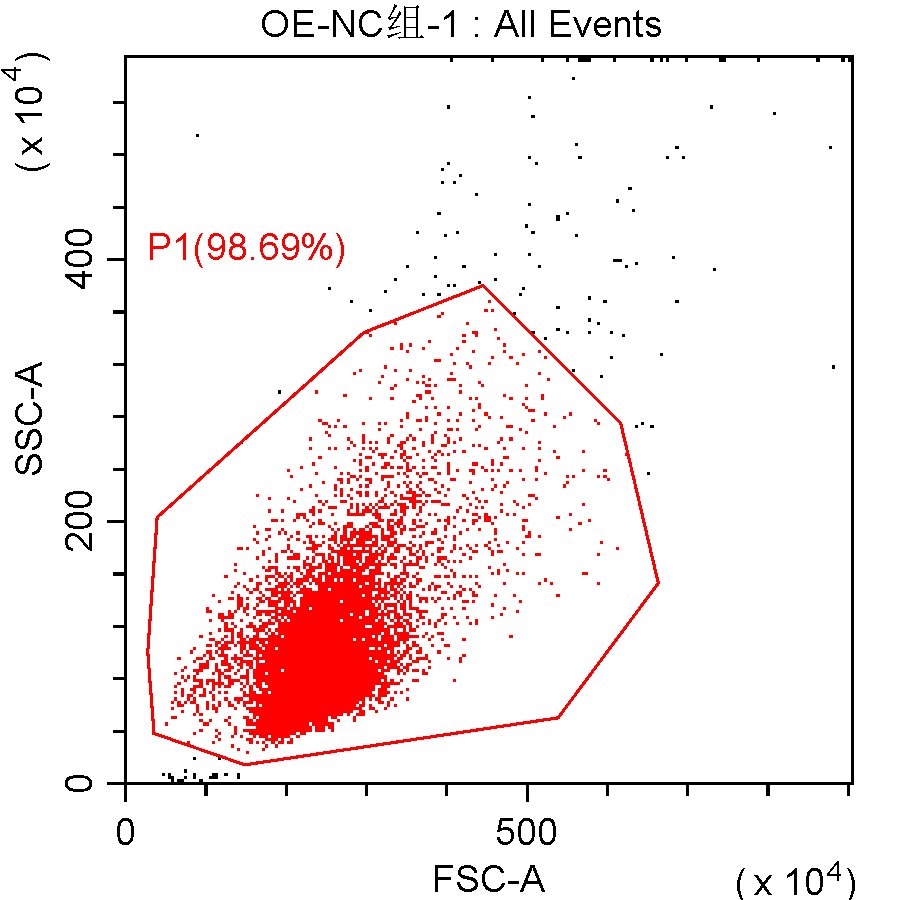

Supplement: Supplemental Information 11 [file peerj-09-11455-s011.zip › fig5A-Apoptosis/MG63/OE-NC组-1_Plot1.bmp]

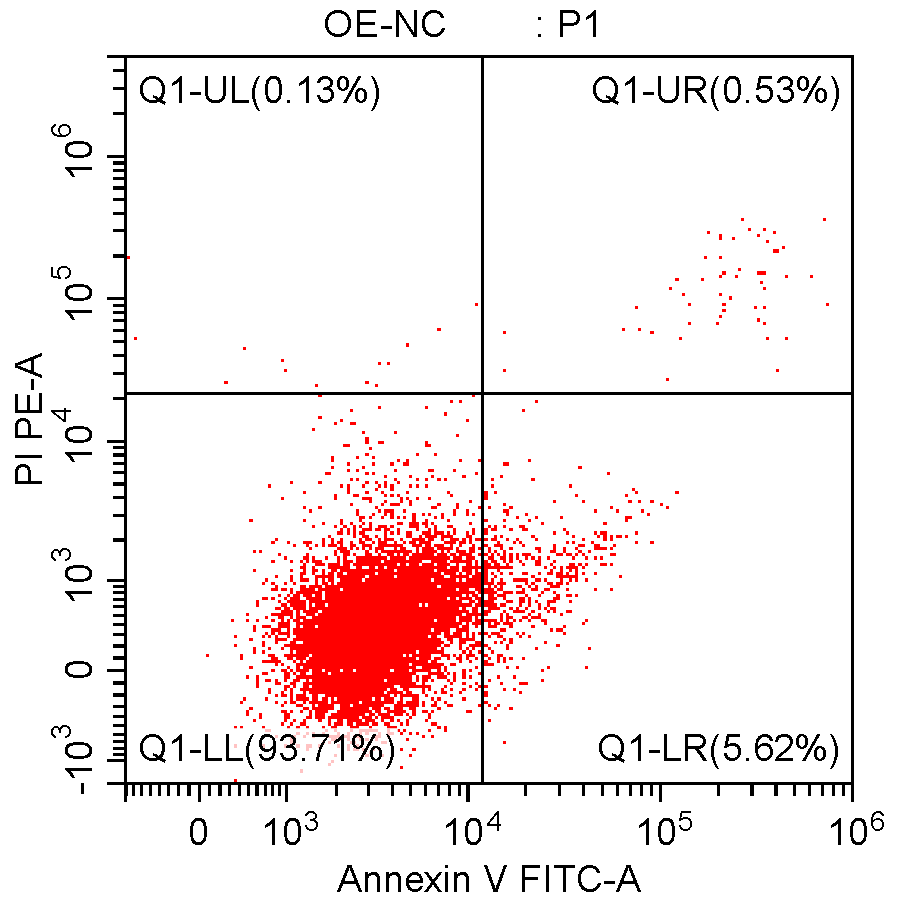

Supplement: Supplemental Information 11 [file peerj-09-11455-s011.zip › fig5A-Apoptosis/MG63/OE-NC组-1_Plot2.bmp]

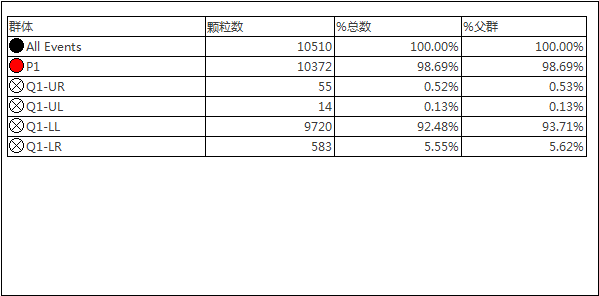

Supplement: Supplemental Information 11 [file peerj-09-11455-s011.zip › fig5A-Apoptosis/MG63/OE-NC组-1_Statistics1.bmp]

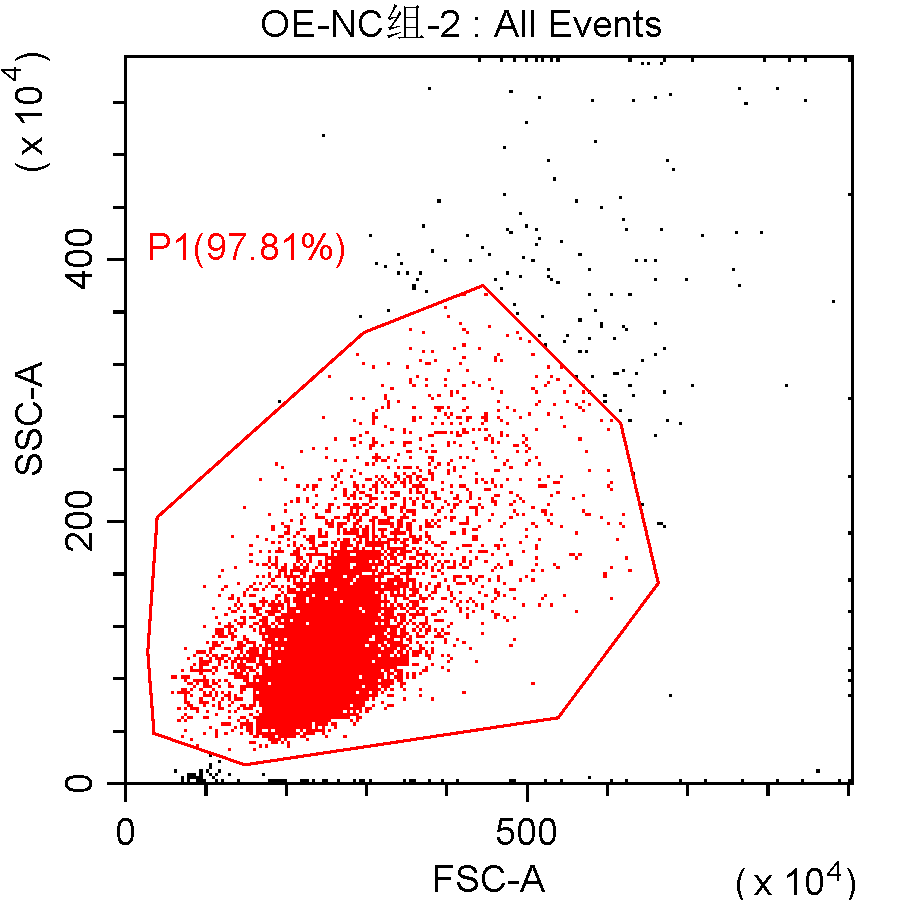

Supplement: Supplemental Information 11 [file peerj-09-11455-s011.zip › fig5A-Apoptosis/MG63/OE-NC组-2_Plot1.bmp]

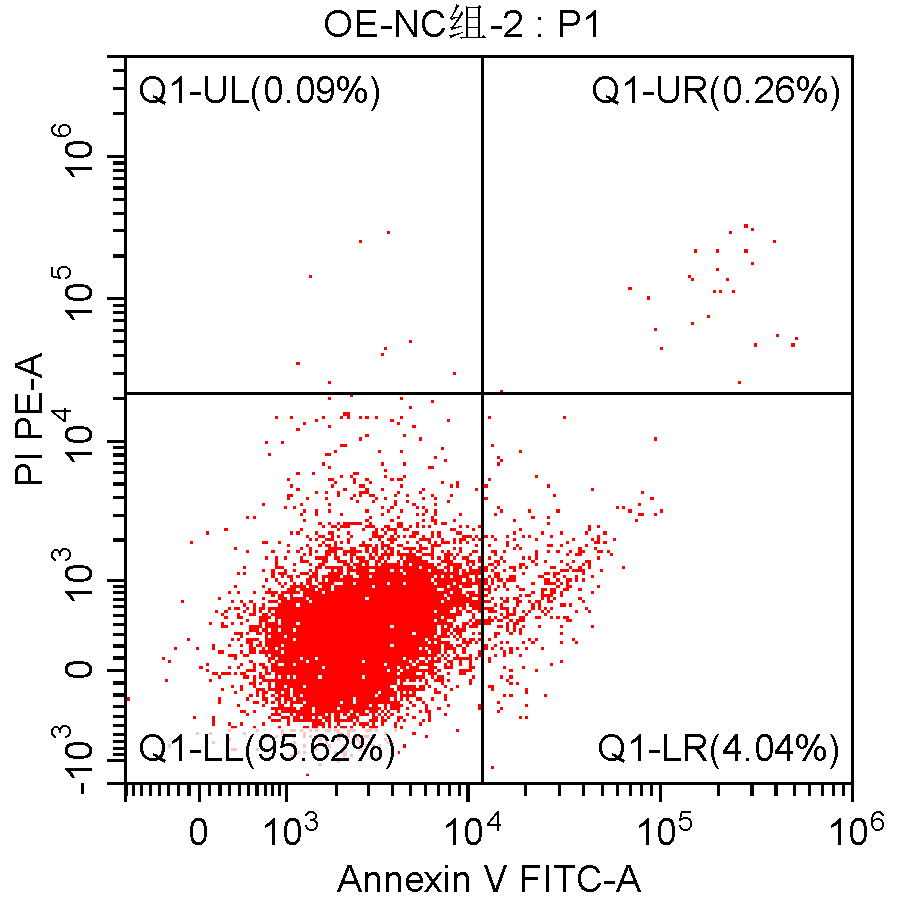

Supplement: Supplemental Information 11 [file peerj-09-11455-s011.zip › fig5A-Apoptosis/MG63/OE-NC组-2_Plot2.bmp]

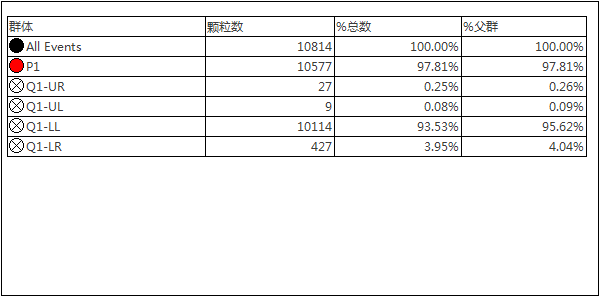

Supplement: Supplemental Information 11 [file peerj-09-11455-s011.zip › fig5A-Apoptosis/MG63/OE-NC组-2_Statistics1.bmp]

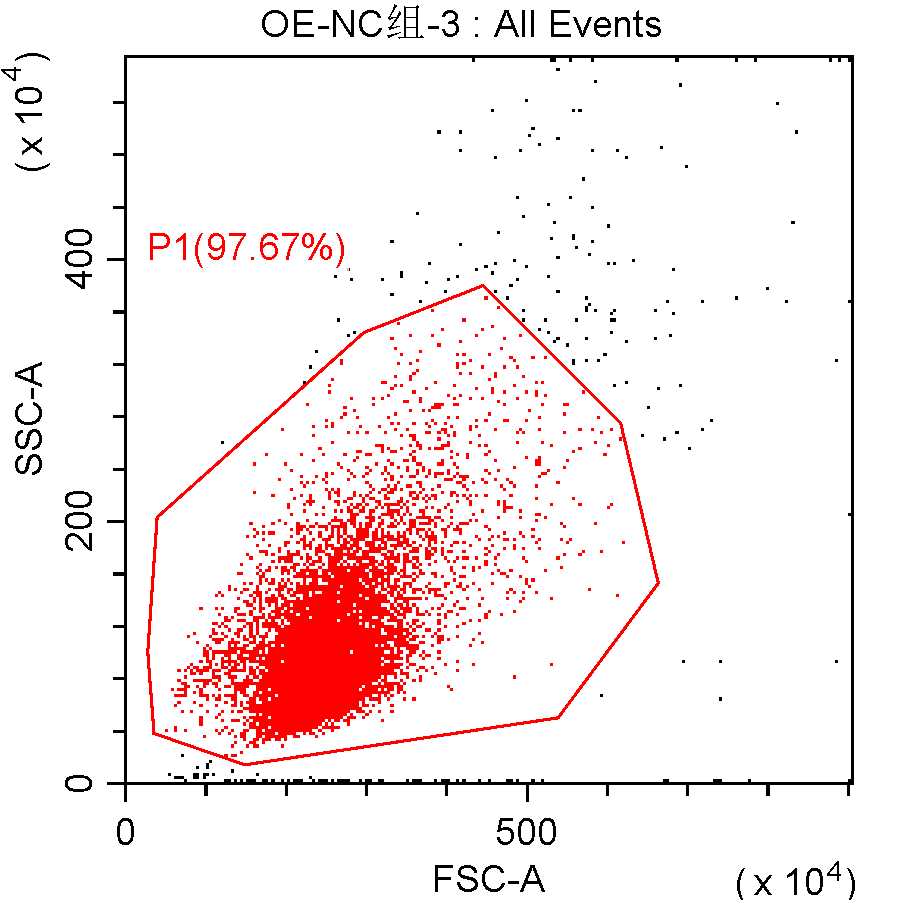

Supplement: Supplemental Information 11 [file peerj-09-11455-s011.zip › fig5A-Apoptosis/MG63/OE-NC组-3_Plot1.bmp]

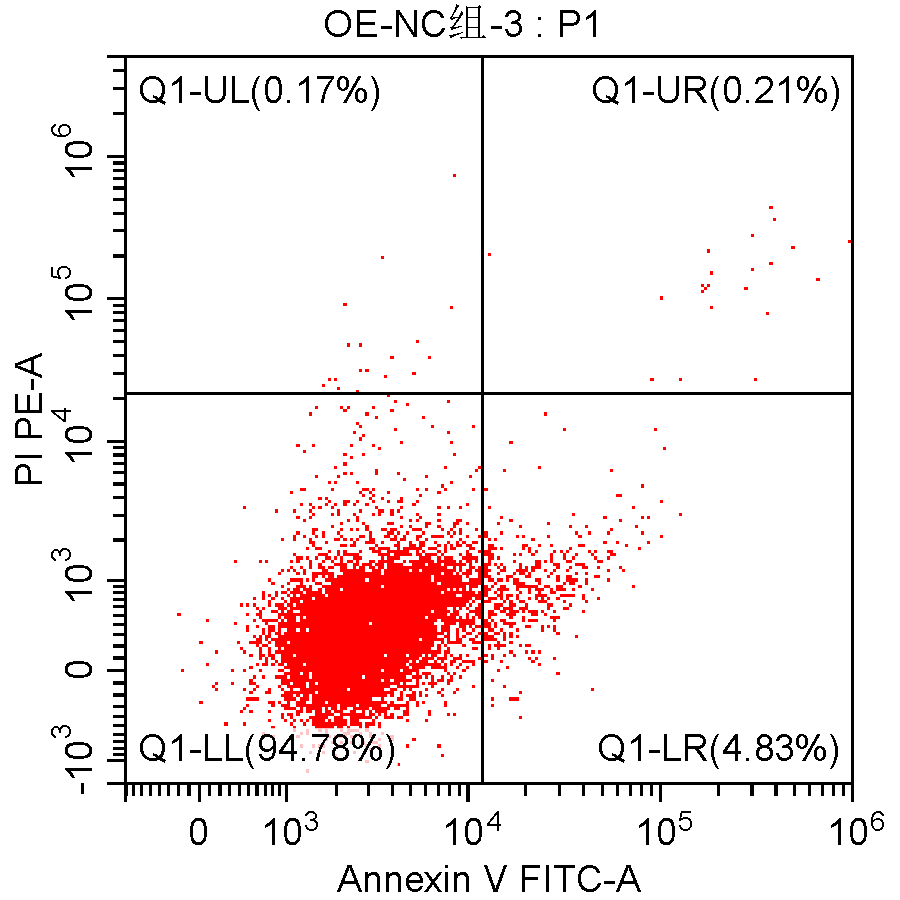

Supplement: Supplemental Information 11 [file peerj-09-11455-s011.zip › fig5A-Apoptosis/MG63/OE-NC组-3_Plot2.bmp]

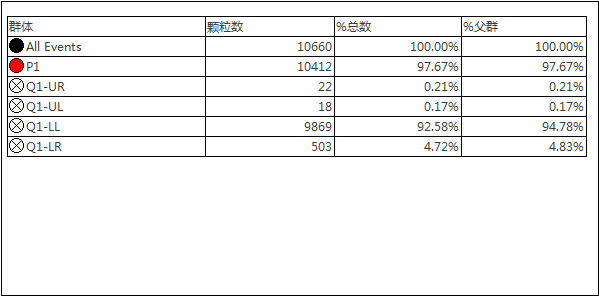

Supplement: Supplemental Information 11 [file peerj-09-11455-s011.zip › fig5A-Apoptosis/MG63/OE-NC组-3_Statistics1.bmp]

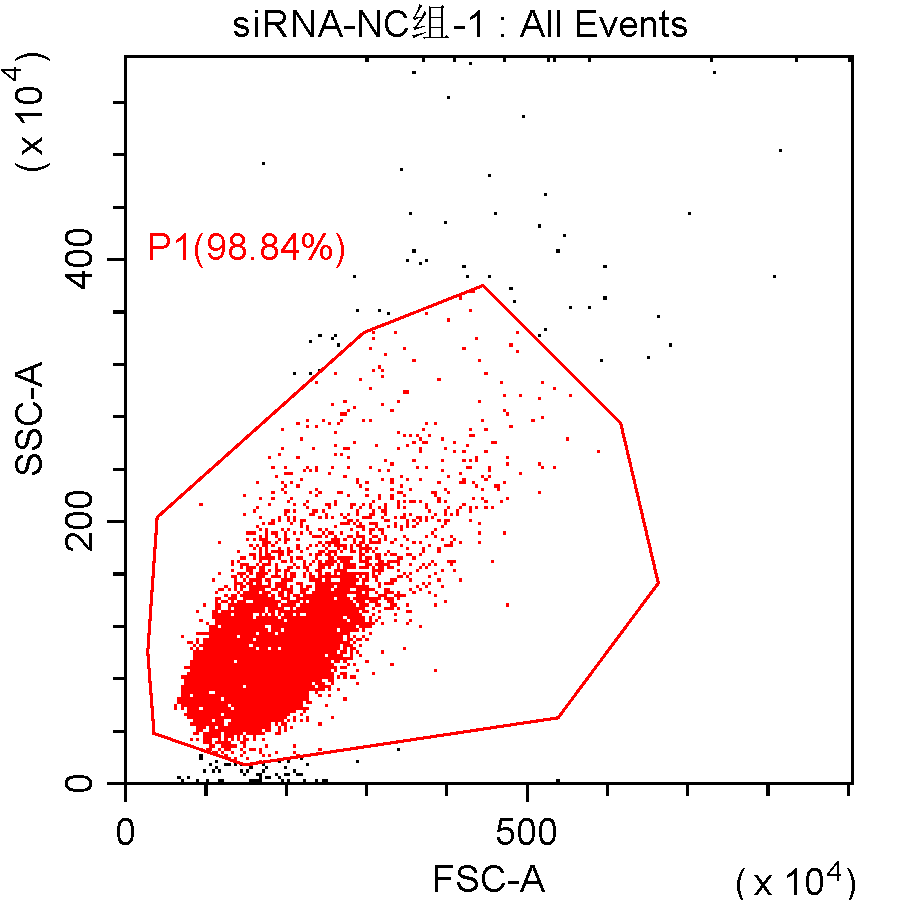

Supplement: Supplemental Information 11 [file peerj-09-11455-s011.zip › fig5A-Apoptosis/MG63/siRNA-NC组-1_Plot1.bmp]

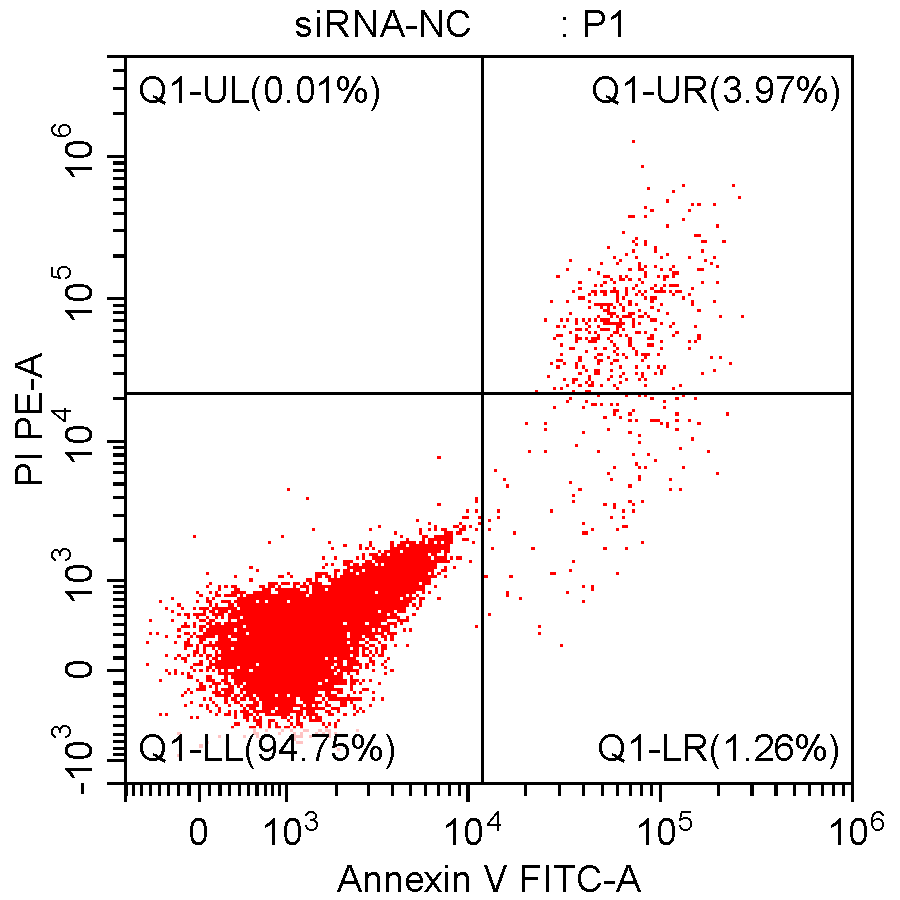

Supplement: Supplemental Information 11 [file peerj-09-11455-s011.zip › fig5A-Apoptosis/MG63/siRNA-NC组-1_Plot2.bmp]

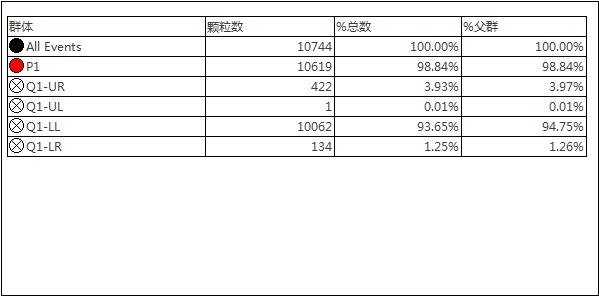

Supplement: Supplemental Information 11 [file peerj-09-11455-s011.zip › fig5A-Apoptosis/MG63/siRNA-NC组-1_Statistics1.bmp]

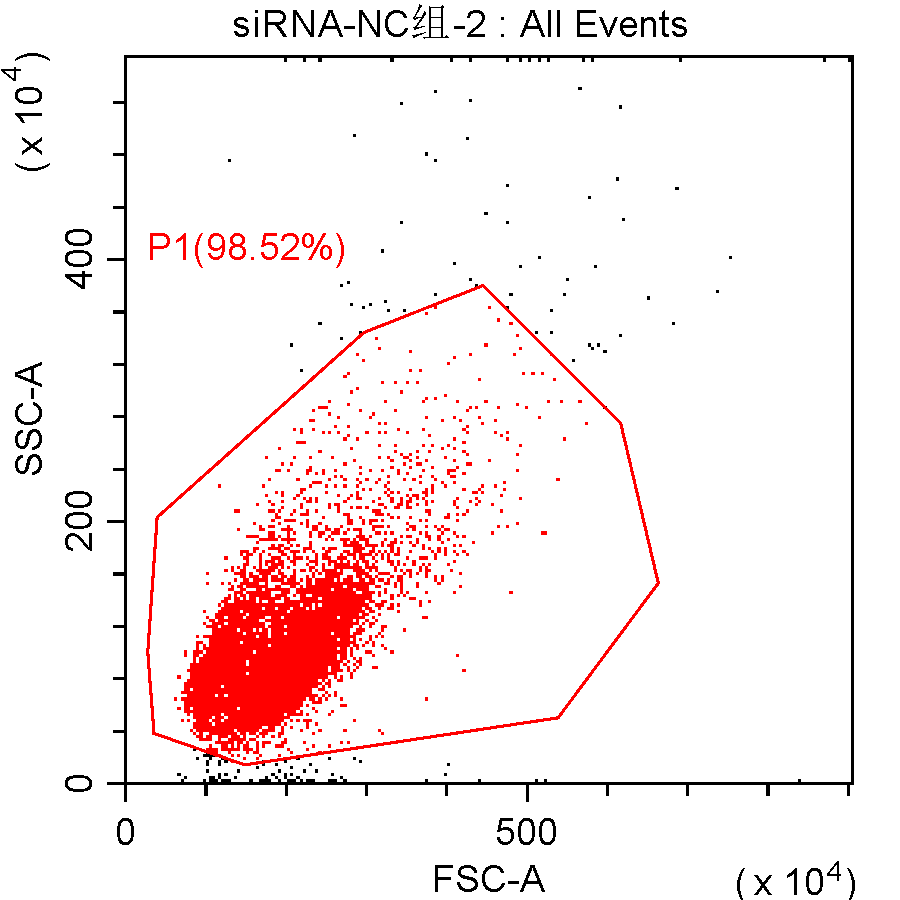

Supplement: Supplemental Information 11 [file peerj-09-11455-s011.zip › fig5A-Apoptosis/MG63/siRNA-NC组-2_Plot1.bmp]

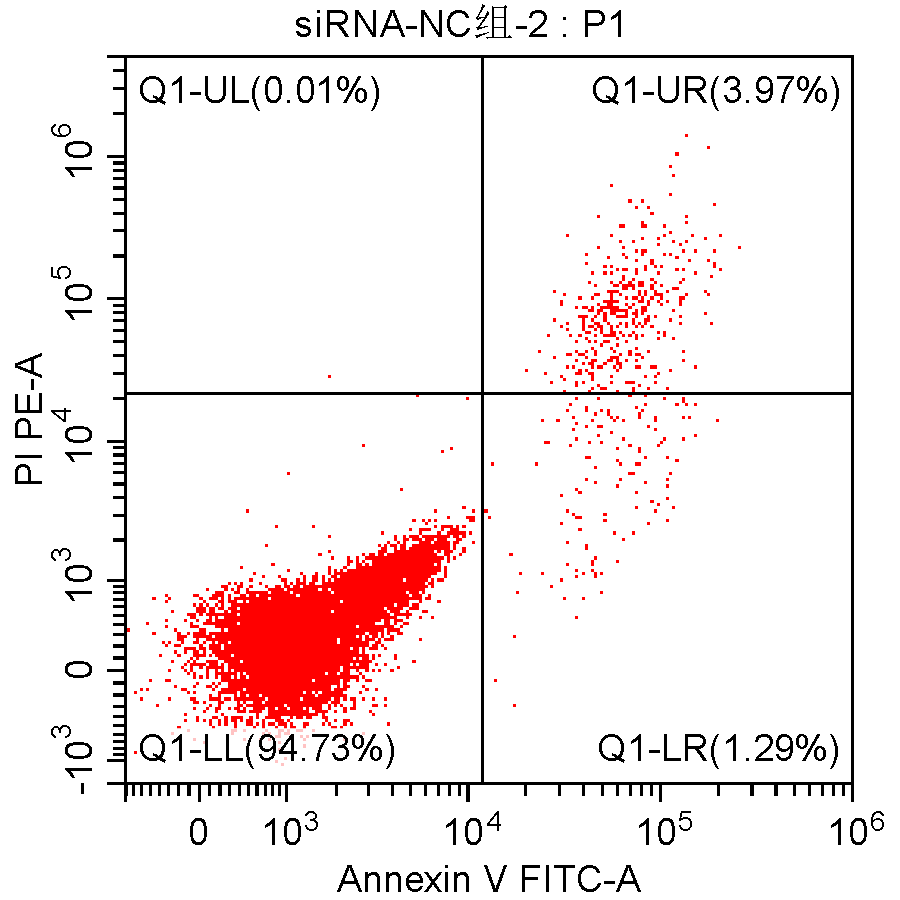

Supplement: Supplemental Information 11 [file peerj-09-11455-s011.zip › fig5A-Apoptosis/MG63/siRNA-NC组-2_Plot2.bmp]

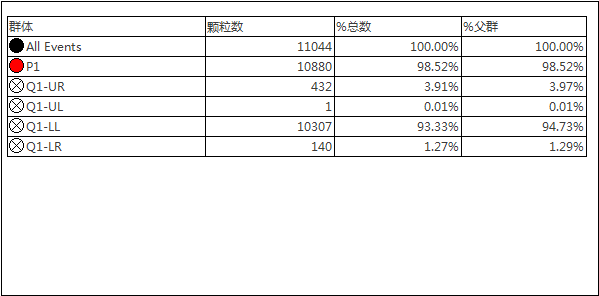

Supplement: Supplemental Information 11 [file peerj-09-11455-s011.zip › fig5A-Apoptosis/MG63/siRNA-NC组-2_Statistics1.bmp]

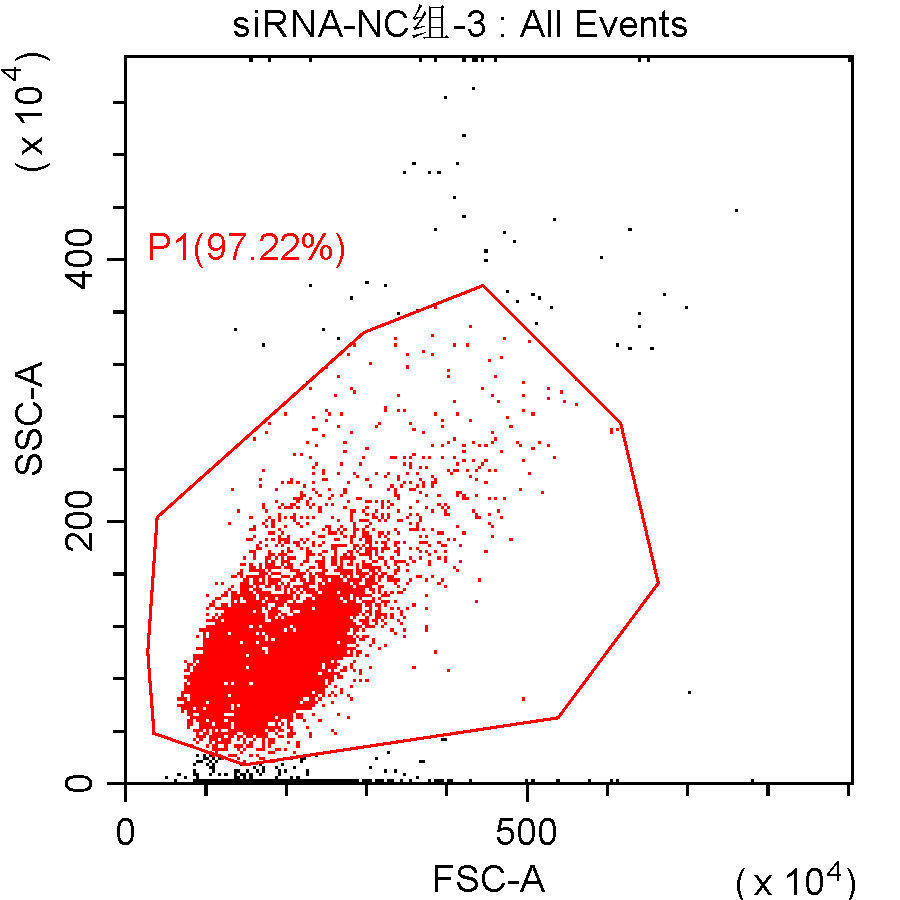

Supplement: Supplemental Information 11 [file peerj-09-11455-s011.zip › fig5A-Apoptosis/MG63/siRNA-NC组-3_Plot1.bmp]

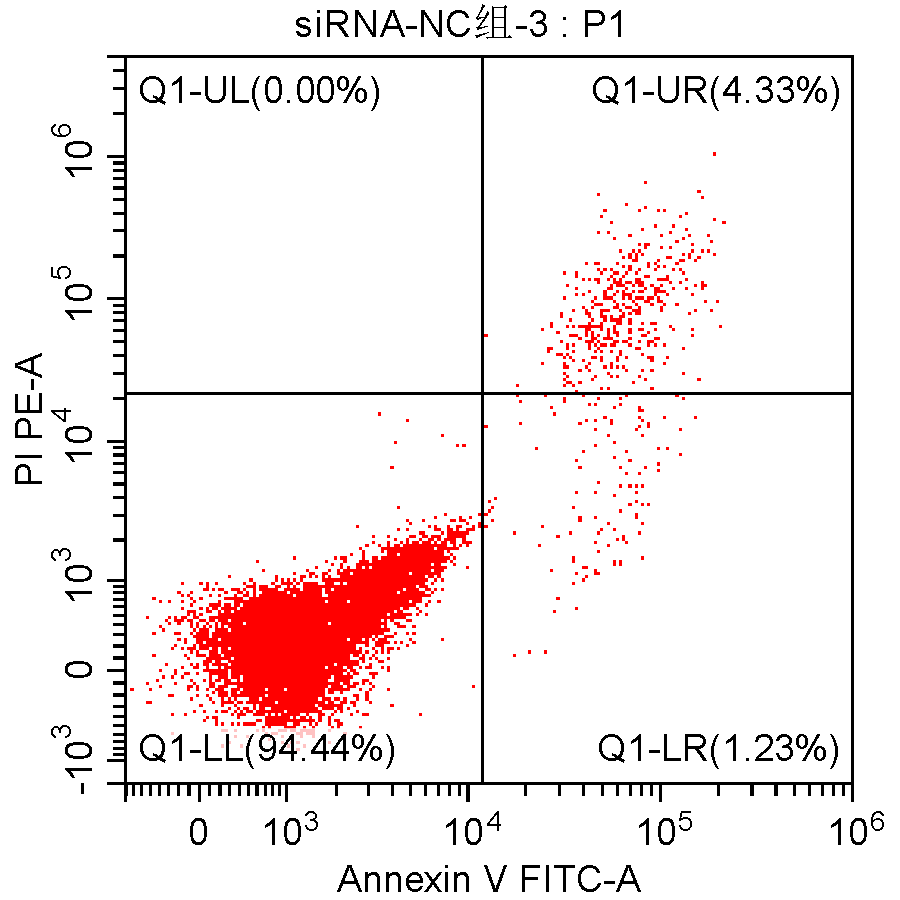

Supplement: Supplemental Information 11 [file peerj-09-11455-s011.zip › fig5A-Apoptosis/MG63/siRNA-NC组-3_Plot2.bmp]

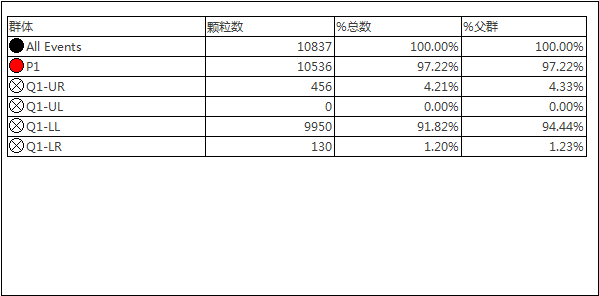

Supplement: Supplemental Information 11 [file peerj-09-11455-s011.zip › fig5A-Apoptosis/MG63/siRNA-NC组-3_Statistics1.bmp]

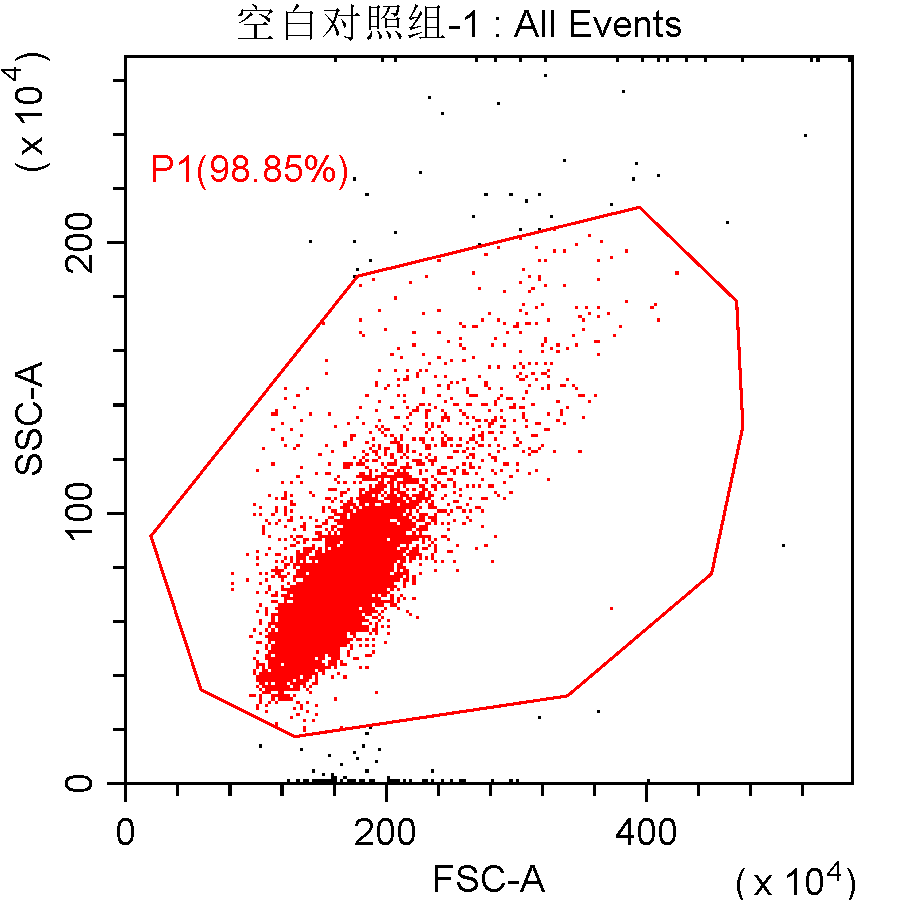

Supplement: Supplemental Information 11 [file peerj-09-11455-s011.zip › fig5A-Apoptosis/U20S/Control-1_Plot1.bmp]

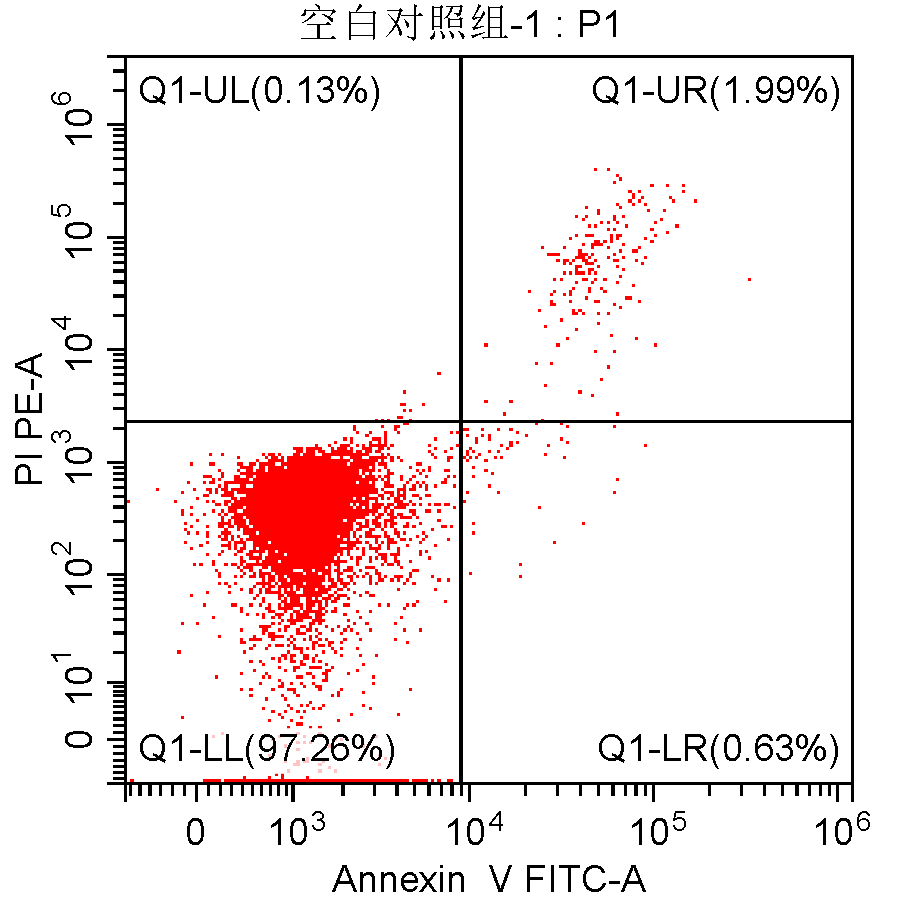

Supplement: Supplemental Information 11 [file peerj-09-11455-s011.zip › fig5A-Apoptosis/U20S/Control-1_Plot2.bmp]

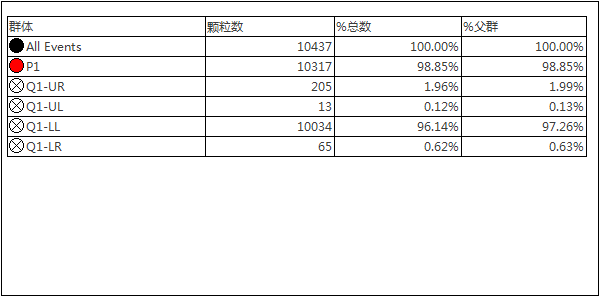

Supplement: Supplemental Information 11 [file peerj-09-11455-s011.zip › fig5A-Apoptosis/U20S/Control-1_Statistics1.bmp]

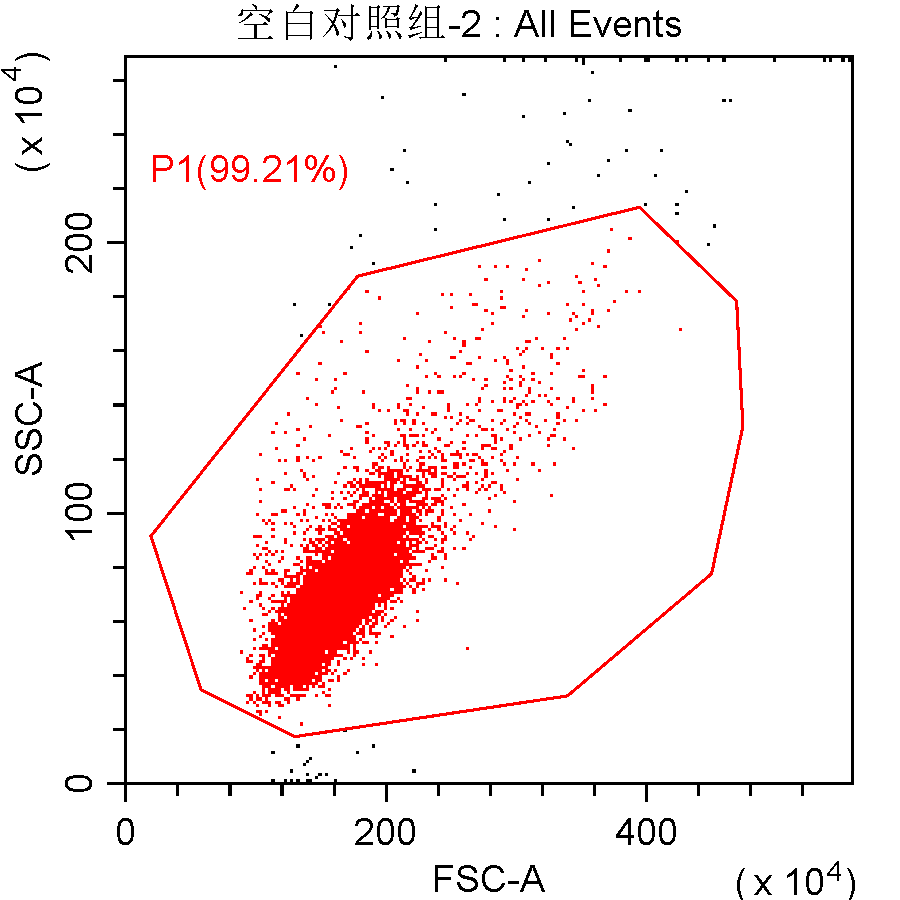

Supplement: Supplemental Information 11 [file peerj-09-11455-s011.zip › fig5A-Apoptosis/U20S/Control-2_Plot1.bmp]

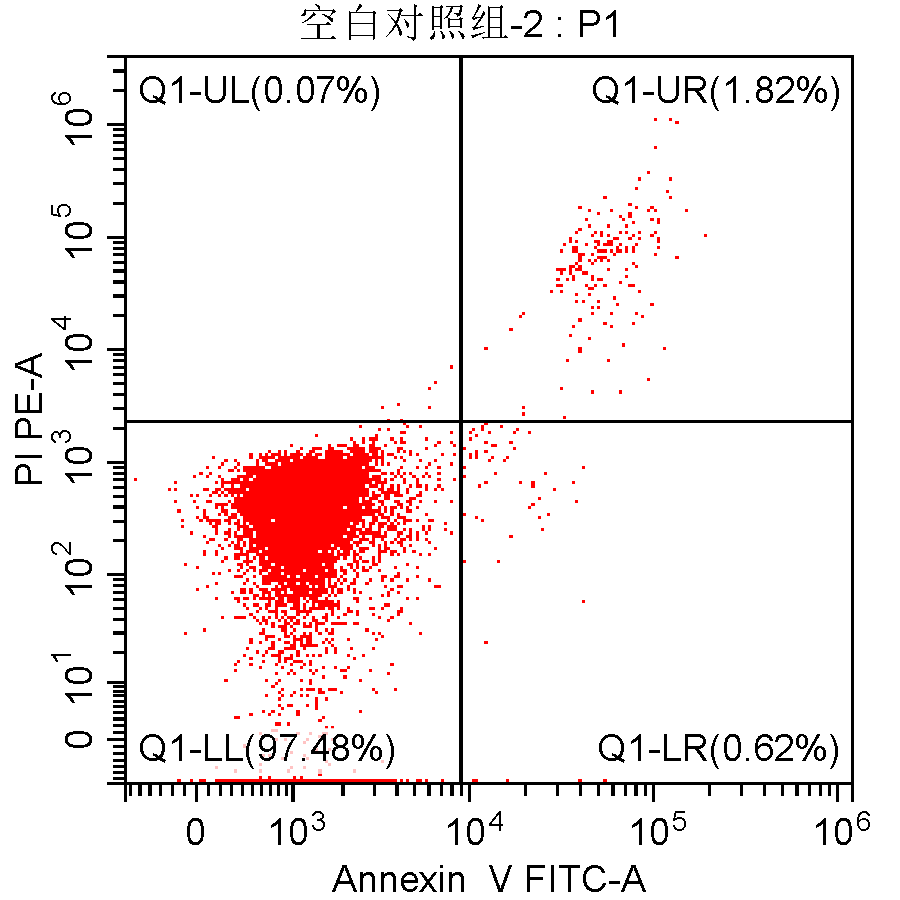

Supplement: Supplemental Information 11 [file peerj-09-11455-s011.zip › fig5A-Apoptosis/U20S/Control-2_Plot2.bmp]

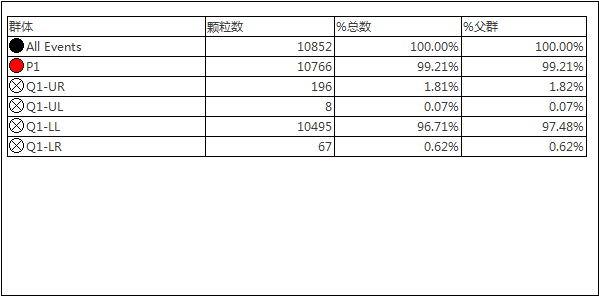

Supplement: Supplemental Information 11 [file peerj-09-11455-s011.zip › fig5A-Apoptosis/U20S/Control-2_Statistics1.bmp]

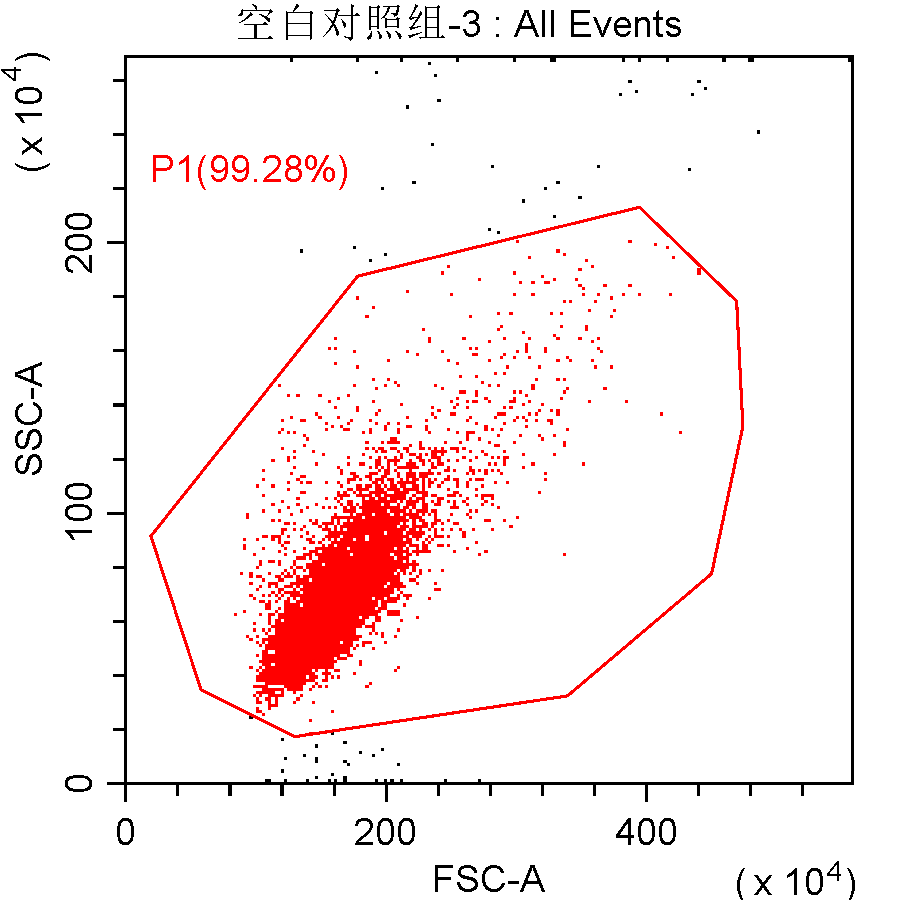

Supplement: Supplemental Information 11 [file peerj-09-11455-s011.zip › fig5A-Apoptosis/U20S/Control-3_Plot1.bmp]

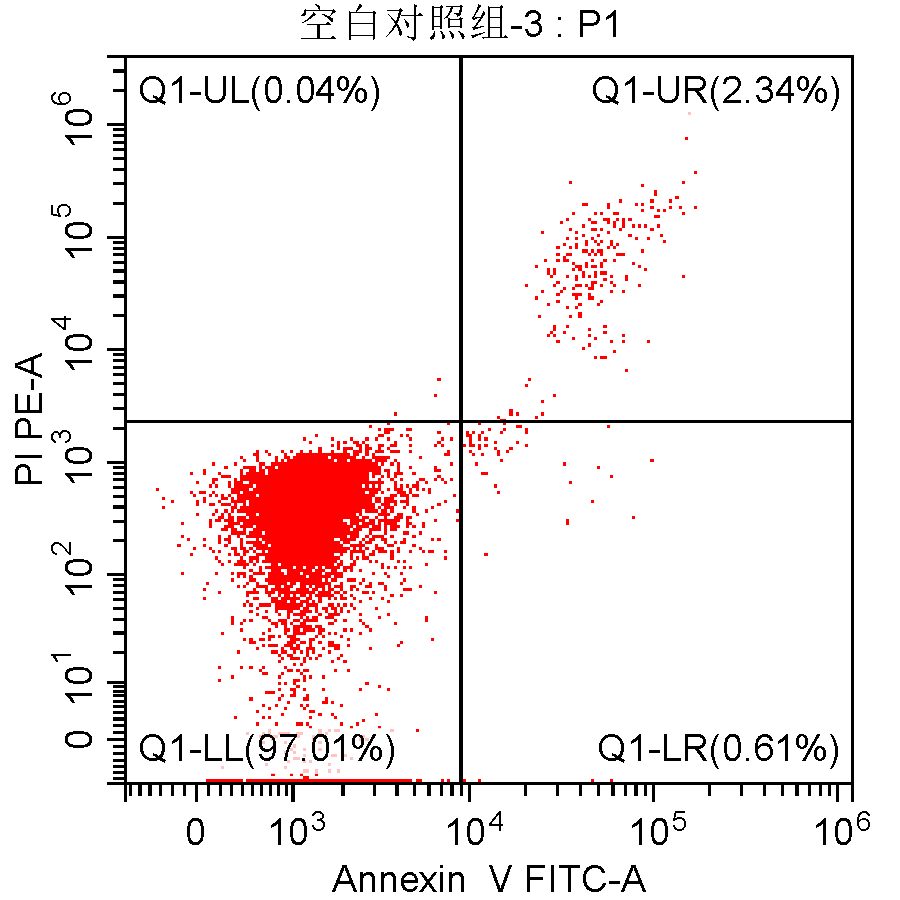

Supplement: Supplemental Information 11 [file peerj-09-11455-s011.zip › fig5A-Apoptosis/U20S/Control-3_Plot2.bmp]

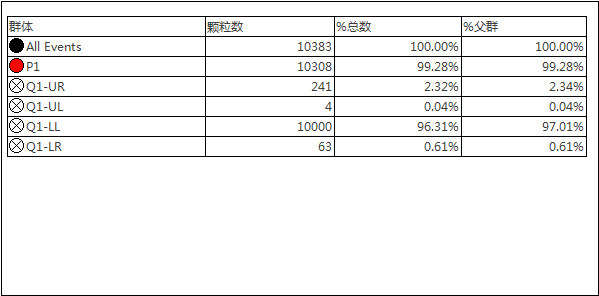

Supplement: Supplemental Information 11 [file peerj-09-11455-s011.zip › fig5A-Apoptosis/U20S/Control-3_Statistics1.bmp]

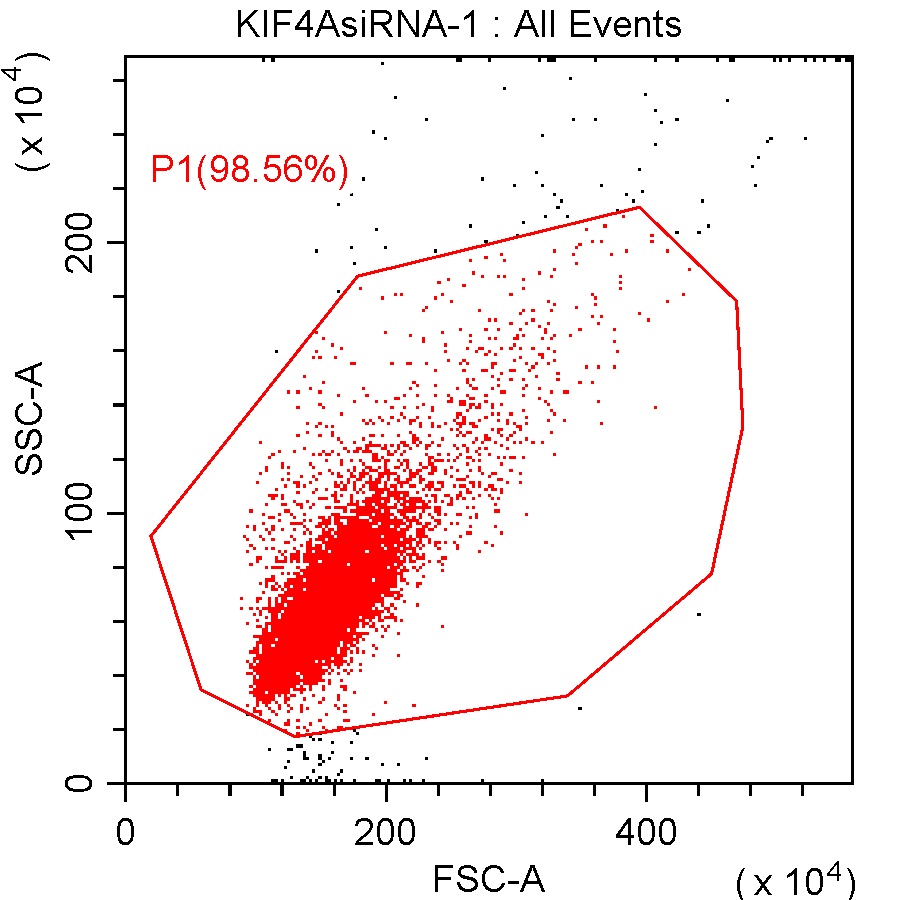

Supplement: Supplemental Information 11 [file peerj-09-11455-s011.zip › fig5A-Apoptosis/U20S/KIF4AsiRNA-1_Plot1.bmp]

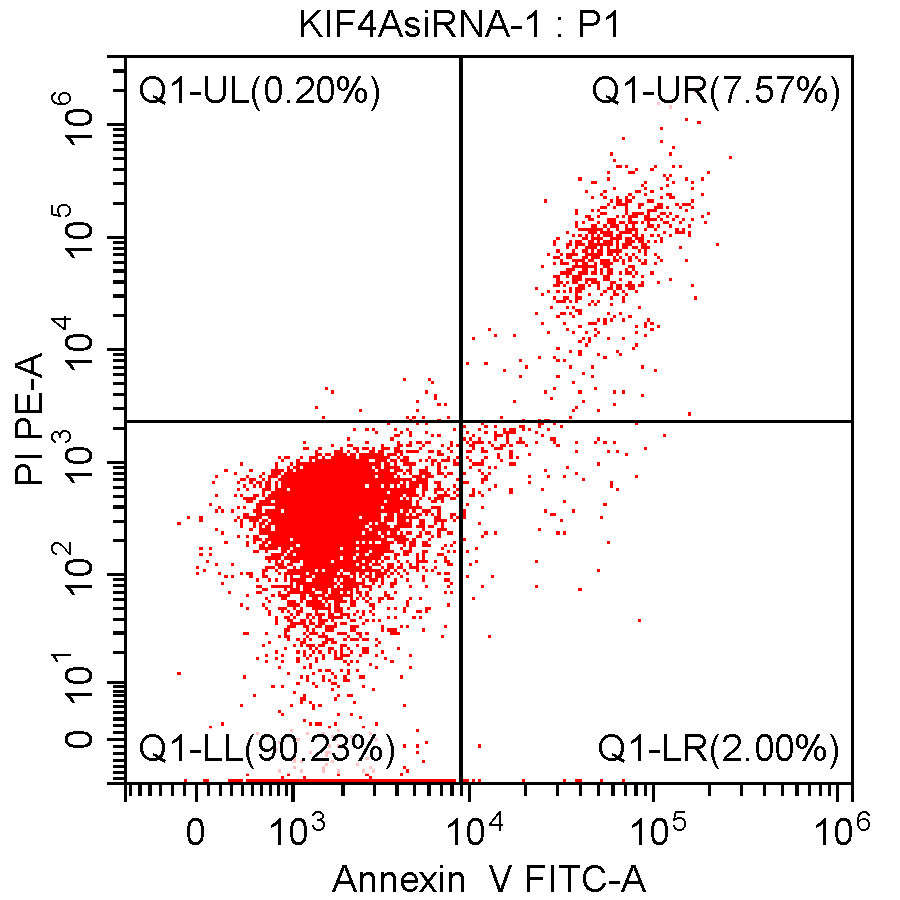

Supplement: Supplemental Information 11 [file peerj-09-11455-s011.zip › fig5A-Apoptosis/U20S/KIF4AsiRNA-1_Plot2.bmp]
