# Supplementary material for: Identification of KIF4A as a pan-cancer diagnostic and prognostic biomarker via bioinformatics analysis and validation in osteosarcoma cell lines
Source: PeerJ. 2021 May 21;9:e11455. doi: 10.7717/peerj.11455 (PMC8142929; doi:10.7717/peerj.11455)
Supplement: Supplemental Information 9 [file peerj-09-11455-s009.zip › transwell.pptx]

## Slide 1
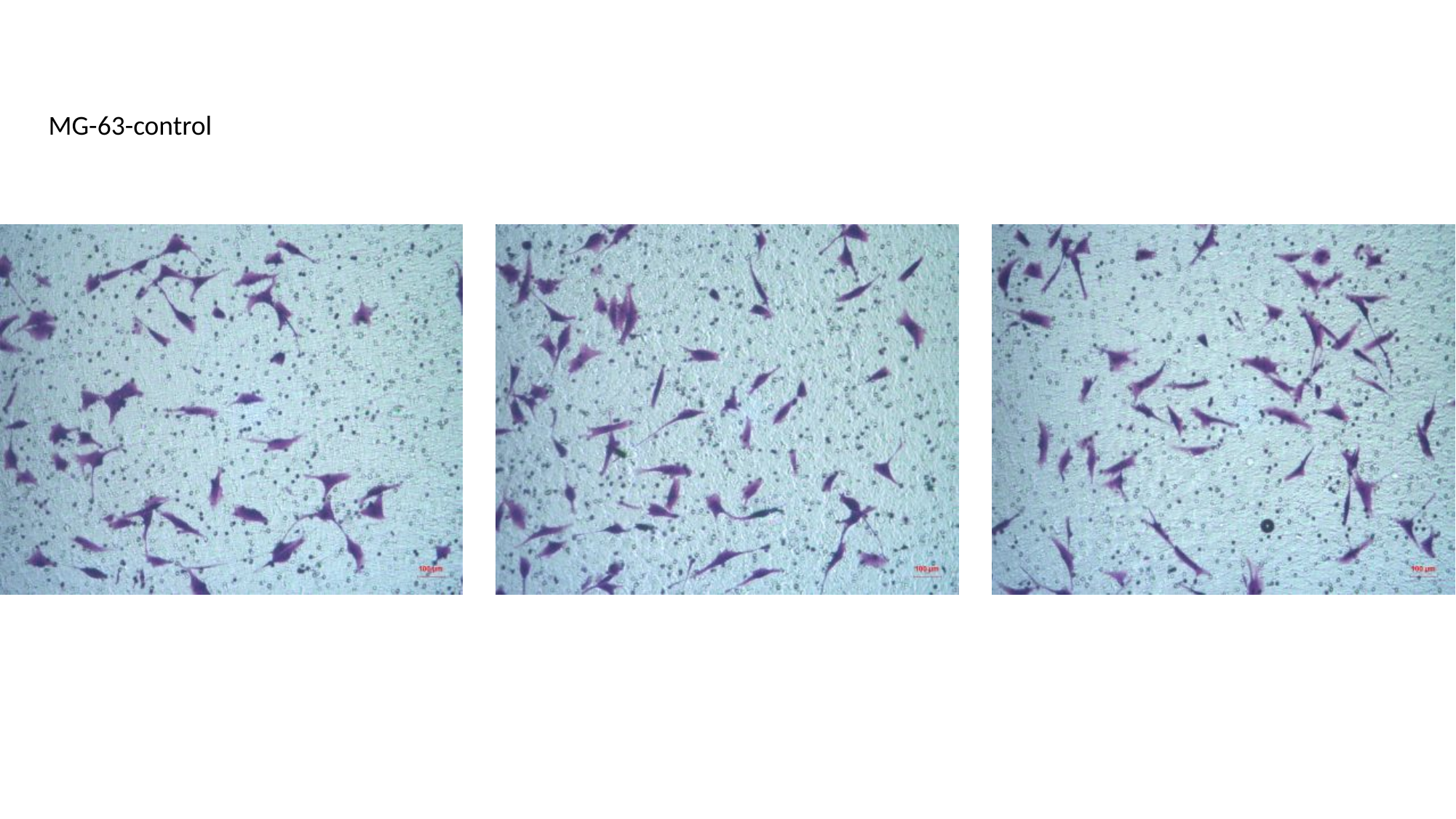

MG-63-control

## Slide 2
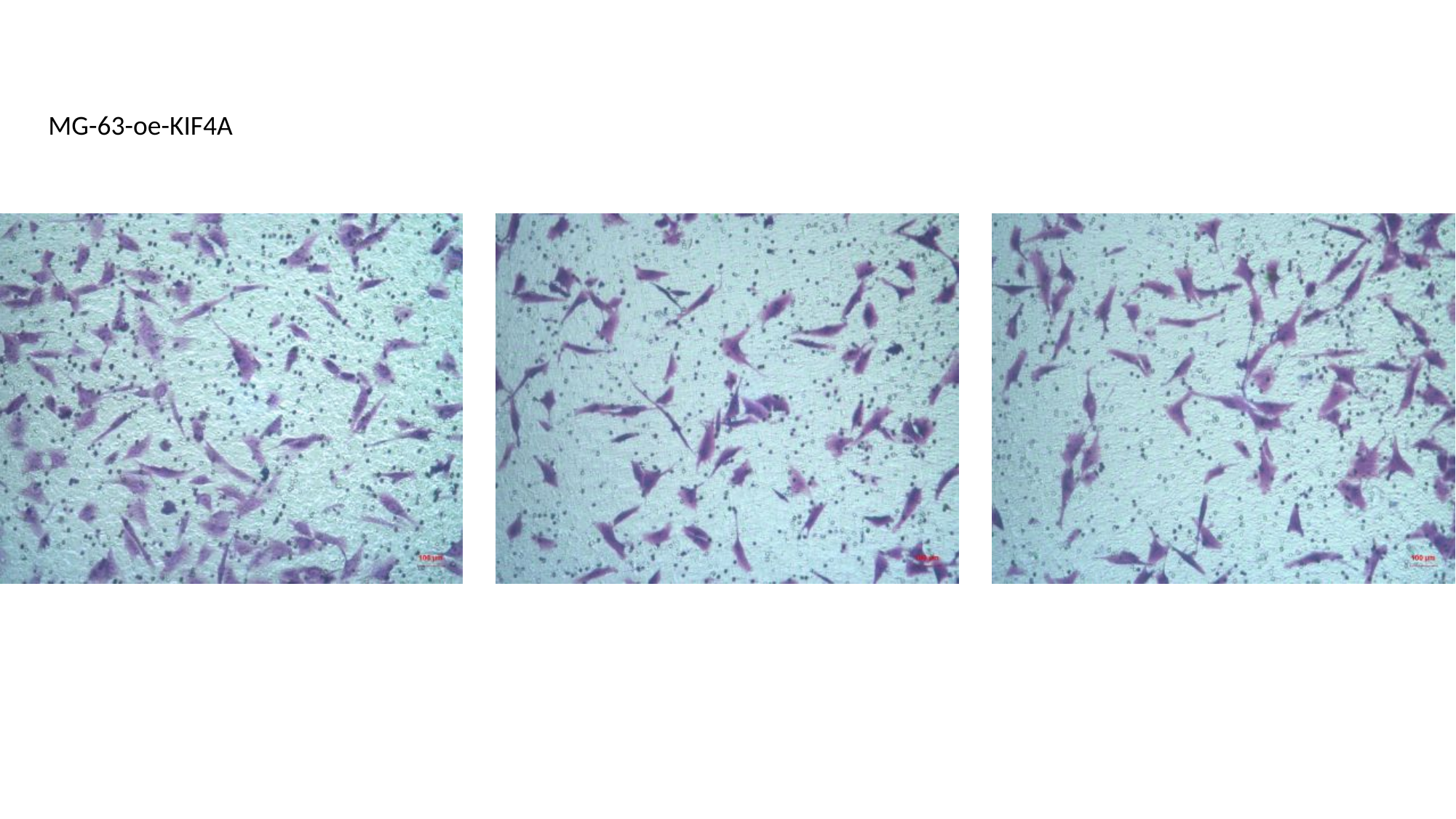

MG-63-oe-KIF4A

## Slide 3
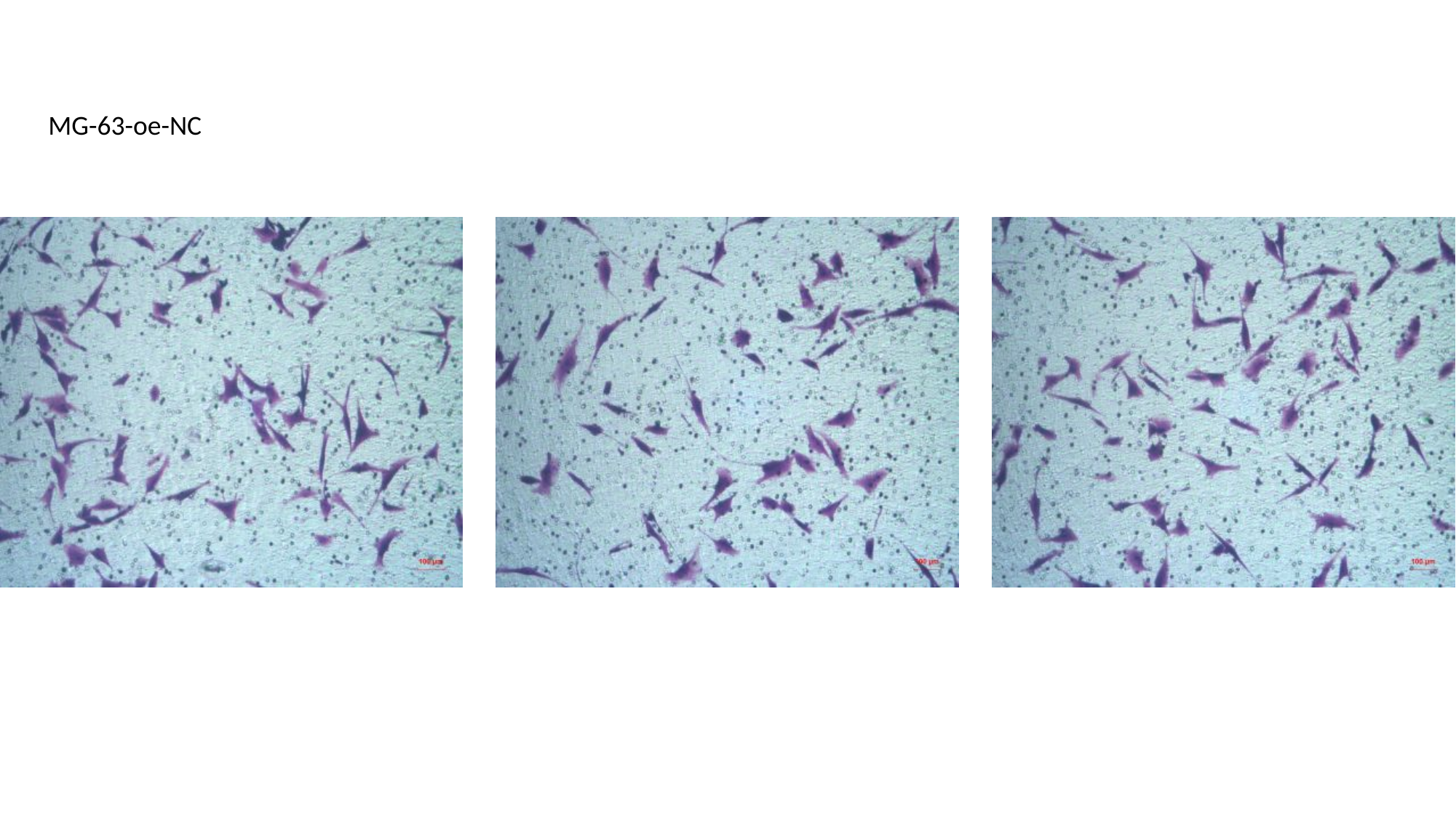

MG-63-oe-NC

## Slide 4
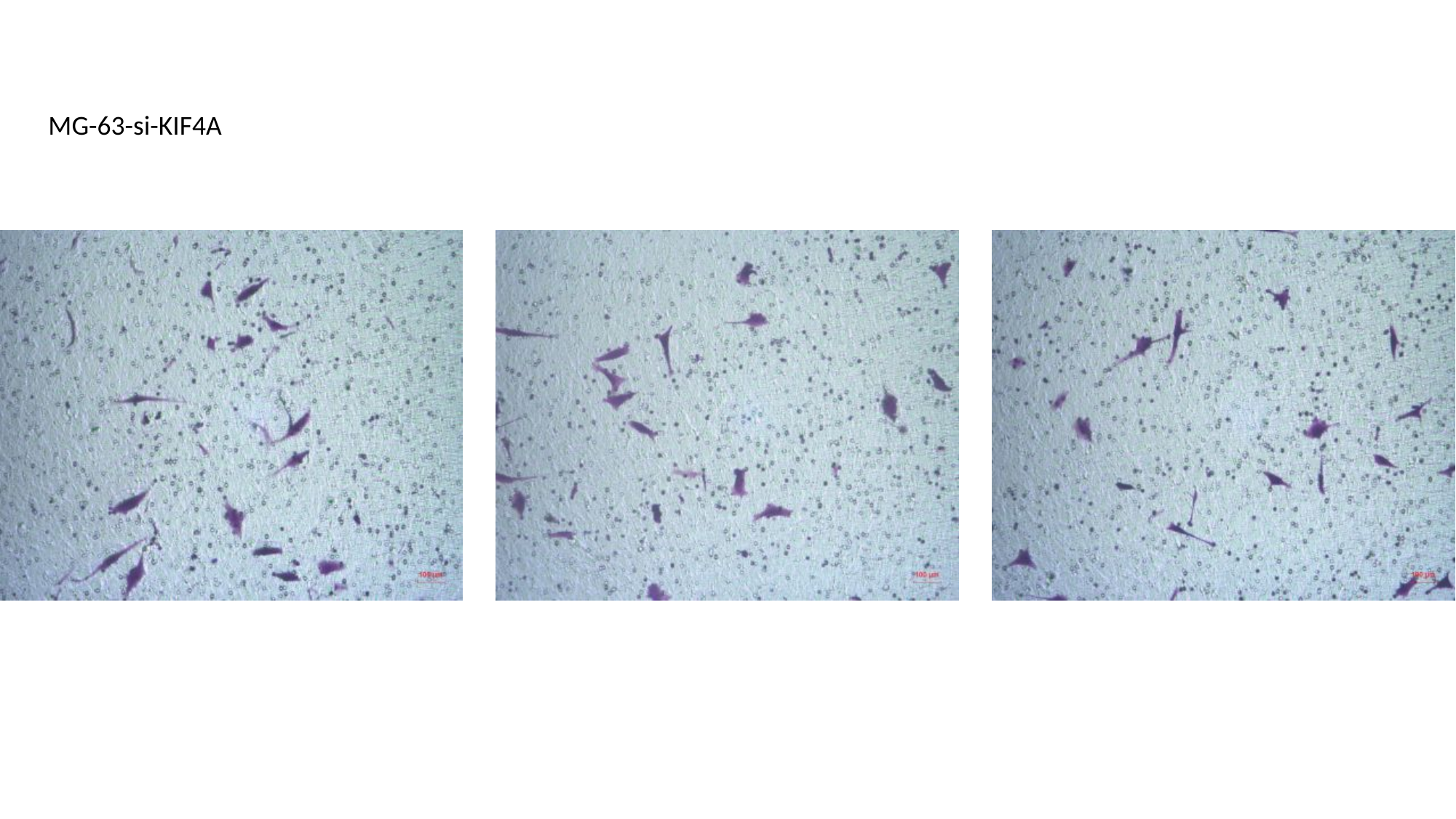

MG-63-si-KIF4A

## Slide 5
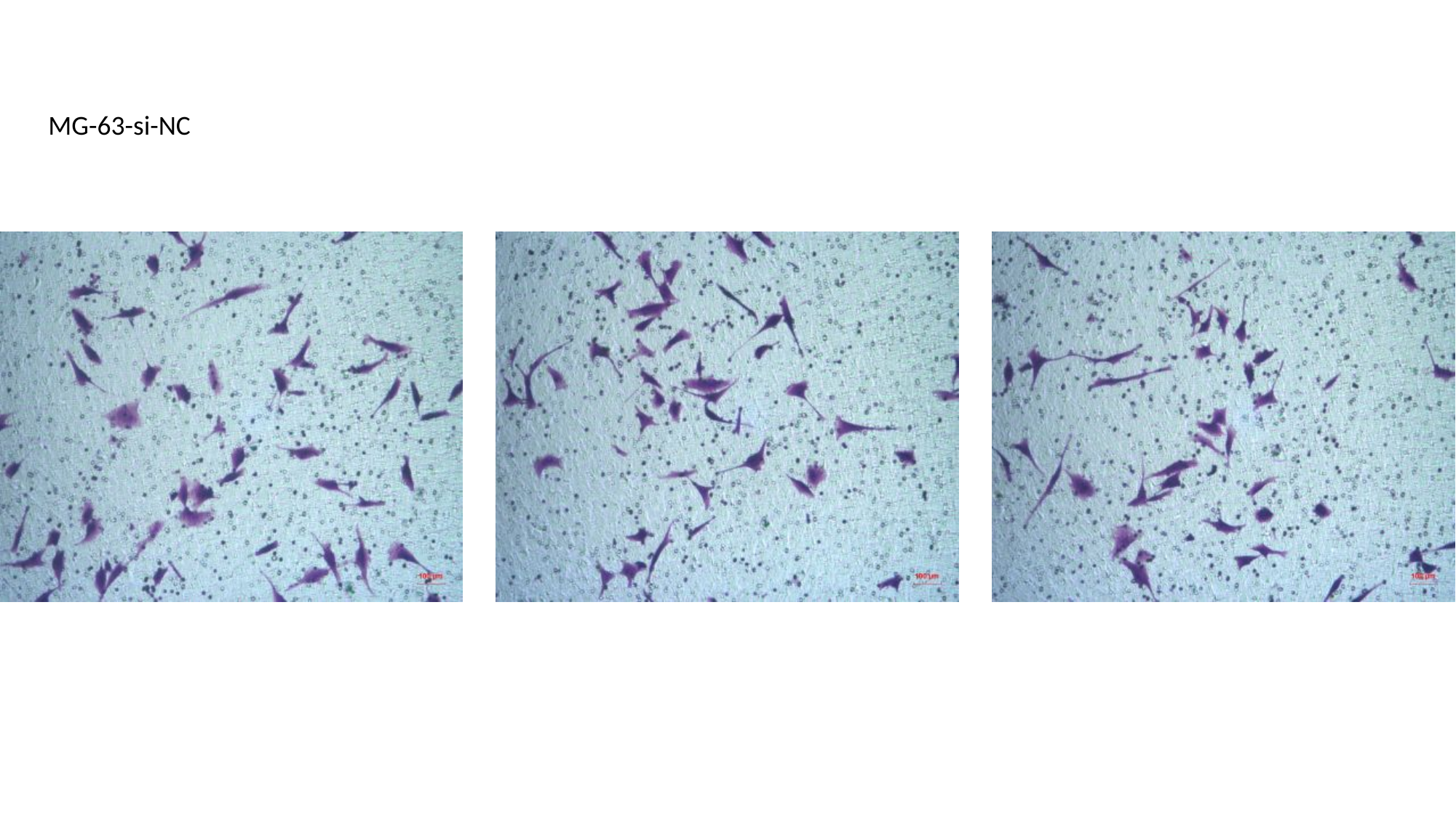

MG-63-si-NC

## Slide 6
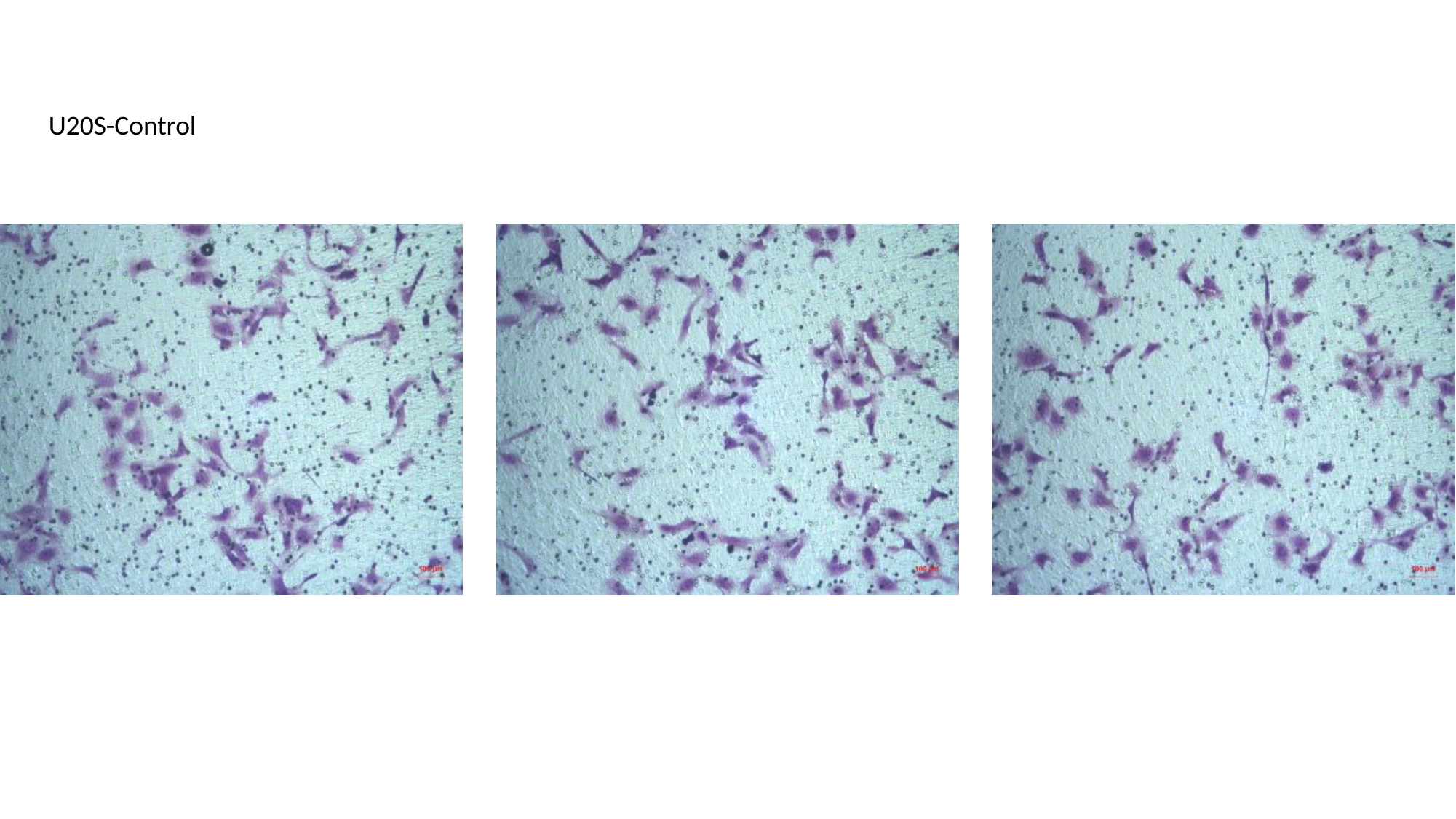

U20S-Control

## Slide 7
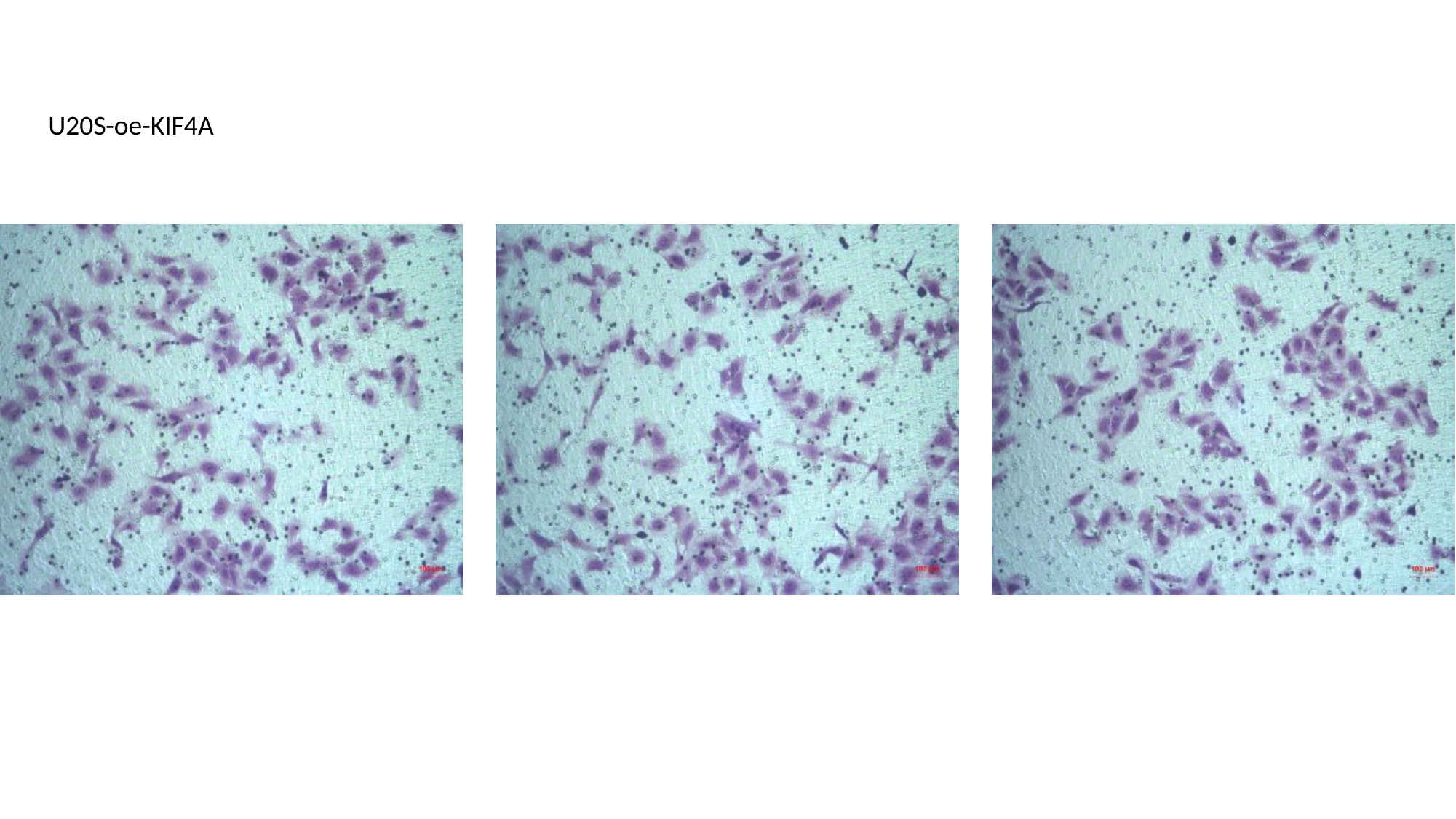

U20S-oe-KIF4A

## Slide 8
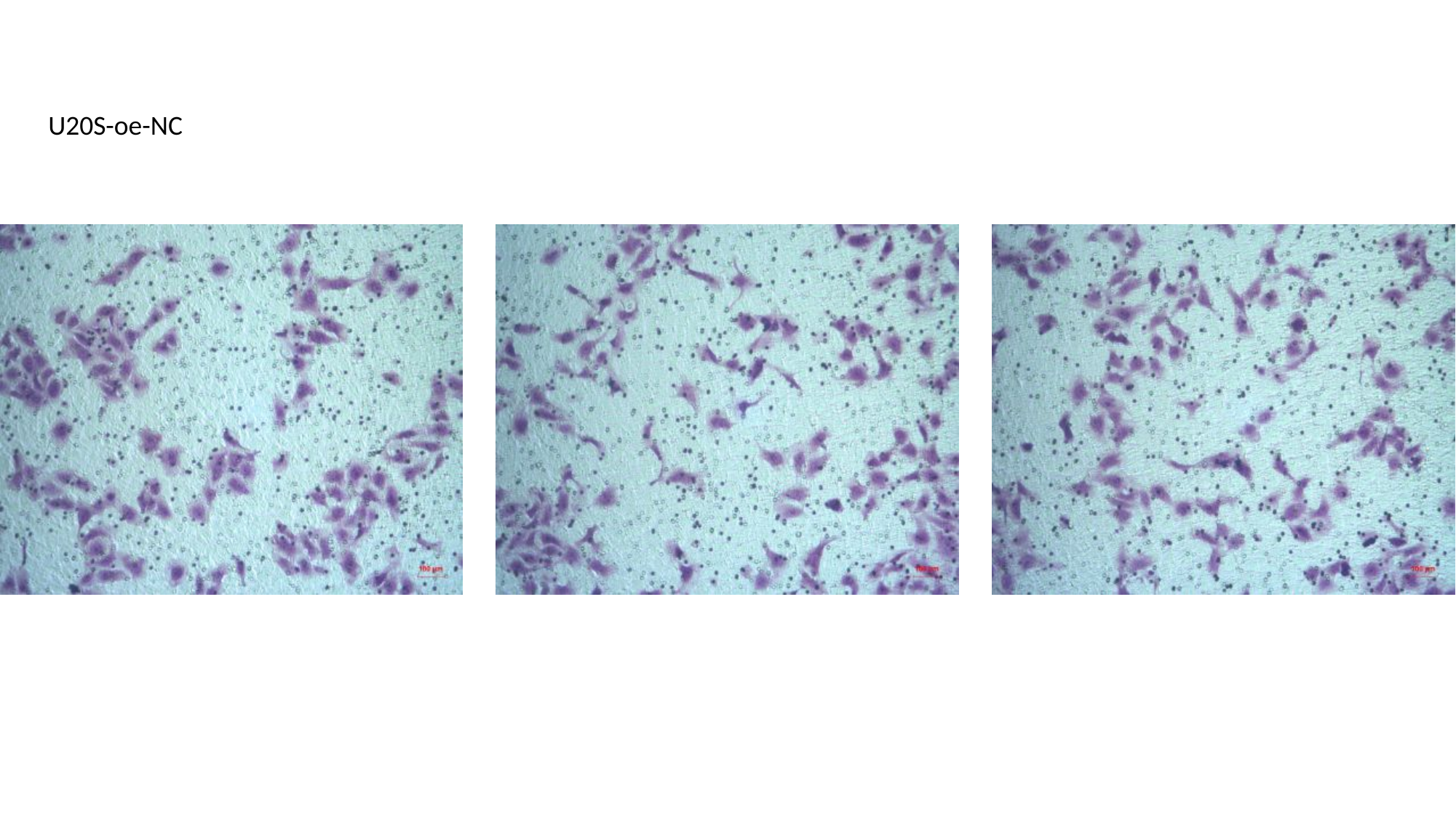

U20S-oe-NC

## Slide 9
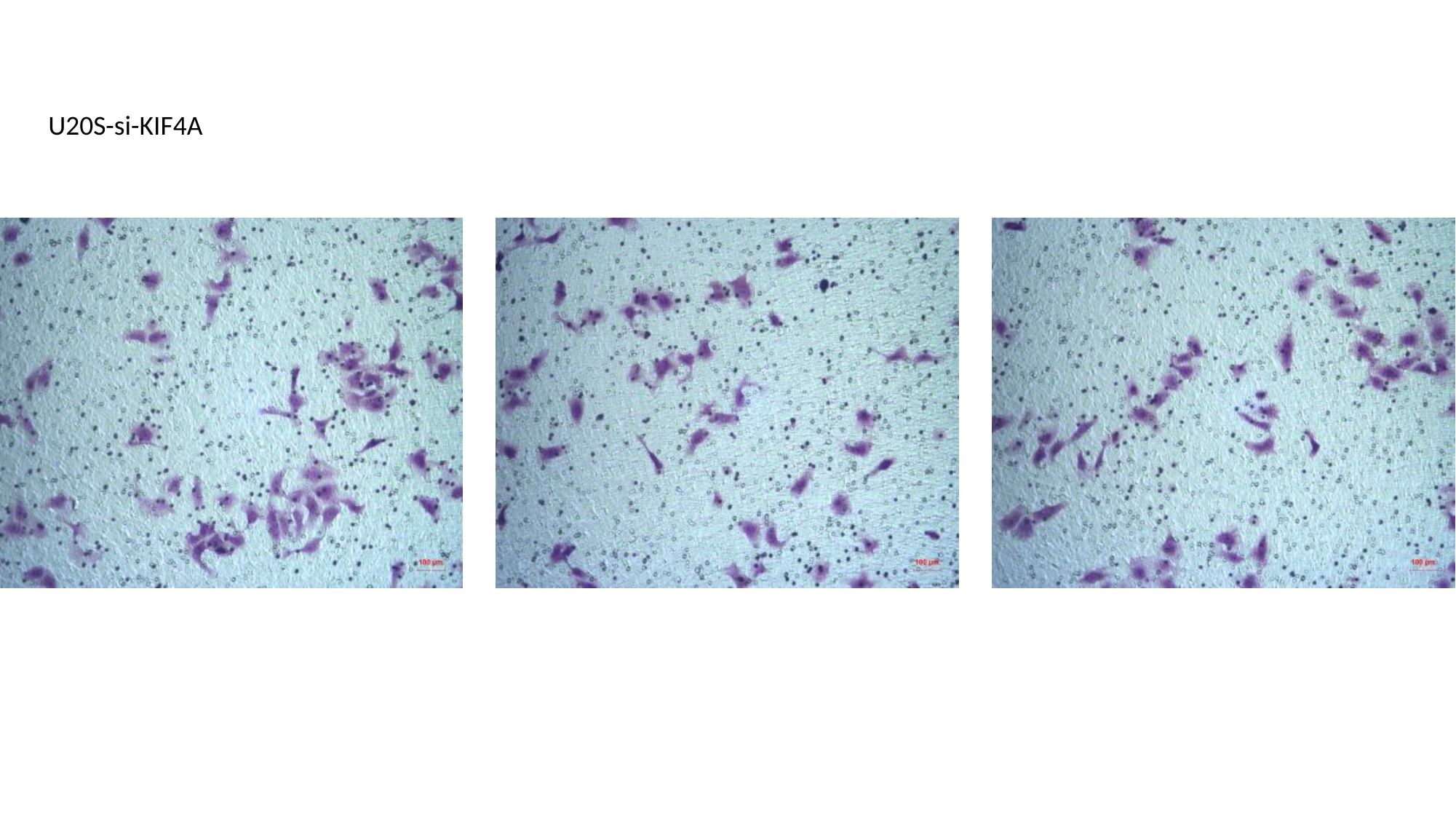

U20S-si-KIF4A

## Slide 10
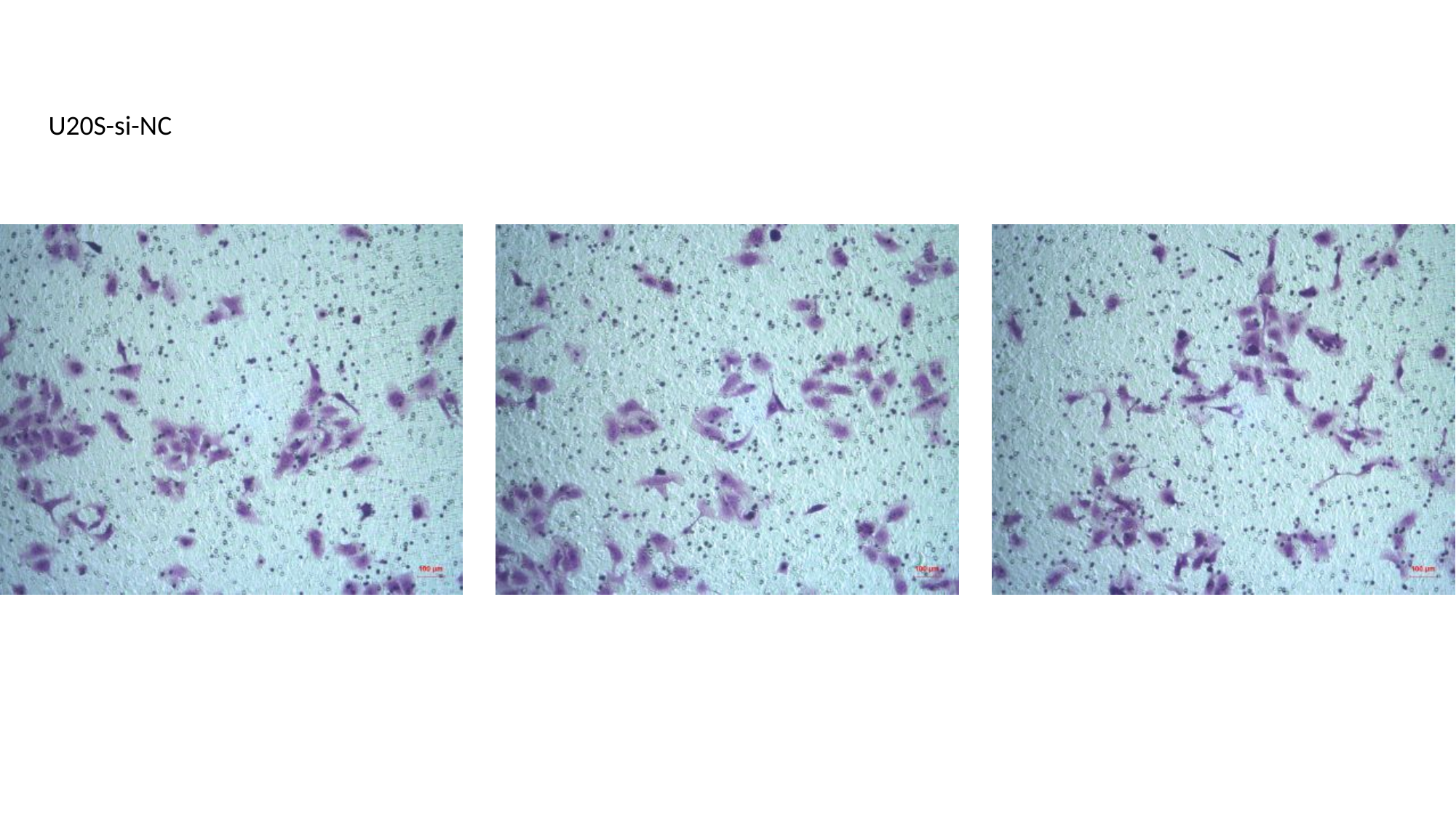

U20S-si-NC
