# Supplementary material for: Identification of KIF4A as a pan-cancer diagnostic and prognostic biomarker via bioinformatics analysis and validation in osteosarcoma cell lines
Source: PeerJ. 2021 May 21;9:e11455. doi: 10.7717/peerj.11455 (PMC8142929; doi:10.7717/peerj.11455)
Supplement: Supplemental Information 10 [file peerj-09-11455-s010.zip › fig4B-cell scratch test.pptx]

## Slide 1
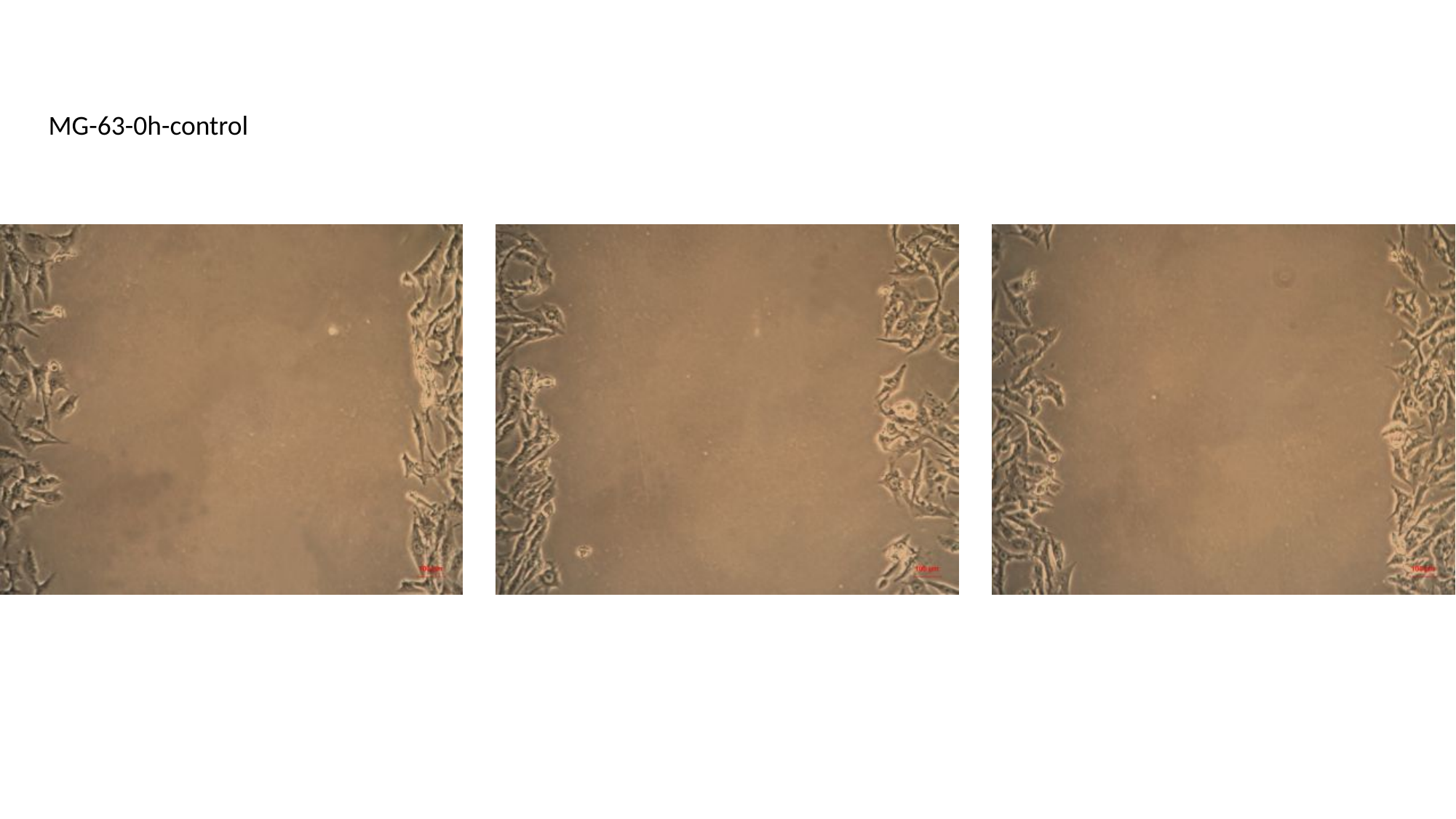

MG-63-0h-control

## Slide 2
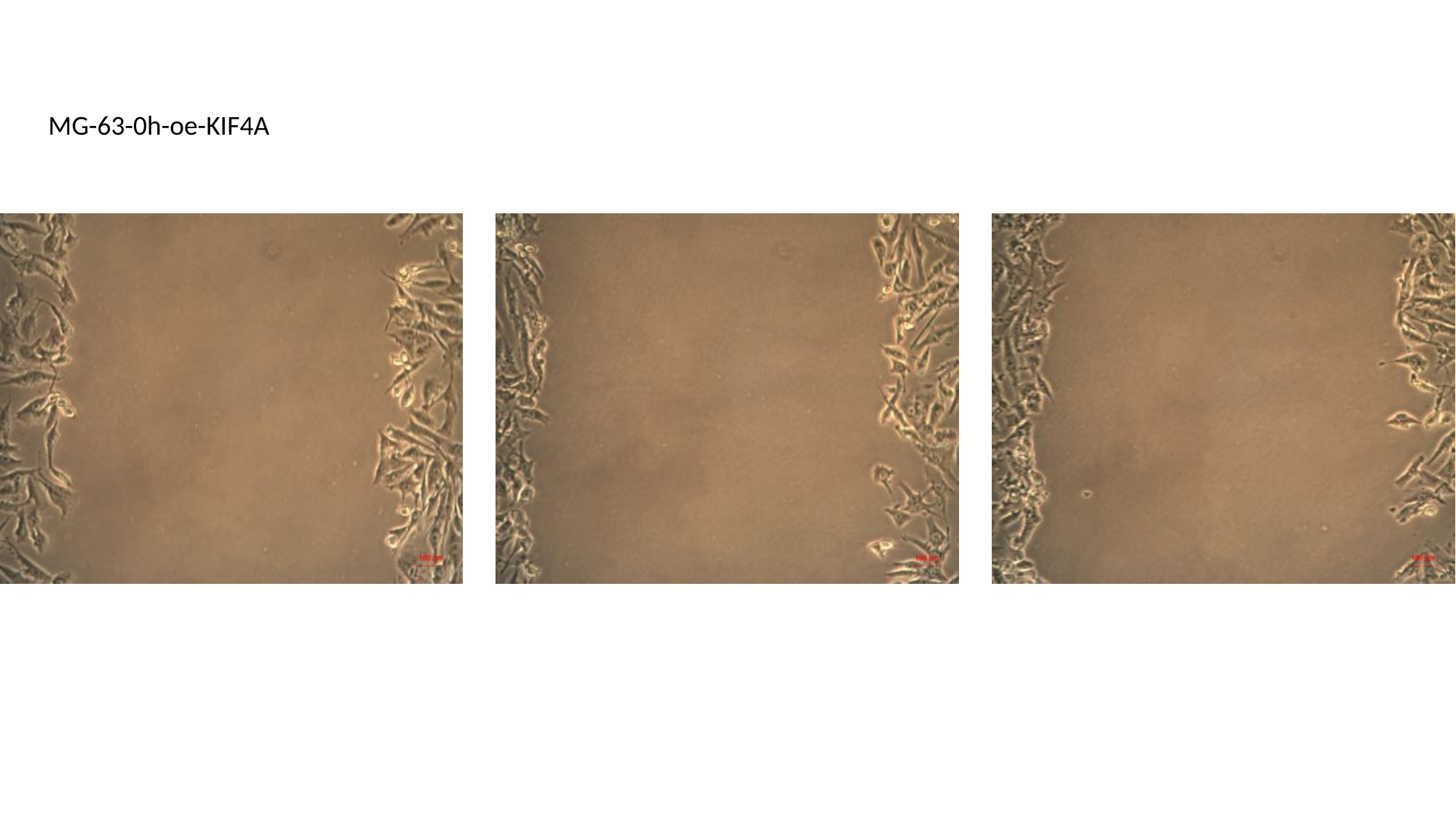

MG-63-0h-oe-KIF4A

## Slide 3
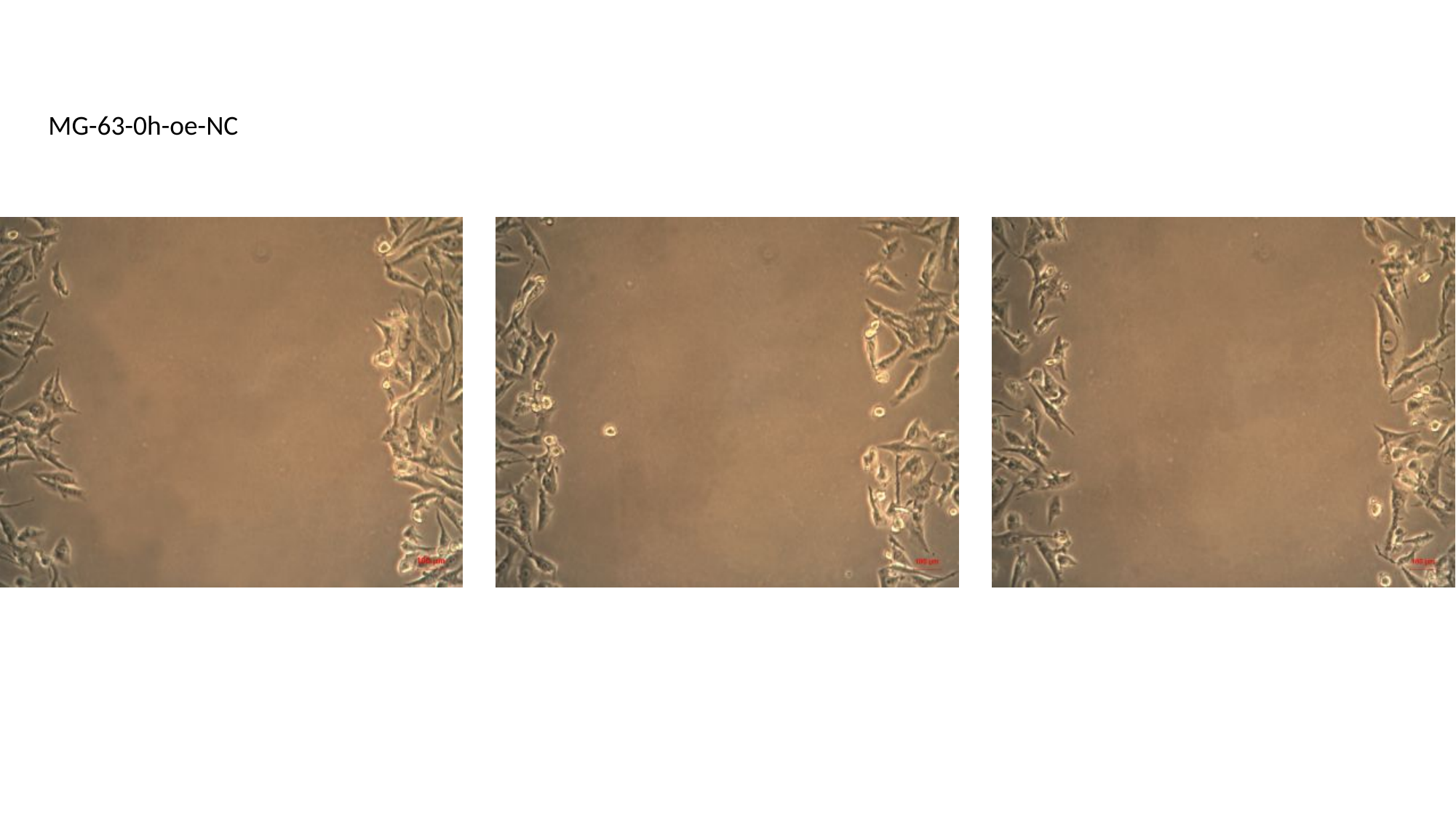

MG-63-0h-oe-NC

## Slide 4
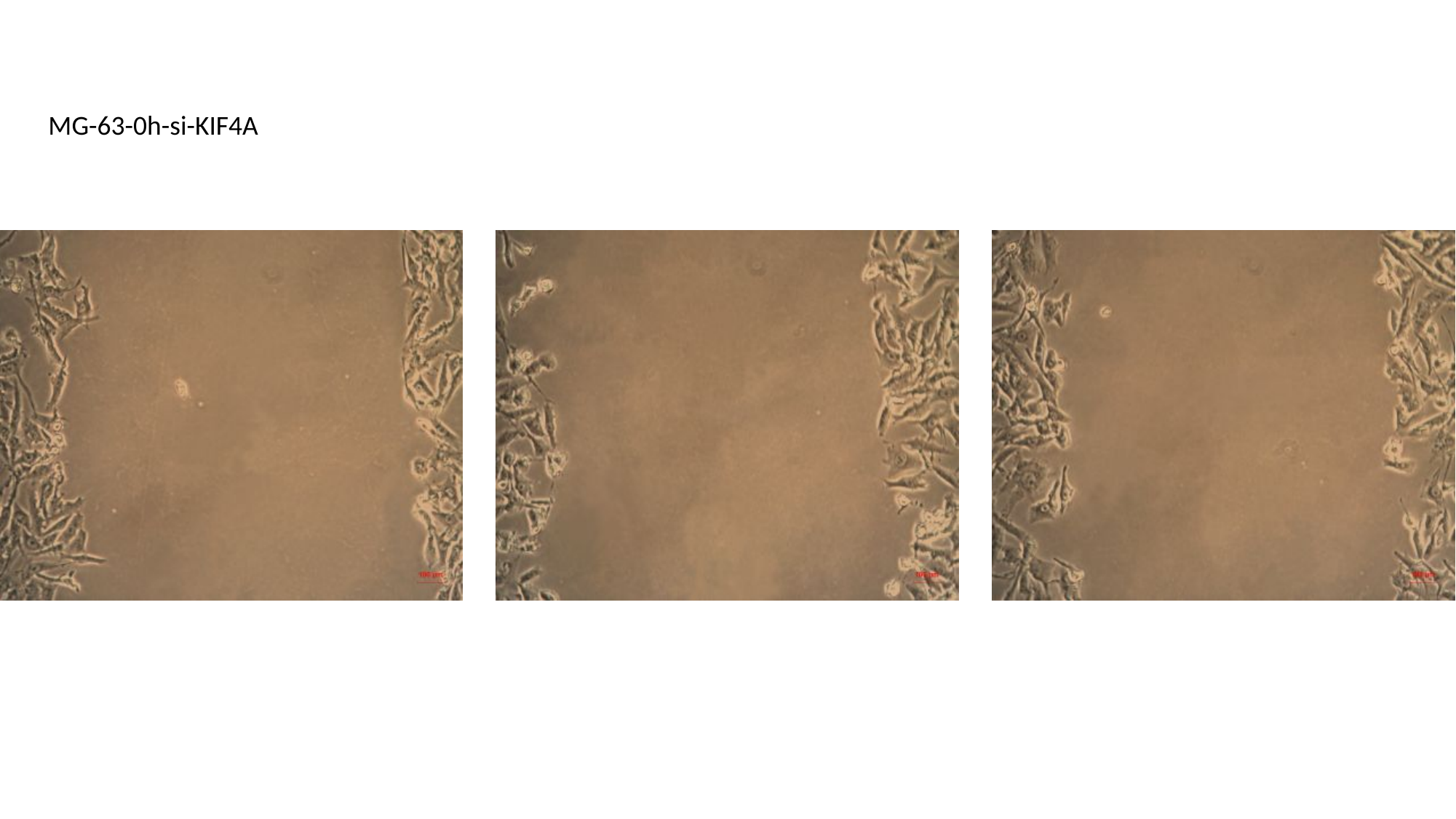

MG-63-0h-si-KIF4A

## Slide 5
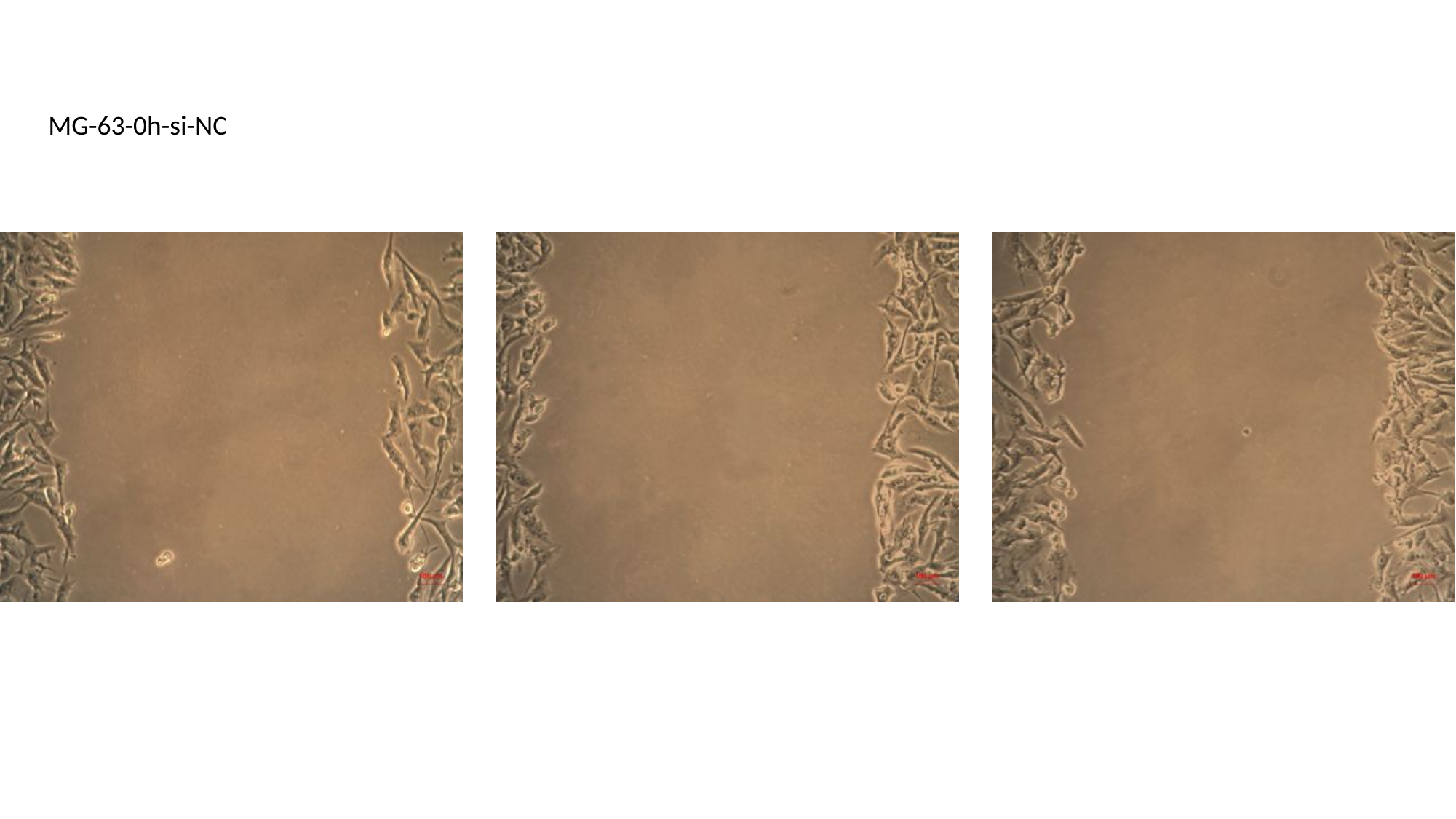

MG-63-0h-si-NC

## Slide 6
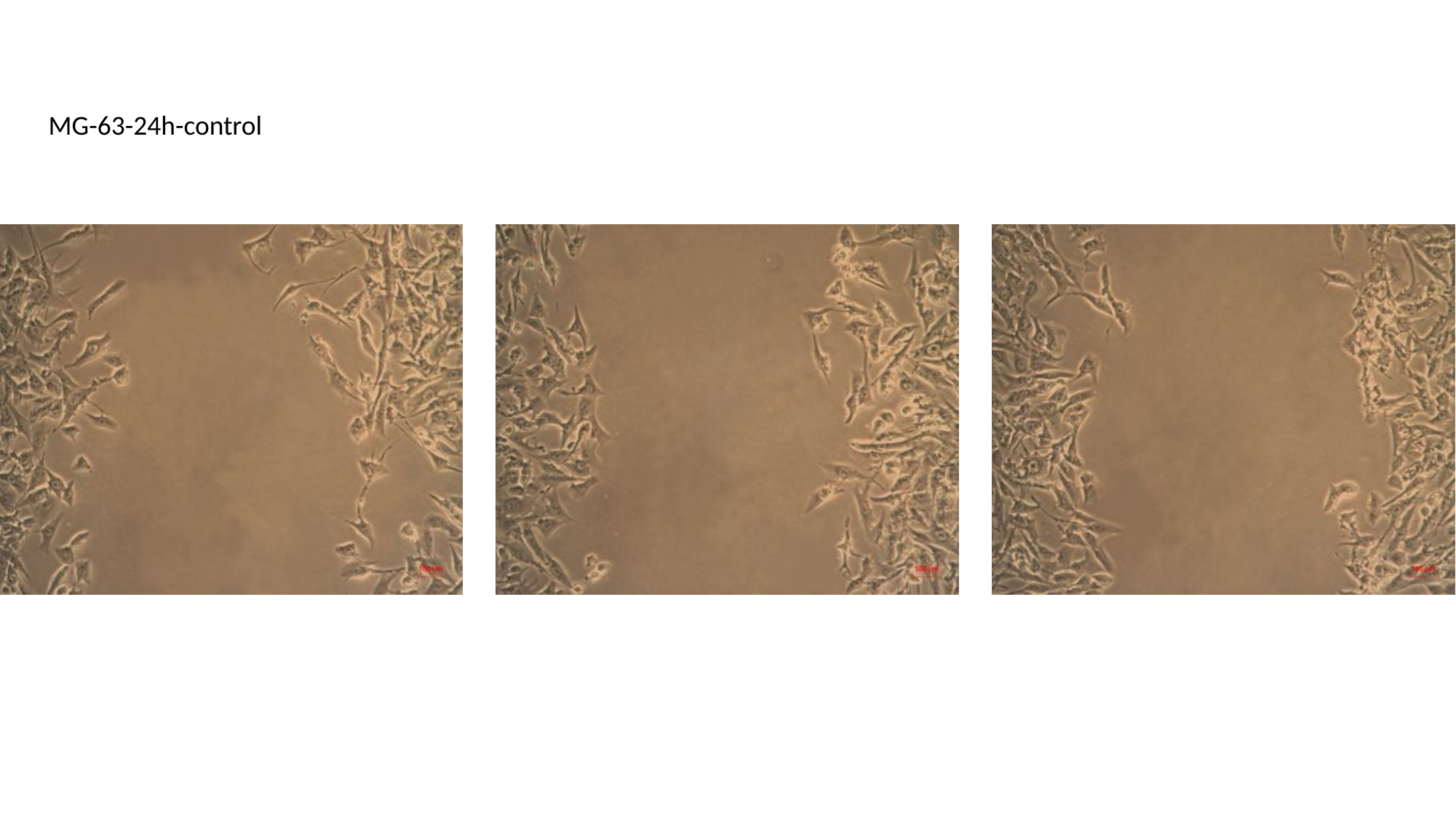

MG-63-24h-control

## Slide 7
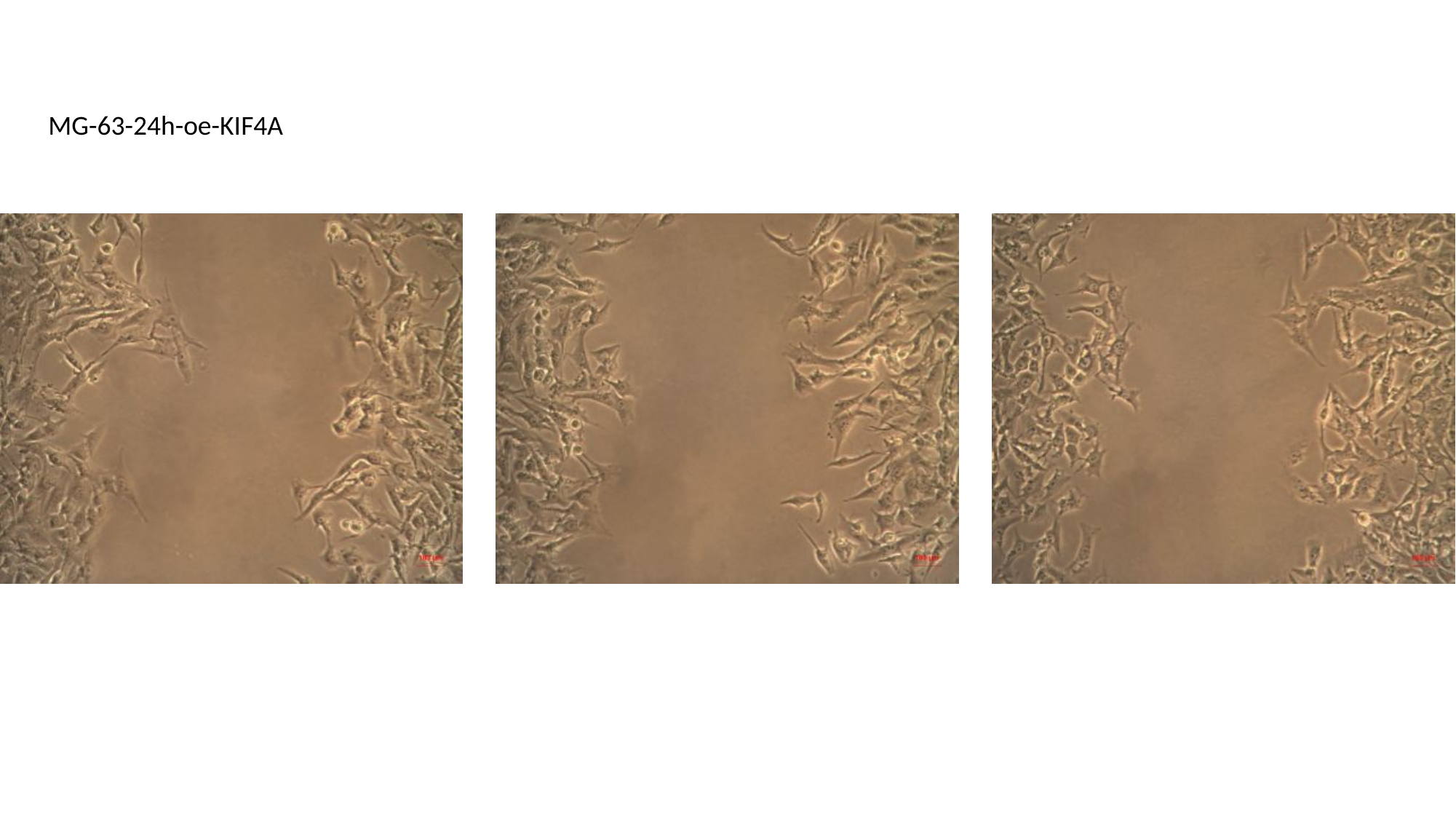

MG-63-24h-oe-KIF4A

## Slide 8
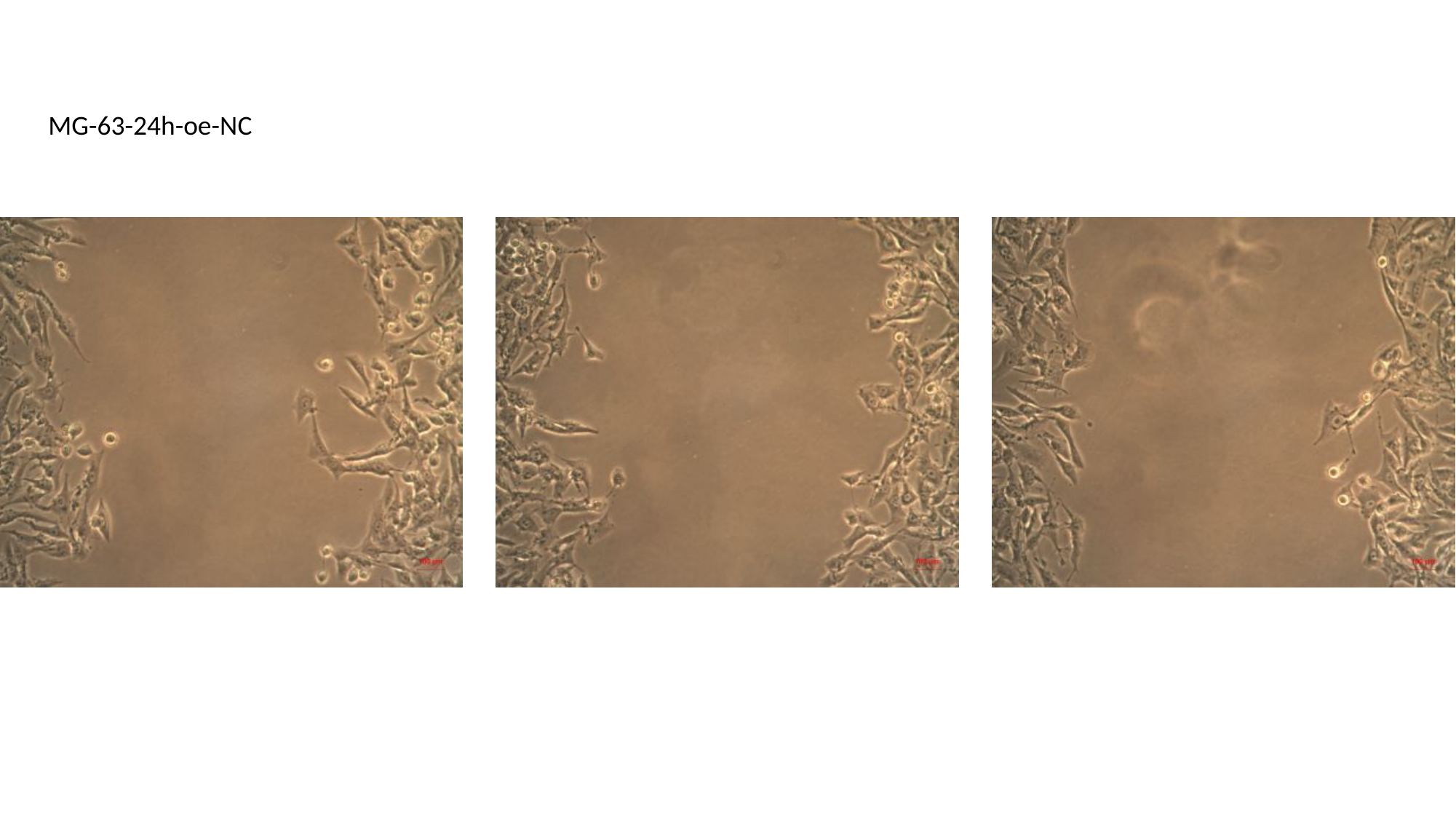

MG-63-24h-oe-NC

## Slide 9
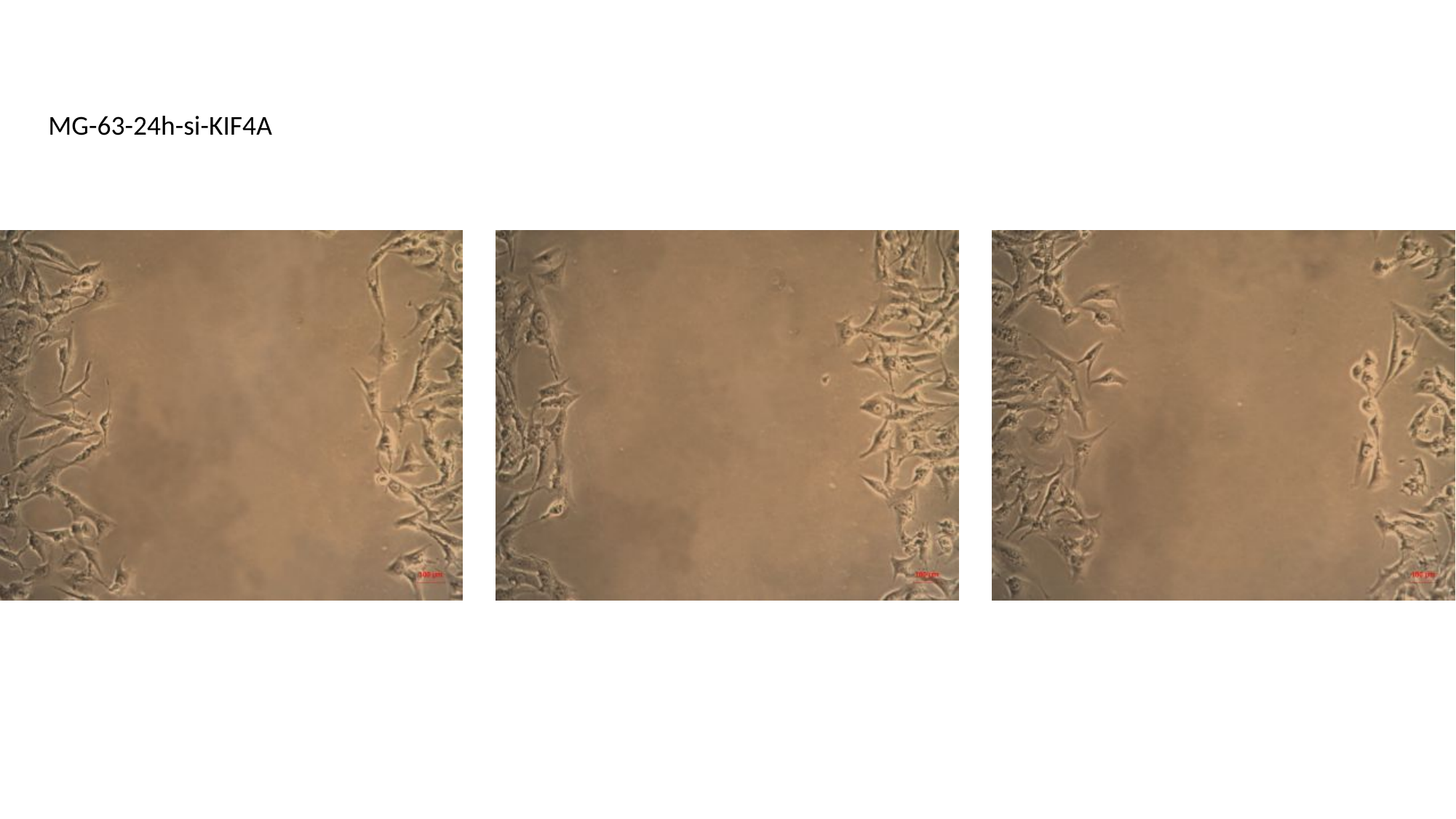

MG-63-24h-si-KIF4A

## Slide 10
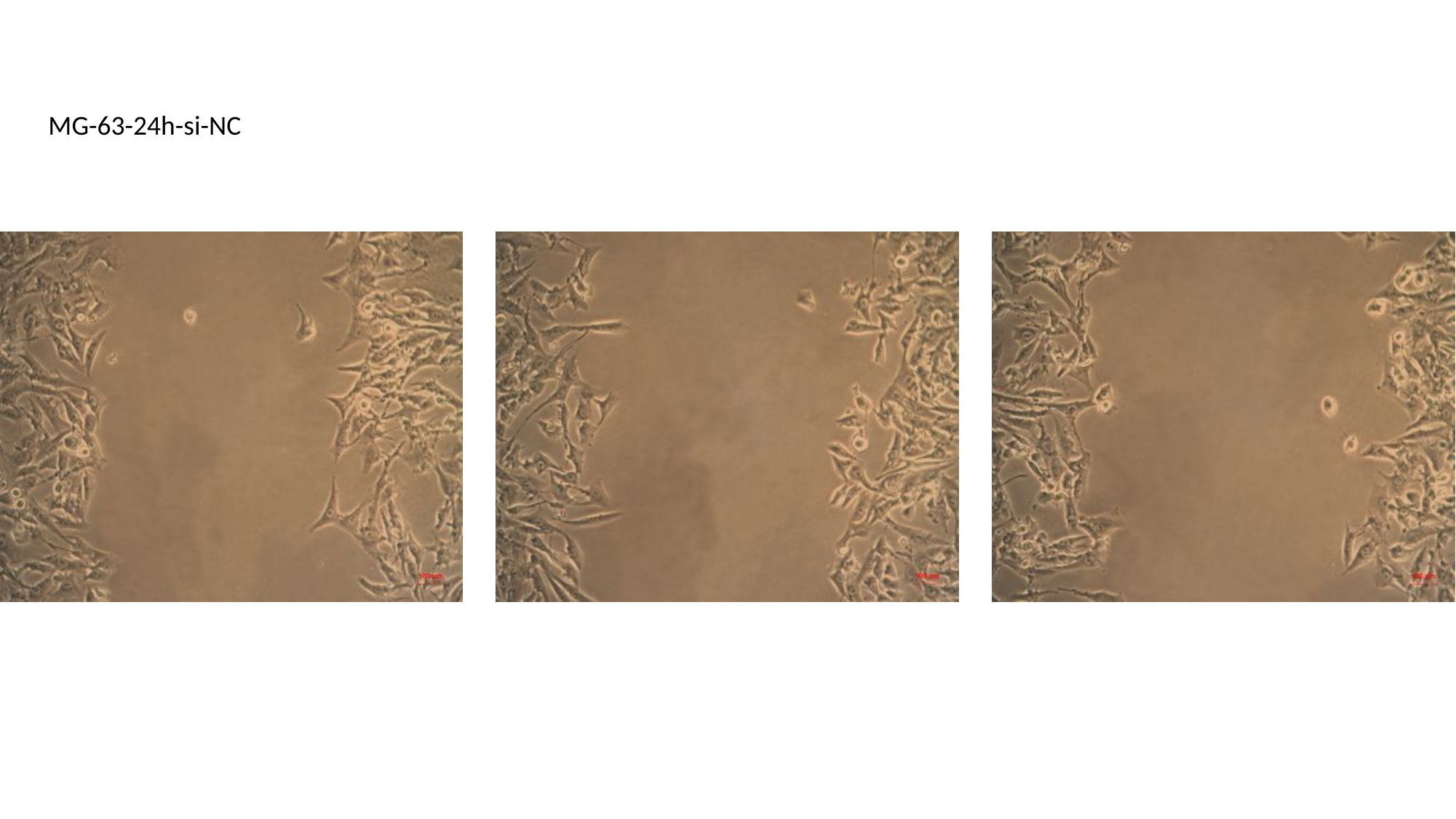

MG-63-24h-si-NC

## Slide 11
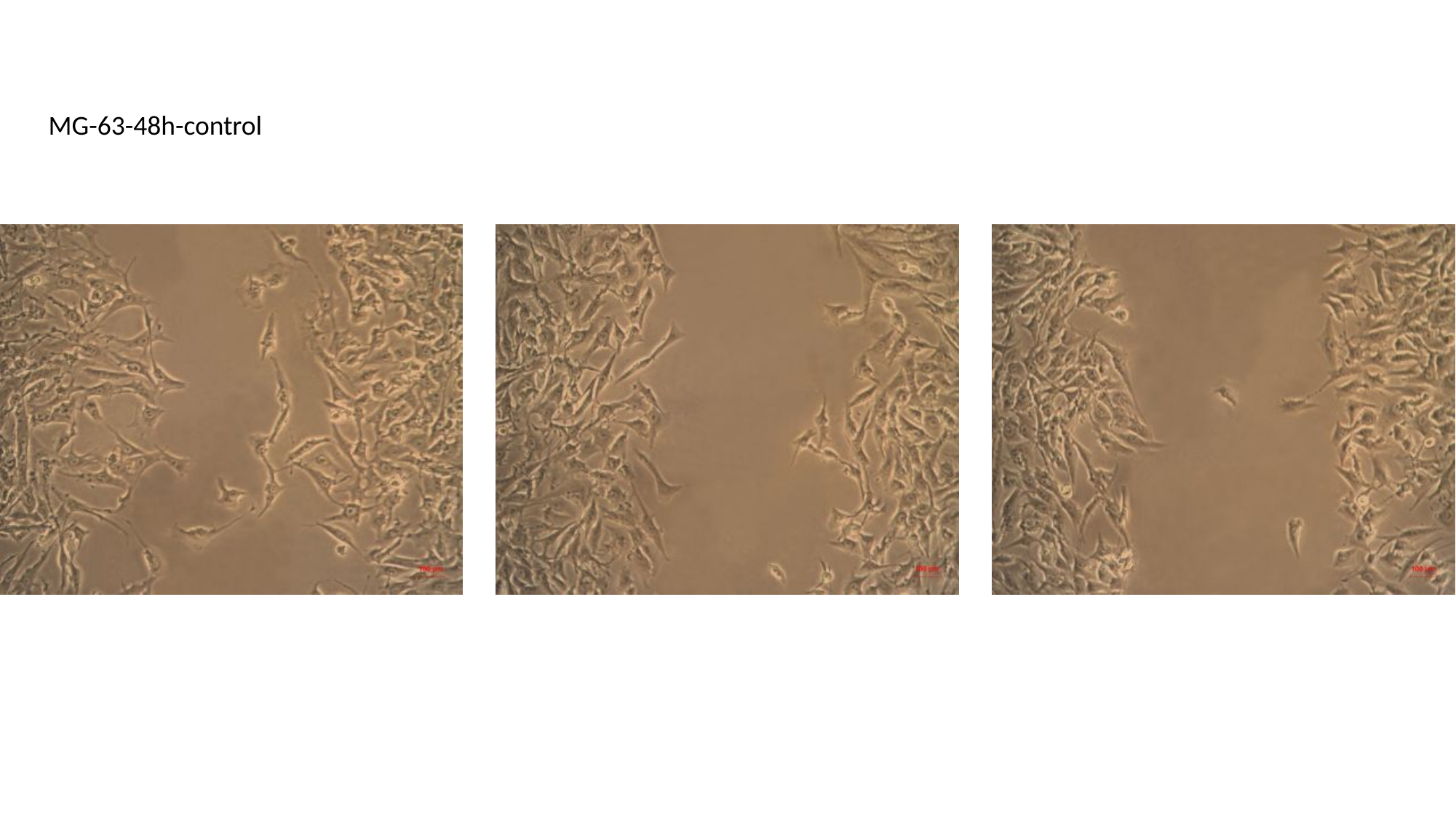

MG-63-48h-control

## Slide 12
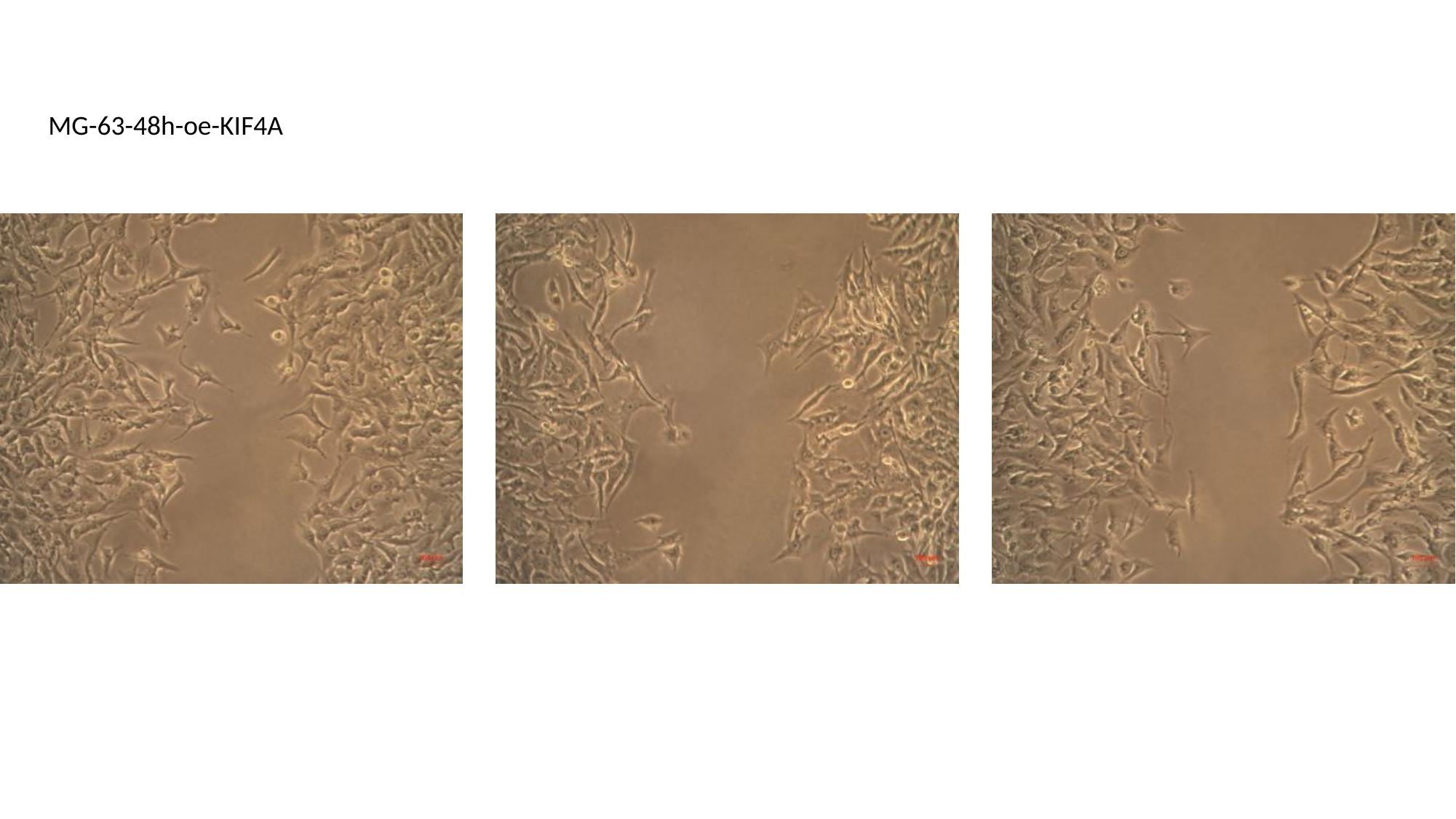

MG-63-48h-oe-KIF4A

## Slide 13
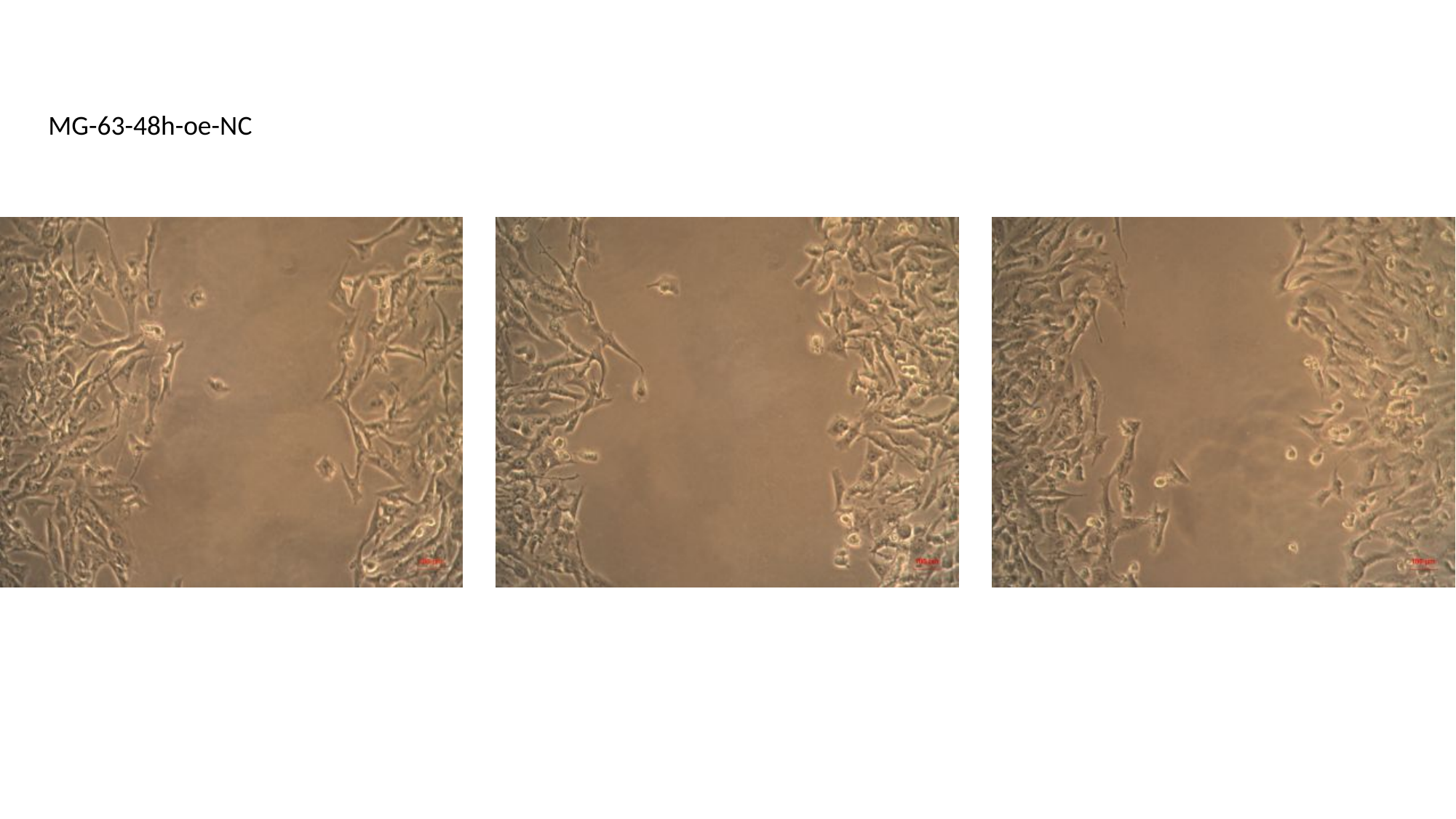

MG-63-48h-oe-NC

## Slide 14
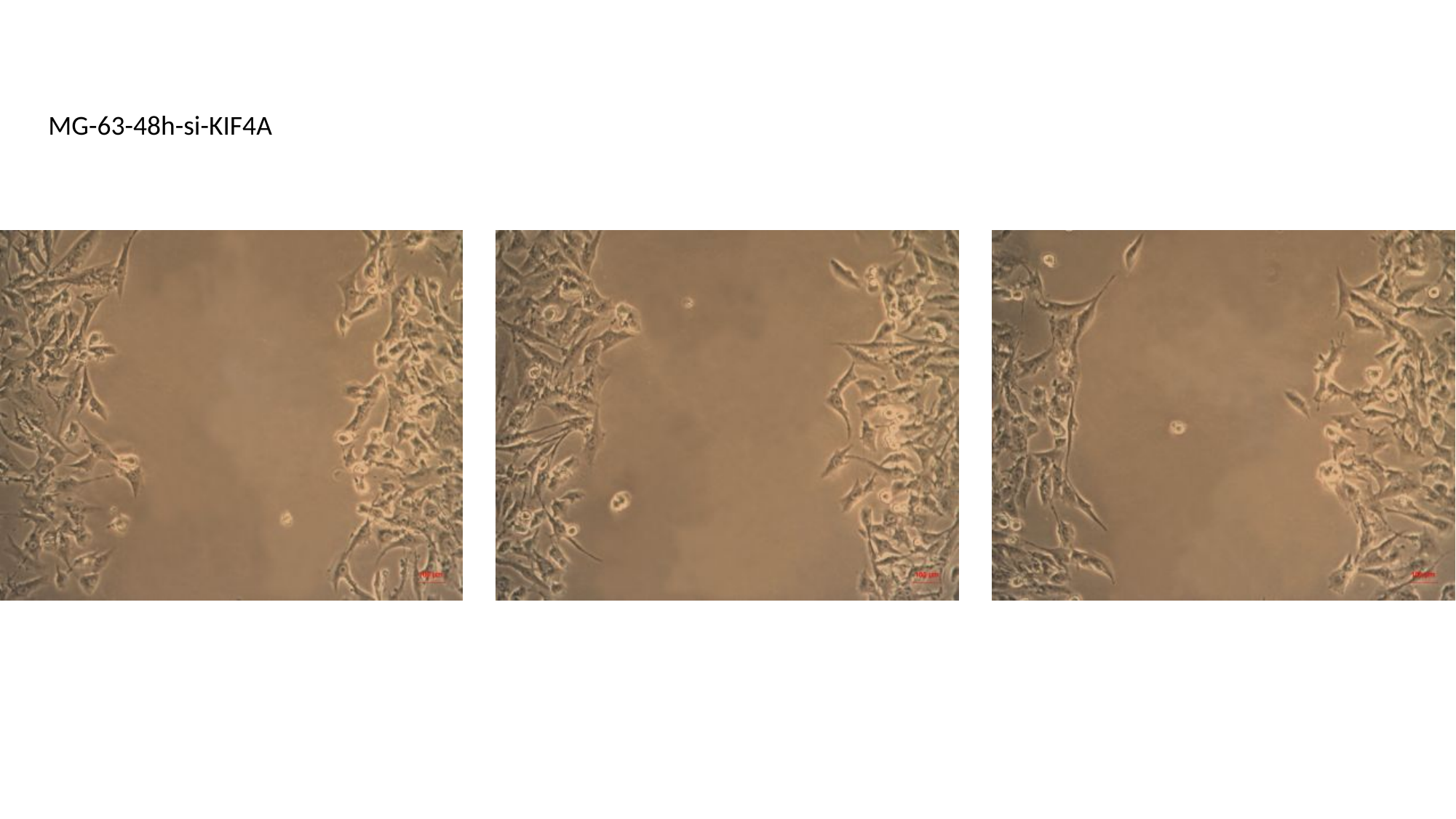

MG-63-48h-si-KIF4A

## Slide 15
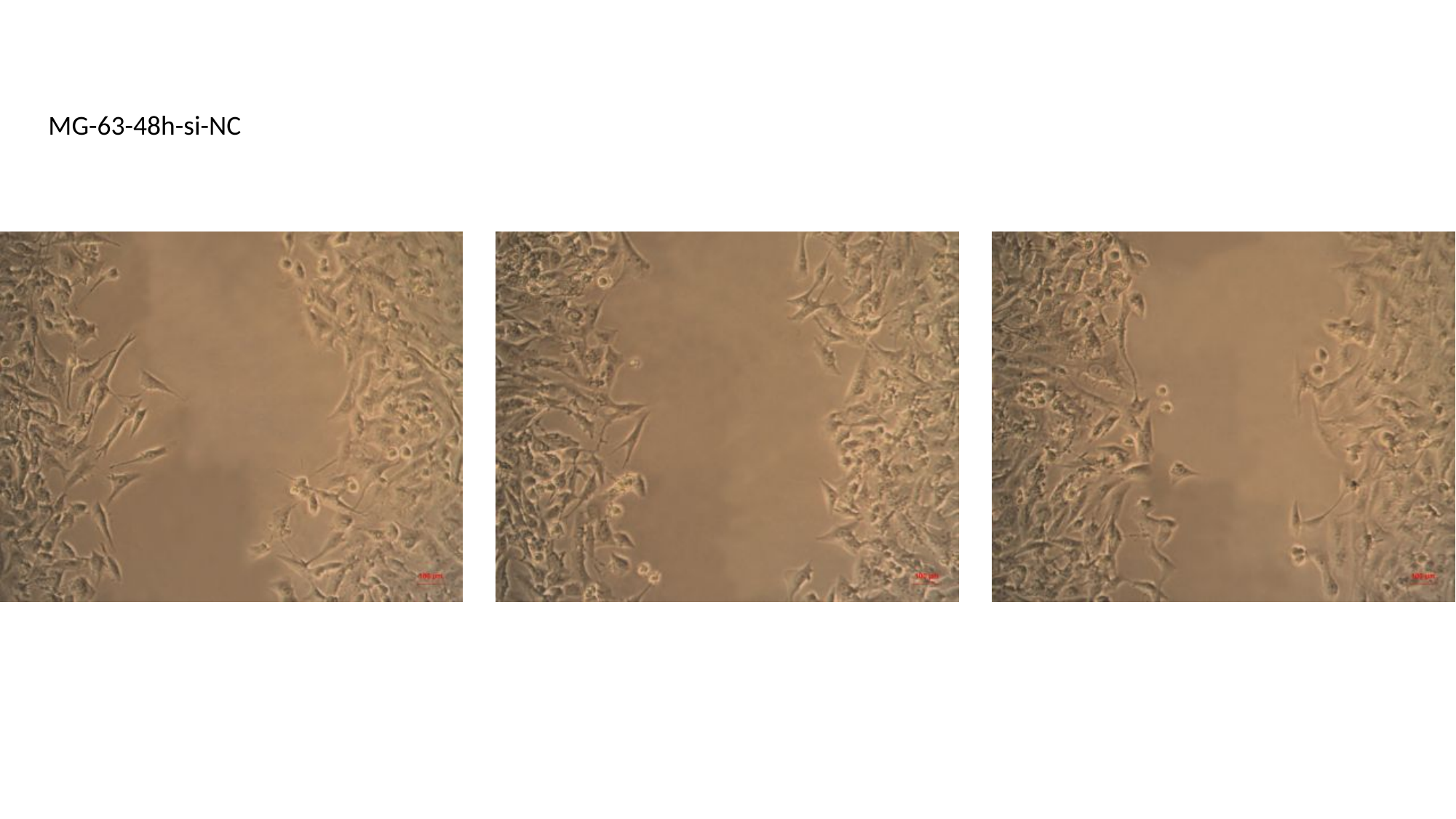

MG-63-48h-si-NC

## Slide 16
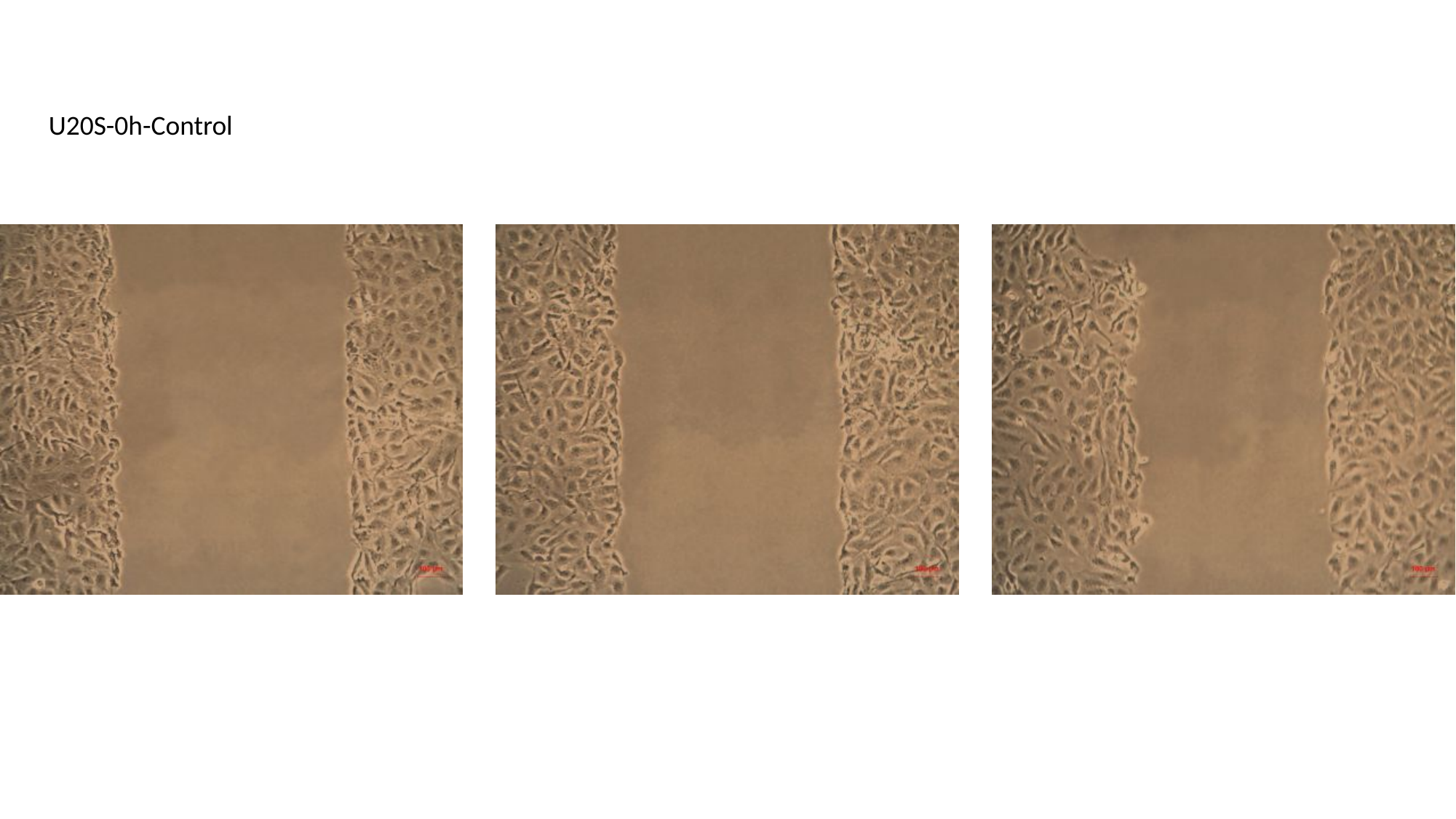

U20S-0h-Control

## Slide 17
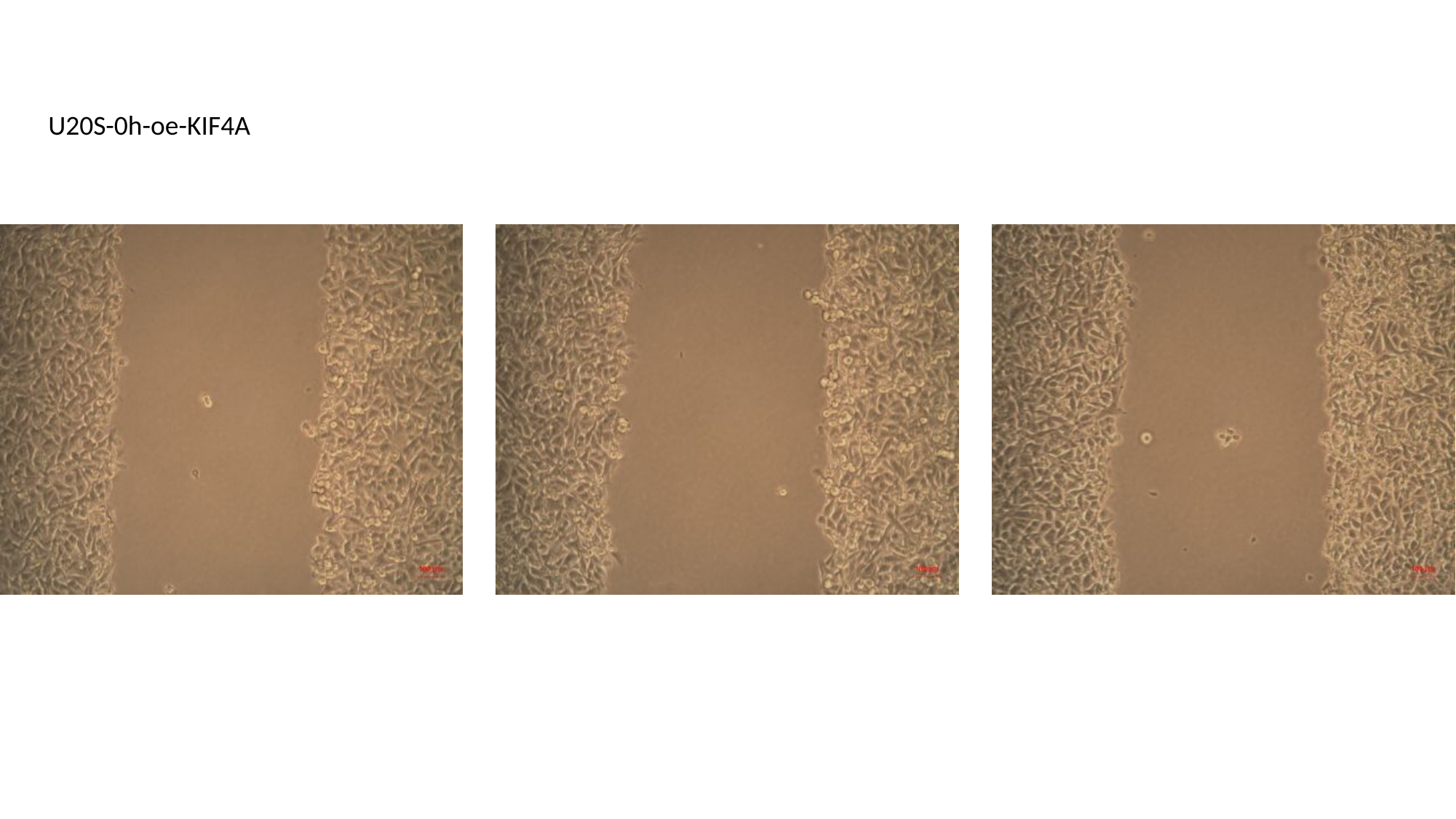

U20S-0h-oe-KIF4A

## Slide 18
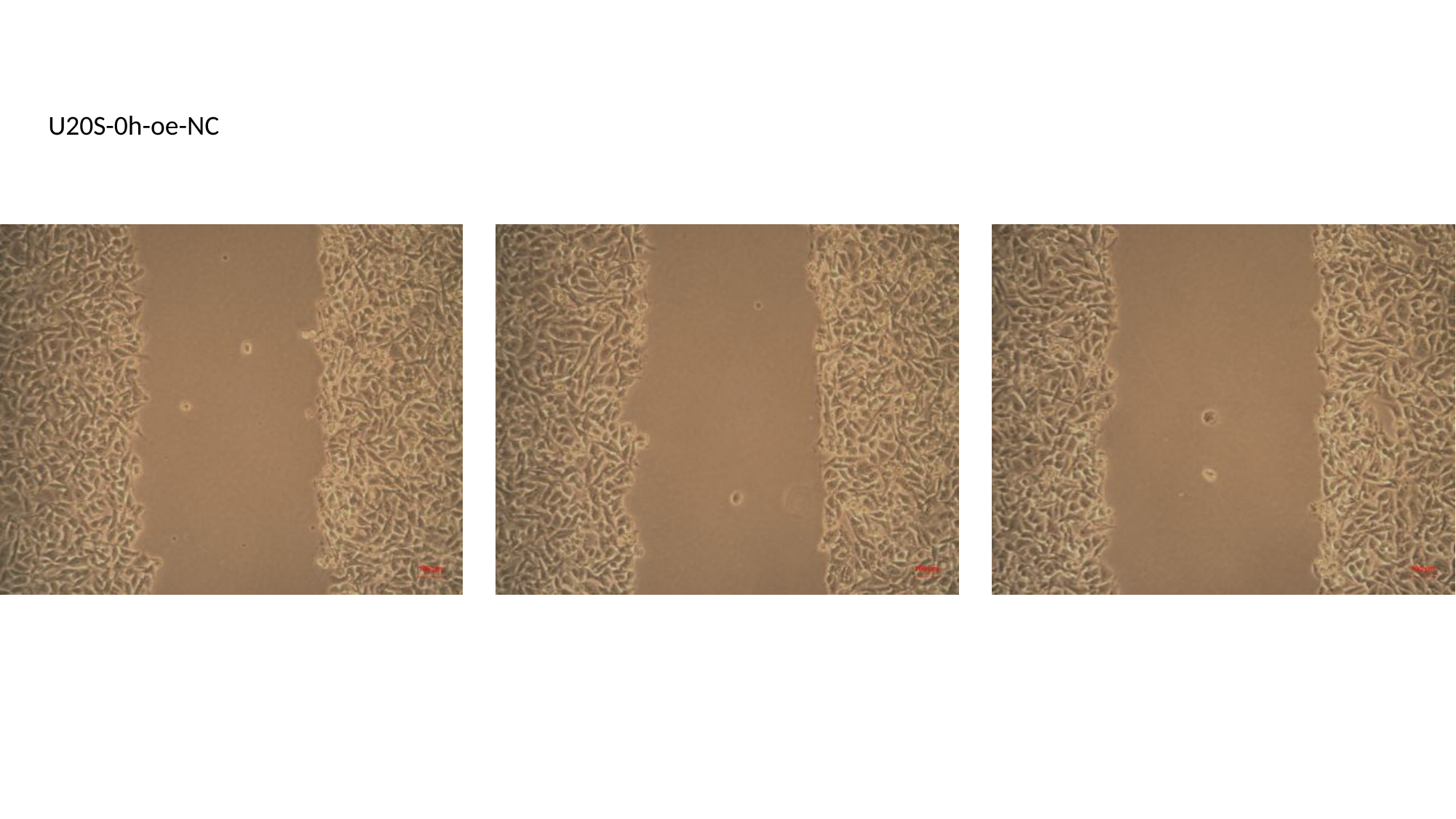

U20S-0h-oe-NC

## Slide 19
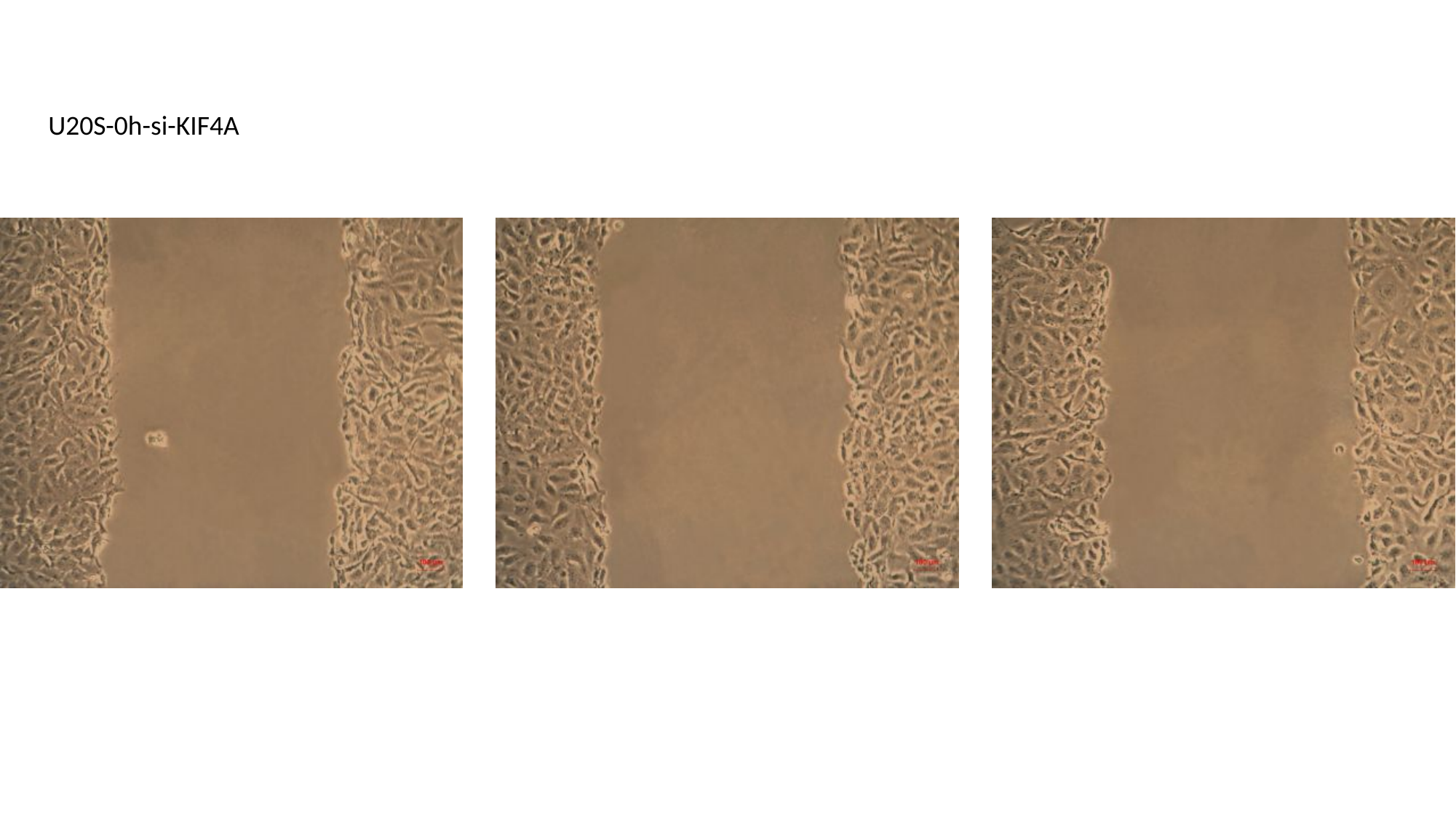

U20S-0h-si-KIF4A

## Slide 20
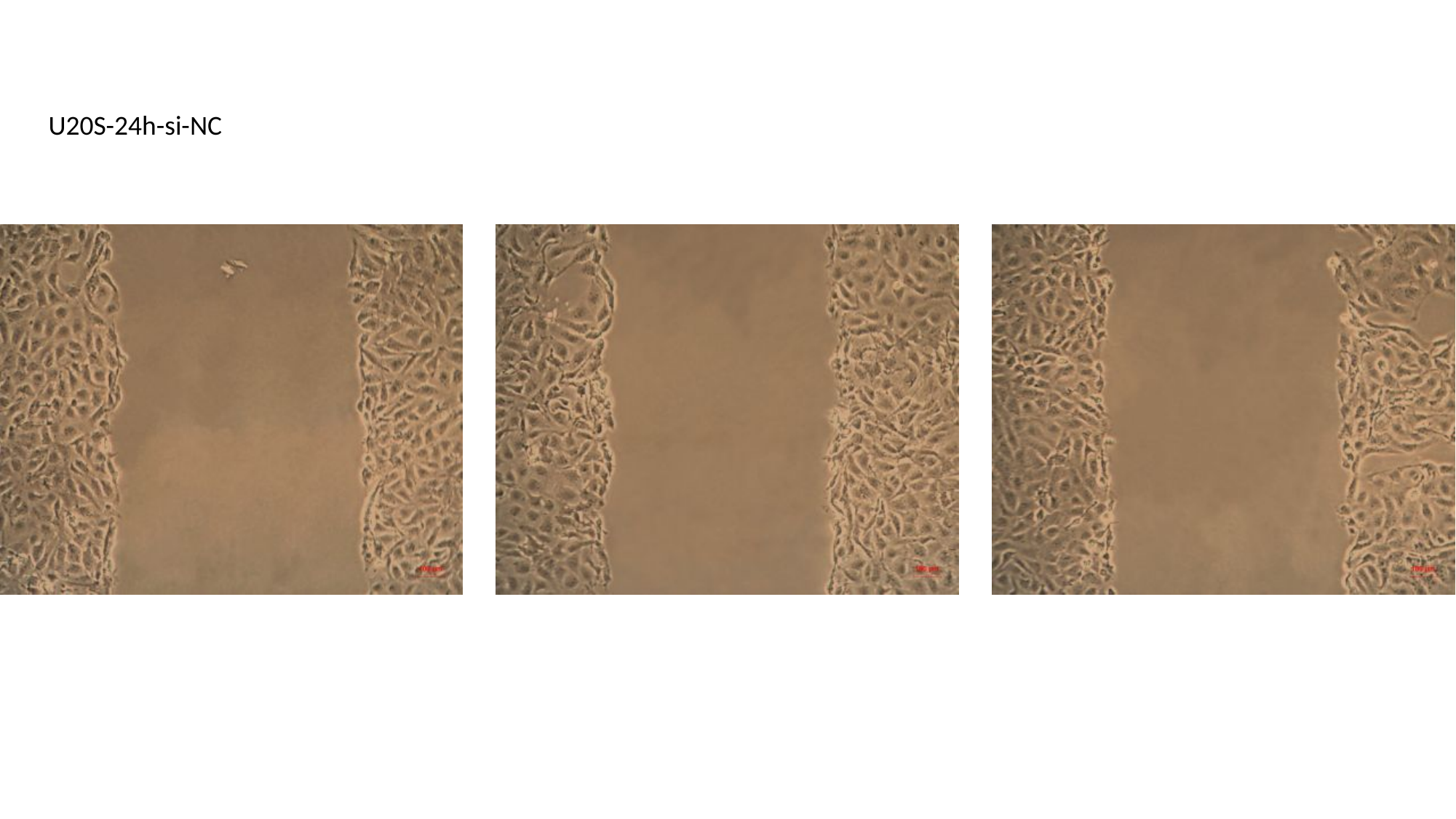

U20S-24h-si-NC

## Slide 21
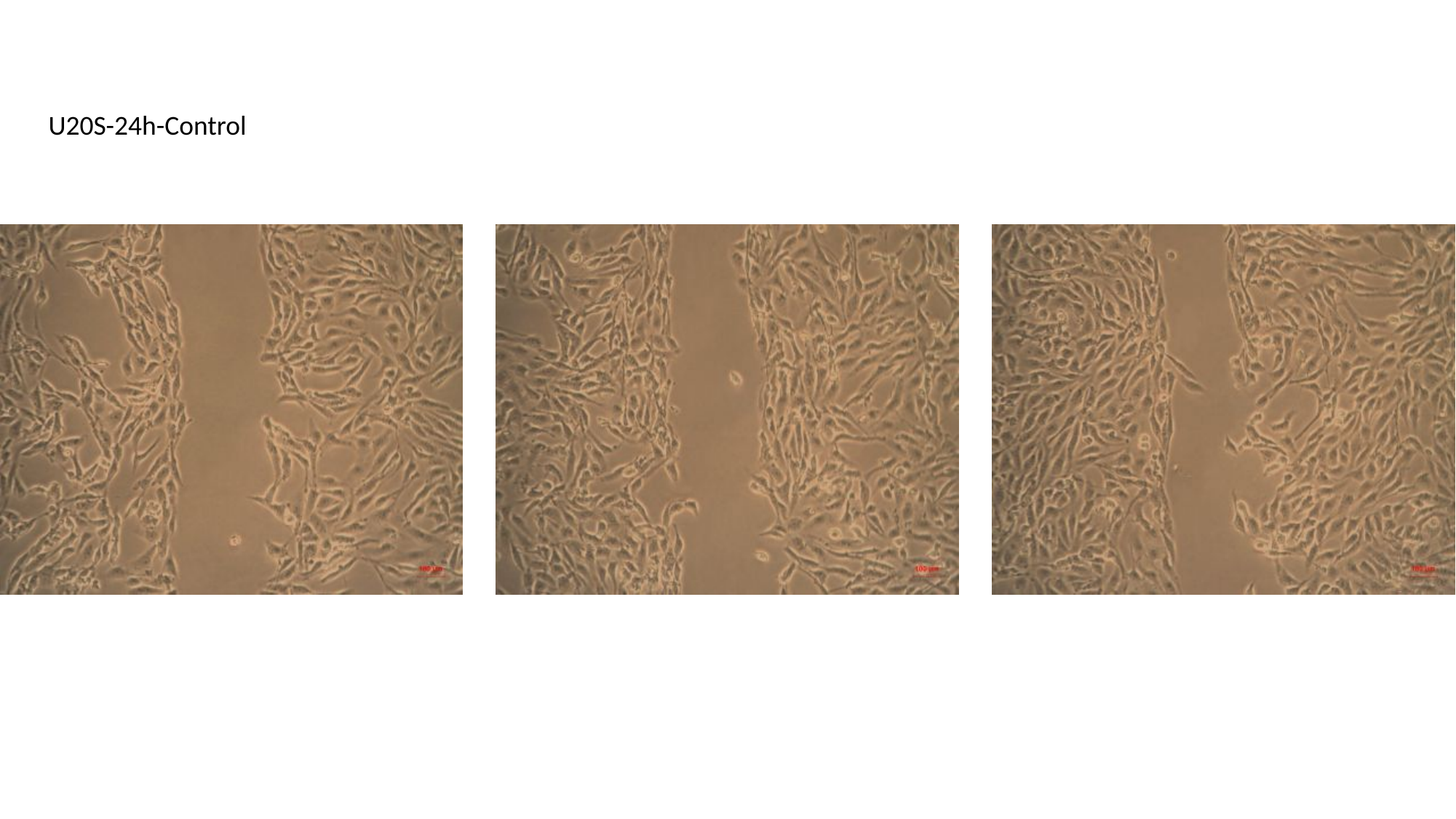

U20S-24h-Control

## Slide 22
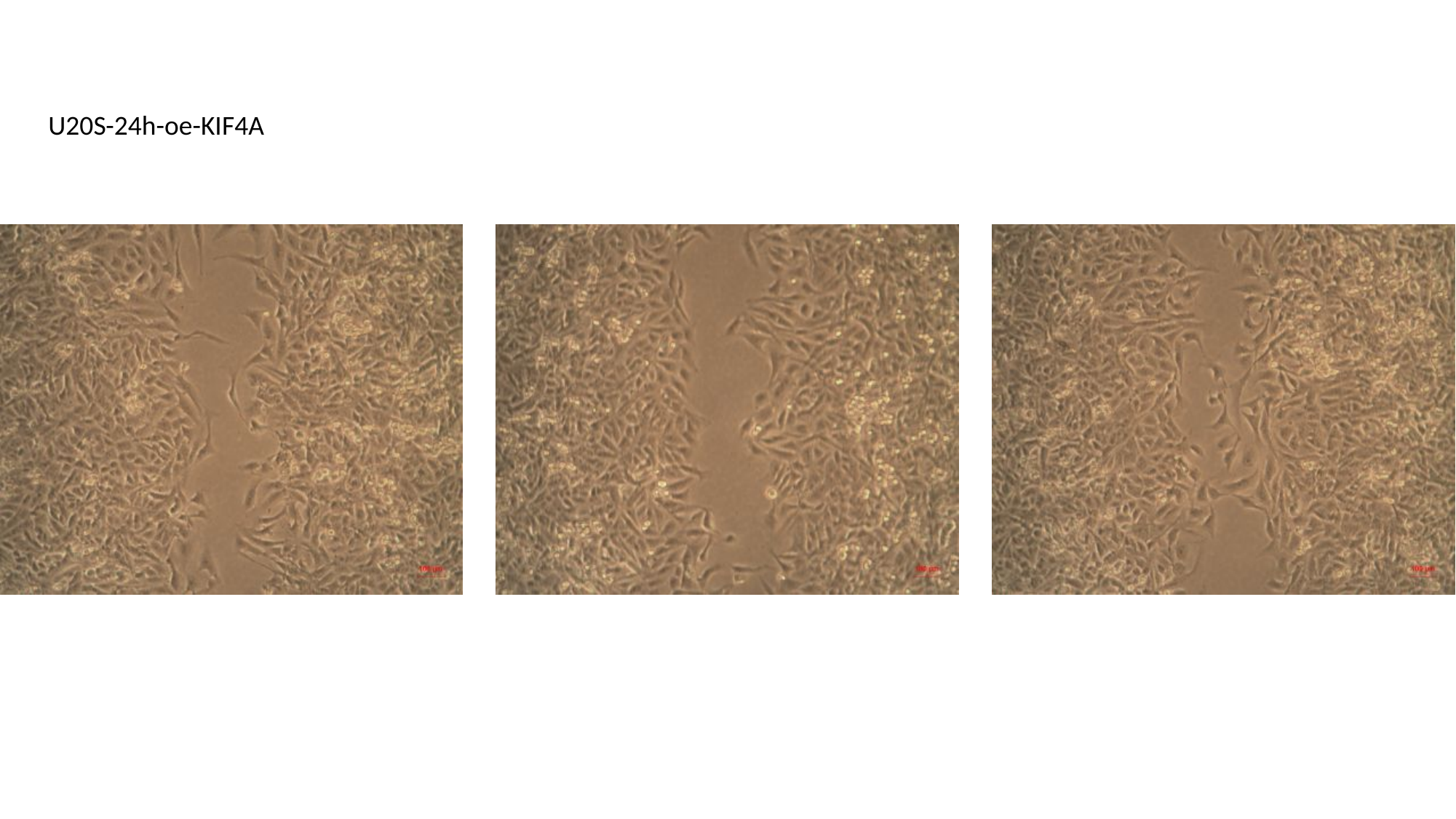

U20S-24h-oe-KIF4A

## Slide 23
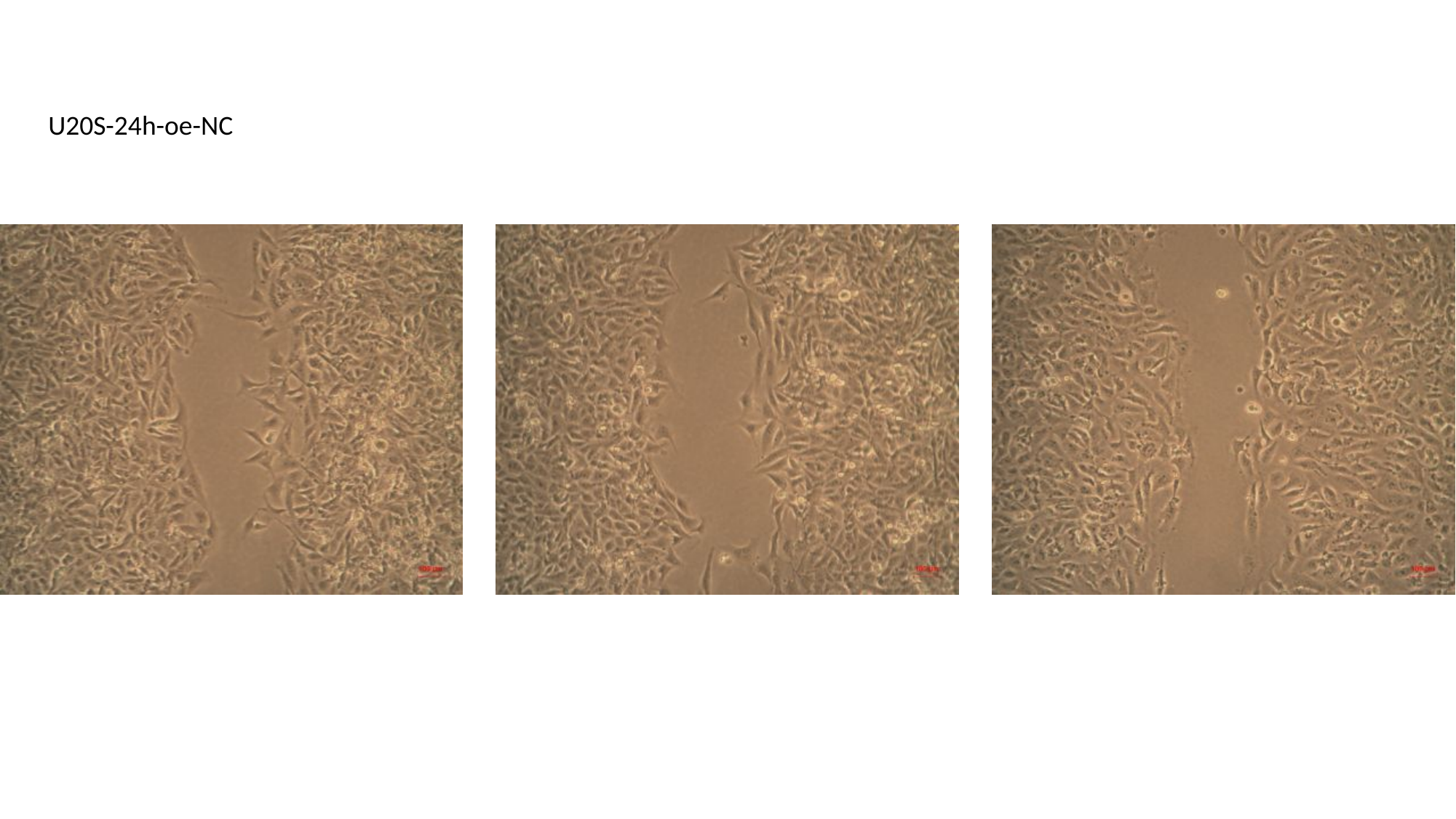

U20S-24h-oe-NC

## Slide 24
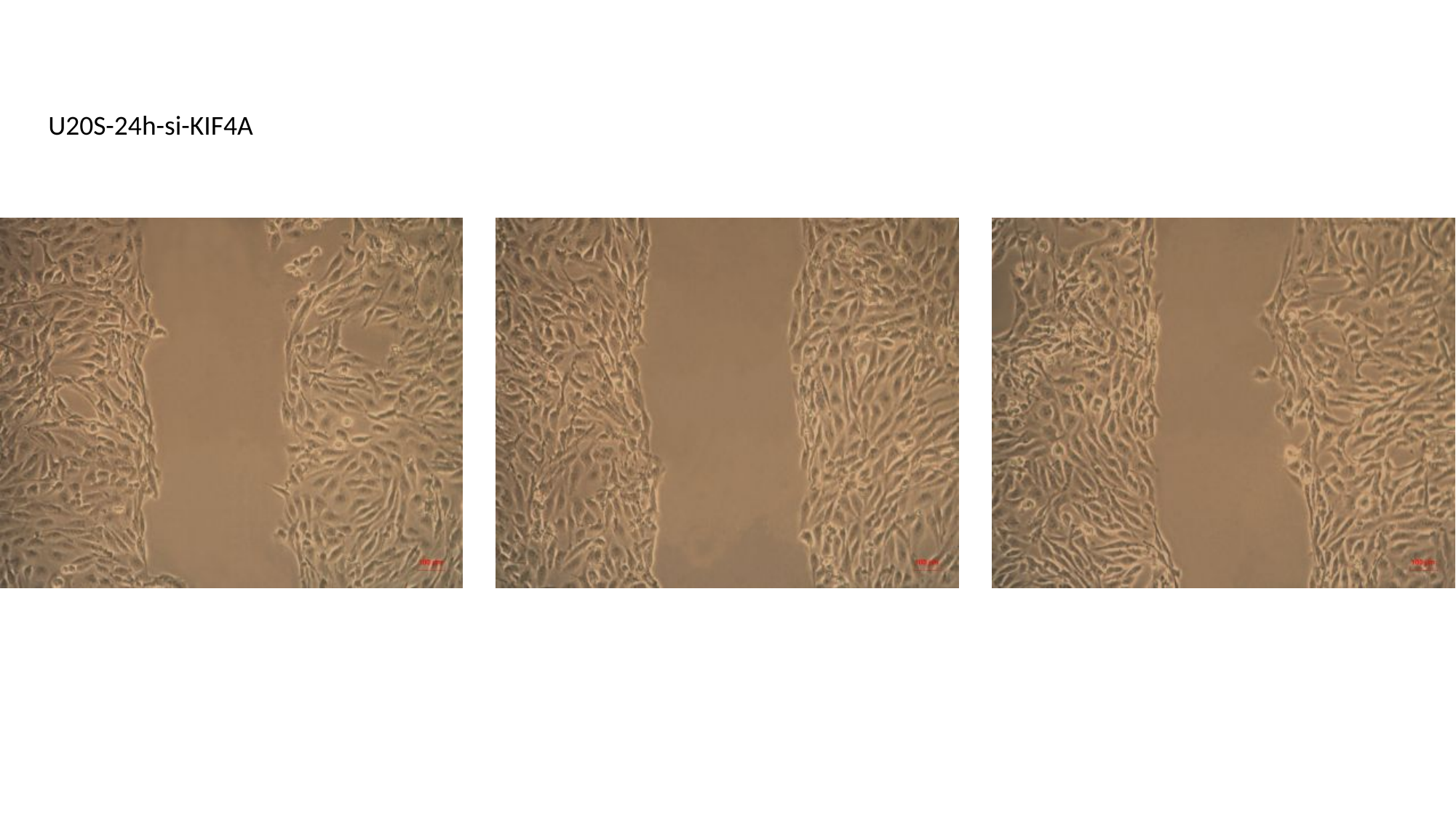

U20S-24h-si-KIF4A

## Slide 25
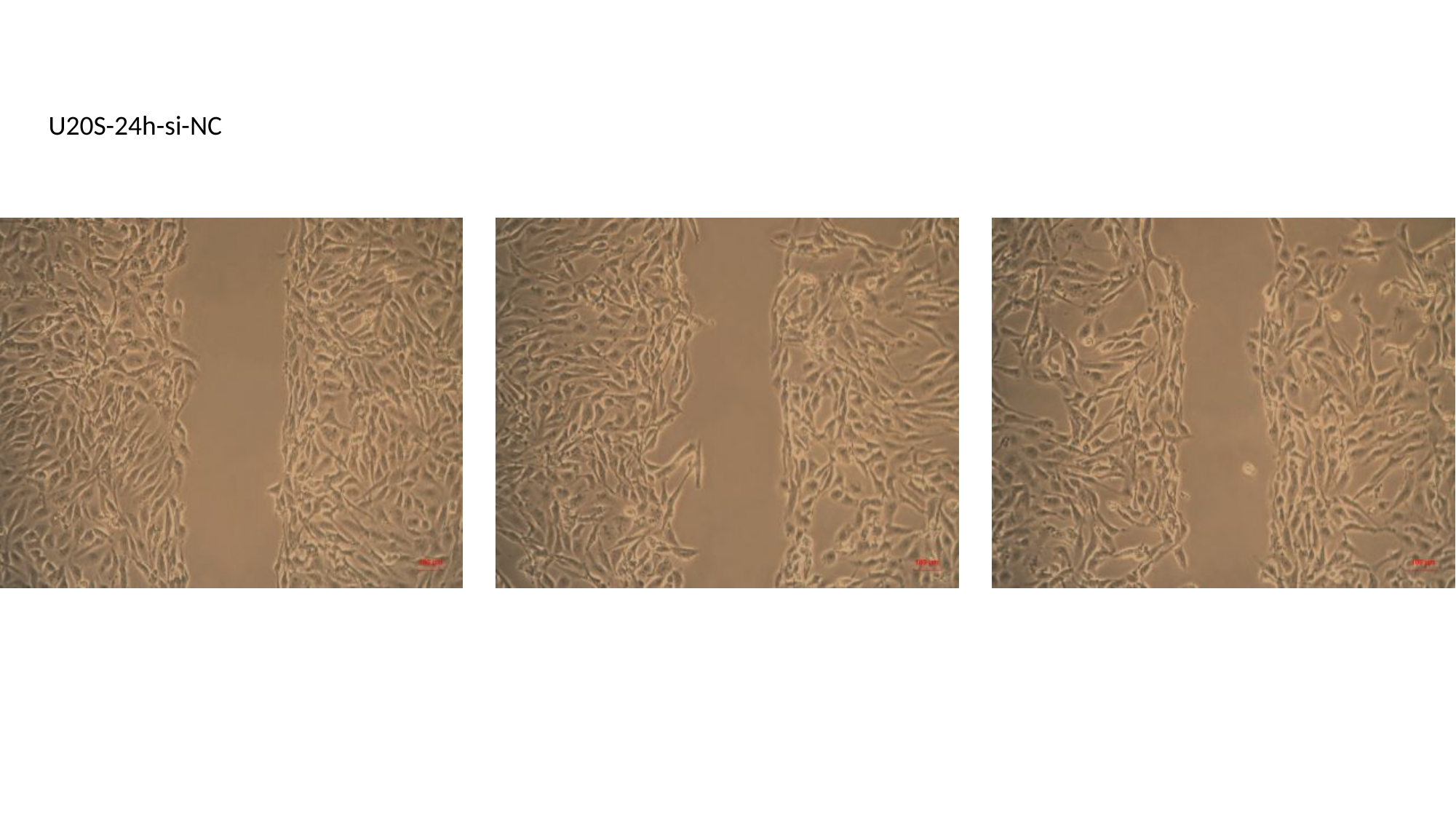

U20S-24h-si-NC

## Slide 26
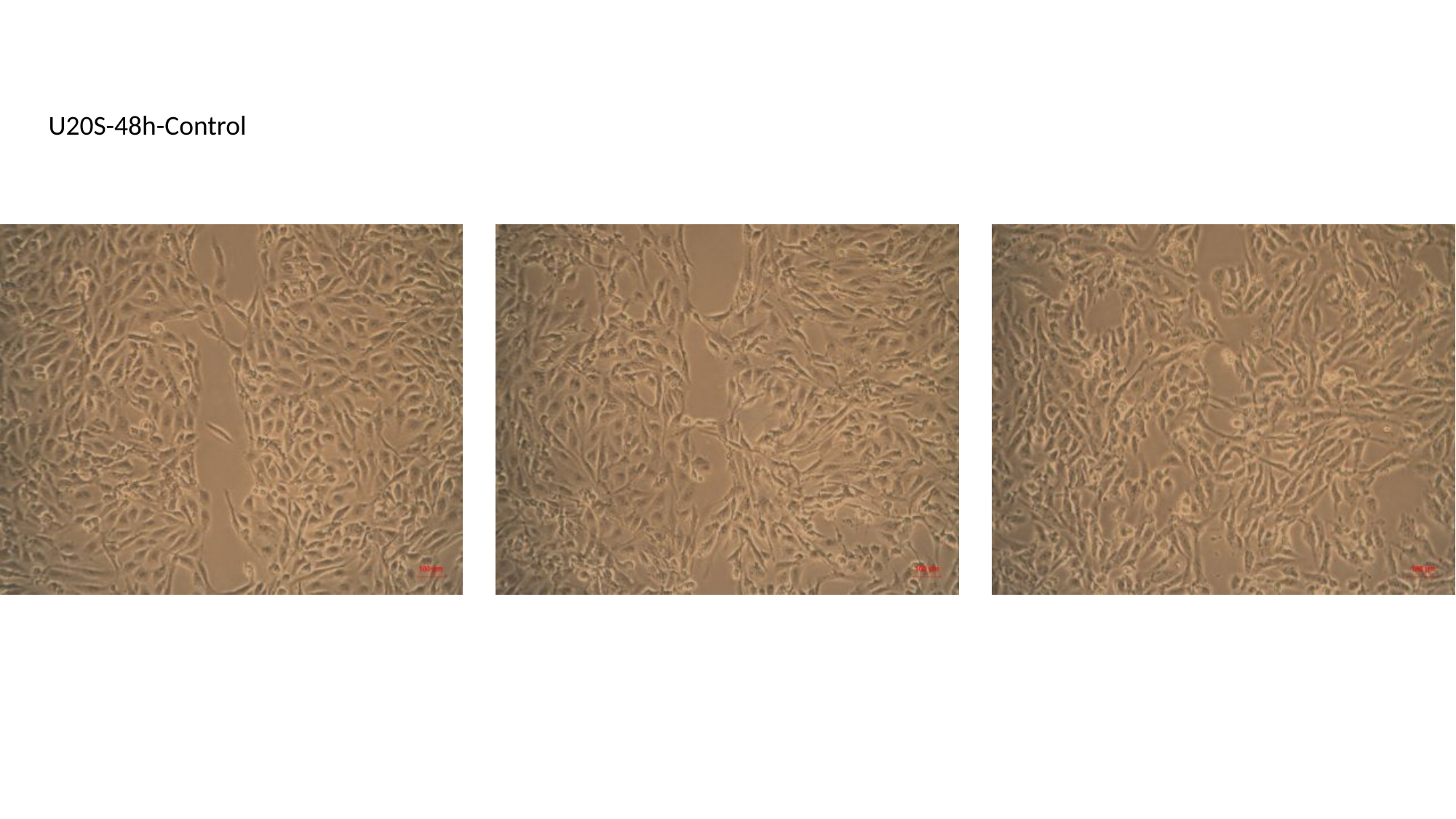

U20S-48h-Control

## Slide 27
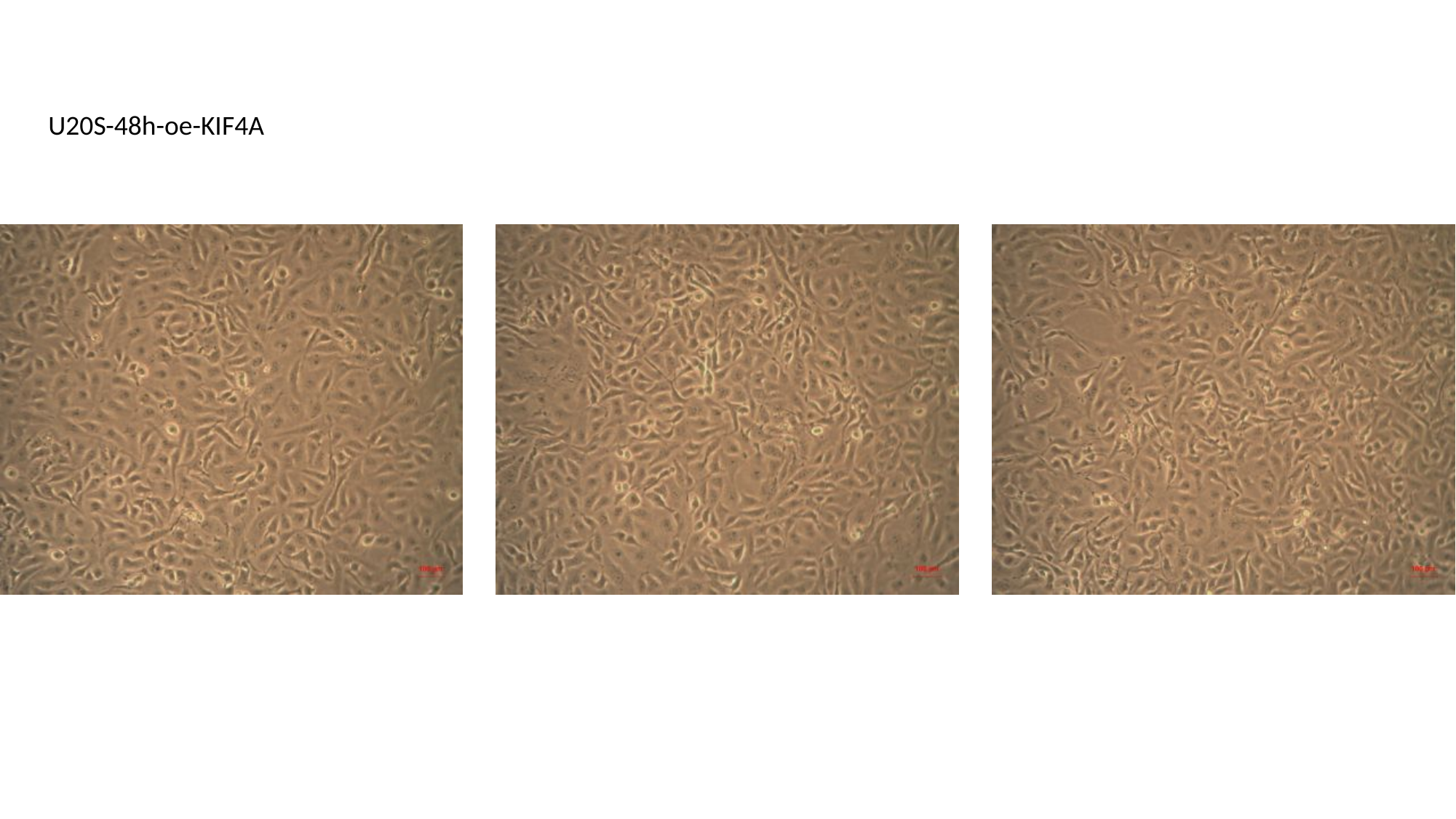

U20S-48h-oe-KIF4A

## Slide 28
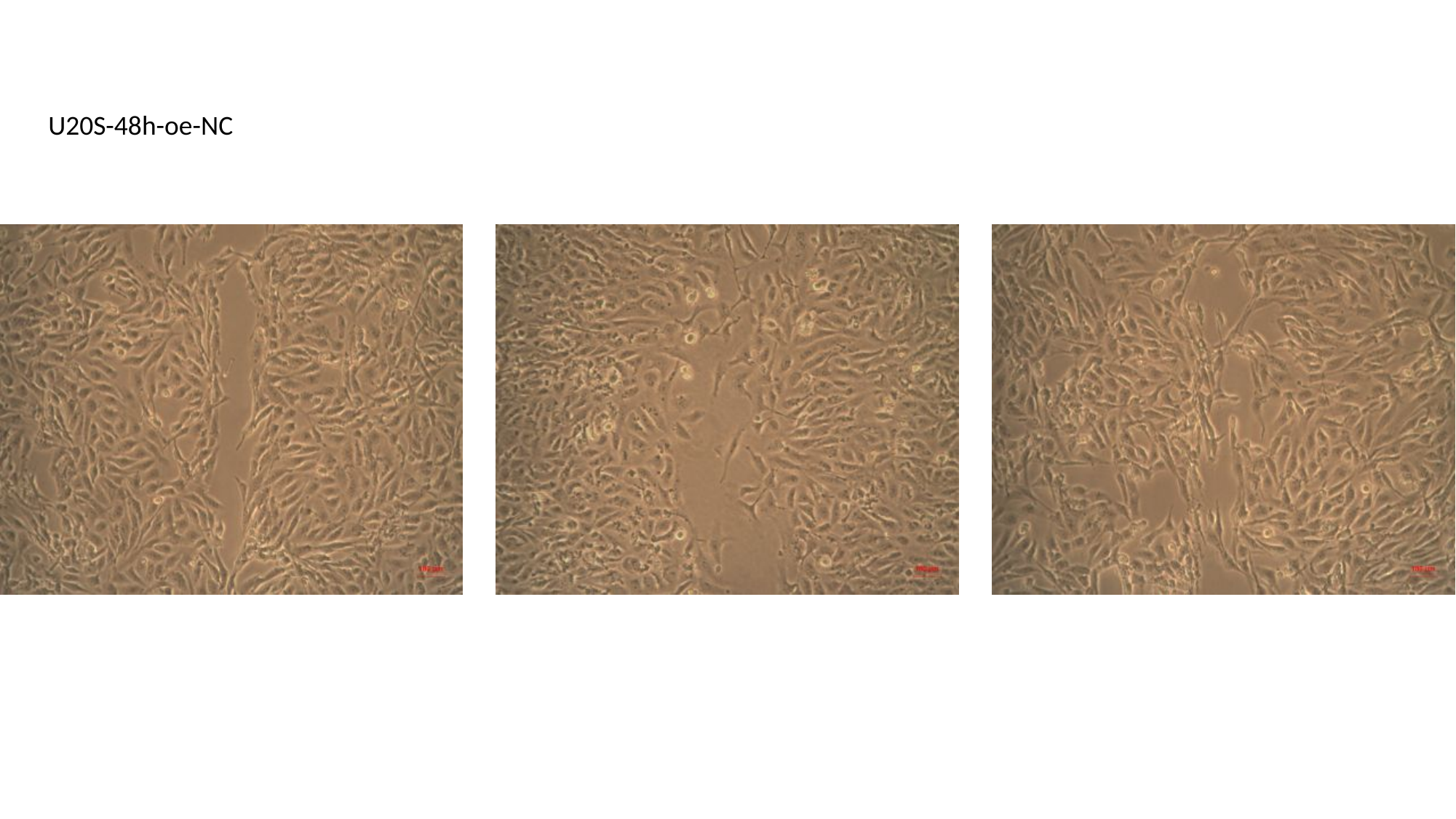

U20S-48h-oe-NC

## Slide 29
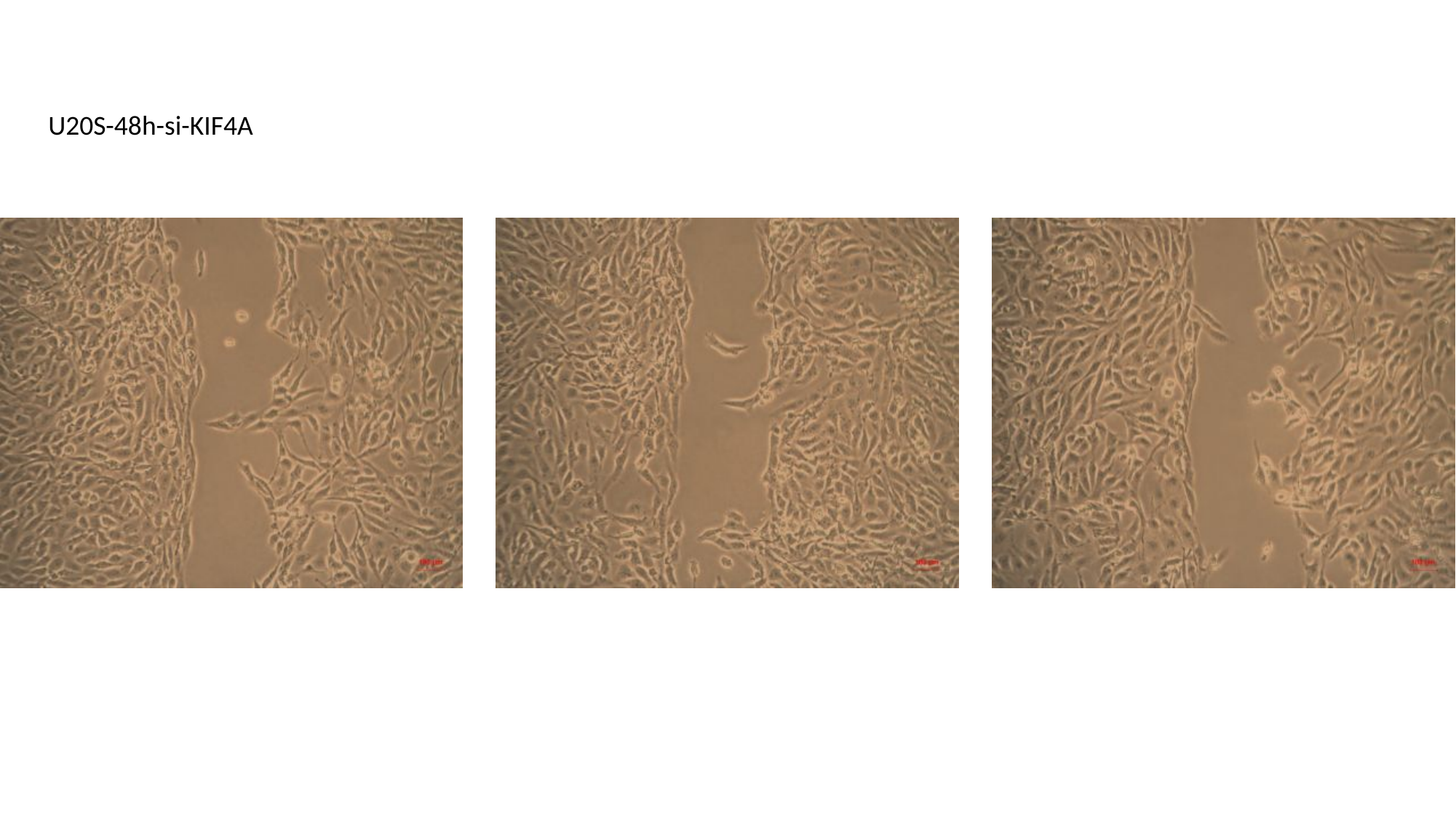

U20S-48h-si-KIF4A

## Slide 30
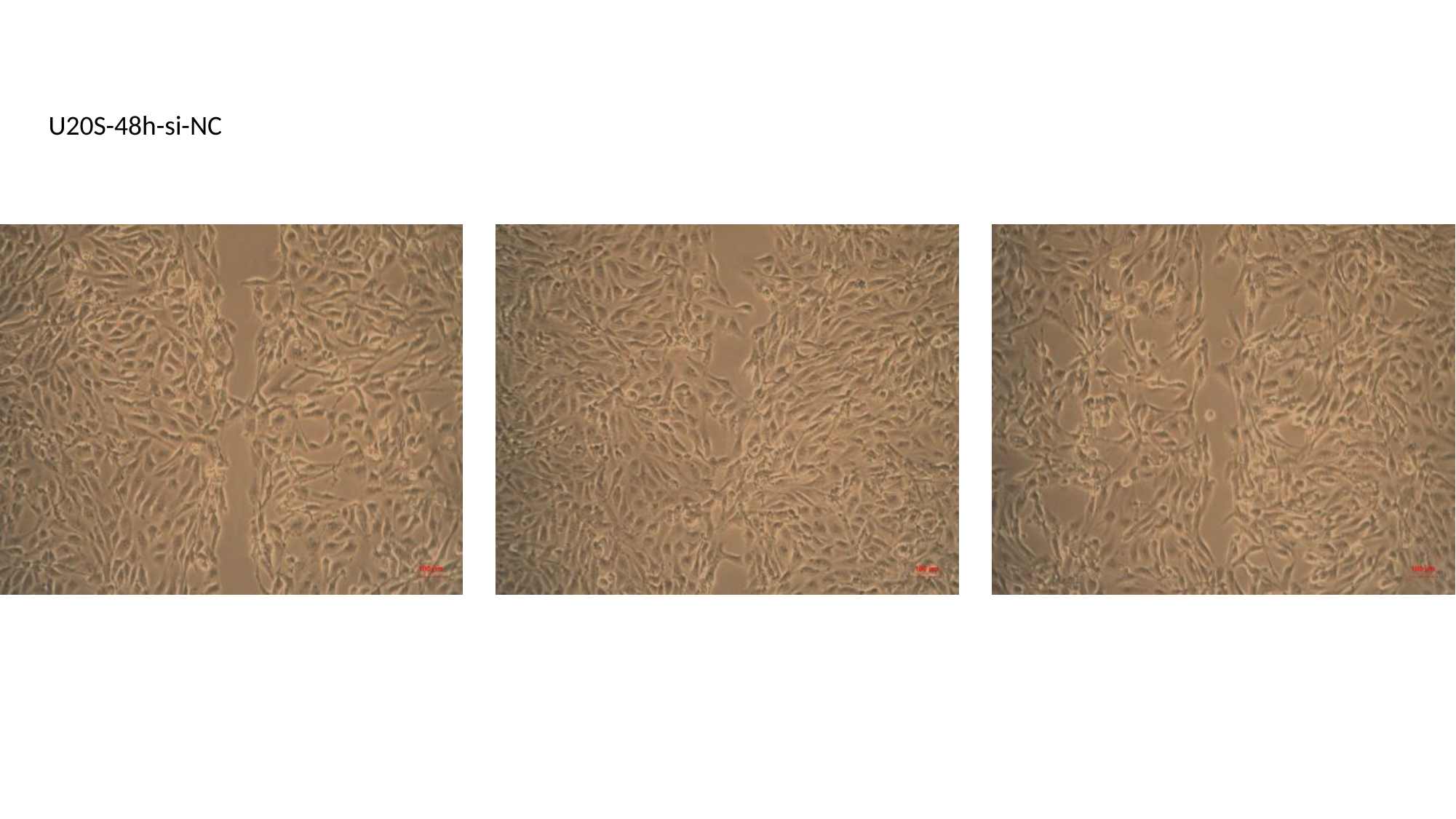

U20S-48h-si-NC
