# Supplementary figures and images for: Identification of KIF4A as a pan-cancer diagnostic and prognostic biomarker via bioinformatics analysis and validation in osteosarcoma cell lines (part 2 of 2)
Source: PeerJ. 2021 May 21;9:e11455. doi: 10.7717/peerj.11455 (PMC8142929; doi:10.7717/peerj.11455)

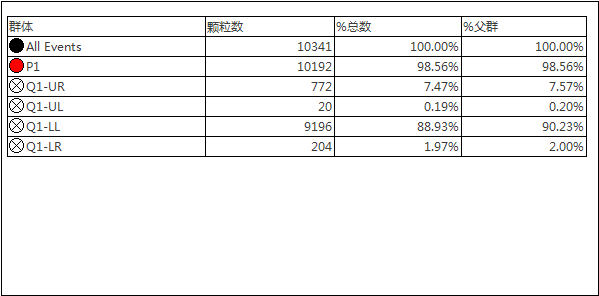

Supplement: Supplemental Information 11 [file peerj-09-11455-s011.zip › fig5A-Apoptosis/U20S/KIF4AsiRNA-1_Statistics1.bmp]

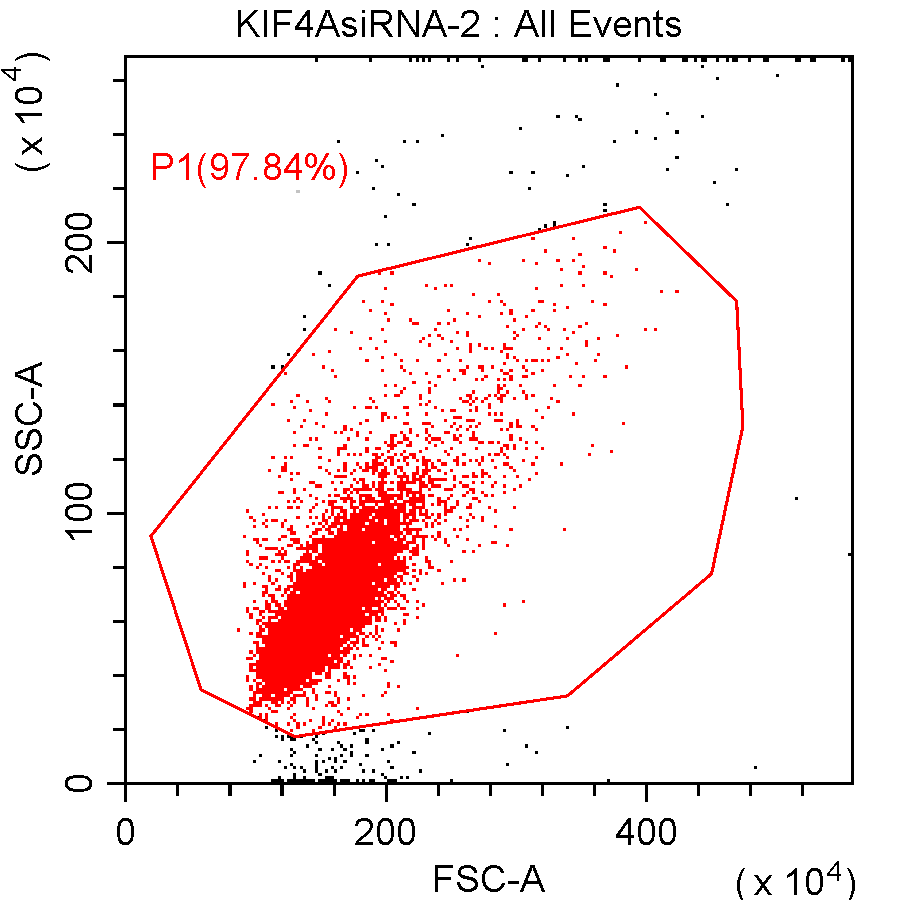

Supplement: Supplemental Information 11 [file peerj-09-11455-s011.zip › fig5A-Apoptosis/U20S/KIF4AsiRNA-2_Plot1.bmp]

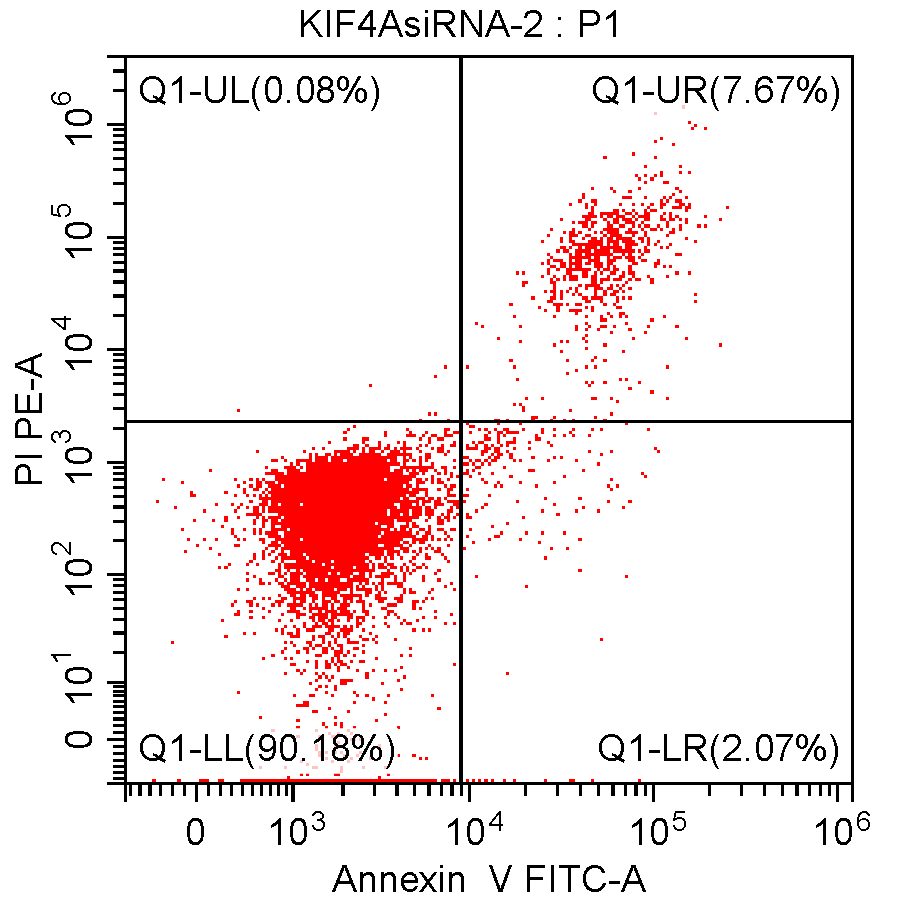

Supplement: Supplemental Information 11 [file peerj-09-11455-s011.zip › fig5A-Apoptosis/U20S/KIF4AsiRNA-2_Plot2.bmp]

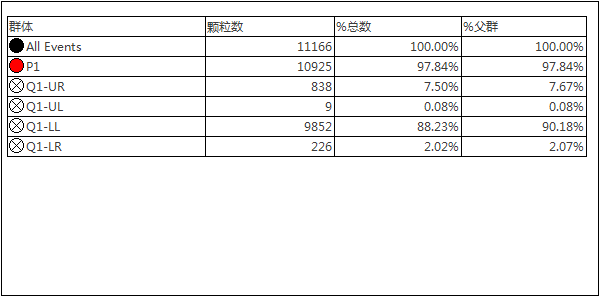

Supplement: Supplemental Information 11 [file peerj-09-11455-s011.zip › fig5A-Apoptosis/U20S/KIF4AsiRNA-2_Statistics1.bmp]

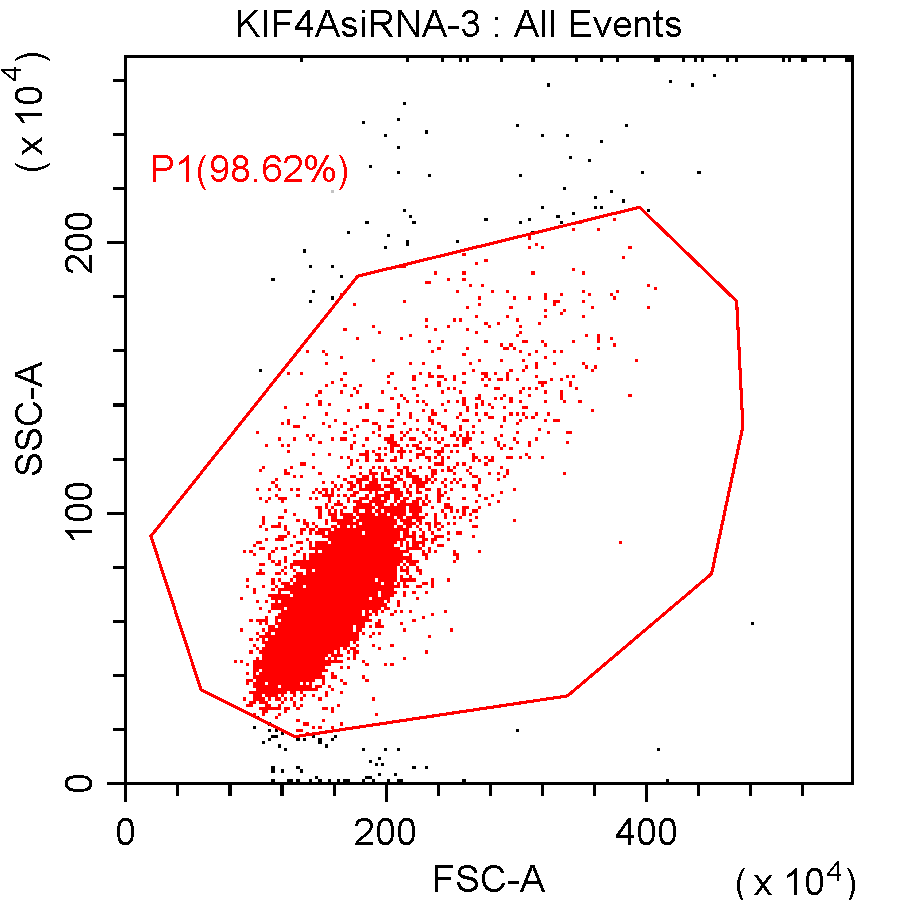

Supplement: Supplemental Information 11 [file peerj-09-11455-s011.zip › fig5A-Apoptosis/U20S/KIF4AsiRNA-3_Plot1.bmp]

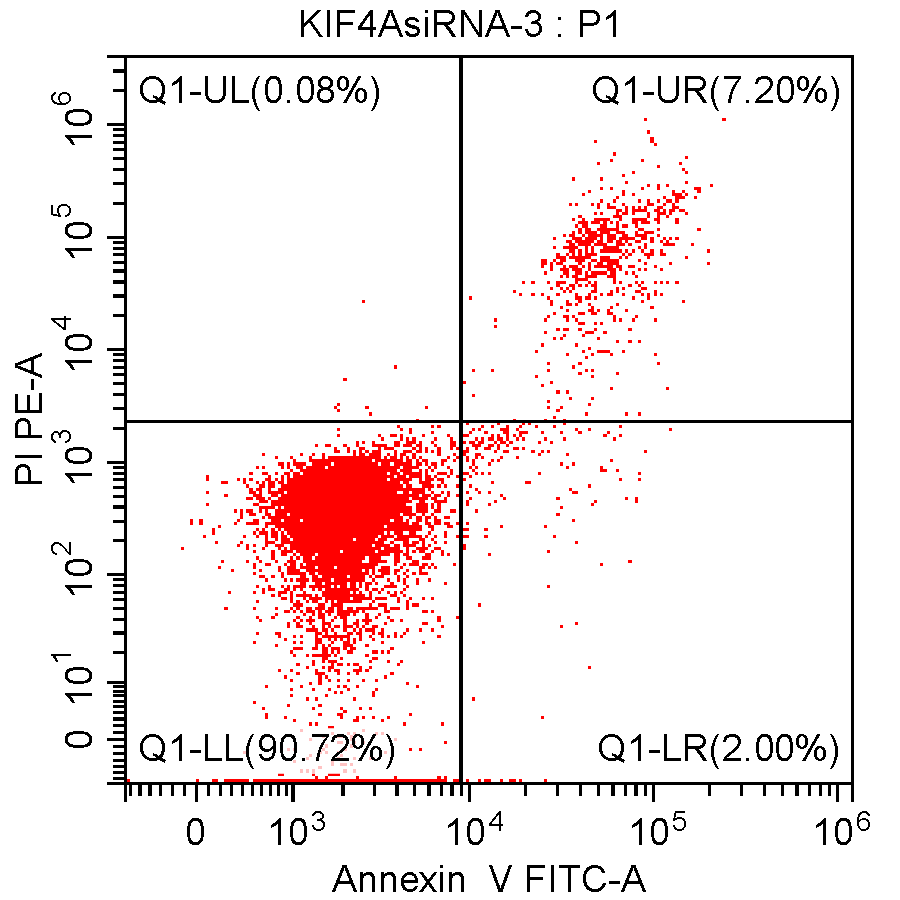

Supplement: Supplemental Information 11 [file peerj-09-11455-s011.zip › fig5A-Apoptosis/U20S/KIF4AsiRNA-3_Plot2.bmp]

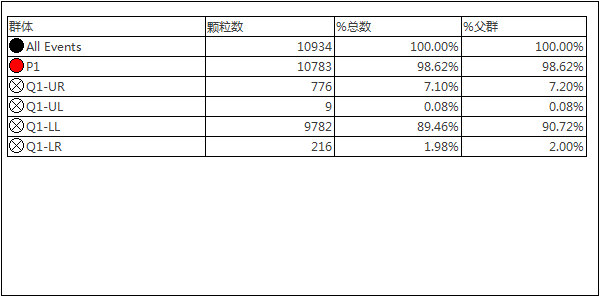

Supplement: Supplemental Information 11 [file peerj-09-11455-s011.zip › fig5A-Apoptosis/U20S/KIF4AsiRNA-3_Statistics1.bmp]

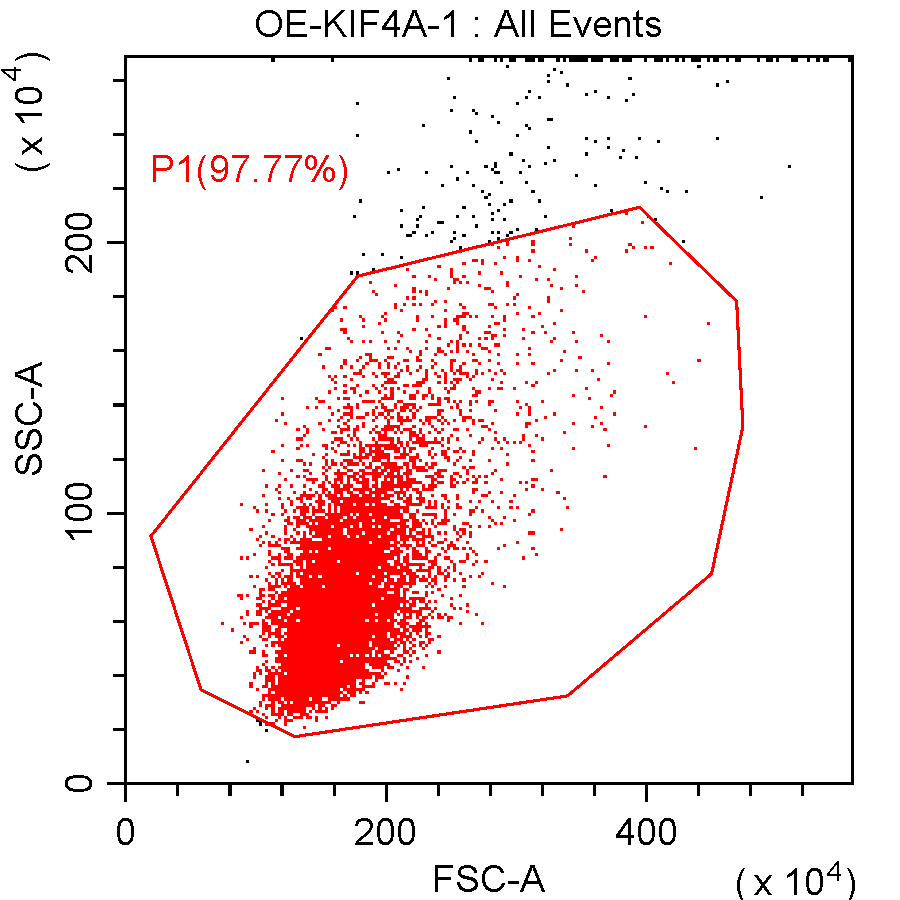

Supplement: Supplemental Information 11 [file peerj-09-11455-s011.zip › fig5A-Apoptosis/U20S/OE-KIF4A-1_Plot1.bmp]

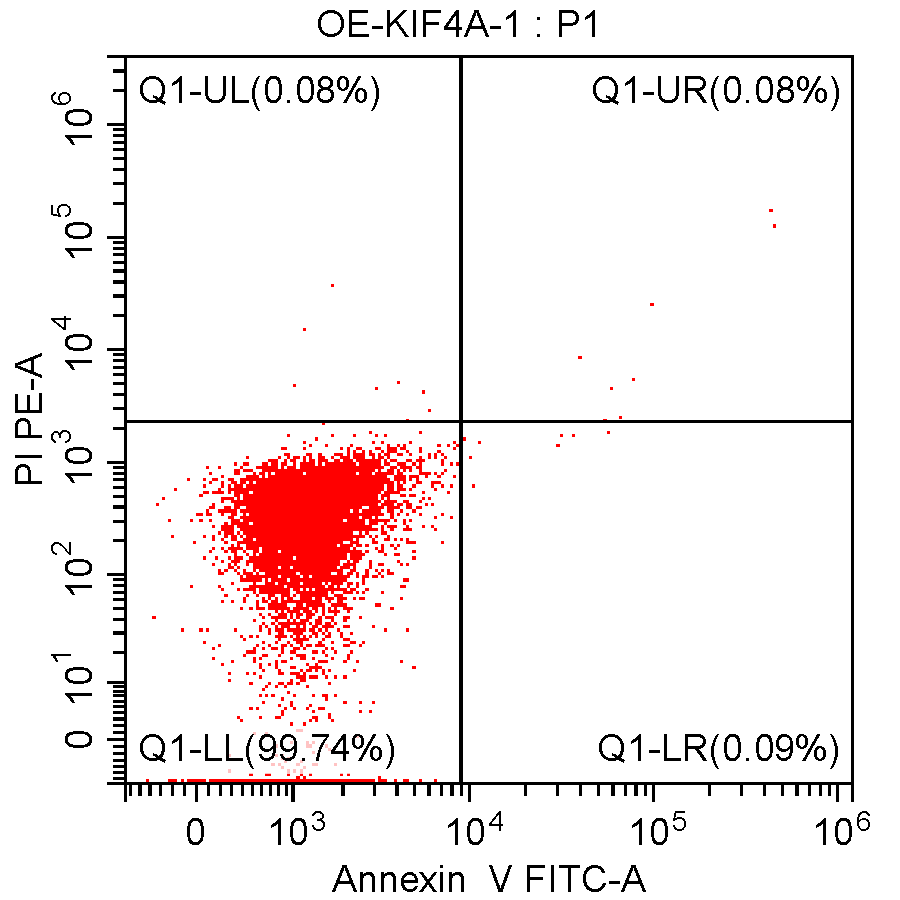

Supplement: Supplemental Information 11 [file peerj-09-11455-s011.zip › fig5A-Apoptosis/U20S/OE-KIF4A-1_Plot2.bmp]

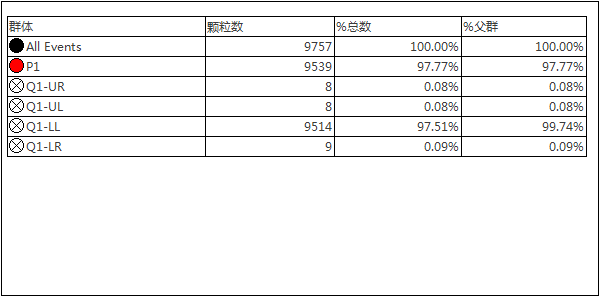

Supplement: Supplemental Information 11 [file peerj-09-11455-s011.zip › fig5A-Apoptosis/U20S/OE-KIF4A-1_Statistics1.bmp]

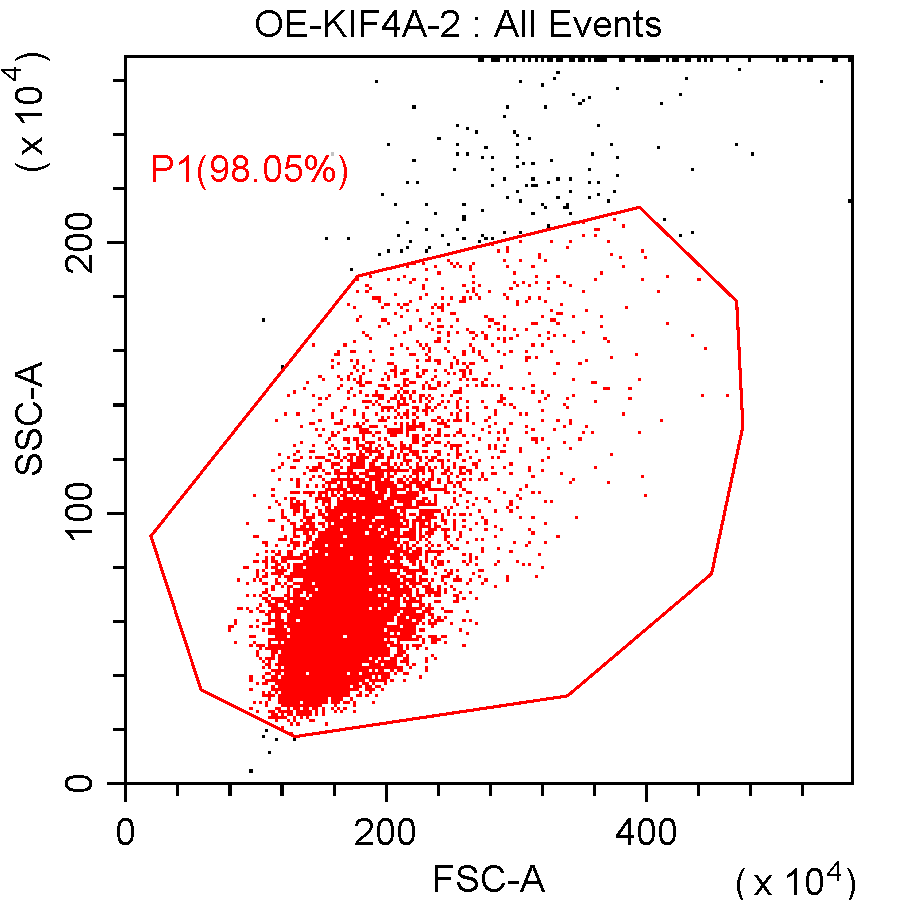

Supplement: Supplemental Information 11 [file peerj-09-11455-s011.zip › fig5A-Apoptosis/U20S/OE-KIF4A-2_Plot1.bmp]

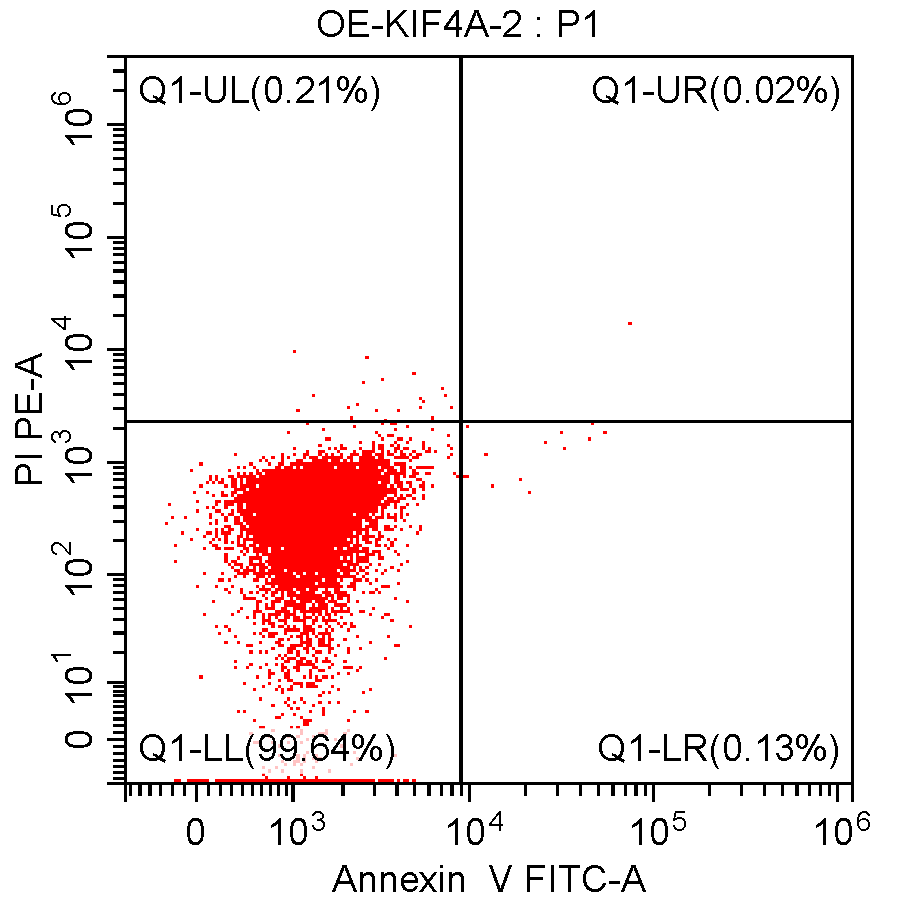

Supplement: Supplemental Information 11 [file peerj-09-11455-s011.zip › fig5A-Apoptosis/U20S/OE-KIF4A-2_Plot2.bmp]

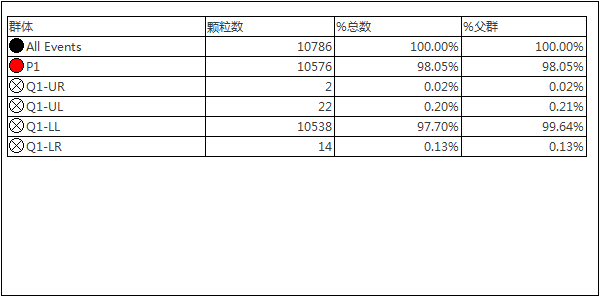

Supplement: Supplemental Information 11 [file peerj-09-11455-s011.zip › fig5A-Apoptosis/U20S/OE-KIF4A-2_Statistics1.bmp]

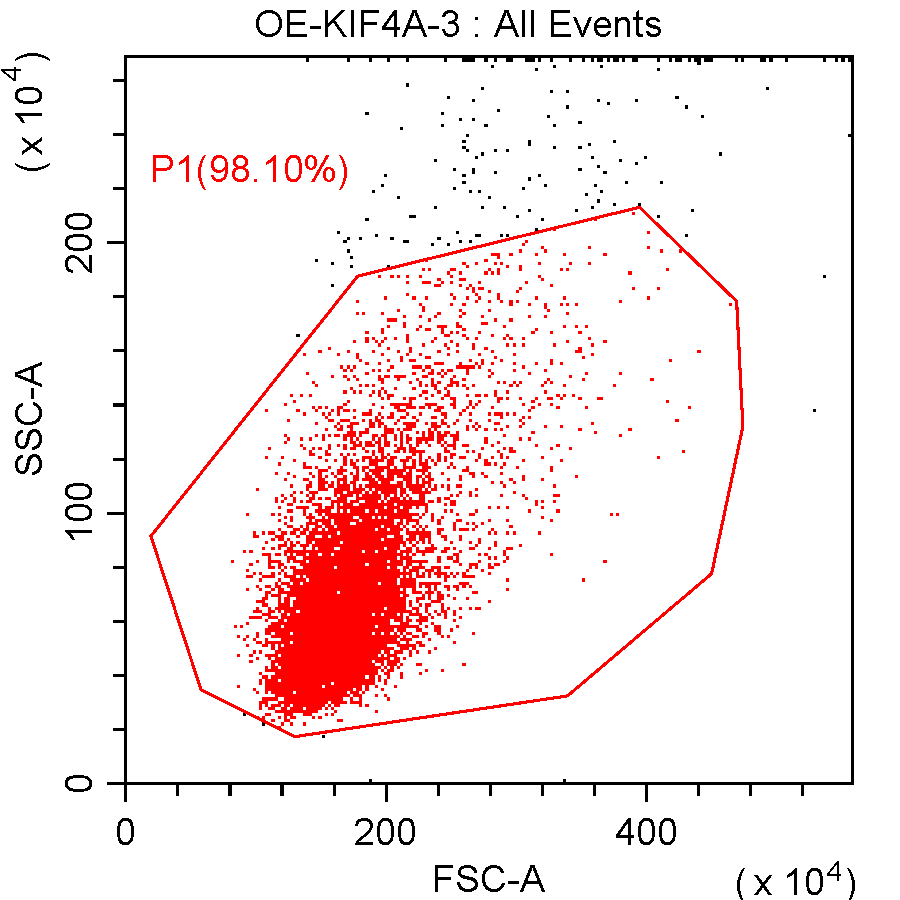

Supplement: Supplemental Information 11 [file peerj-09-11455-s011.zip › fig5A-Apoptosis/U20S/OE-KIF4A-3_Plot1.bmp]

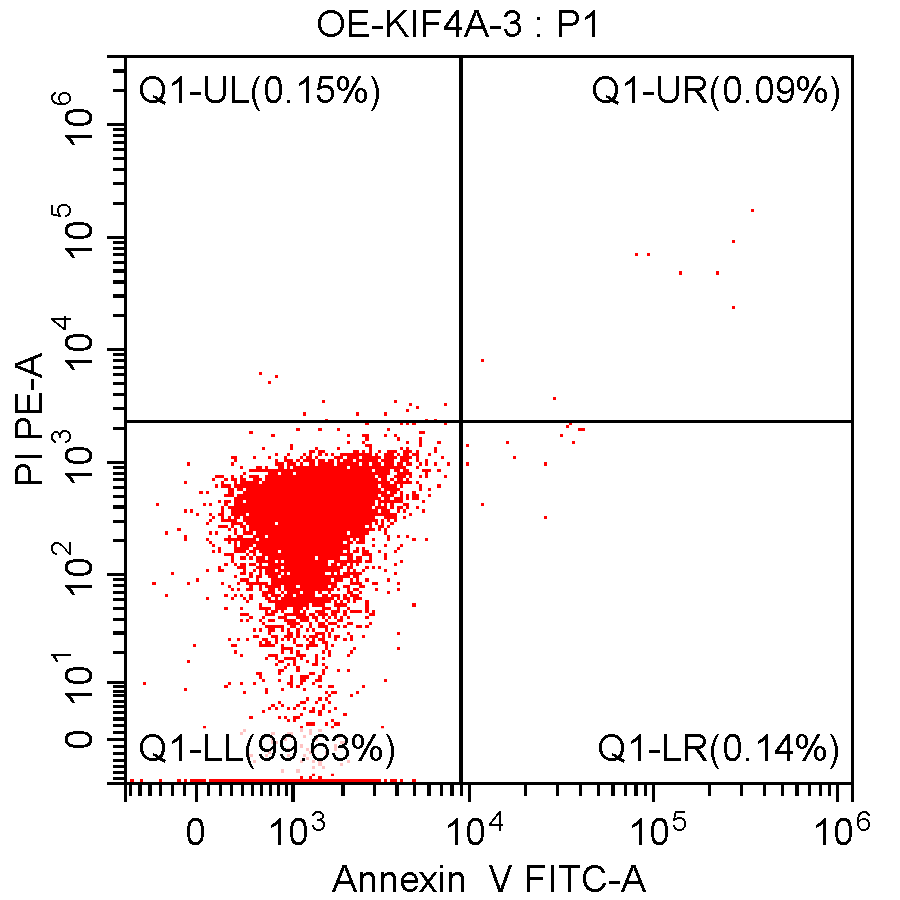

Supplement: Supplemental Information 11 [file peerj-09-11455-s011.zip › fig5A-Apoptosis/U20S/OE-KIF4A-3_Plot2.bmp]

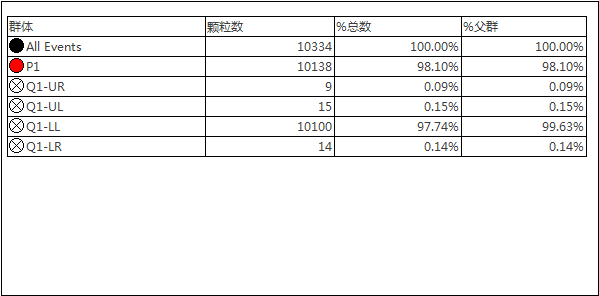

Supplement: Supplemental Information 11 [file peerj-09-11455-s011.zip › fig5A-Apoptosis/U20S/OE-KIF4A-3_Statistics1.bmp]

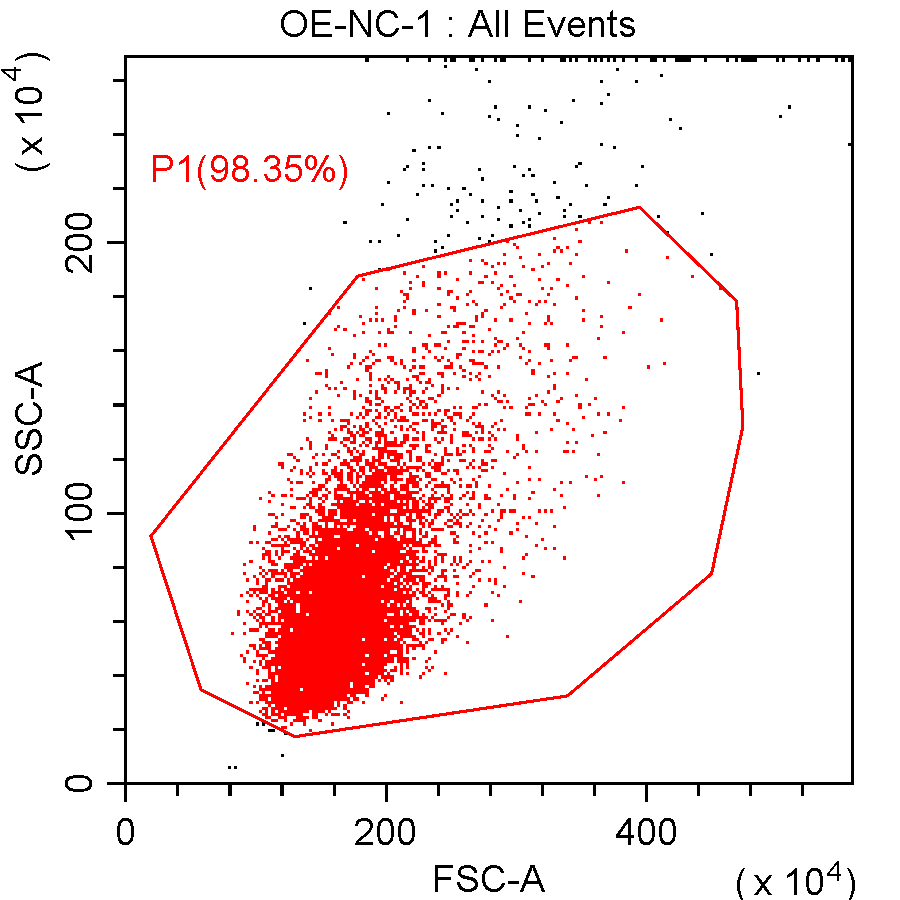

Supplement: Supplemental Information 11 [file peerj-09-11455-s011.zip › fig5A-Apoptosis/U20S/OE-NC-1_Plot1.bmp]

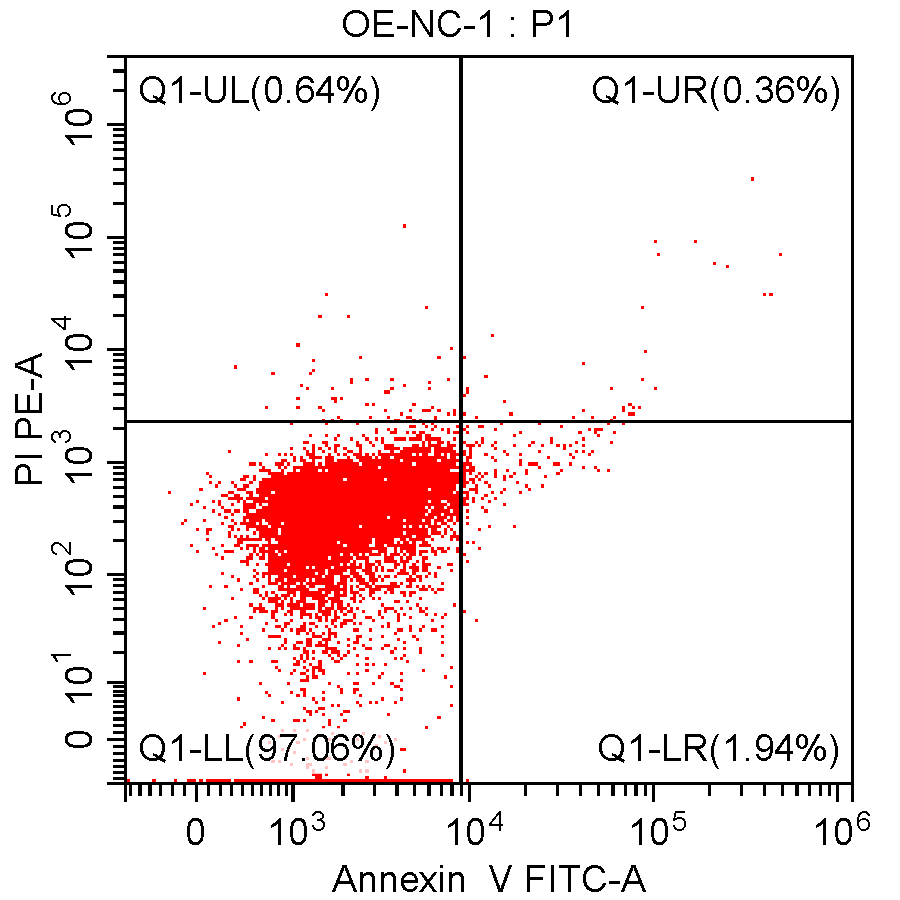

Supplement: Supplemental Information 11 [file peerj-09-11455-s011.zip › fig5A-Apoptosis/U20S/OE-NC-1_Plot2.bmp]

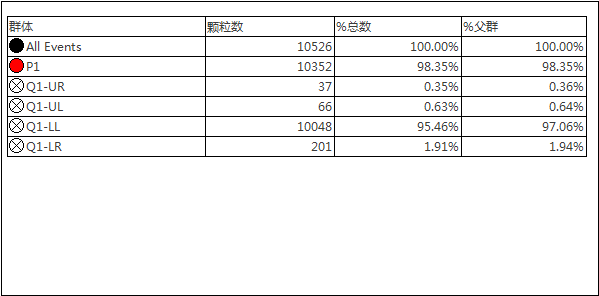

Supplement: Supplemental Information 11 [file peerj-09-11455-s011.zip › fig5A-Apoptosis/U20S/OE-NC-1_Statistics1.bmp]

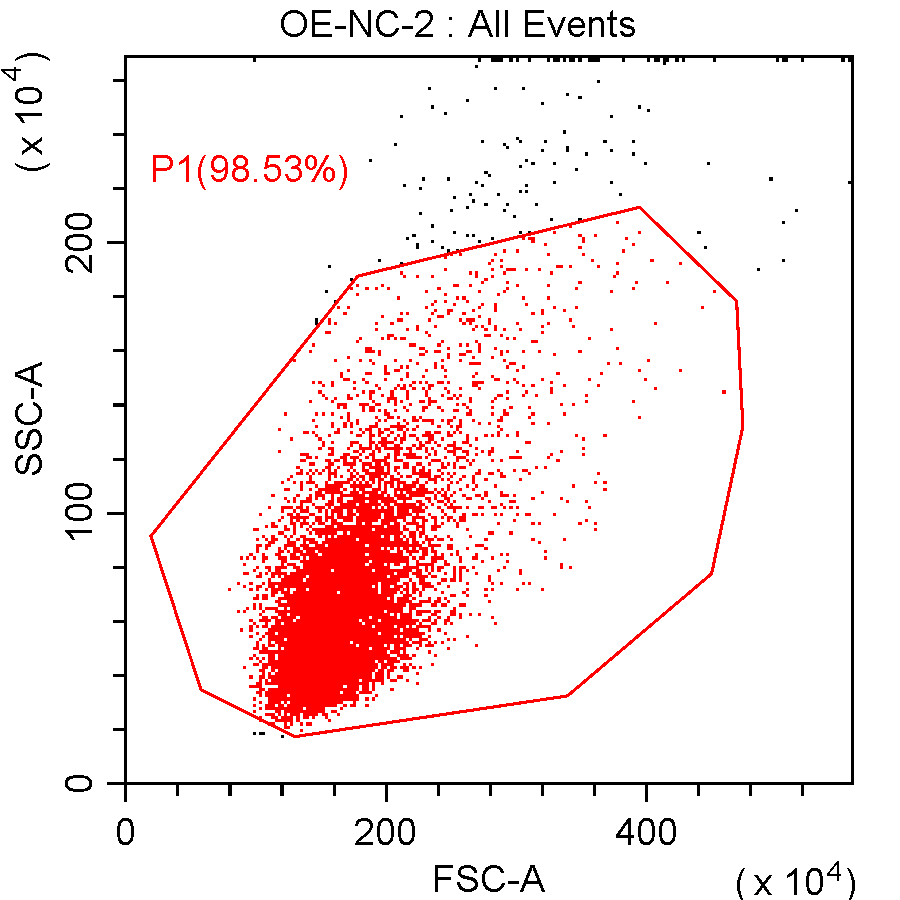

Supplement: Supplemental Information 11 [file peerj-09-11455-s011.zip › fig5A-Apoptosis/U20S/OE-NC-2_Plot1.bmp]

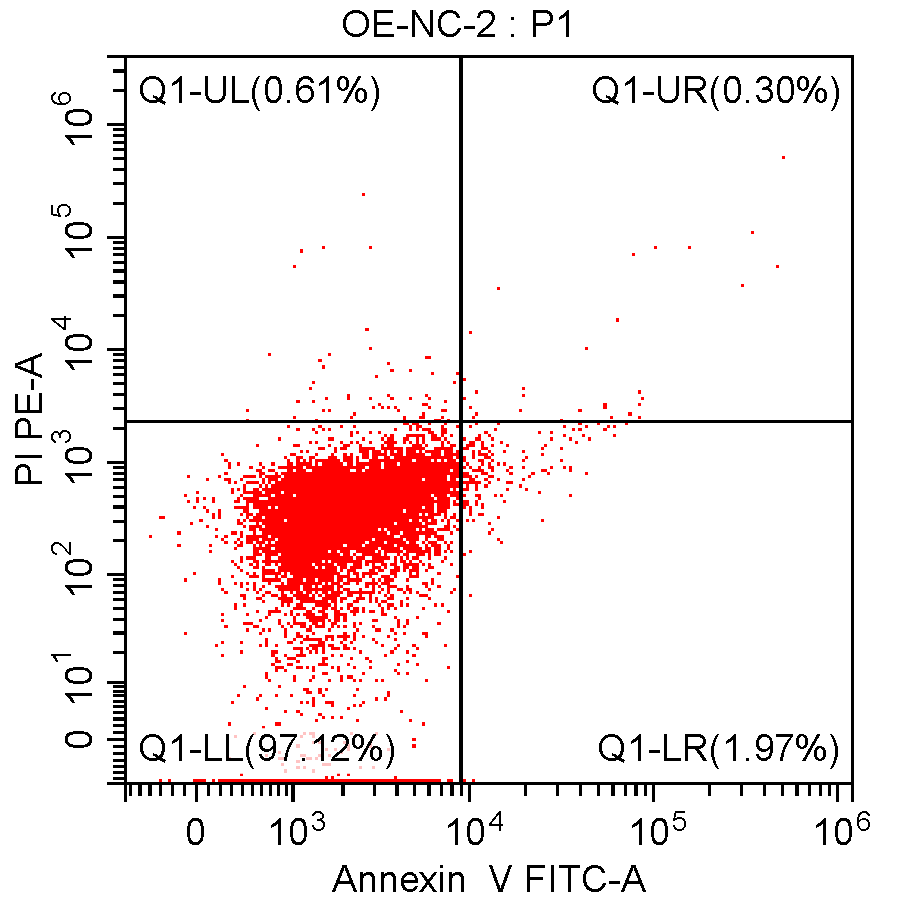

Supplement: Supplemental Information 11 [file peerj-09-11455-s011.zip › fig5A-Apoptosis/U20S/OE-NC-2_Plot2.bmp]

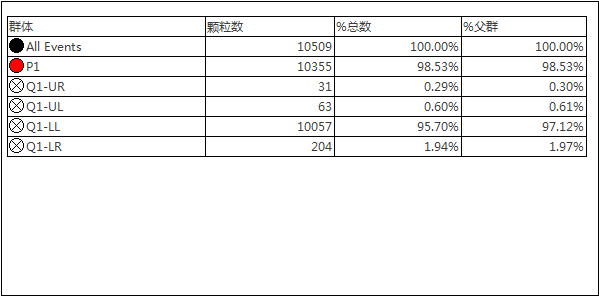

Supplement: Supplemental Information 11 [file peerj-09-11455-s011.zip › fig5A-Apoptosis/U20S/OE-NC-2_Statistics1.bmp]

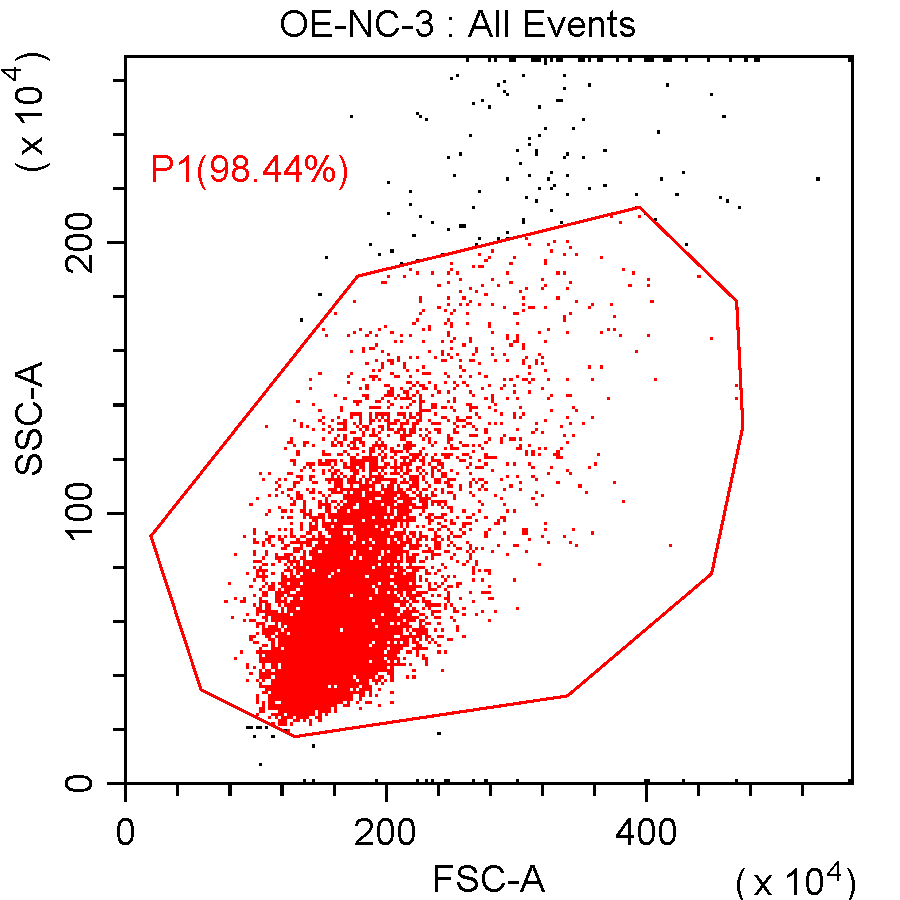

Supplement: Supplemental Information 11 [file peerj-09-11455-s011.zip › fig5A-Apoptosis/U20S/OE-NC-3_Plot1.bmp]

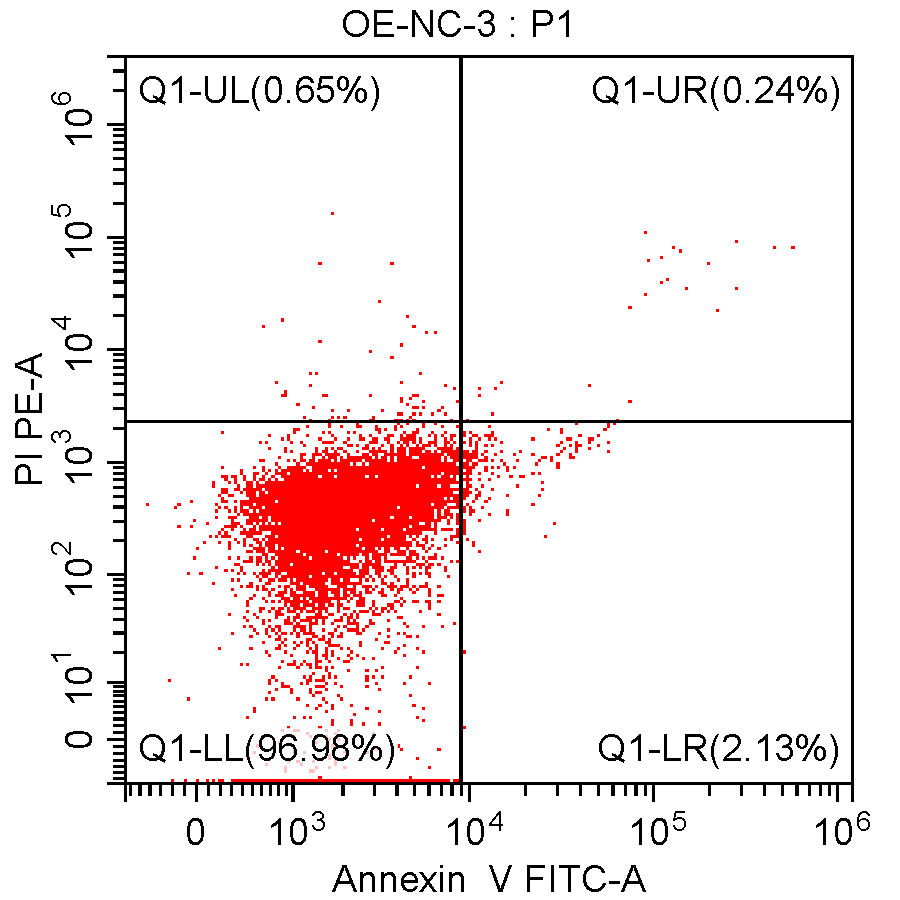

Supplement: Supplemental Information 11 [file peerj-09-11455-s011.zip › fig5A-Apoptosis/U20S/OE-NC-3_Plot2.bmp]

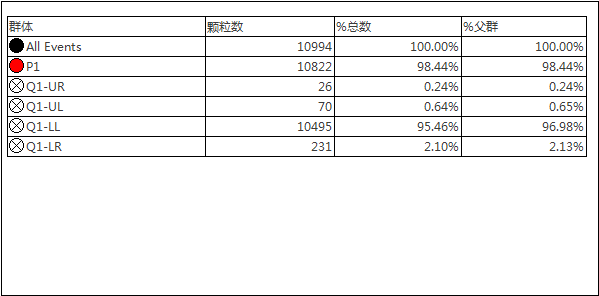

Supplement: Supplemental Information 11 [file peerj-09-11455-s011.zip › fig5A-Apoptosis/U20S/OE-NC-3_Statistics1.bmp]

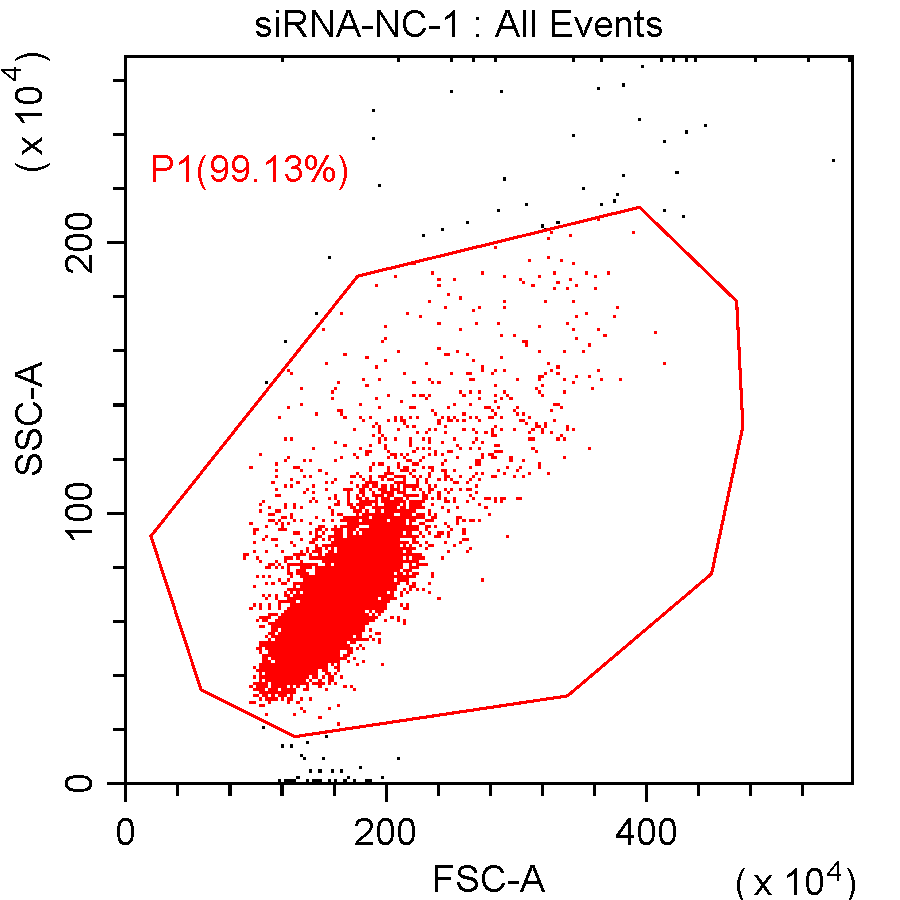

Supplement: Supplemental Information 11 [file peerj-09-11455-s011.zip › fig5A-Apoptosis/U20S/siRNA-NC-1_Plot1.bmp]

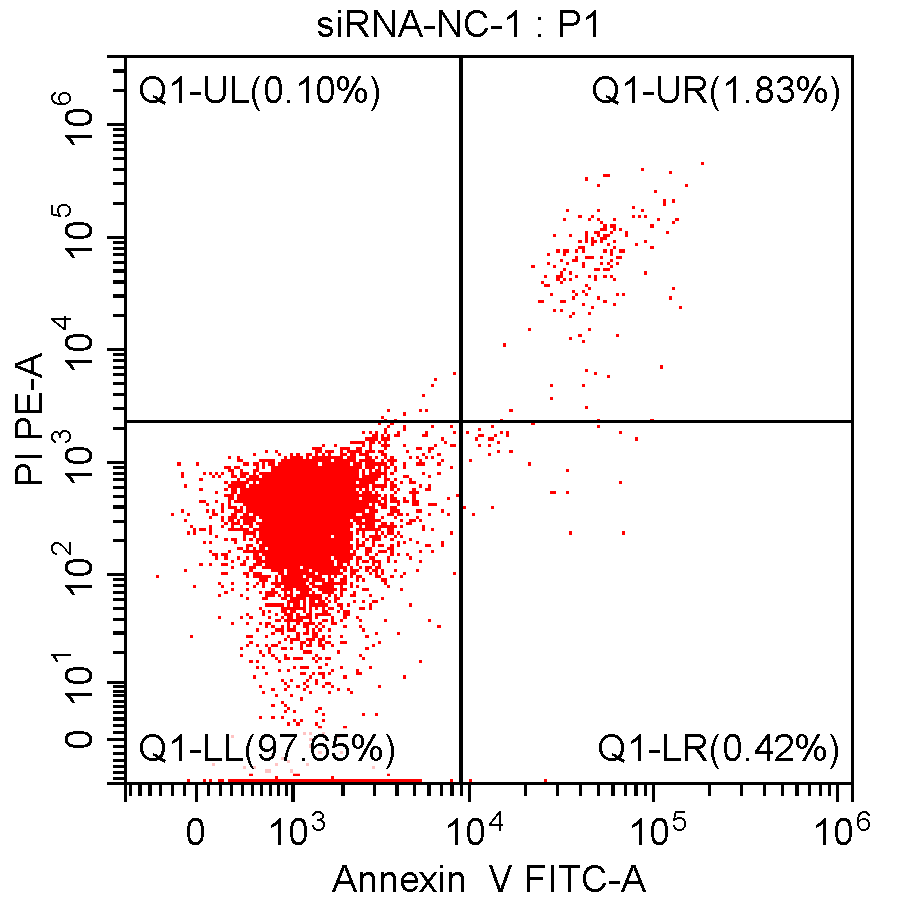

Supplement: Supplemental Information 11 [file peerj-09-11455-s011.zip › fig5A-Apoptosis/U20S/siRNA-NC-1_Plot2.bmp]

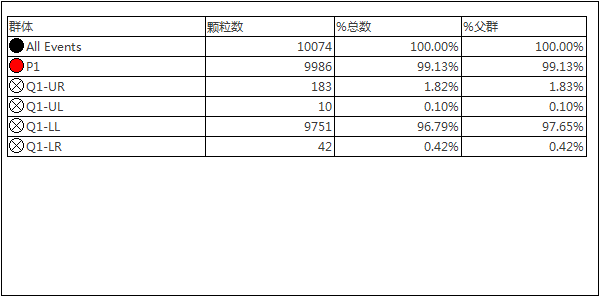

Supplement: Supplemental Information 11 [file peerj-09-11455-s011.zip › fig5A-Apoptosis/U20S/siRNA-NC-1_Statistics1.bmp]

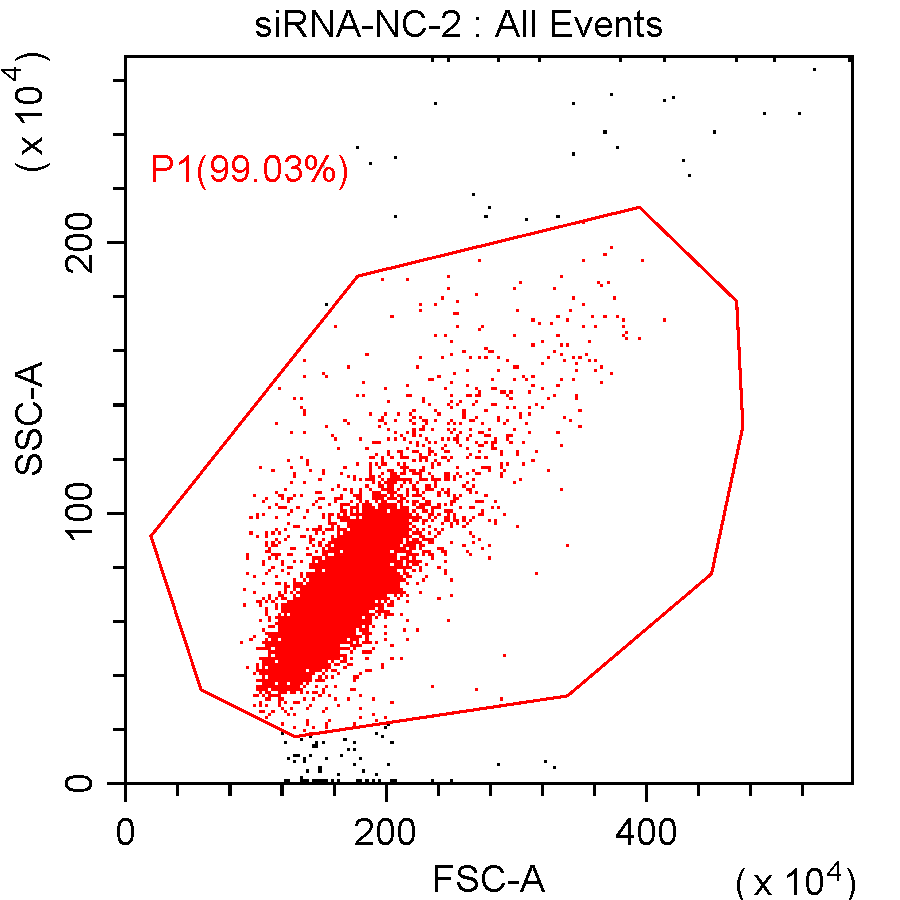

Supplement: Supplemental Information 11 [file peerj-09-11455-s011.zip › fig5A-Apoptosis/U20S/siRNA-NC-2_Plot1.bmp]

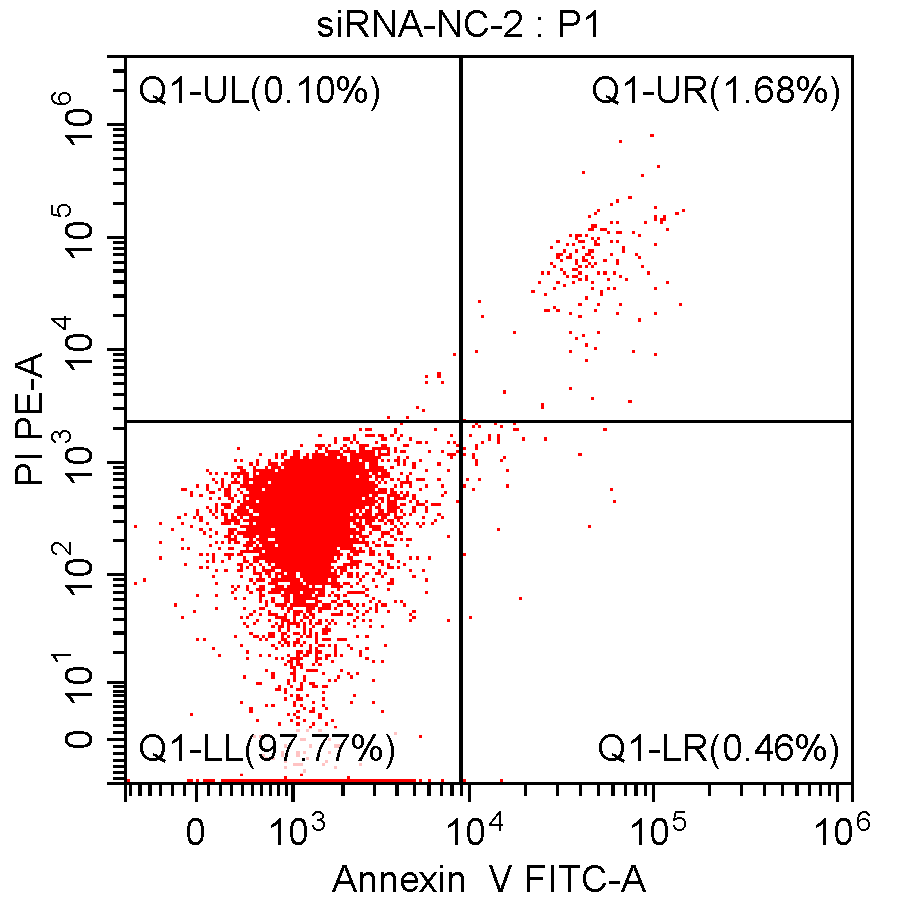

Supplement: Supplemental Information 11 [file peerj-09-11455-s011.zip › fig5A-Apoptosis/U20S/siRNA-NC-2_Plot2.bmp]

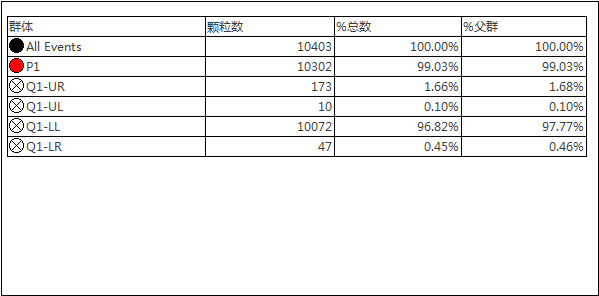

Supplement: Supplemental Information 11 [file peerj-09-11455-s011.zip › fig5A-Apoptosis/U20S/siRNA-NC-2_Statistics1.bmp]

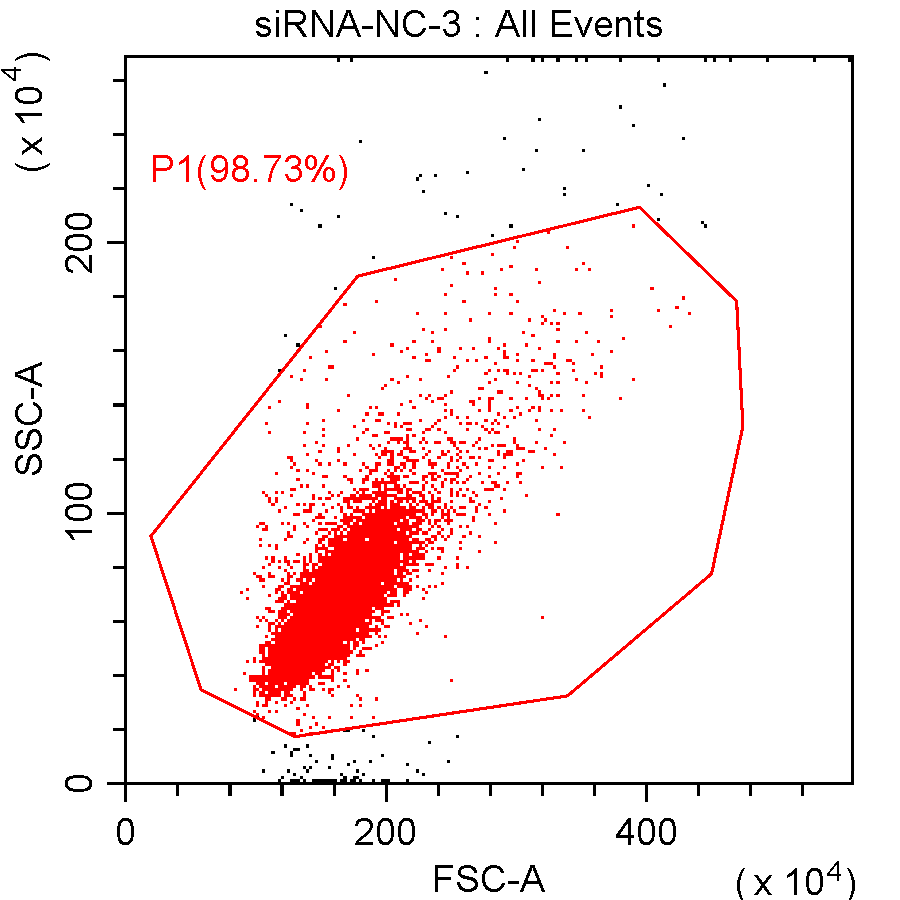

Supplement: Supplemental Information 11 [file peerj-09-11455-s011.zip › fig5A-Apoptosis/U20S/siRNA-NC-3_Plot1.bmp]

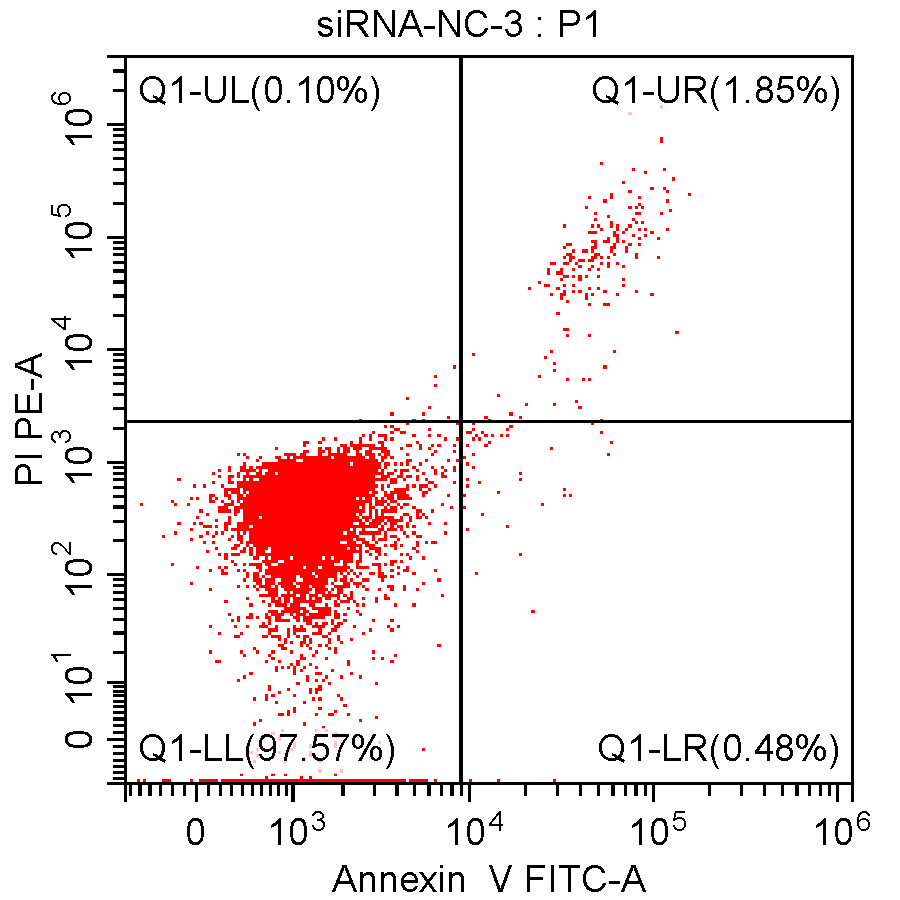

Supplement: Supplemental Information 11 [file peerj-09-11455-s011.zip › fig5A-Apoptosis/U20S/siRNA-NC-3_Plot2.bmp]

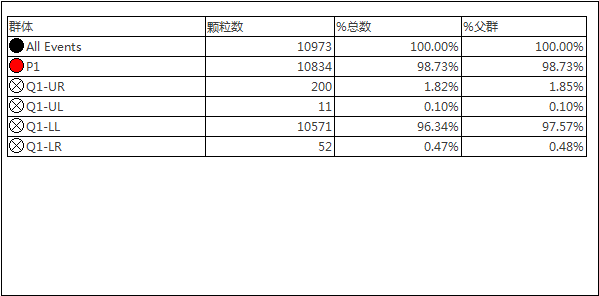

Supplement: Supplemental Information 11 [file peerj-09-11455-s011.zip › fig5A-Apoptosis/U20S/siRNA-NC-3_Statistics1.bmp]

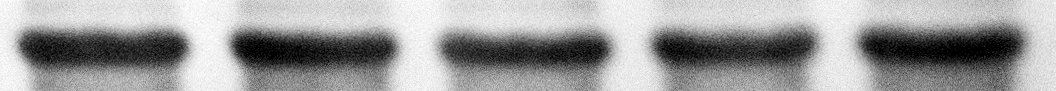

Supplement: Supplemental Information 14 [file peerj-09-11455-s014.zip › fig5C -WB/Mg63/actin-1.tif]

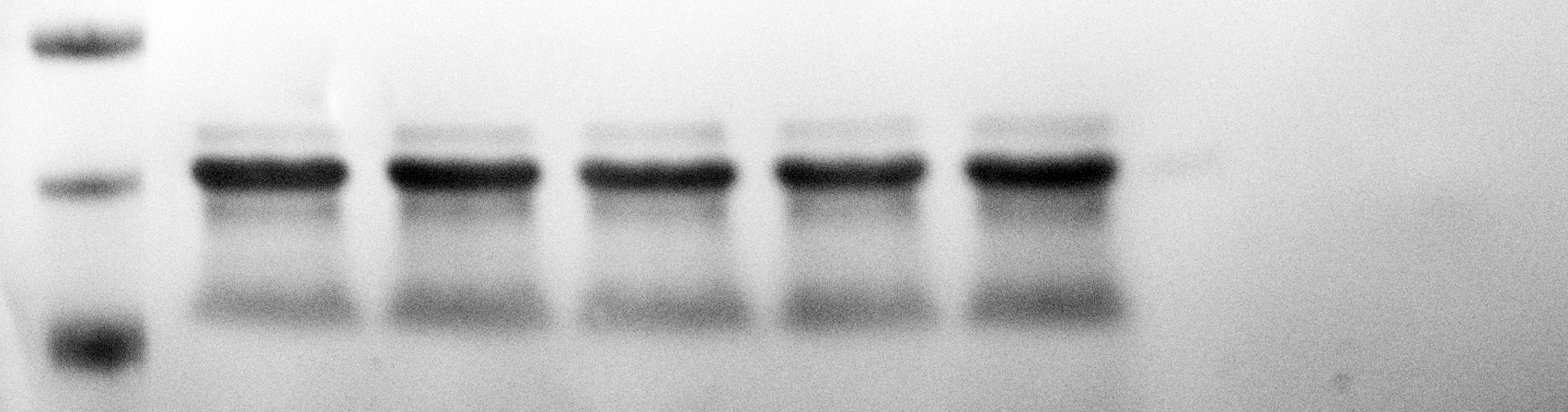

Supplement: Supplemental Information 14 [file peerj-09-11455-s014.zip › fig5C -WB/Mg63/actin.tif]

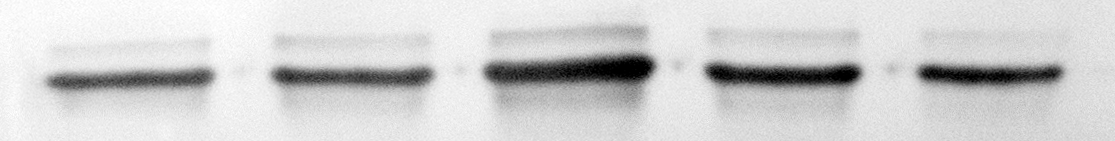

Supplement: Supplemental Information 14 [file peerj-09-11455-s014.zip › fig5C -WB/Mg63/bax-1.tif]

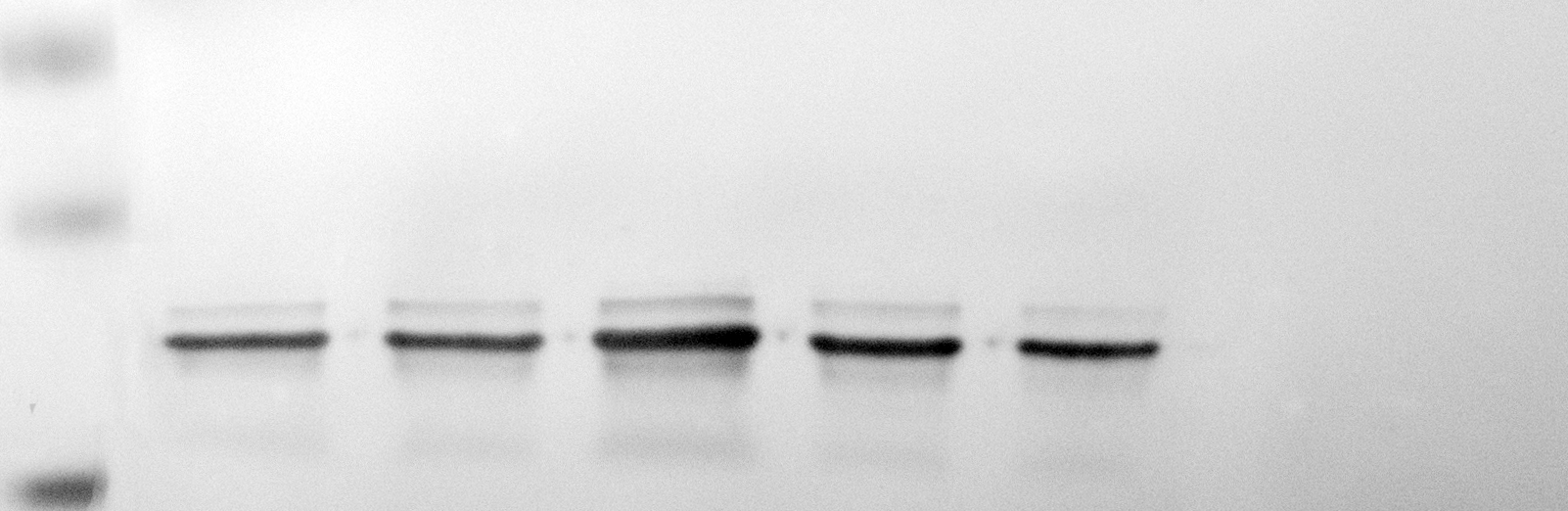

Supplement: Supplemental Information 14 [file peerj-09-11455-s014.zip › fig5C -WB/Mg63/bax.tif]

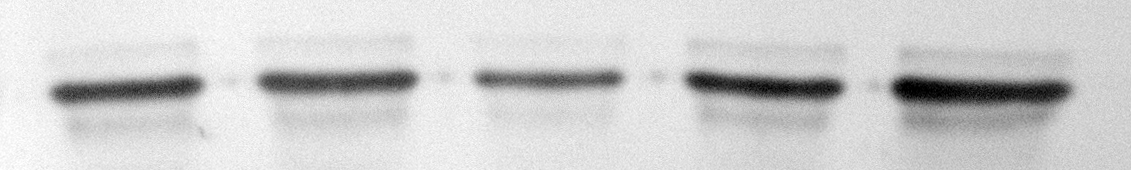

Supplement: Supplemental Information 14 [file peerj-09-11455-s014.zip › fig5C -WB/Mg63/bcl2-1.tif]

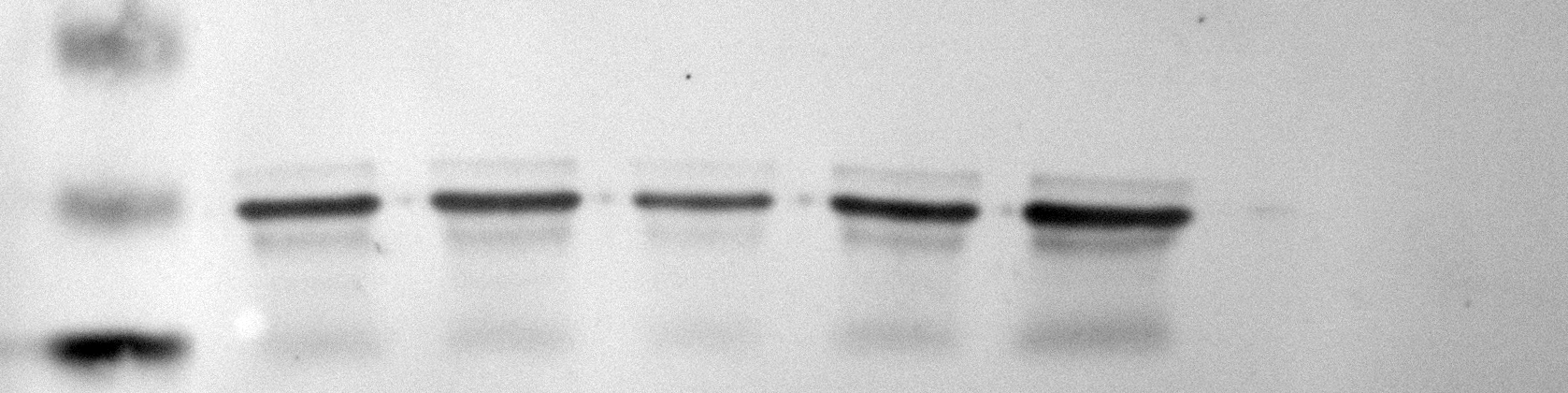

Supplement: Supplemental Information 14 [file peerj-09-11455-s014.zip › fig5C -WB/Mg63/bcl2.tif]

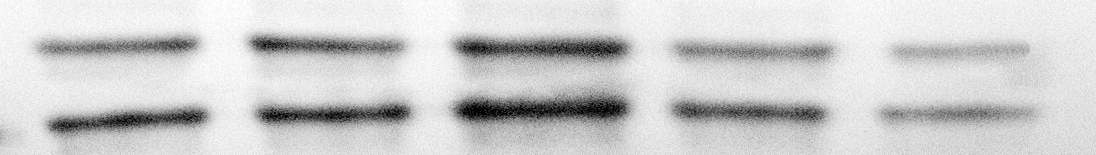

Supplement: Supplemental Information 14 [file peerj-09-11455-s014.zip › fig5C -WB/Mg63/caspase3-1.tif]

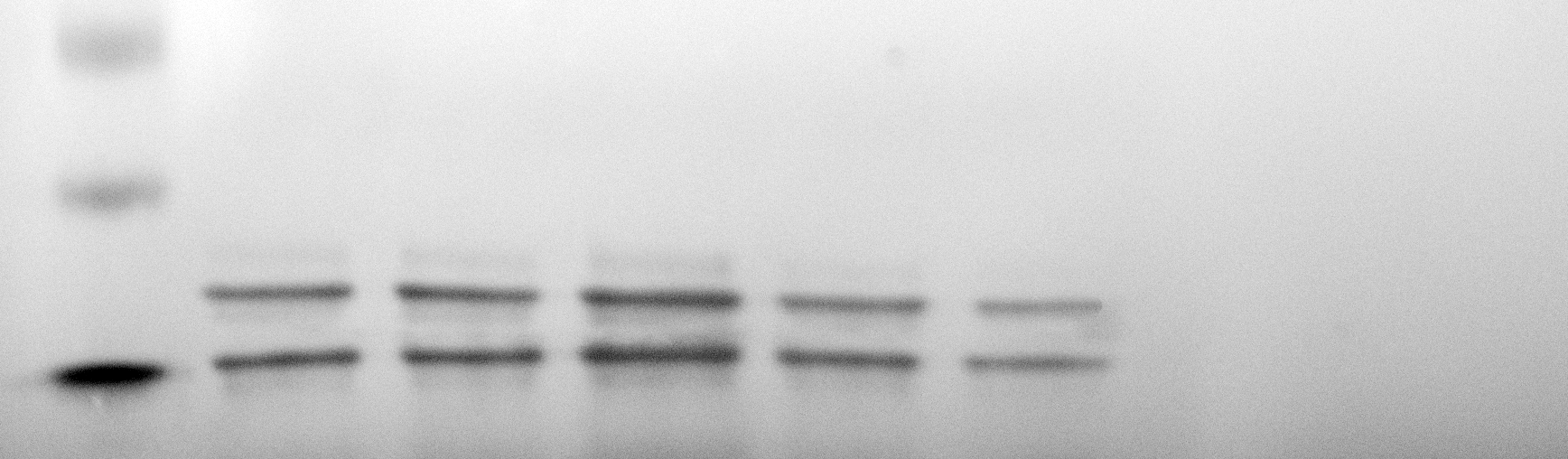

Supplement: Supplemental Information 14 [file peerj-09-11455-s014.zip › fig5C -WB/Mg63/caspase3.tif]

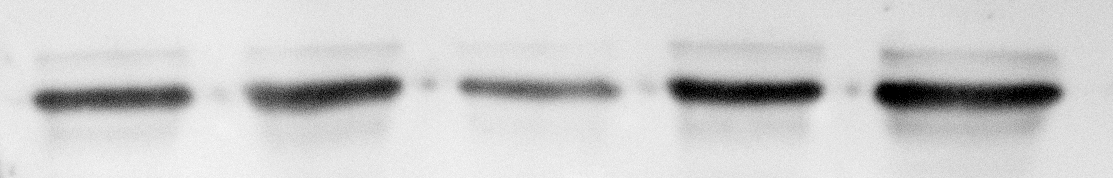

Supplement: Supplemental Information 14 [file peerj-09-11455-s014.zip › fig5C -WB/Mg63/p-β-catenin-1.tif]

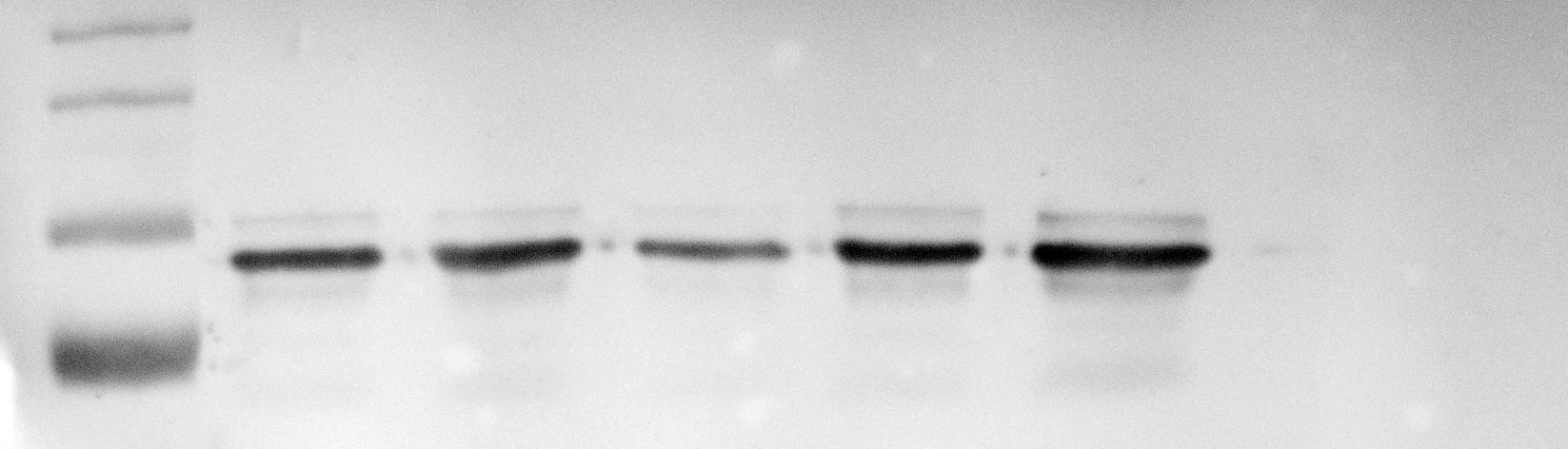

Supplement: Supplemental Information 14 [file peerj-09-11455-s014.zip › fig5C -WB/Mg63/p-β-catenin.tif]

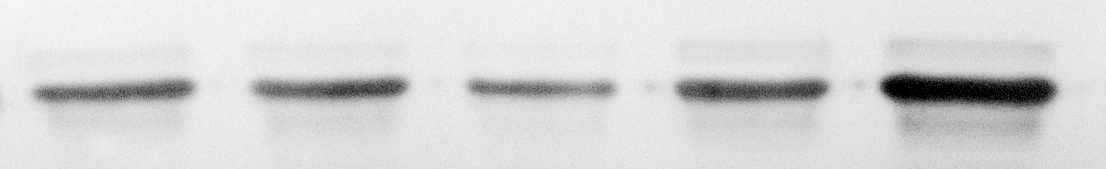

Supplement: Supplemental Information 14 [file peerj-09-11455-s014.zip › fig5C -WB/Mg63/wnt-1.tif]

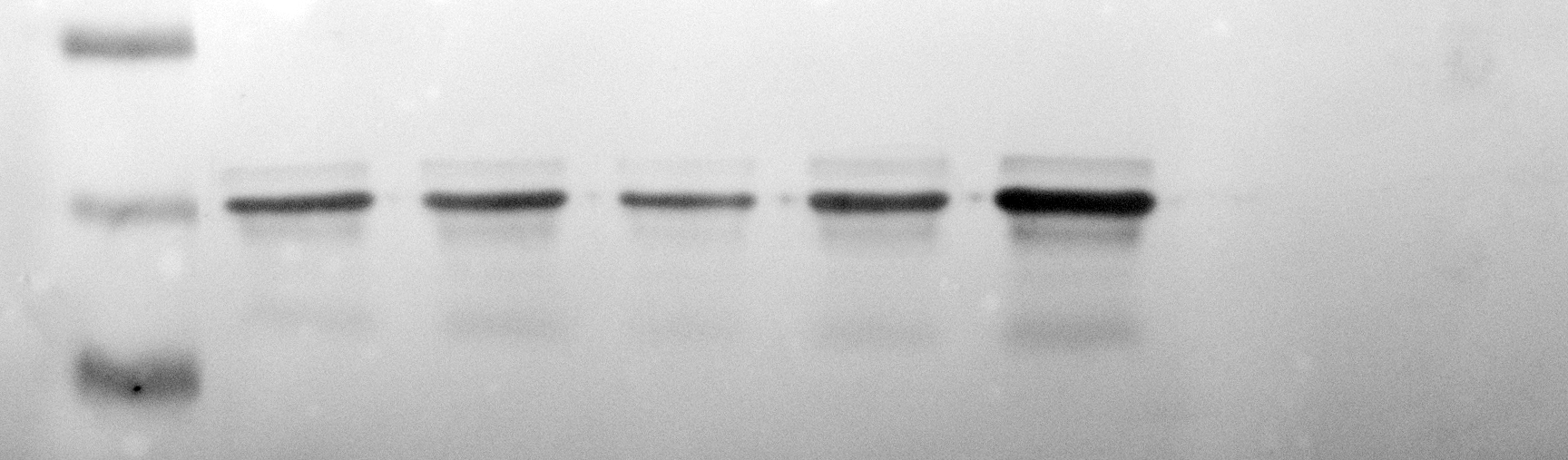

Supplement: Supplemental Information 14 [file peerj-09-11455-s014.zip › fig5C -WB/Mg63/wnt.tif]

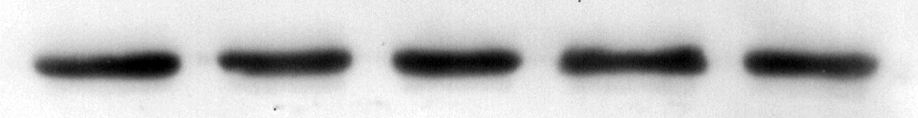

Supplement: Supplemental Information 14 [file peerj-09-11455-s014.zip › fig5C -WB/Mg63/β-catenin-1.tif]

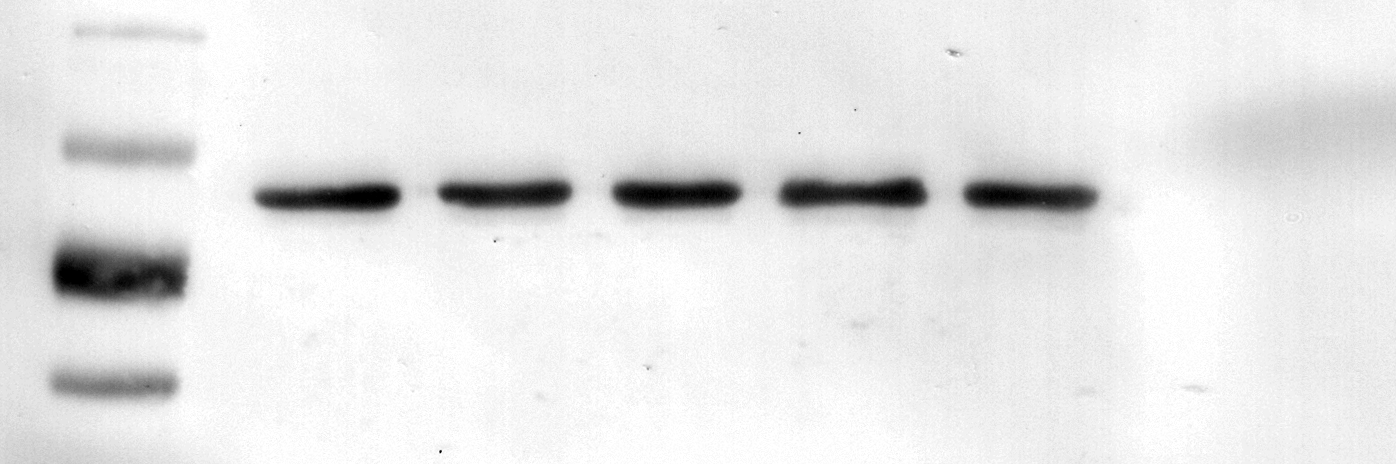

Supplement: Supplemental Information 14 [file peerj-09-11455-s014.zip › fig5C -WB/Mg63/β-catenin.tif]

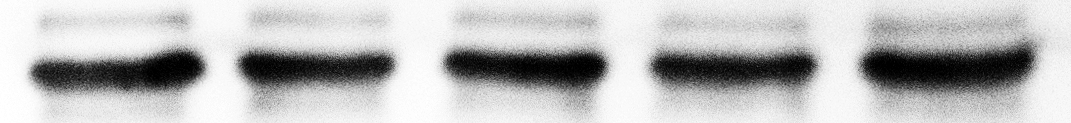

Supplement: Supplemental Information 14 [file peerj-09-11455-s014.zip › fig5C -WB/U2OS/actin-1.tif]

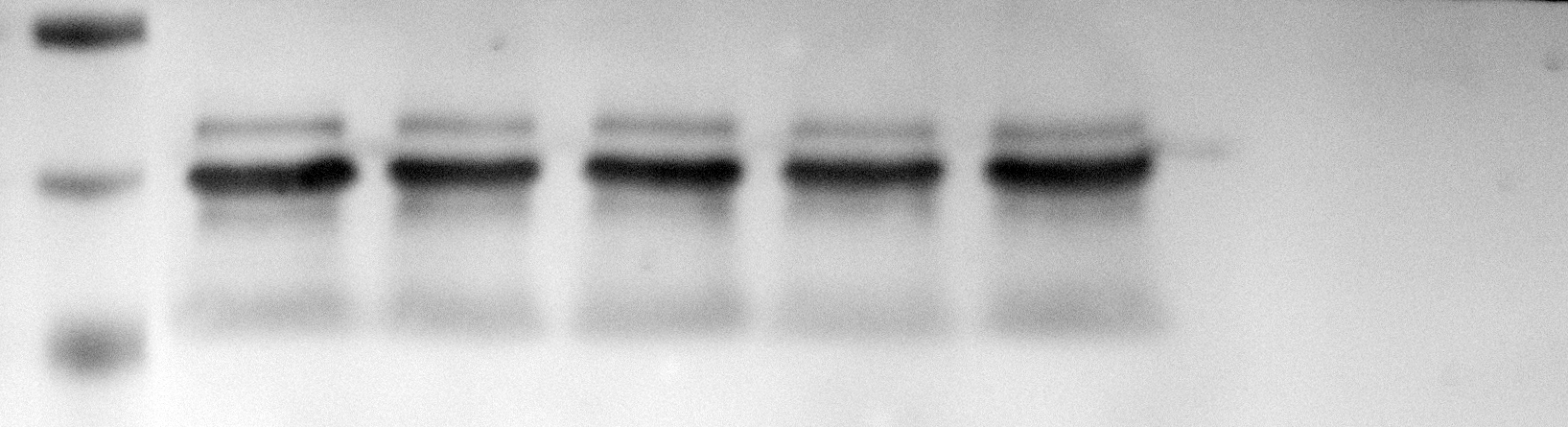

Supplement: Supplemental Information 14 [file peerj-09-11455-s014.zip › fig5C -WB/U2OS/actin.tif]

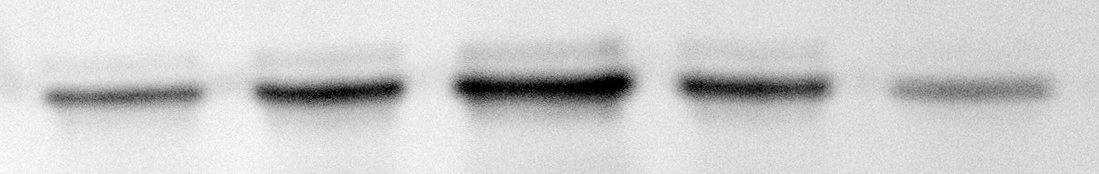

Supplement: Supplemental Information 14 [file peerj-09-11455-s014.zip › fig5C -WB/U2OS/bax-1.tif]

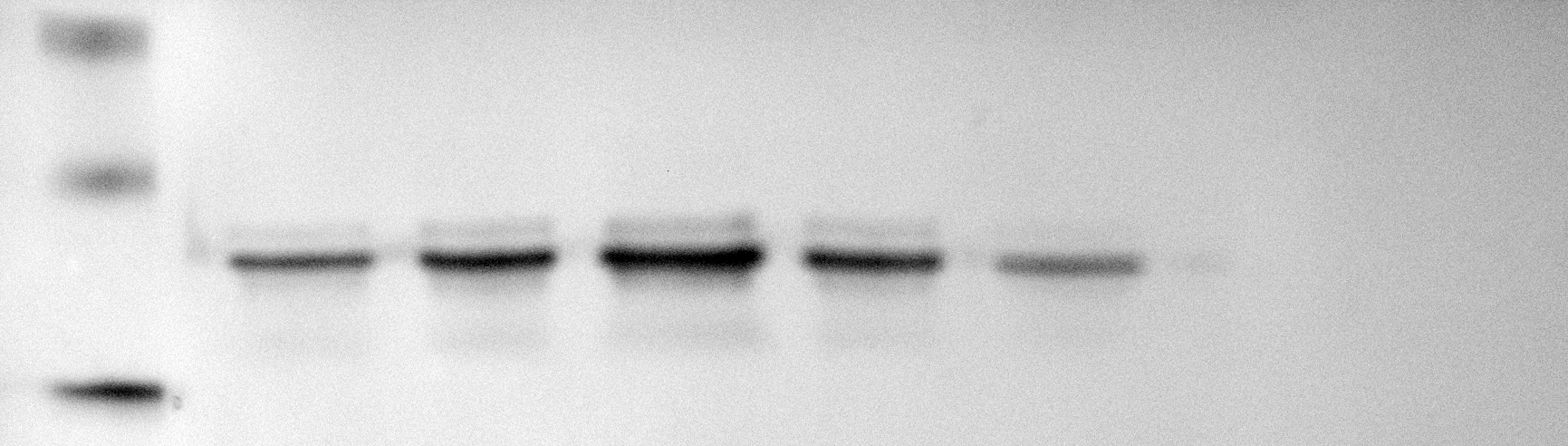

Supplement: Supplemental Information 14 [file peerj-09-11455-s014.zip › fig5C -WB/U2OS/bax.tif]

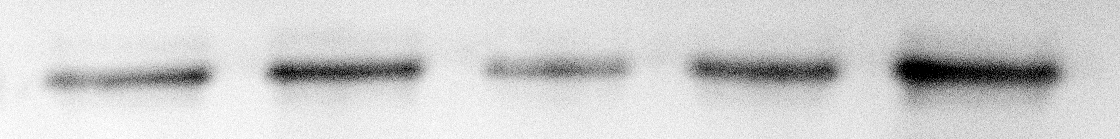

Supplement: Supplemental Information 14 [file peerj-09-11455-s014.zip › fig5C -WB/U2OS/bcl2-1.tif]

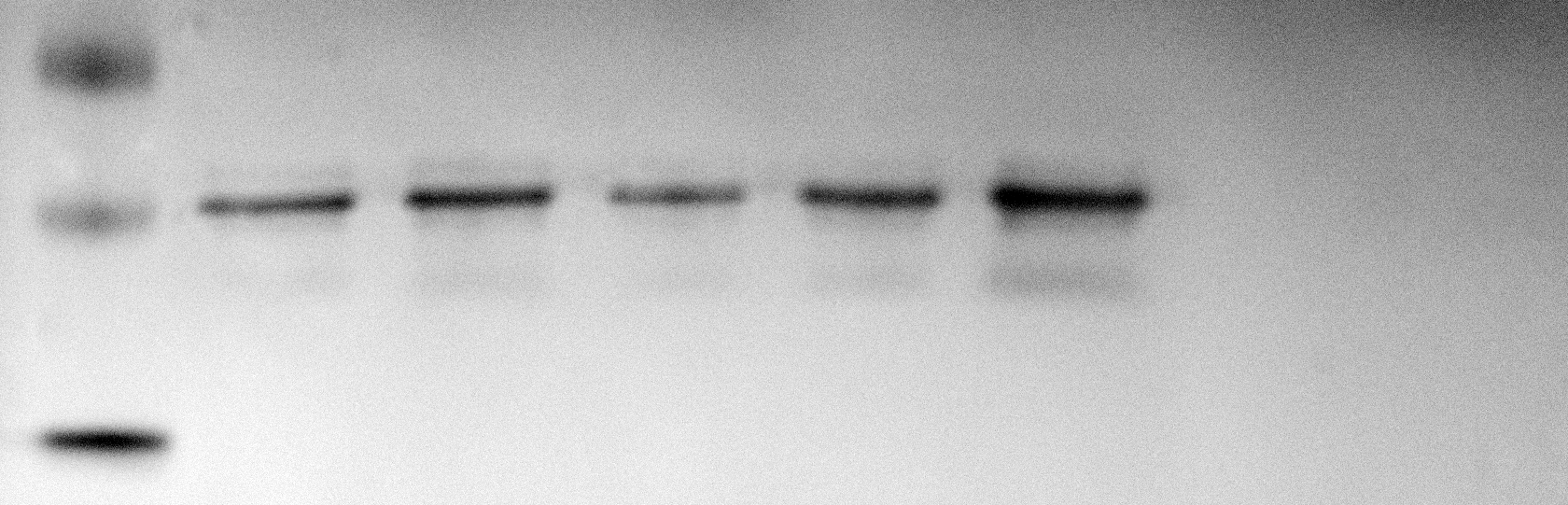

Supplement: Supplemental Information 14 [file peerj-09-11455-s014.zip › fig5C -WB/U2OS/bcl2.tif]

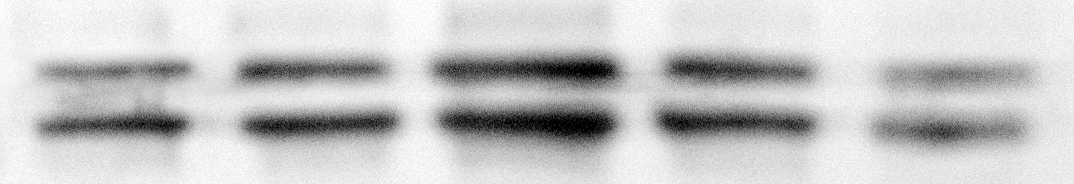

Supplement: Supplemental Information 14 [file peerj-09-11455-s014.zip › fig5C -WB/U2OS/caspase3-1.tif]

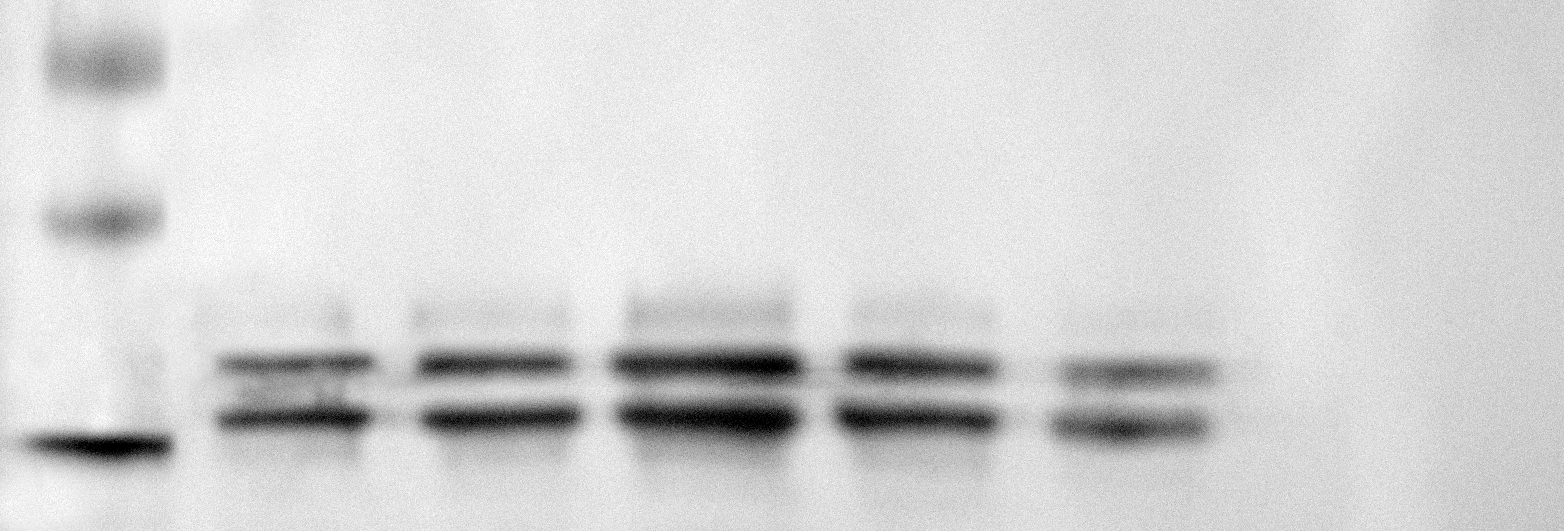

Supplement: Supplemental Information 14 [file peerj-09-11455-s014.zip › fig5C -WB/U2OS/caspase3.tif]

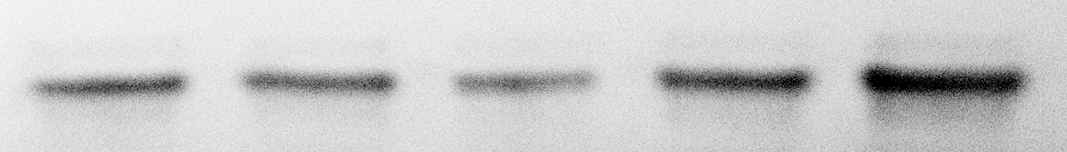

Supplement: Supplemental Information 14 [file peerj-09-11455-s014.zip › fig5C -WB/U2OS/p-β-catenin-1.tif]

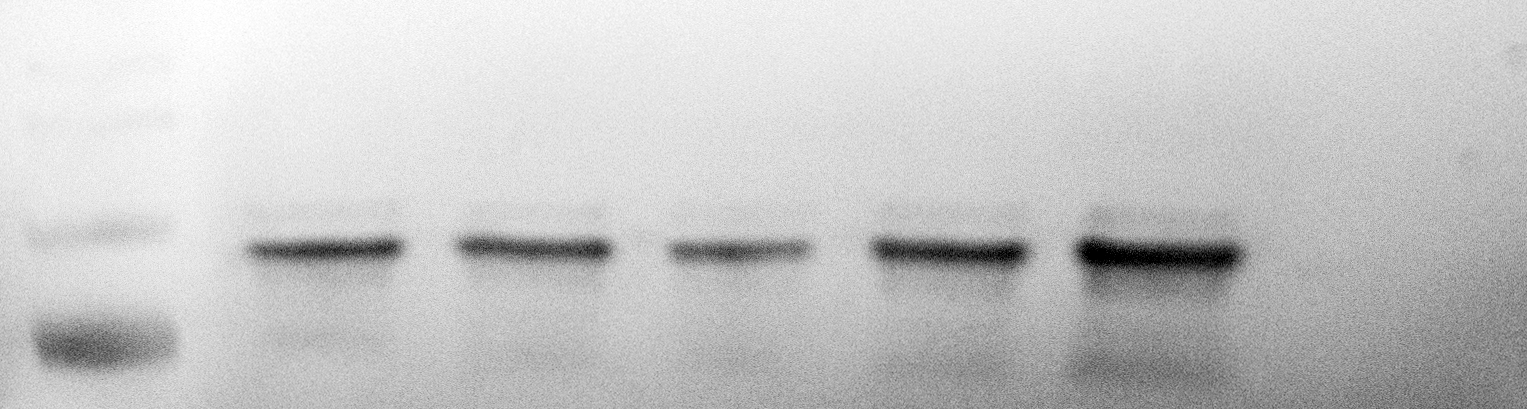

Supplement: Supplemental Information 14 [file peerj-09-11455-s014.zip › fig5C -WB/U2OS/p-β-catenin.tif]

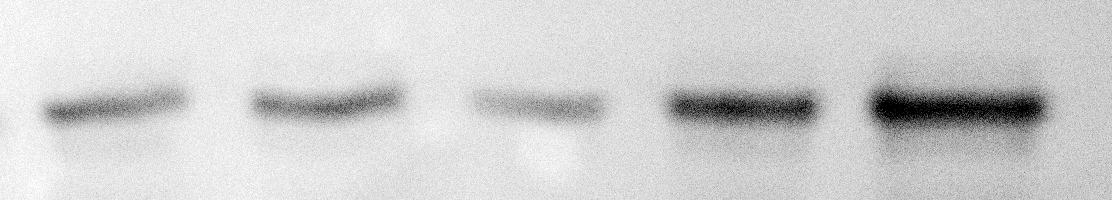

Supplement: Supplemental Information 14 [file peerj-09-11455-s014.zip › fig5C -WB/U2OS/wnt3a-1.tif]

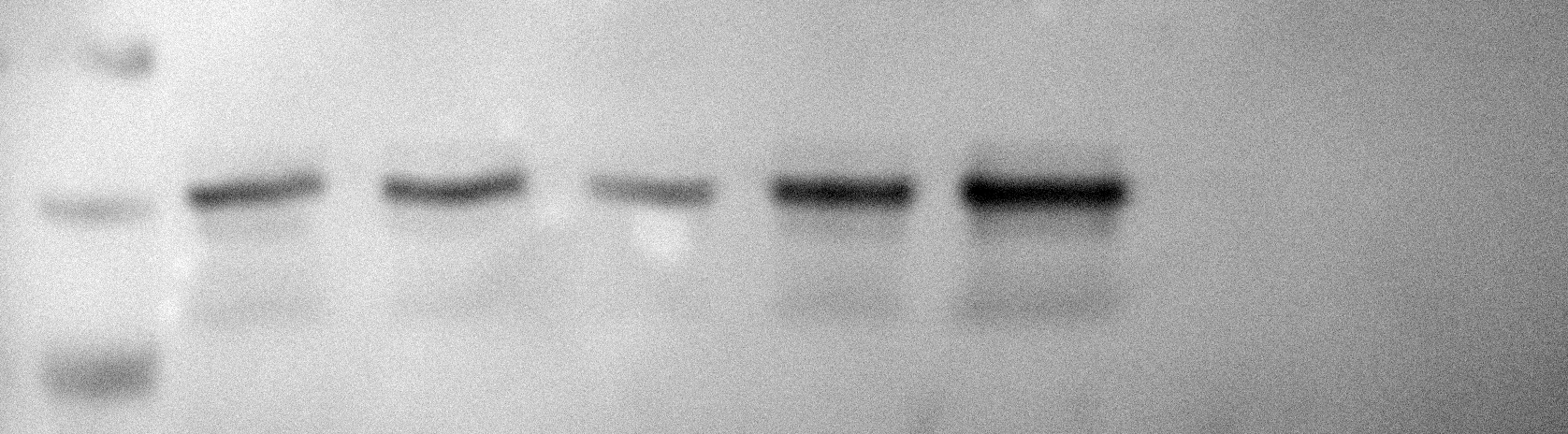

Supplement: Supplemental Information 14 [file peerj-09-11455-s014.zip › fig5C -WB/U2OS/wnt3a.tif]

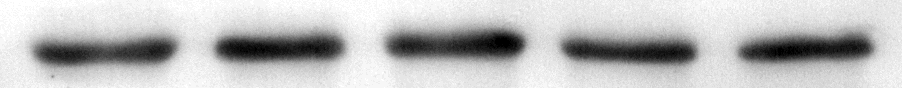

Supplement: Supplemental Information 14 [file peerj-09-11455-s014.zip › fig5C -WB/U2OS/β-catenin-1.tif]

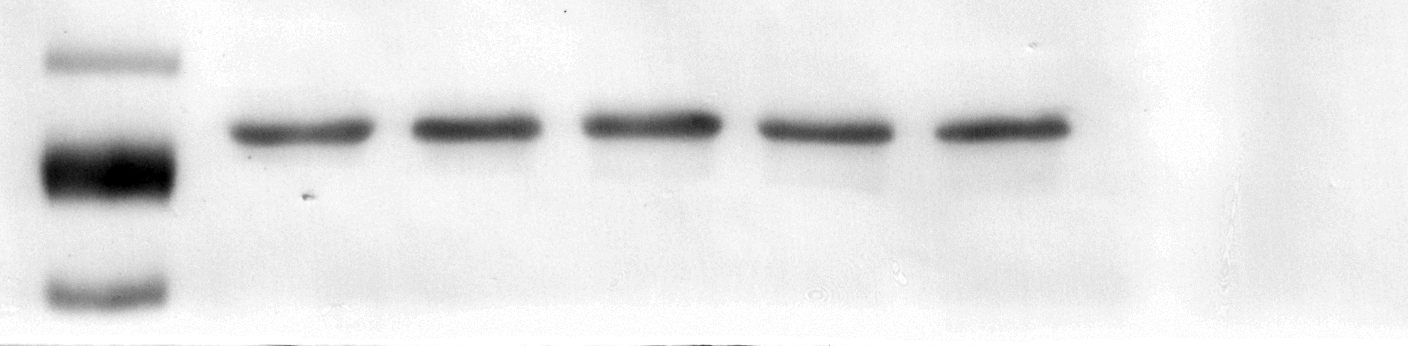

Supplement: Supplemental Information 14 [file peerj-09-11455-s014.zip › fig5C -WB/U2OS/β-catenin.tif]
