# Supplementary material for: Identification of KIF4A as a pan-cancer diagnostic and prognostic biomarker via bioinformatics analysis and validation in osteosarcoma cell lines
Source: PeerJ. 2021 May 21;9:e11455. doi: 10.7717/peerj.11455 (PMC8142929; doi:10.7717/peerj.11455)
Supplement: Supplemental Information 14 [file peerj-09-11455-s014.zip › fig5C -WB/Mg63/Gray value.docx]

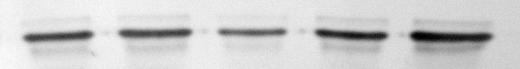
 bcl2


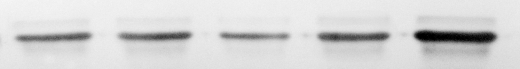
 wnt


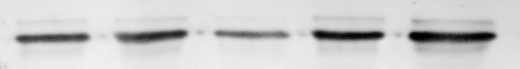
 p-β-catenin


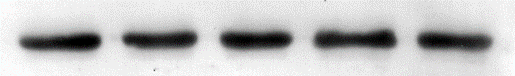
 β-cateninA


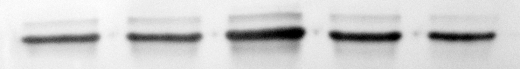
 Bax


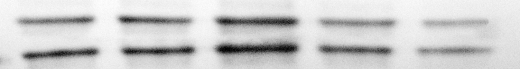
 Caspase3


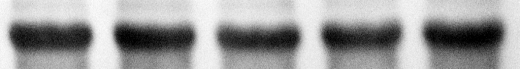
 actin

| No. | con | Si-NC | Si-KIF4A | oe-NC | oei-KIF4A |
| --- | --- | --- | --- | --- | --- |
| bcl2 | 32.63 | 32.72 | 21.58 | 36.56 | 67.74 |
| actin | 108.40 | 108.95 | 104.79 | 104.32 | 110.63 |
| bcl2 t/actin | 0.30 | 0.30 | 0.21 | 0.35 | 0.61 |

| No. | con | Si-NC | Si-KIF4A | oe-NC | oei-KIF4A |
| --- | --- | --- | --- | --- | --- |
| wnt | 30.82 | 32.48 | 19.01 | 33.51 | 65.44 |
| actin | 108.40 | 108.95 | 104.79 | 104.32 | 110.63 |
| wnt/actin | 0.28 | 0.30 | 0.18 | 0.32 | 0.59 |

| No. | con | Si-NC | Si-KIF4A | oe-NC | oei-KIF4A |
| --- | --- | --- | --- | --- | --- |
| p-β-catenin | 38.94 | 40.31 | 22.43 | 44.41 | 65.74 |
| actin | 108.40 | 108.95 | 104.79 | 104.32 | 110.63 |
| p-β-catenin/actin | 0.36 | 0.37 | 0.21 | 0.43 | 0.59 |

| No. | 1 | 2 | 3 | 4 | 5 |
| --- | --- | --- | --- | --- | --- |
| β-cateninA | 45.52 | 42.49 | 47.15 | 51.11 | 49.78 |
| actin | 108.40 | 108.95 | 104.79 | 104.32 | 110.63 |
| β-catenin A/actin | 0.42 | 0.39 | 0.45 | 0.49 | 0.45 |

| No. | con | Si-NC | Si-KIF4A | oe-NC | oei-KIF4A |
| --- | --- | --- | --- | --- | --- |
| Bax | 39.02 | 40.31 | 72.30 | 39.64 | 23.23 |
| actin | 108.40 | 108.95 | 104.79 | 104.32 | 110.63 |
| Bax/actin | 0.36 | 0.37 | 0.69 | 0.38 | 0.21 |

| No. | | con | Si-NC | Si-KIF4A | oe-NC | oei-KIF4A |
| --- | --- | --- | --- | --- | --- | --- |
| Caspase3 | 19 | 31.90 | 32.08 | 51.66 | 32.03 | 17.86 |
|  | 17 | 41.76 | 41.53 | 75.03 | 44.85 | 24.96 |
| actin |  | 108.40 | 108.95 | 104.79 | 104.32 | 110.63 |
| Caspase3/  actin | 19 | 0.29 | 0.29 | 0.49 | 0.31 | 0.16 |
|  | 17 | 0.39 | 0.38 | 0.72 | 0.43 | 0.23 |
