# Supplementary material for: Identification of KIF4A as a pan-cancer diagnostic and prognostic biomarker via bioinformatics analysis and validation in osteosarcoma cell lines
Source: PeerJ. 2021 May 21;9:e11455. doi: 10.7717/peerj.11455 (PMC8142929; doi:10.7717/peerj.11455)
Supplement: Supplemental Information 14 [file peerj-09-11455-s014.zip › fig5C -WB/U2OS/Gray value.docx]

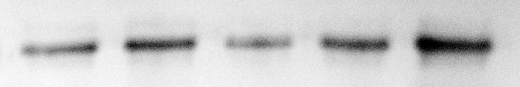
 bcl2


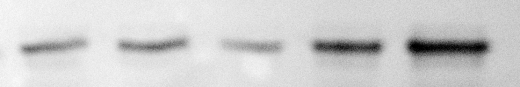
 wnt


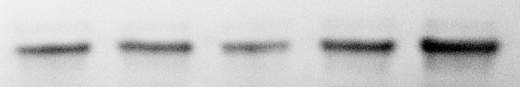
 p-β-catenin


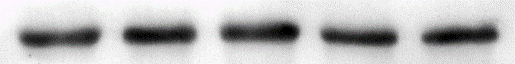
 β-catenin


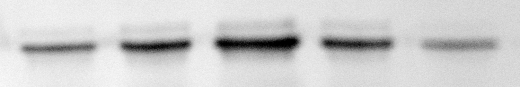
 Bax


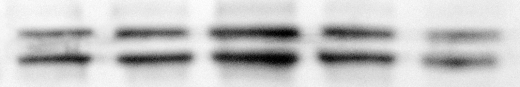
 Caspase3


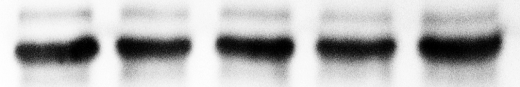
 actin

| No. | con | Si-NC | Si-KIF4A | oe-NC | oei-KIF4A |
| --- | --- | --- | --- | --- | --- |
| bcl2 | 42.68 | 44.15 | 21.53 | 45.75 | 82.83 |
| actin | 94.11 | 92.60 | 97.94 | 94.72 | 104.88 |
| bcl2 t/actin | 0.45 | 0.48 | 0.22 | 0.48 | 0.79 |

| No. | con | Si-NC | Si-KIF4A | oe-NC | oei-KIF4A |
| --- | --- | --- | --- | --- | --- |
| wnt | 34.87 | 37.17 | 24.81 | 34.31 | 76.85 |
| actin | 94.11 | 92.60 | 97.94 | 94.72 | 104.88 |
| wnt/actin | 0.37 | 0.40 | 0.25 | 0.36 | 0.73 |

| No. | con | Si-NC | Si-KIF4A | oe-NC | oei-KIF4A |
| --- | --- | --- | --- | --- | --- |
| p-β-catenin | 36.67 | 37.01 | 20.96 | 38.89 | 76.84 |
| actin | 94.11 | 92.60 | 97.94 | 94.72 | 104.88 |
| p-β-catenin/actin | 0.39 | 0.40 | 0.21 | 0.41 | 0.73 |

| No. | 1 | 2 | 3 | 4 | 5 |
| --- | --- | --- | --- | --- | --- |
| β-catenin | 50.01 | 53.63 | 56.95 | 51.90 | 51.95 |
| actin | 108.73 | 109.46 | 111.68 | 108.14 | 103.90 |
| β-cateninA -2 /actin | 0.46 | 0.49 | 0.51 | 0.48 | 0.50 |

| No. | con | Si-NC | Si-KIF4A | oe-NC | oei-KIF4A |
| --- | --- | --- | --- | --- | --- |
| Bax | 30.73 | 32.58 | 69.38 | 34.24 | 22.83 |
| actin | 94.11 | 92.60 | 97.94 | 94.72 | 104.88 |
| Bax/actin | 0.33 | 0.35 | 0.71 | 0.36 | 0.22 |

| No. | | con | Si-NC | Si-KIF4A | oe-NC | oei-KIF4A |
| --- | --- | --- | --- | --- | --- | --- |
| Caspase3 | 19 | 36.46 | 37.58 | 61.73 | 38.99 | 19.17 |
|  | 17 | 43.83 | 46.46 | 79.82 | 45.38 | 22.27 |
| actin |  | 94.11 | 92.60 | 97.94 | 94.72 | 104.88 |
| Caspase3/  actin | 19 | 0.39 | 0.41 | 0.63 | 0.41 | 0.18 |
|  | 17 | 0.47 | 0.50 | 0.81 | 0.48 | 0.21 |
