# Supplementary material for: Identification of KIF4A as a pan-cancer diagnostic and prognostic biomarker via bioinformatics analysis and validation in osteosarcoma cell lines
Source: PeerJ. 2021 May 21;9:e11455. doi: 10.7717/peerj.11455 (PMC8142929; doi:10.7717/peerj.11455)
Supplement: Supplemental Information 6 [file peerj-09-11455-s006.zip › fig3B-WB/Gray value.doc]

MG63-KIF4A

MG63-β-actin

| 编号 | 1 | 2 | 3 | 4 | 5 |
| --- | --- | --- | --- | --- | --- |
| KIF4A | 24.96 | 25.45 | 13.21 | 27.95 | 50.58 |
| actin | 82.15 | 77.16 | 83.39 | 78.87 | 87.08 |
| KIF4A /actin | 0.3 | 0.33 | 0.16 | 0.35 | 0.58 |

U20S-KIF4A

U20S-β-actin

| 编号 | 1 | 2 | 3 | 4 | 5 |
| --- | --- | --- | --- | --- | --- |
| KIF4A | 25.86 | 35.33 | 15.81 | 22.01 | 41.14 |
| actin | 78.61 | 85.81 | 84.78 | 93.48 | 79.21 |
| KIF4A/actin | 0.33 | 0.31 | 0.19 | 0.28 | 0.52 |
