# Supplementary material for: Identification of KIF4A as a pan-cancer diagnostic and prognostic biomarker via bioinformatics analysis and validation in osteosarcoma cell lines
Source: PeerJ. 2021 May 21;9:e11455. doi: 10.7717/peerj.11455 (PMC8142929; doi:10.7717/peerj.11455)
Supplement: Supplemental Information 14 [file peerj-09-11455-s014.zip › fig5C -WB/U2OS/Gray value.doc]

β-catenin A-2

actin2

| 编号 | 1 | 2 | 3 | 4 | 5 |
| --- | --- | --- | --- | --- | --- |
| β-catenin A-2 | 49.93 | 53.27 | 57.16 | 52.02 | 51.52 |
| actin | 108.73 | 109.46 | 111.68 | 108.14 | 103.90 |
| β-cateninA -2 /actin | 0.46 | 0.49 | 0.51 | 0.48 | 0.50 |
| 第二次 | 0.46 | 0.49 | 0.51 | 0.48 | 0.50 |
| 第三次 | 0.46 | 0.49 | 0.51 | 0.48 | 0.50 |

（注：β-cateninA是MG63细胞系，β-catenin-2A是U205细胞系）
